# Total synthesis of biseokeaniamides A–C and late-stage electrochemically-enabled peptide analogue synthesis†

**DOI:** 10.1039/d0sc03701j

**Published:** 2020-07-30

**Authors:** Yutong Lin, Lara R. Malins

**Affiliations:** Research School of Chemistry, Australian National University Canberra ACT 2601 Australia lara.malins@anu.edu.au

## Abstract

The first total synthesis of cytotoxic cyanobacterial peptide natural products biseokeaniamides A–C is reported employing a robust solid-phase approach to peptide backbone construction followed by coupling of a key thiazole building block. To rapidly access natural product analogues, we have optimized an operationally simple electrochemical oxidative decarboxylation–nucleophilic addition pathway which exploits the reactivity of native C-terminal peptide carboxylates and abrogates the need for building block syntheses. Electrochemically-generated *N*,*O*-acetal intermediates are engaged with electron-rich aromatics and organometallic reagents to forge modified amino acids and peptides. The value of this late-stage modification method is highlighted by the expedient and divergent production of bioactive peptide analogues, including compounds which exhibit enhanced cytotoxicity relative to the biseokeaniamide natural products.

## Abstract 2

A late-stage electrochemical decarboxylation enables rapid access to structural analogues of biseokeaniamides A–C, cytotoxic lipopeptide natural products.

## Graphical abstract

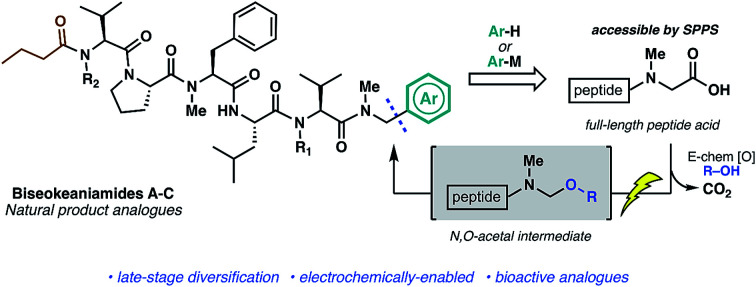

## Introduction

The rich structural diversity of peptide natural products extends well beyond the canonical amino acids to the incorporation of modified residues and rigidifying elements—motifs which are often crucial to bioactivity. Replicating and expanding on Nature's chemical arsenal is an aspirational goal of synthetic chemistry, and an endeavor further fuelled by the growing importance of peptide natural products as lead structures for therapeutic development.^1^ Nevertheless, the optimization of strategies for the direct, late-stage chemical modification of complex peptides is challenging, particularly methods which exploit proteinogenic functionalities for the mild and selective diversification of native peptide substrates.^2^

**Scheme 1:**
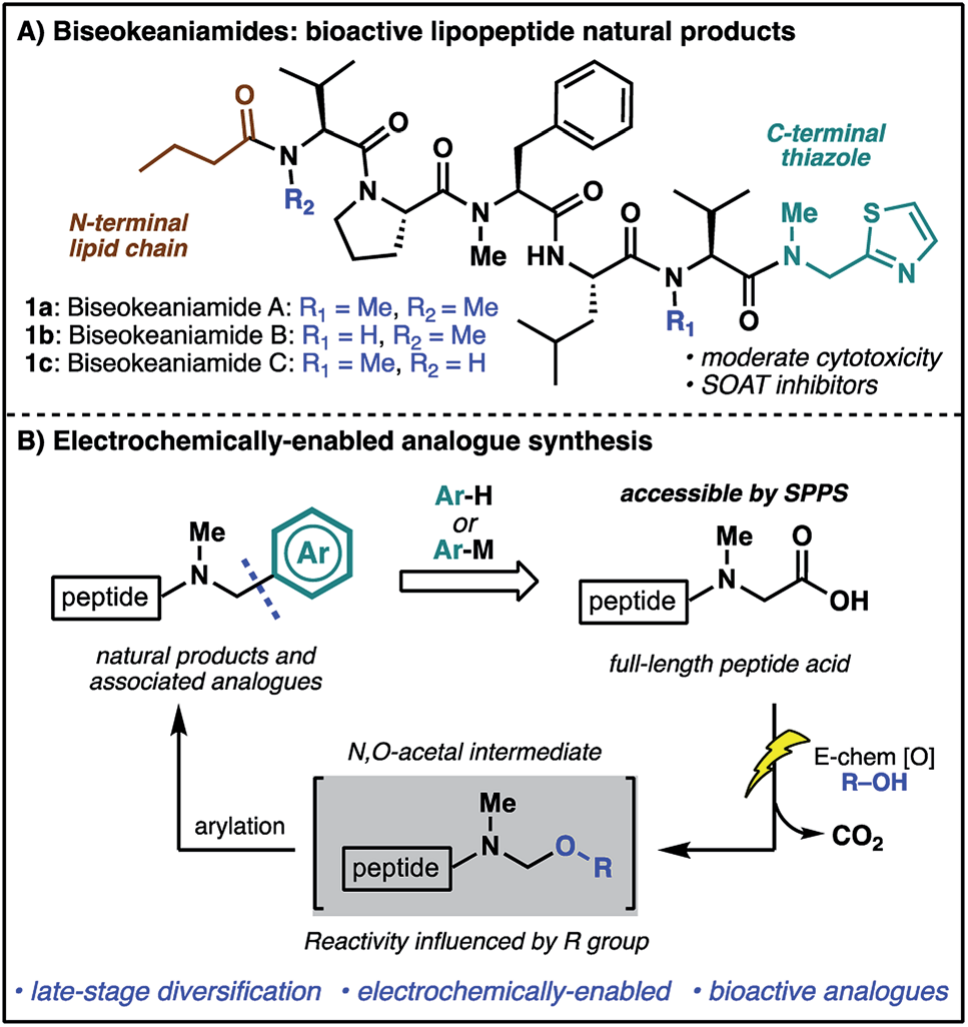
(A) The biseokeaniamide natural products; (B) an electrochemical approach to late-stage peptide modification for the rapid synthesis of natural product analogues.

In our efforts to develop new strategies for the late-stage modification of peptides, we have become particularly interested in C-terminal modifications owing to the fundamental importance of C-terminal composition to peptide and protein bioactivity.^3^ The prospect of exploiting the ubiquitous C-terminal peptide carboxylate motif for direct access to differentially functionalized peptide products provides additional impetus for the development of new synthetic tools.^4^ In the course of these endeavors, we identified marine cyanobacterial natural products biseokeaniamides A–C (**1a–c**, Scheme 1A) as promising targets for methodology development. Isolated from *Okeania* sp. cyanobacterium in 2017,^5^ these lipopeptides feature an intriguing C-terminal thiazole motif as well as an N-terminal lipid chain and a heavily *N*-methylated backbone. The analogues vary only in their pattern of *N*-methylation, thus offering additional opportunities for probing the effects of *N*-methylation on peptide conformation and hydrophobicity, important features in overcoming the conventional liabilities of peptide drugs (*e.g.* poor bioavailability and membrane permeability).^6^ Importantly, compounds **1a–c** also exhibit moderate cytotoxicity and were shown to inhibit sterol *O*-acyltransferase (SOAT), an enzyme which mediates the esterification of cholesterol and is implicated in hypercholesterolemia and atherosclerosis—disease states which involve the accumulation of cholesterol esters. The novel structural features together with opportunities to probe structure–activity relationships of natural product analogues prompted the undertaking of a synthesis campaign. Our goals were two-fold: (1) complete the first total synthesis of biseokeaniamides A–C; (2) explore new methods for late-stage, C-terminal analogue synthesis which directly exploit the reactivity of C-terminal carboxylic acids. Herein, we disclose the realization of these endeavors through an efficient solid-phase approach to biseokeaniamides A–C and the development of a strategy for C-terminal peptide modification which leverages a key electrochemical oxidative decarboxylation step to generate reactive *N*,*O*-acetals capable of engaging a diverse array of nucleophiles (Scheme 1B). This direct strategy for the diversification of C-terminal peptide acids is applied to peptide substrates and biseokeaniamide analogues, leading to a collection of natural product derivatives, including those which exhibit enhanced cytotoxic activity relative to the natural products.

## Results and discussion

### First total synthesis of biseokeaniamides A–C

**Scheme 2:**
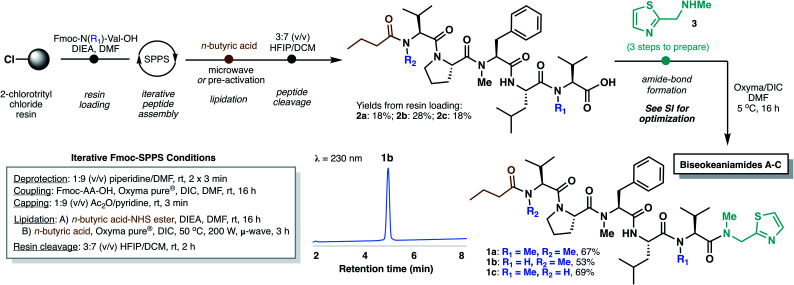
Total synthesis of biseokeaniamides A–C employing iterative Fmoc-SPPS followed by coupling of thiazole building block **3**.

In designing a synthetic approach to the biseokeaniamides, we envisioned that the robust nature of solid-phase peptide synthesis (SPPS) could be exploited to rapidly construct the peptide backbone structure. A handful of other C-terminal thiazole-containing peptide natural products have been isolated from marine organisms (*e.g.* cytotoxic peptides dolastatin 10 ^7^ and symplostatin 1,^8^ barbamide,^9^ lyngbyapeptins A,^10^ B and C,^11^ the highly backbone *N*-methylated apramides A–G,^12^ micromide,^13^ and virenamides A–E^14^) and several have been the subject of recent total synthesis campaigns.^13,15^ However, to the best of our knowledge, none have exploited a solid-phase approach to peptide backbone construction. Since conventional solid-phase methods require immobilization of the C-terminal peptide acid and elongation in the *C*- to *N*-direction, incorporation of the terminal thiazole motif must necessarily occur after resin cleavage. As such, we envisioned a rapid, first-generation approach to the biseokeaniamides could be accomplished through the synthesis of truncated acid derivatives **2a–2c** followed by coupling of a preformed thiazole building block **3** (Scheme 2).

Accordingly, Fmoc-Val-OH and Fmoc-Me-Val-OH were first loaded onto the highly acid-labile 2-chlorotrityl chloride resin to account for the differential backbone methylation patterns of biseokeaniamides A and C (R_1_ = Me) and biseokeaniamide B (R_1_ = H). The peptides were elongated using manual Fmoc-SPPS. Due to the heavily *N*-methylated backbone structures of the biseokeaniamides and the preponderance of bulky hydrophobic residues (*e.g.* Val, Leu), each coupling was carried out for 16 h using the highly activating coupling combination of Oxyma Pure® and DIC.^16^ Lower yields and incomplete couplings were observed with alternative reagents (*e.g.* PyBOP). The N-terminal *n*-butyric acid lipid tail was coupled on-resin using Oxyma/DIC under microwave irradiation (when R_2_ = Me) or through the coupling of a preactivated *n*-butyric acid-NHS ester (when R_2_ = H) (see ESI† for details). Liberation of peptides **2a–2c** from the resin was accomplished by treatment with HFIP in DCM, affording the truncated natural products in 18–28% isolated yield (based on the original resin loadings) following reverse-phase HPLC purification.

Thiazole **3** was prepared in three steps according to literature methods,^13^ and a variety of coupling conditions and reactant stoichiometries were screened with truncated biseokeaniamide C peptide **2c** in order to reduce epimerization—a notable drawback of peptide elongation in the *N*- to *C*-direction. Standard protocols involving preactivation of the C-terminal Val residue in **2c** with Oxyma/DIC and subsequent treatment with excess thiazole **3** led to complete loss of stereochemical integrity at the Val α-position (see ESI†). However, optimization of reaction temperature (5 °C) and elimination of the preactivation step preferentially afforded the natural product over the corresponding d-Val epimer (d.r. = 85 : 15). With optimal conditions in hand, thiazole **3** was coupled to precursor peptides **2a–2c**, to afford the natural products **1a–1c** in 53–69% yield following HPLC purification, which enabled facile removal of the minor diastereomer. Importantly, spectral data for the three compounds is fully consistent with that reported for the original isolates (see ESI†).^5^

### Devising a strategy for C-terminal modification

Although our optimized methods enabled robust access to the natural products, the inability to fully suppress epimerization in the coupling of the terminal thiazole was a powerful motivation for further exploration of late-stage installation protocols which would circumvent the need for peptide extension in the *N*- to *C*-direction. Existing syntheses of C-terminal thiazole-containing natural products have largely exploited building block approaches, particularly in cases where the thiazole component bears defined α-chirality.^15^ As the biseokeaniamides feature a sarcosine (*N*-methyl glycine)-derived terminal thiazole, the preservation of α-chirality upon incorporation of the aryl motif is not a prerequisite, thus opening additional opportunities for late-stage installation. Such an approach would avoid the preparation of a discrete thiazole building block and open new vistas for the divergent functionalization of a common peptide precursor, facilitating rapid access to natural product analogues. This attractive late-stage functionalization logic has been a prominent feature of several recent approaches to modified peptides.^2^

To this end, our attention first turned to the feasibility of direct thiazole incorporation using decarboxylative cross-coupling chemistry—a robust approach to C–C bond formation,^17^ including in the diversification of peptides.^18^ Preparation of a biseokeaniamide precursor (bearing a carboxylic acid in place of the C-terminal thiazole motif, see **4**Scheme 5B, *vide infra*) and activation as the corresponding redox-active ester^19^ were followed by treatment with various nickel catalysts and organothiazole derivatives. Unfortunately, in our hands, attempts at decarboxylative arylation were unsuccessful, likely owing to the thermal instability of the thiazole-derived organozinc reagent.^20^ Nevertheless determined to exploit the C-terminal carboxylate functionality, we were encouraged by initial reports from Seebach and coworkers disclosed in the late 1980s describing the electrochemical oxidative decarboxylation of amino acids and small peptides in the presence of methanol to forge *N*,*O*-acetals (*e.g.* Boc-Ala-*N*(Me)-methoxymethyl acetal, derived from Boc-Ala-Sar-OH and MeOH).^21^ Acetal intermediates could be engaged (*via in situ* formation of the corresponding *N*-acyliminium) with various nucleophiles, including phosphites,^22^ allylsilanes, and simple Grignard reagents.^21^ Notably, in recent years electrochemical transformations have attracted considerable attention as mild, tunable, and sustainable complements to conventional synthetic chemistry.^23^ However, there remains remarkably few examples of the exploitation of anodic oxidation on *peptide substrates* aside from electrolyses of cyclic dipeptide^24^ and β-lactam derivatives^25^ and seminal work leveraging Shono-type oxidations of peptide analogues bearing electroauxiliaries.^26^ We therefore envisioned that broader application of electrochemically-generated peptide *N*,*O*-acetals—as common intermediates for the divergent synthesis of natural product analogues—would be a valuable addition to the existing toolbox of late-stage peptide modifications.

### Electrochemical decarboxylation of a sarcosine model

**Scheme 3:**
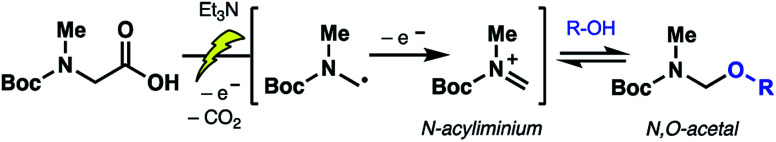
Proposed mechanism of the electrochemical oxidative decarboxylation to forge *N*,*O*-acetal intermediates.

To probe the feasibility of the proposed electrochemical oxidative decarboxylation and evaluate the scope of suitable nucleophiles, we first examined the reactivity of Boc-sarcosine (**5**) as a model substrate (Table 1). Compound **5** was dissolved in methanol and treated with triethylamine (15 mol%) for *in situ* generation of the electrolyte.^21*b*^ Electrolysis was performed at constant current (8 mA) in an undivided electrochemical cell equipped with carbon electrodes (see ESI†). Mechanistically, this non-Kolbe electrolysis likely proceeds through an initial oxidation of the carboxylate with concomitant loss of CO_2_ to afford a stabilized α-nitrogen radical. A second oxidation affords the *N*-acyliminium, which is trapped by the solvent methanol (Scheme 3).^21*b*^ Accordingly, concentration of the crude electrolysis reaction afforded the methanol *N*,*O*-acetal **6a**. As attempts to purify **6a** on silica gel led to decomposition, the material was used crude in subsequent transformations.

**Table 1:** Probing the electrochemical generation and reactivity of *N*,*O*-acetals

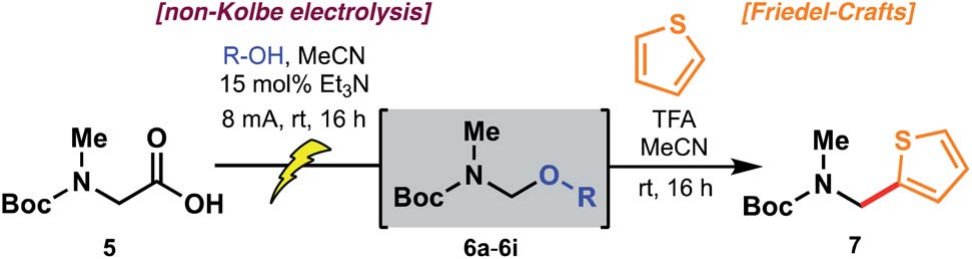				
Entry	R =	p*K*_a_ (R–OH)^27,28^	*N*,*O*-Acetal	Yield of **7**^a^
1	Me^b^	15.5	**6a**	n.d. (46%^c^)
2	CF_3_CH_2_^d^	12.5	**6b**	53%
3	(CF_3_)_2_CH^d^	9.3	**6c**	69%
4	Ac^d^	4.76	**6d**	65%
5	Bz	4.2	**6e**	71%
6	CHO	3.77	**6f**	47%
7	ClCH_2_CO	2.86	**6g**	73%
8	Cl_2_CHCO	1.29	**6h**	66%
9	Cl_3_CCO	0.85	**6i**	11%

a Yield determined by ^1^H NMR using dibromomethane as an internal standard, 0.1 mmol scale; n.d. = not determined.

b Neat methanol used as solvent.

c Friedel–Crafts reaction under μ-wave irradiation (50 °C), isolated yield (see ESI for details).

d 0.05 mmol scale.

The reactivity of the methanol-derived *N*,*O*-acetal **6a** was examined using a Friedel–Crafts-type reaction with thiophene in the presence of TFA (2.0 equiv.), which serves to regenerate the *N*-acyliminium for nucleophilic attack. Interestingly, under these reaction conditions, no arylated product **7** was observed (Table 1). We hypothesized that *N*,*O*-acetal **6a** was not sufficiently reactive at room temperature to generate the requisite *N*-acyliminium under the conditions employed, or that methanol was outcompeting thiophene as a nucleophile leading to reversible generation of the starting *N*,*O*-acetal. However, repeating the reaction under microwave irradiation (50 °C) led to productive formation of thiophene **7**. As an alternative approach to enhancing reactivity at room temperature, we reasoned that electrolysis in the presence of alcohols with lower p*K*_a_ values^27^ (*e.g.* better leaving groups) might afford *N*,*O*-acetals with superior reactivity in the arylation reaction (see Table 1) owing to more facile formation of the key *N*-acyliminium intermediate (see Scheme 3). Pleasingly, the novel trifluoroethanol (TFE) (p*K*_a_ = 12.5)^28^-derived acetal **6b** reacted with thiophene at ambient temperature to afford **7** in 53% yield over the two steps (entry 2). Electrolysis in the presence of hexafluoroisopropanol (HFIP) (p*K*_a_ = 9.3)^28^ afforded *N*,*O*-acetal **6c**, which proceeded to product **7** in 69% yield over the two steps (entry 3). To further probe the correlation between enhanced yield and leaving group ability of intermediate *N*,*O*-acetals, various carboxylic acids (*e.g.* acetic acid, entry 4; benzoic acid, entry 5; formic acid, entry 6; and chloroacetic acid derivatives, entries 7–9) were also screened. While p*K*_a_ and yield were not correlated in all cases, owing to the volatility of certain intermediate *N*,*O*-acetals (*e.g.***6f**, R = CHO) and the subtle interplay between enhanced reactivity and a tendency toward competitive hydrolysis (*e.g.***6i**, R = Cl_3_CCO), it is notable that the highest overall yield (73%) was obtained with chloroacetic acid (entry 7, p*K*_a_ = 2.86 ^27^). Considering both operational simplicity and ease of handling, MeOH, TFE, HFIP, and acetic acid-derived acetals **6a–6d** were identified as the most viable *N*,*O*-acetal intermediates in subsequent chemical transformations.

**Scheme 4:**
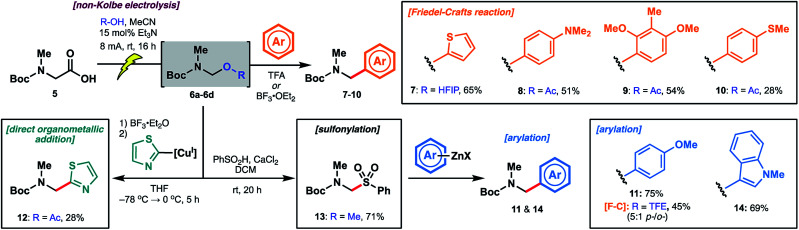
Electrolysis of Boc-Sar-OH to generate *N*,*O*-acetal intermediates followed by diversification with various classes of nucleophiles.

Encouraged by initial results with thiophene, we explored several additional electron-rich aromatic nucleophiles in Friedel–Crafts reactions. Using acetic acid-derived *N*,*O*-acetal **6d**, dimethylaniline (**8**), 2,6-dimethoxytoluene (**9**) and thioanisole (**10**)-derived carbamates were accessible in moderate yields (Scheme 4). Anisole **11** afforded a mixture of *ortho*/*para*-substituted products in 45% yield *via* the TFE-derived *N*,*O*-acetal **6b**. In the interest of expanding the scope of arylation chemistry beyond electron-rich aromatic substrates, we next probed direct organometallic addition to the acetal intermediates. Although Grignard additions to amino acid-derived *N*,*O*-acetals have been explored,^21*a*^ the scope of organometallic reagents (*e.g.* methylmagnesium chloride, cyclohexylmagnesium bromide) is limited. Inspired by the structure of the biseokeaniamides, we focused our attention on the addition of thiazole-derived organometallic nucleophiles so as to devise an alternative approach to the late-stage installation of C-terminal thiazoles. After extensive optimization, including screening of various *N*,*O*-acetals, we were able to obtain thiazole derivative **12** in a modest 28% yield over the two steps. The optimal method employed BF_3_·OEt_2_ for the generation of the reactive *N*-acyliminium at −78 °C followed by the addition of thiazole-2-cuprate, generated *in situ* from the corresponding organo-lithium reagent. Notably, treatment of **12** with TFA led to facile deprotection of the Boc group, providing an alternative route to key building block **3** (see ESI†). Sulfonylation of the original methanol *N*,*O*-acetal **6a** likewise afforded **13** in 71% yield and provided an indirect approach to organometallic addition.^29^ It is intriguing that room temperature activation of **6a** was possible under the sulfonylation conditions (PhSO_2_H, CaCl_2_) given the recalcitrance of **6a** to TFA-promoted Friedel–Crafts reaction with thiophene—an observation which suggests that, in addition to p*K*_a_, the method of activation is an important determinant of acetal reactivity. Engaging intermediate sulfone **13** with arylzinc reagents readily forged the target arylated products, including anisole derivative **11** (obtained as a single regioisomer and in higher yield than the corresponding Friedel–Crafts approach) as well as *N*-methylindole **14**. The instability of the thiazole zinc reagent at room temperature, however, precluded the application of this method to the synthesis of thiazole derivative **12**.

### Late-stage modification of a model tetrapeptide

**Scheme 5:**
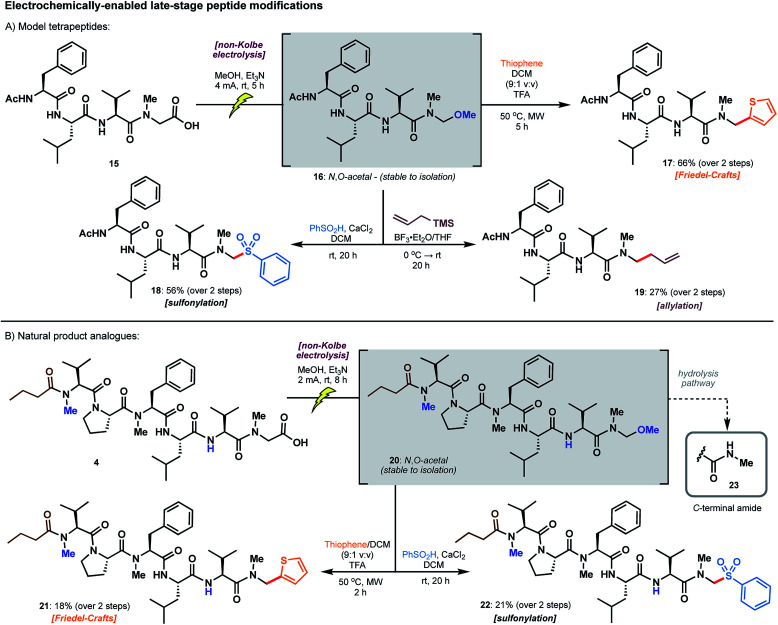
Late-stage peptide modifications employing: (A) model tetrapeptide **15** and (B) biseokeaniamide peptide carboxylic acid derivative **4**.

The utility of model *N*,*O*-acetals as divergent precursors to arylated products **7–14** next prompted extension of the chemistry to model peptide substrates. *N*-acyl tetrapeptide **15**, featuring a C-terminal sarcosine residue, bears structural similarity to the biseokeaniamides and served as a practical model for the screening of conditions (Scheme 5A). Remarkably, electrolysis in the presence of methanol under the conditions previously described led to an *N*,*O*-acetal intermediate **16** which was stable to isolation following HPLC purification; accordingly, the structure of **16** was unambiguously characterized, confirming selective oxidation of the C-terminal carboxylate. As competitive hydrolysis of more reactive *N*,*O*-acetals (derived from electrolysis in solvents other than methanol) occurred, crude peptide *N*,*O*-acetals were used in the screening of subsequent reactions for late-stage modification. Intriguingly, thiophene addition was most successful with the methanol-derived acetal **16** under microwave irradiation conditions (50 °C) in the presence of excess TFA, affording **17** in 66% yield over the two steps from acid **15** (Scheme 5A). Despite the favorable reactivity of more activated *N*,*O*-acetals in the model sarcosine study (see Table 1), employing acetic acid and chloroacetic acid-derived tetrapeptide acetals did not lead to improvements in yield. Notably, direct access to thiophene product **17** is not otherwise possible using existing literature methods, and a hypothetical building block approach to the incorporation of a C-terminal *N*-methylamino thiophene unit would likely be plagued by epimerization. Tetrapeptide **17** is also the first example of a C-terminal thiophene peptide, rendering this chemistry a valuable addition to the menu of late-stage modifications.

*N*,*O*-Acetal **16** was further amenable to sulfonylation^29^ under conditions similar to those employed for the model amino acid substrate, leading to arylsulfone **18** in 56% yield over two steps. Unfortunately, replication of the thiazole cuprate addition (see **12**, Scheme 4) was unsuccessful on the peptide substrate. However, reminiscent of work by Seebach on the allylation of small peptide *N*,*O*-acetals,^21^ we were able to access terminal alkene **19** upon treatment with allylsilane and BF_3_·OEt_2_ (27% over two steps). In each of these transformations, hydrolysis of the intermediate *N*,*O*-acetal to afford the C-terminal *N*-methyl amide was a frequently observed byproduct (see ESI†), particularly in the presence of Lewis acid activators. This side pathway was enhanced with more reactive *N*,*O*-acetals (*e.g.* acetic acid and chloroacetic acid derivatives), even when employing anhydrous reaction conditions, thus providing key rationale for the preferential use of the methanol-derived *N*,*O*-acetal in peptide-based systems.

### Preparation of natural product analogues

The late-stage decarboxylative modifications were finally applied to the biseokeaniamide scaffold to generate analogues of the natural products (Scheme 5B). Peptide acid **4** bearing a C-terminal sarcosine residue, and derived directly from solid-phase peptide synthesis on 2-chlorotrityl chloride resin (see ESI†), was electrolysed in the presence of methanol to afford *N*,*O*-acetal **20**, which was once again stable to isolation. Taken crude, treatment of **20** under the optimized conditions devised for the diversification of the model tetrapeptide afforded thiophene **21** and sulfone **22** in synthetically useful yields (*ca.* 20%) over the two steps. Although competitive hydrolysis (see **23**, Scheme 5B)^30^ was observed as a common byproduct, the protocols nevertheless abrogate the need for the synthesis of various pre-functionalized building blocks and avoid all complications with epimerization. These electrochemically-enabled approaches therefore expedite the production of diverse natural product analogues by exploiting the inherent reactivity of accessible C-terminal peptide carboxylates.

### Evaluation of cytotoxicity

**Fig. 1:**
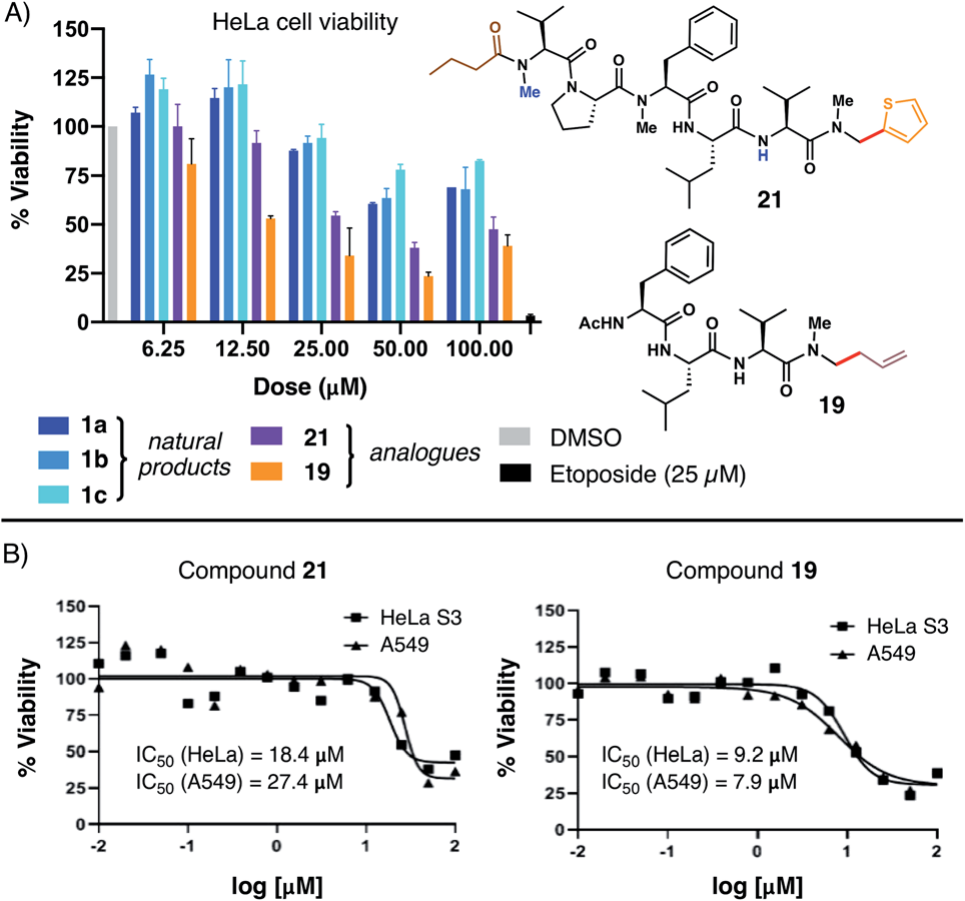
(A) Comparative HeLa cell viability assays employing **1a–1c** and analogues **21** and **19**; (B) dose–response curves for compounds **21** and **19***versus* HeLa and A549 cells.

With several natural product analogues in hand, we ultimately proceeded to probe the cytotoxicity of our compounds relative to the biseokeaniamide natural products. Tetrapeptides (**15–19**), natural product analogues (**21**, **22**) and their precursors (*e.g.* acid derivatives and *N*,*O*-acetal intermediates), as well as peptide hydrolysis byproducts were screened in cell viability assays against HeLa and A549 cancer cell lines (see ESI† for details) and compared to the natural products **1a–1c**. Notably, thiophene natural product derivative **21** and tetrapeptide **19** both exhibited enhanced cytotoxicity in relation to the natural products (Fig. 1A), suggesting that C-terminal modification may play an important role in optimizing bioactivity. Compound **19** in particular had observed IC_50_ values in the low micromolar range against both cancer cell lines (Fig. 1B). Further structural modifications may deliver additional analogues with enhanced therapeutic capacity. Given the relative hydrophobicity of the peptides, future work will focus in particular on the effect of *N*-methylation and C-terminal modifications on peptide conformation and membrane permeability. Likewise, in light of the observed activity of **1a–1c** as SOAT inhibitors, evaluation of the peptide analogues as anti-atherosclerotic agents will be the subject of subsequent studies.

## Conclusions

Herein we have disclosed the first total synthesis of cyanobacterial natural products biseokeaniamides A–C, structurally unique lipopeptides bearing a C-terminal thiazole motif. A solid-phase approach to the construction of the highly *N*-methylated peptide backbone was employed followed by installation of a preformed *N*-methyl amino thiazole building block to enable a rapid approach to the peptides. Motivated by the resilience of the solid-phase approach and a desire to overcome challenges with epimerization upon building block incorporation, we examined non-Kolbe electrolysis as a means of direct decarboxylative C-terminal peptide modification. Harnessing the reactivity of key *N*,*O*-acetal intermediates forged *via* electrochemical decarboxylation, we accomplished the divergent synthesis of various model sarcosine derivatives using Friedel–Crafts reactions, direct organometallic addition and sulfonylation chemistry. These methods were readily extendable to model peptides and natural product precursors to deliver C-terminally modified peptides from native, unactivated peptide substrates in an epimerization-free manner and without the need for lengthy building block syntheses. In some cases, peptide analogues exhibited improved cytotoxicity relative to the natural products, showcasing the value of these late-stage modification strategies for the efficient syntheses of bioactive peptides. Given that C-terminal peptide carboxylic acids are directly accessible using solid-phase peptide synthesis, we envisage that this proof-of-principle study will find widespread application in the late-stage modification of peptides.

## Conflicts of interest

The authors declare no competing financial interests.

## Supplementary Material

SC-011-D0SC03701J-s001
