# Supplementary material for: Total synthesis of biseokeaniamides A–C and late-stage electrochemically-enabled peptide analogue synthesis
Source: Chem Sci. 2020 Jul 30;11(39):10752–8. doi: 10.1039/d0sc03701j (PMC8162360; doi:10.1039/d0sc03701j)

Electronic Supplementary Material (ESI) for Chemical Science.
This journal is © The Royal Society of Chemistry 2020

Supporting Information

Total Synthesis of Biseokeaniamides A-C and Late-Stage Electrochemically-Enabled Peptide Analogue Synthesis

Yutong Lin, Lara R. Malins*
*Email: lara.malins@anu.edu.au

Table of contents

TABLE OF CONTENTS 2
GENERAL METHODS 3
GENERAL PROCEDURES FOR THE TOTAL SYNTHESIS OF BISEOKEANIAMIDES A-C 5
COMPARISON OF ISOLATED AND SYNTHETIC NATURAL PRODUCTS (1D NMR) 16
EXPERIMENTAL PROCEDURES: SARCOSINE MODEL 25
EXPERIMENTAL PROCEDURES: TETRAPEPTIDE MODEL 33
EXPERIMENTAL PROCEDURES: NATURAL PRODUCT ANALOGUES 39
EVALUATION OF BIOLOGICAL ACTIVITY 46
REFERENCES 48
NMR SPECTRAL DATA 49

General methods

Melting points were measured using an SRS Optimelt automated melting point system and were uncorrected. NMR spectra were recorded on Bruker AVANCE 400 MHz, 600 MHz and 700 MHz instruments and were calibrated using residual undeuterated solvent ($CHCl_3$ at 7.26 ppm ^{1}H NMR, 77.16 ppm ^{13}C NMR; CH_3OH at 3.31 ppm ^{1}H NMR, 49.00 ppm ^{13}C NMR). ^{19}F NMR spectra were recorded using 2,2,2-trifluoroethanol (δ –77.80 ppm) as internal standard. ^{1}H NMR data were recorded as follows: chemical shift δ (ppm) [multiplicity, coupling constant(s) *J* (Hz), relative integral] where multiplicity was defined as: s = singlet; d = doublet; t = triplet; q = quartet; m = multiplet; br = broad or combinations of the above. 2D NMR (COSY, HSQC, HMBC) were conducted to assist assignment when necessary. Isolated yields refer to chromatographically and spectroscopically homogeneous material. ^{1}H NMR yields, if specified, were calculated using dibromomethane as an internal standard. Infrared spectra were recorded on a Perkin-Elmer UATR Two spectrometer as a thin film or solid.

Low-resolution mass spectrometry (LRMS) and high-resolution mass spectrometry (HRMS) were performed using positive electrospray ionisation (ESI^+) on a Micromass ZMD ESI-Quadrupole, a Waters LCT Premier XE, a Thermo-Fischer Scientific Orbitrap Elite™ Hybrid Ion Trap-Orbitrap or an Orbitrap QExactive mass spectrometer. UV-Vis absorbance was measured using a Shimadzu UV-2450 spectrophotometer. Preparative HPLC was performed on a Waters Alliance Separation Module 2690, with a Waters 996 photodiode array detector, the system was operated using Empower 3 software. All separations employed linear gradients (unless otherwise specified) of water containing 0.1% trifluoroacetic acid and MeCN containing 0.1% trifluoroacetic acid at a constant flow rate of 10 mL/min (preparative HPLC, Alltima, C18, 5 μm; 22 × 250 mm) or 5 mL/min (semi-preparative HPLC, Luna, C18, 5 μm; 10 × 250 mm). UPLC was performed on a Waters Acquity system outfitted with a Waters UV Detector. Separations employed linear gradients (otherwise specified) of water containing 0.1% formic acid (Solvent A) and MeCN containing 0.1% formic acid (Solvent B) at a constant flow rate (0.2 – 0.4 mL/min, BEH, C18, 1.7 μm). Analytical thin layer chromatography (TLC) was performed on aluminum-backed Merck silica gel 60 F_{254} plates. Eluted plates were visualized using a 254 nm UV lamp and/or by treatment with a suitable dip followed by heating. These dips included phosphomolybdic acid: ceric sulfate: sulfuric acid (conc.): water (37.5 g: 7.5 g: 37.5 g: 720 mL) or potassium permanganate: potassium carbonate: 5% sodium hydroxide aqueous solution: water (3 g: 20 g: 5 mL: 300 mL). Flash chromatographic separations were carried out with Merck silica gel 60 (40 – 63 μm). THF, Et_2O, and DCM were dried using a Glass Contour solvent purification system. Solvent compositions were mixed in v/v as specified. All solvents and reagents were used as supplied unless otherwise specified.

The mass of all peptide compounds was determined after transfer to a 1.5 mL Eppendorf® tube and following lyophilization, with a calibrated 6-decimal point balance. All microwave reactions were performed using a CEM Discovery instrument, with continuous irradiation power from 0 to 200 W utilizing the standard absorbance level of 300 W maximum power. The reactions were carried out in a 10 mL sealed vessel equipped with a magnetic stirrer. Disposable solid-phase reaction vessels and pressure caps were purchased from Torviq. A Heidolph Rotamax 120 platform shaker (operating at 100 rpm) was used for the general mixing and agitation of solid-phase reactions. IKA® Electrasyn 2.0 was utilized as the potentiostat in all the electrolysis reactions, with complete vials 5 mL, 10 mL, 20 mL as required. The instrument was purchased as the complete ElectraSyn 2.0 Starter Package, ID # 0020008980.

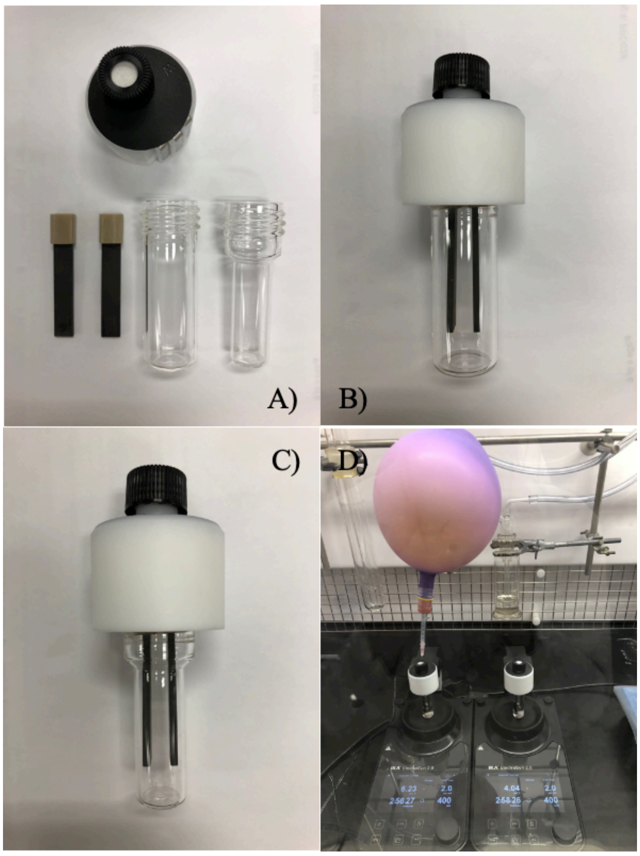

A) Dissected vial and carbon electrodes; B) 10 mL complete vial;
C) 5 mL complete vial; D) reaction set-up (Left: anhydrous; Right: non-anhydrous).

General procedures for the total synthesis of biseokeaniamides A-C

Compound **3**

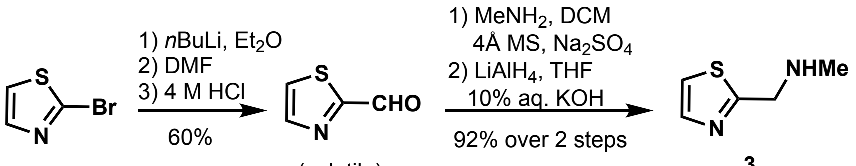

Thiazole-2-carbaldehyde

A round-bottom flask was charged with 2-bromothiazole (1.64 g, 10 mmol, 1.0 equiv.) and Et_2O (10 mL) was added under an atmosphere of argon. The solution was cooled to –78 °C, then *n*BuLi/hexane solution (1.26 M, 10.9 mL, 1.2 equiv.) was added dropwise. The reaction was stirred at –78 °C for 0.5 h. *N,N*-dimethylformamide (DMF) (1.23 mL, 16 mmol, 1.6 equiv.) was added to the flask slowly and the mixture was warmed to –40 °C. After stirring for an additional 1.5 h at –40 °C, the reaction mixture was warmed to 0 °C and quenched with aqueous 4 M HCl. The two layers were separated and the organic layer was extracted with aqueous 4 M HCl. The combined aqueous layer was neutralized with anhydrous K_2CO_3, and extracted with Et_2O (3 × 20 mL). The combined organic layers were dried over $MgSO_4$ and concentrated under reduced pressure. The volatile aldehyde product, thiazole-2-carbaldehyde, was subjected to the following transformation without further purification.

***N*-methyl-1-(thiazol-2-yl)methanamine** (Compound **3**)

To a flask containing 2-formylthiazole (113 mg, 1.0 mmol, 1.0 equiv.), Na_2SO_4 (1.42 g, 10 mmol, 10.0 equiv.) and 4 Å molecular sieves was added methylamine (33 wt% solution in EtOH, 8 mmol, 8.0 equiv., 1.0 mL) and dry DCM (15 mL). The reaction mixture was stirred at room temperature for 16 h. Upon completion, the reaction mixture was filtered to remove the Na_2SO_4 and molecular sieves and the filtrate was concentrated under reduced pressure.

The crude imine (223 mg, 1.77 mmol, 1.0 equiv.) was dissolved in dry THF (10 mL). The solution was cooled to 0 °C, and then treated with $LiAlH_4$ powder (94 mg, 2.5 mmol, 1.4 equiv.). The reaction was monitored by TLC, and upon complete consumption of the imine, a small portion of 10% aqueous KOH solution was added and the mixture left to stir at 0 °C for 0.5 h. $MgSO_4$ was added and the reaction mixture was filtered through Celite®. The filtrate was concentrated under reduced pressure to give the desired product in 92% yield (208 mg) over 2 steps.

Physical State: yellow oil.
^{1}H NMR (600 MHz, $CDCl_3$): δ 7.72 (d, *J* = 3.1 Hz, 1H), 7.27 (d, *J* = 3.3 Hz, 1H), 4.10 (s, 2H), 2.53 (s, 3H), 1.79 (br, 1H).
HRMS (ESI-TOF): calc'd for $C_5H_9N_2S$ $[M+H]^+$: 129.0481, found: 129.0480.

Spectral data matches the literature.[1]

Activation of *n*Butyric acid:

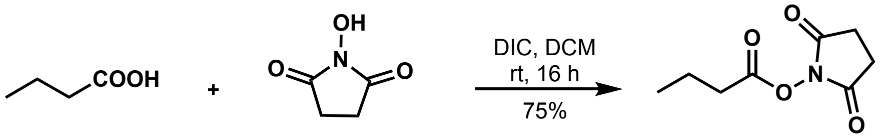

2,5-Dioxopyrrolidin-1-yl butyrate

Butyric acid (1.0 mL, 10.9 mmol, 1.0 equiv.) and *N*-hydroxysuccinimide (1.38 g, 12.0 mmol, 1.1 equiv.) were dissolved in DCM (22 mL, 0.5 M). *N,N'*-diisopropylcarbodiimide (DIC) (1.77 mL, 11.3 mmol, 1.04 equiv.) was added dropwise. The solution was stirred at room temperature for 16 h. Upon completion, the reaction mixture was concentrated under reduced pressure and directly subjected to column chromatography (silica gel, 20:1 DCM/MeOH) to yield the title compound (1.51 g, 75% yield).

Spectral data matches the literature.[2]

General Scheme for Solid-Phase Peptide Synthesis (SPPS):

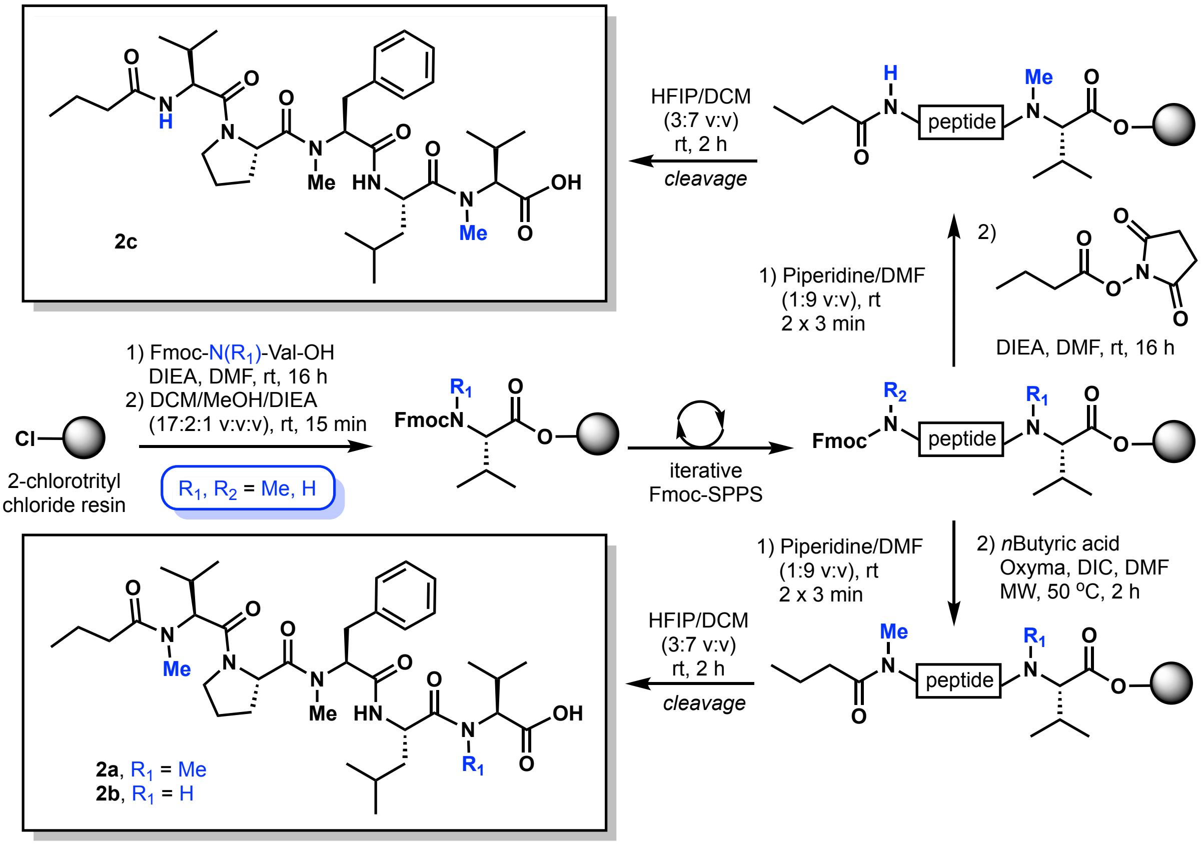

Loading 2-chlorotrityl chloride resin – Coupling of Fmoc-N(R_1)-Val-OH [R = H or Me]

2-Chlorotrityl chloride resin (1.0 equiv., substitution = 1.4 mmol/g) was swollen in DCM for 30 min then washed with DCM (5 × 3 mL) and DMF (5 × 3 mL). A solution of the Fmoc-Val-OH or Fmoc-N(Me)-Val-OH (4.0 equiv.) and *N*,*N*-diisopropylethylamine (DIEA, 8.0 equiv.) in DMF (final concentration 0.1 M) was added to the resin (1.0 equiv.) and agitated at room

temperature. After 16 h, the resin was washed with DMF (5 × 3 mL), DCM (5 × 3 mL), and DMF (5 × 3 mL).

Capping: A solution of DCM/MeOH/DIEA (17:2:1 v:v:v) was added to the resin. After 15 min the resin was washed with DMF (5 × 3 mL), DCM (5 × 3 mL) and DMF (5 × 3 mL). The resin-bound residue was submitted to iterative peptide assembly (Fmoc-SPPS).

The loading efficiency was evaluated through treatment of the resin with 10% piperidine/DMF (2 × 3 min) to deprotect the Fmoc group. The combined deprotection solutions were diluted to 10 mL with 10% piperidine/DMF. An aliquot of this mixture (12.5 µL) was diluted 800-fold with 10% piperidine/DMF and the UV absorbance of the piperidine-fulvene adduct was measured (λ = 301 nm, ε = 7800 M^{-1} cm^{-1}) to quantify the amount of amino acid loaded onto the resin. The theoretical maximum for the reported yields of all isolated peptides are based on the numerical value obtained from the resin loading.

General iterative peptide assembly (Fmoc-SPPS)

Peptides were elongated using iterative Fmoc-solid-phase peptide synthesis (Fmoc-SPPS), according to the following general protocols:

Deprotection: The resin was treated with piperidine/DMF (1:9 v:v, 2 × 3 min) and washed with DMF (5 × 3 mL), DCM (5 × 3 mL) and DMF (5 × 3 mL).

General amino acid coupling: A preactivated solution of Fmoc-protected amino acid (4.0 equiv.), ethyl cyano(hydroxyimino)acetate (Oxyma Pure®) (4.0 equiv.), and *N,N'*-diisopropylcarbodiimide (DIC) (4.0 equiv.) in DMF (final concentration 0.1 M with respect to the resin bound peptide) was added to the resin. After 16 h, the resin was washed with DMF (5 × 3 mL), DCM (5 × 3 mL) and DMF (5 × 3 mL).

Capping: A solution of acetic anhydride/pyridine (1:9 v:v) was added to the resin. After 3 min the resin was washed with DMF (5 × 3 mL), DCM (5 × 3 mL) and DMF (5 × 3 mL).

n**Butyric succinimide ester coupling**

A solution of butyric succinimide ester (4.0 equiv.) and DIEA (4.0 equiv.) in dry DMF (final concentration of 0.1 M with respect to resin) was added to the resin (1.0 equiv.). The resin was shaken for 16 h and the progress of the reaction checked by cleavage of a small portion of resin beads followed by UPLC-MS analysis. The coupling procedure was repeated if necessary, and upon completion, the resin was washed with DMF (5 × 3 mL) and DCM (10 × 3 mL), followed by resin cleavage.

n**Butyric acid coupling (microwave conditions)**

The resin was transferred to a microwave vessel, and then a solution of butyric acid (4.0 equiv.), ethyl cyano(hydroxyimino)acetate (Oxyma Pure®) (4.0 equiv.) and *N,N'*-diisopropyl-carbodiimide (DIC) (4.0 equiv.) in DMF (final concentration 0.1 M) was added. The vessel was capped and irradiated in a mono-mode microwave cavity (200 W, 50 °C, 3 h). Upon completion, the resin was transferred to a disposable solid-phase reaction vessel, washed, and subjected to resin cleavage, as described below.

Cleavage: A mixture of 1,1,1,3,3,3-hexafluoro-2-propanol (HFIP) and DCM (3:7 v:v) was added to the resin. After 2 h, the resin was filtered and washed with DCM (3 × 3 mL).

Work-up: The combined cleavage solution and DCM washes were concentrated under a stream of nitrogen. The residue was subsequently dissolved in water/acetonitrile containing 0.1% TFA, filtered and purified by reverse-phase HPLC.

Compound **2a**

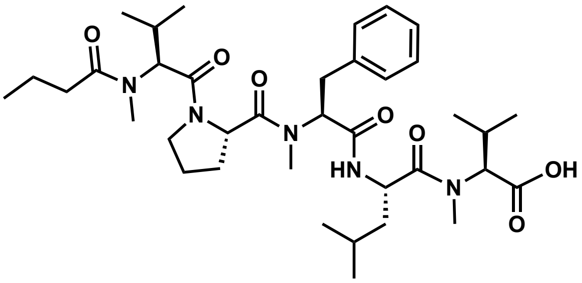

Lipo-carboxylic acid **2a** was prepared on a 146 µmol scale on 2-chlorotrityl chloride resin using standard Fmoc-SPPS. Butyric acid was incorporated according to the general procedures for SPPS under microwave conditions. Following cleavage from the resin and removal of volatiles, the crude peptide was purified by preparative reverse-phase HPLC (40% MeCN to 75% MeCN over 20 min, 10 mL/min) to afford peptide **2a** (18.0 mg, 18% yield based on the original resin loading).

Physical State: white fluffy solid (following lyophilization).
^{1}H NMR (700 MHz, CD_3OD, major rotamer) δ 9.04 (d, *J* = 7.2 Hz, 1H, N*H*), 7.34 – 7.18 (m, 5H), 5.13 (dd, *J* = 11.6, 3.2 Hz, 1H), 4.98 (d, *J* = 10.9 Hz, 1H), 4.83 – 4.80 (m, 1H), 4.76 (d, *J* = 10.3 Hz, 1H), 4.57 – 4.52 (m, 1H), 3.83 – 3.78 (m, 1H), 3.56 – 3.50 (m, 1H), 3.21 (dd, *J* = 14.3, 3.2 Hz, 1H), 3.16 (s, 3H), 3.02 (s, 3H), 3.00 – 2.97 (m, 1H), 2.85 (s, 3H), 2.43 – 2.33 (m, 2H), 2.28 – 2.18 (m, 2H), 1.89 – 1.76 (m, 3H), 1.65 – 1.53 (m, 3H), 1.44 – 1.39 (m, 1H), 1.07 (d, *J* = 6.5 Hz, 3H), 0.98 – 0.94 (m, 9H), 0.92 (d, *J* = 6.7 Hz, 3H), 0.89 (d, *J* = 6.5 Hz, 3H), 0.88 – 0.83 (m, 2H), 0.80 (d, *J* = 6.8 Hz, 3H).
^{13}C NMR (176 MHz, CD_3OD) δ 176.4, 175.9, 175.3, 173.5, 171.8, 170.0, 139.4, 130.6, 129.9, 128.0, 64.2, 63.8, 60.5, 56.9, 50.4, 49.4, 40.1, 36.5, 34.7, 32.2, 31.4, 30.1, 29.8, 28.9, 28.3, 26.2, 26.0, 23.8, 21.0, 20.5, 19.6, 19.5, 19.5, 18.8, 14.2.
HRMS (ESI-TOF): calc'd for $C_{37}H_{58}N_5O_7$ $[M-H]^-$: 684.4331; found: 684.4315.
UPLC trace:

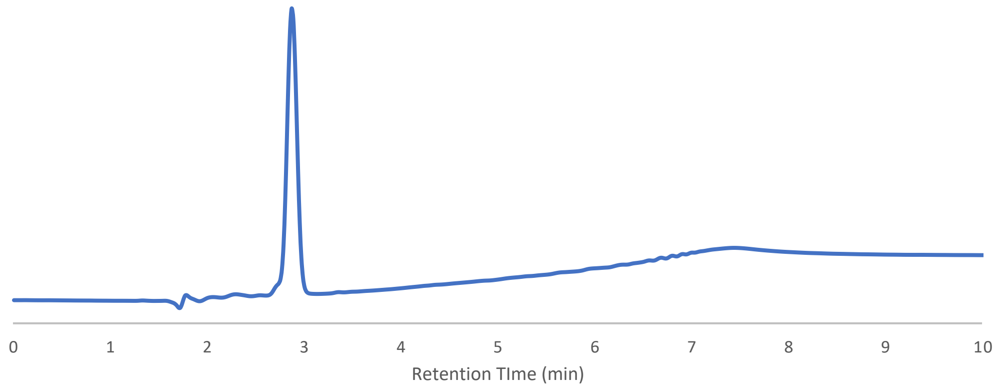

Purified peptide **2c** (Rt = 2.9 min, 70% to 95% B over 5 min, λ = 230 nm).

Compound **2b**

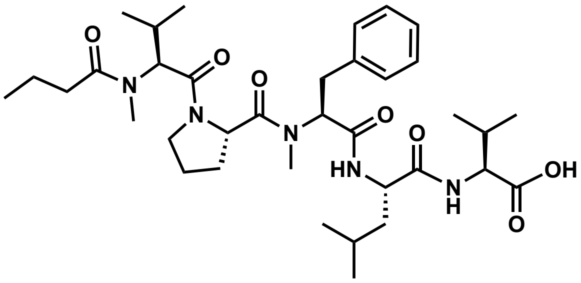

Lipo-carboxylic acid **2b** was prepared on a 162 μmol scale on 2-chlorotrityl chloride resin using standard Fmoc-SPPS. Butyric acid was incorporated according to the general procedure for SPPS under microwave conditions. Following cleavage from the resin and removal of volatiles, the crude peptide was purified by preparative reverse-phase HPLC (60% MeCN to 85% MeCN over 20 min, 10 mL/min) to afford peptide **2b** (30.5 mg, 28% yield based on the original resin loading).

Physical State: white fluffy solid (following lyophilization).
^{1}H NMR (600 MHz, CD_3OD) δ 8.93 (d, *J* = 7.4 Hz, 1H, N*H*), 8.15 (d, *J* = 8.5 Hz, 1H, N*H*), 7.35 – 7.17 (m, 5H), 5.14 (dd, *J* = 11.7, 3.3 Hz, 1H), 4.97 (d, *J* = 10.9 Hz, 1H), 4.55 – 4.52 (m, 1H), 4.51 – 4.46 (m, 1H), 4.35 – 4.31 (m, 1H), 3.82 – 3.77 (m, 1H), 3.56 – 3.50 (m, 1H), 3.22 (dd, *J* = 14.4, 3.1 Hz, 1H), 3.00 (s, 3H), 2.99 – 2.95 (m, 1H), 2.85 (s, 3H), 2.42 – 2.29 (m, 2H), 2.23 – 2.16 (m, 2H), 1.86 – 1.80 (m, 2H), 1.78 – 1.70 (m, 1H), 1.65 – 1.51 (m, 4H), 1.03 – 0.99 (m, 6H), 0.97 – 0.92 (m, 9H), 0.89 (d, *J* = 6.5 Hz, 3H), 0.87 – 0.82 (m, 2H), 0.80 (d, *J* = 6.7 Hz, 3H).
^{13}C NMR (151 MHz, CD_3OD) δ 176.3, 175.6, 175.3, 174.7, 171.8, 170.2, 139.4, 130.6, 129.9, 128.0, 64.3, 60.5, 59.1, 56.9, 53.6, 49.6, 41.2, 36.4, 34.7, 31.8, 31.4, 30.1, 29.8, 28.9, 26.2, 25.8, 23.8, 21.1, 19.7, 19.6, 19.5, 18.8, 18.5, 14.1.
HRMS (ESI-TOF): calc'd for $C_{36}H_{57}N_5O_7Na$ $[M+Na]^+$: 694.4150; found: 694.4142.
UPLC trace:

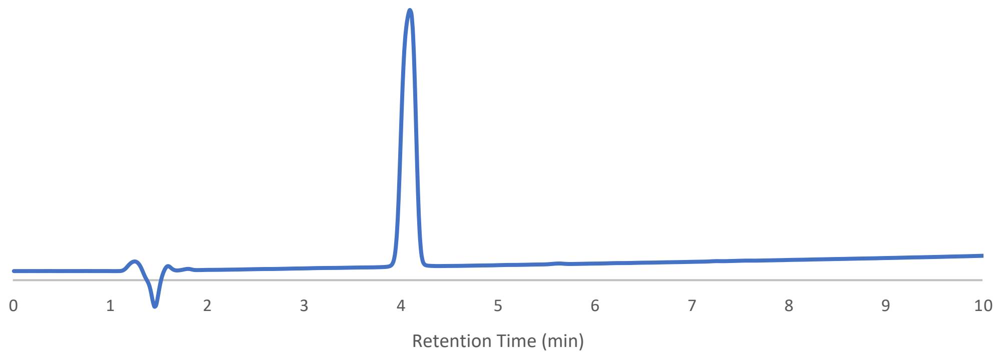

Purified peptide **2b** (Rt = 4.1 min, 50% to 95% B over 10 min, λ = 230 nm).

Compound **2c**

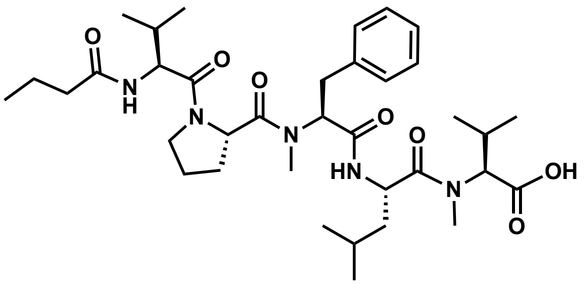

Lipo carboxylic acid **2c** was prepared on a 478 µmol scale on 2-chlorotrityl chloride resin using standard Fmoc-SPPS. NHS-activated butyric acid was incorporated according to the general procedure for SPPS. Following cleavage from the resin and removal of volatiles, the crude peptide was purified by preparative reverse-phase HPLC (40% MeCN to 75% MeCN over 20 min, 10 mL/min) to afford peptide **2c** (57.8 mg, 18% yield based on the original resin loading).

Physical State: white fluffy solid (following lyophilization).
^{1}H NMR (400 MHz, CD_3OD) δ 7.41 – 7.15 (m, 5H), 5.19 (dd, J = 11.7, 3.3 Hz, 1H), 4.91 – 4.79 (m, 2H), 4.52 – 4.45 (m, 1H), 4.37 (d, J = 9.0 Hz, 1H), 3.90 – 3.82 (m, 1H), 3.60 – 3.48 (m, 1H), 3.17 (s, 3H), 3.16 – 3.10 (m, 1H), 3.06 – 3.00 (m, 1H), 2.84 (s, 3H), 2.29 – 2.15 (m, 3H), 2.06 – 1.97 (m, 1H), 1.93 – 1.82 (m, 2H), 1.70 – 1.54 (m, 4H), 1.46 – 1.36 (m, 1H), 1.13 – 1.05 (m, 3H), 1.02 – 0.75 (m, 20H).
^{13}C NMR (101 MHz, CD_3OD) δ 176.0, 175.8, 175.5, 173.4, 171.6, 171.6, 139.2, 130.7, 129.9, 128.0, 63.9, 63.6, 57.5, 56.7, 49.9, 49.7 (assigned through HSQC), 49.1, 40.3, 38.7, 34.8, 32.2, 32.0, 30.0, 29.9, 28.3, 26.2, 26.0, 23.8, 21.2, 20.4, 19.6, 19.5, 19.0, 14.1.
HRMS (ESI-TOF): calc'd for $C_{36}H_{56}N_5O_7$ $[M-H]^-$: 670.4174; found: 670.4162.
UPLC trace:

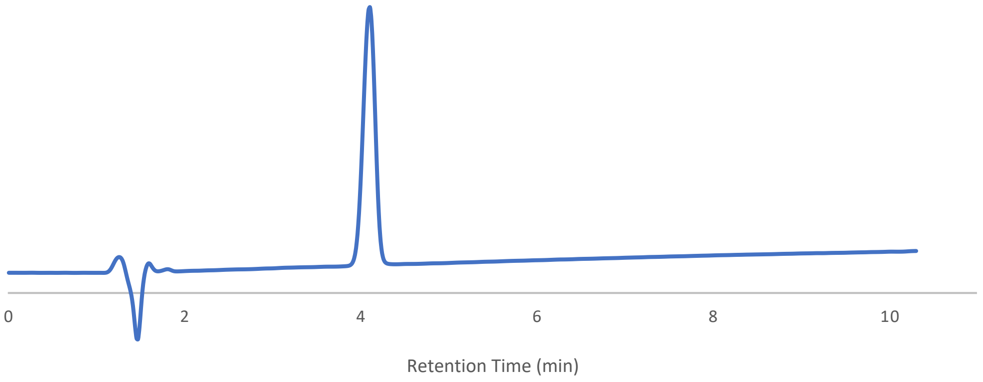

Purified peptide **2c** (Rt = 4.1 min, 50% to 95% B over 10 min, λ = 230 nm).

Screening conditions for decreasing epimerization:

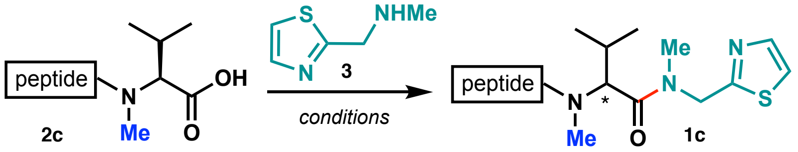

Entry	Coupling Reagents	Equiv. of thiazole 3	Preactivation time	Addition temp.	Reaction temp.	*d.r.*[a] (L-Val/D-Val)
1[b]	5 equiv. Oxyma<br>5 equiv. DIC	5.0	30 min	r.t.	r.t.	48:52
2	10 equiv. Oxyma<br>10 equiv. DIC	10.0	1 min	r.t.	r.t.	72:28
3	10 equiv. Oxyma<br>10 equiv. DIC	10.0	—	r.t.	r.t.	77:23
4	10 equiv. Oxyma<br>10 equiv. DIC	10.0	—	0 °C	r.t.	81:19
5	10 equiv. Oxyma<br>10 equiv. DIC	10.0	—	0 °C	5 °C	85:15
6	10 equiv. Oxyma<br>10 equiv. DIC	5.0	—	0 °C	5 °C	75:25
7	10 equiv. HOBt<br>10 equiv. DIC	10.0	—	0 °C	5 °C	61:39
8	10 equiv. HOBt<br>10 equiv. EDC	10.0	—	0 °C	5 °C	69:31

[a] *d.r.* was determined by integration of UPLC peak area, λ = 210 nm.
[b] Final concentration 0.13 M.

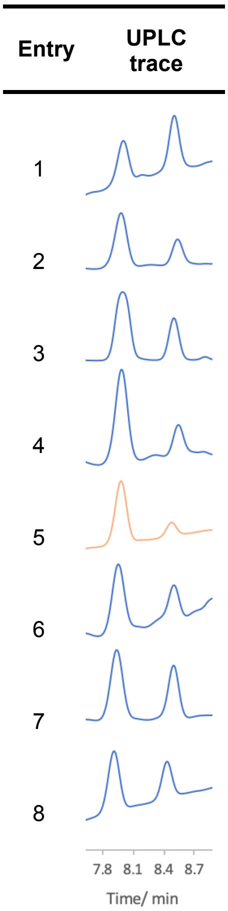

Preparation: A pre-tared 1.5 mL Eppendorf® tube was charged peptide acid **2c** (0.5 mg – 1.0 mg), and re-lyophilized to obtain an accurate mass of peptide starting material. Building block **3** and all other reagents were freshly prepared as stock solutions (2 M in DMF) and sonicated until complete dissolution. For entries 4-8, the stock solutions were cooled to 0 °C in an ice bath before addition. The reactions were conducted at a final concentration of 0.07 M with respect to peptide 2**c** unless otherwise specified.

Optimization of N-methyl-1-(thiazol-2-yl)methanamine coupling: To the 1.5 mL Eppendorf® tube containing peptide **2c** (0.5 mg – 1.0 mg) was added *N*-methyl-1-(thiazol-2-yl) methanamine solution (10.0 equiv., 2 M in DMF) and Oxyma Pure® solution (ethyl (hydroxyimino)cyanoacetate, 10.0 equiv., 2 M in DMF). At the specified addition temperature, DIC solution (*N,N'*-diisopropylcarbodiimide, 10.0 equiv., 2 M in DMF) was added and the reaction was kept at the specified reaction temperature overnight (usually 16 h). Consumption of the starting peptide and the diastereomeric ratio was monitored by UPLC (50% B isocratic, λ = 210 nm; biseokeaniamide C: Rt = 7.9 min; diastereomer: Rt = 8.4 min).

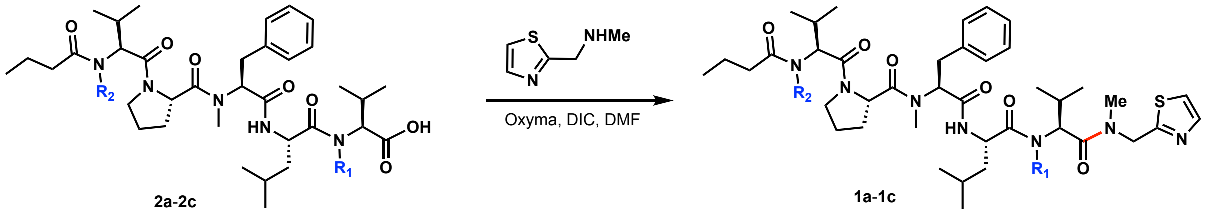

Optimized protocol: In a 1.5 mL Eppendorf® vial, peptide acid **2a**, **2b** or **2c** (1.0 equiv.) was weighed and lyophilized. *N*-methyl-1-(thiazol-2-yl)methanamine (10.0 equiv., 2 M in DMF) and Oxyma Pure® (ethyl (hydroxyimino)cyanoacetate, 10.0 equiv., 2 M in DMF) were added and the mixture was cooled to 0 °C. A solution of *N,N'*-diisopropylcarbodiimide in DMF (2 M, 10.0 equiv.) was added at this temperature and the reaction was kept at 5 °C overnight (usually 16 h, final concentration 0.07 M). Consumption of the starting peptide was monitored by UPLC. Upon completion, the reaction mixture was subjected directly to preparative reverse-phase HPLC purification.

Compound **1a**

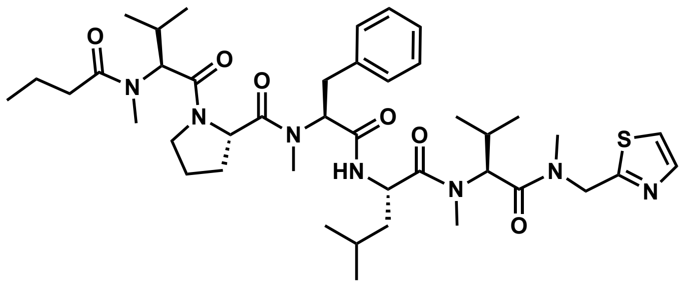

Biseokeaniamide A

Following the optimized procedure on a 5.71 μmol scale of peptide acid **2a**. Purification by preparative reverse-phase HPLC (50% MeCN hold for 7 min, then 50% MeCN to 80% MeCN over 20 min, 0.1% formic acid, 5 mL/min) afforded biseokeaniamide A **1a** in 67% yield (3.04 mg).

Spectral data matches the literature.[3]

Physical State: yellow fluffy solid (following lyophilization).
^{1}H NMR (600 MHz, CD_3OD) δ 9.06 (d, *J* = 7.4 Hz, 1H, N*H*), 7.73 (d, *J* = 3.3 Hz, 1H), 7.58 (d, *J* = 3.3 Hz, 1H), 7.35 – 7.17 (m, 5H), 5.23 – 5.07 (m, 3H), 4.98 (d, *J* = 10.6 Hz, 1H), 4.80 – 4.72 (m, 1H), 4.64 (d, *J* = 15.3 Hz, 1H), 4.60 – 4.51 (m, 1H), 3.82 – 3.75 (m, 1H), 3.56 – 3.48 (m, 1H), 3.22 (dd, *J* = 14.3, 3.1 Hz, 1H), 3.13 – 3.07 (m, 5H), 3.02 – 2.95 (m, 2H), 2.93 (s, 3H), 2.84 (s, 3H), 2.43 – 2.23 (m, 3H), 2.18 – 2.10 (m, 1H), 1.88 – 1.69 (m, 3H), 1.64 – 1.52 (m, 3H), 1.34 – 1.15 (m, 1H), 0.97 – 0.89 (m, 14H), 0.89 – 0.84 (m, 6H), 0.79 (d, *J* = 6.8 Hz, 3H).
^{13}C NMR (151 MHz, CD_3OD) δ 176.3, 175.7, 175.3, 171.8, 171.6, 170.0, 168.0, 143.0, 139.4, 130.6, 129.9, 128.0, 121.7, 64.3, 60.4, 59.8, 56.9, 50.6, 49.6, 49.0, 40.7, 36.4, 36.1, 34.7, 31.2, 30.8, 30.1, 29.8, 28.9, 28.0, 26.2, 26.0, 23.9, 21.0, 19.9, 19.5, 19.5, 18.8, 14.1.
HRMS (ESI-TOF): calc'd for $C_{42}H_{65}N_7O_6SNa$ $[M+Na]^+$: 818.4609; found: 818.4608.

UPLC trace:

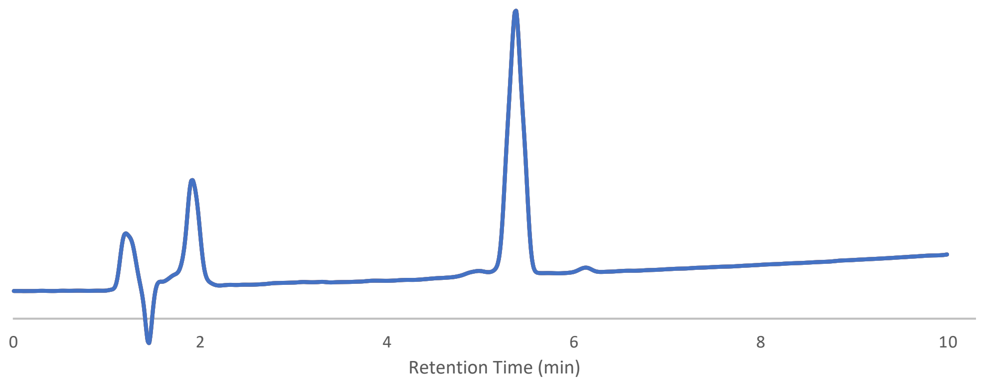

Purified peptide **1a** (Rt = 5.4 min, 50% to 95% B over 10 min, λ = 230 nm).

Compound **1b**

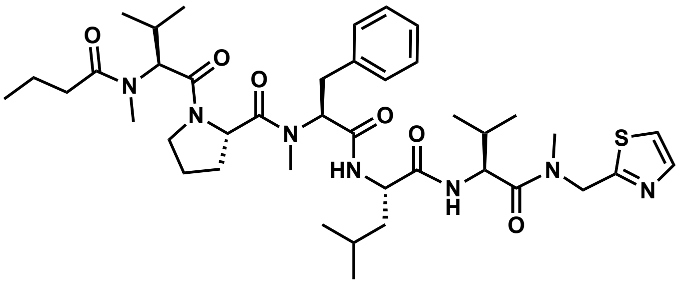

Biseokeaniamide B

Following the optimized procedure on a 15.5 μmol scale of peptide acid **2b**. Purification by preparative reverse-phase HPLC (55% MeCN hold for 5 min, then 55% MeCN to 65% MeCN over 20 min, 5 mL/min) afforded biseokeaniamide B **1b** in 53% yield (6.42 mg).

Spectral data matches the literature.[3]

Physical State: yellow fluffy solid (following lyophilization).
^{1}H NMR (600 MHz, CD_3OD) δ 8.91 (d, *J* = 7.3 Hz, 1H, N*H*), 8.04 (d, *J* = 8.7 Hz, 1H, N*H*), 7.74 (d, *J* = 3.4 Hz, 1H), 7.56 (d, *J* = 3.3 Hz, 1H), 7.34 – 7.17 (m, 5H), 5.16 (dd, *J* = 11.8, 3.4 Hz, 1H), 4.98 (d, *J* = 10.9 Hz, 1H), 4.94 – 4.85 (m, 2H), 4.75 – 4.70 (m, 1H), 4.59 – 4.55 (m, 1H), 4.45 – 4.39 (m, 1H), 3.82 – 3.75 (m, 1H), 3.57 – 3.51 (m, 1H), 3.26 (s, 3H), 3.25 – 3.19 (m, 1H), 3.05 – 2.98 (m, 1H), 2.97 (s, 3H), 2.84 (s, 3H), 2.42 – 2.27 (m, 2H), 2.21 – 2.09 (m, 2H), 1.87 – 1.75 (m, 2H), 1.74 – 1.67 (m, 1H), 1.64 – 1.51 (m, 4H), 0.99 – 0.87 (m, 20H), 0.81 (d, *J* = 6.7 Hz, 3H).
^{13}C NMR (151 MHz, CD_3OD) δ 176.2, 175.3, 175.3, 173.9, 171.9, 170.3, 168.1, 143.0, 139.4, 130.6, 129.9, 128.0, 121.8, 64.4, 60.6, 56.9, 55.6, 53.9, 50.0, 49.6, 41.5, 36.5, 36.4, 34.7, 32.0, 31.4, 30.1, 29.8, 29.0, 26.2, 25.9, 23.8, 21.1, 19.8, 19.6, 19.5, 18.8, 18.6, 14.1.
HRMS (ESI-TOF): calc'd for $C_{41}H_{63}N_7O_6SNa$ $[M+Na]^+$: 804.4453; found: 804.4439.

UPLC trace:

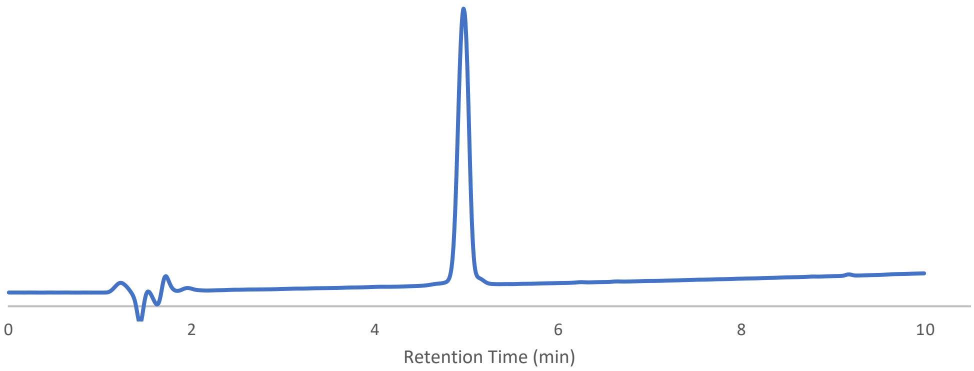

Purified peptide **1b** (Rt = 5.0 min, 50% to 75% B over 10 min, λ = 230 nm).

Compound **1c**

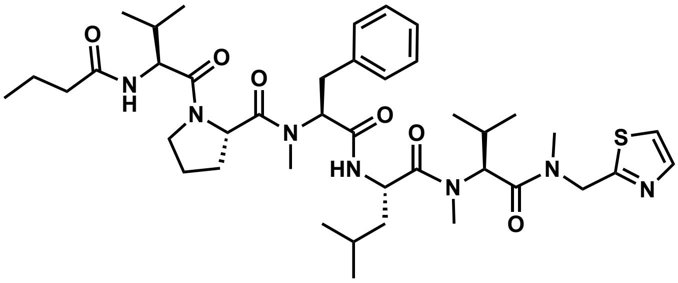

Biseokeaniamide C

Following the optimized procedure on a 5.71 µmol scale of peptide acid **2c**. Purification by preparative reverse-phase HPLC (50% MeCN hold for 5 min, then 50% MeCN to 85% MeCN over 30 min, 5 mL/min) afforded biseokeaniamide C **1c** in 69% yield (3.08 mg).

Spectral data matches the literature.[3]

Physical State: orange fluffy solid (following lyophilization).
^{1}H NMR (600 MHz, CD_3OD) δ 8.92 (d, *J* = 7.4 Hz, 1H, N*H*), 7.73 (dd, *J* = 3.3, 1.1 Hz, 1H), 7.57 (dd, *J* = 3.4, 1.1 Hz, 1H), 7.34 – 7.16 (m, 5H), 5.23 – 5.10 (m, 2H), 5.07 (d, *J* = 15.5 Hz, 1H), 4.88 – 4.79 (m, 1H), 4.72 – 4.67 (m, 1H), 4.52 – 4.42 (m, 1H), 4.36 (d, *J* = 8.7 Hz, 1H), 3.88 – 3.82 (m, 1H), 3.57 – 3.50 (m, 1H), 3.17 – 3.09 (m, 6H), 3.04 – 2.95 (m, 2H), 2.83 (s, 3H), 2.43 – 2.34 (m, 1H), 2.24 – 2.13 (m, 2H), 2.04 – 1.96 (m, 1H), 1.92 – 1.85 (m, 1H), 1.82 – 1.69 (m, 1H), 1.66 – 1.56 (m, 4H), 1.38 – 1.30 (m, 1H), 0.99 – 0.82 (m, 23H).
^{13}C NMR (151 MHz, CD_3OD) δ 175.7, 175.6, 175.4, 171.7, 171.7, 171.5, 168.0, 143.0, 139.2, 130.7, 129.9, 128.0, 121.6, 63.8, 59.9, 57.6, 56.8, 50.2, 49.9, 49.1, 40.9, 38.7, 36.3, 34.9, 32.0, 31.0, 30.0, 29.9, 28.2, 26.2, 26.0, 23.7, 21.4, 20.3, 20.0, 19.6, 18.8, 18.7, 14.1.
HRMS (ESI-TOF): calc'd for $C_{41}H_{63}N_7O_6SNa$ $[M+Na]^+$: 804.4453; found: 804.4462.

UPLC trace:

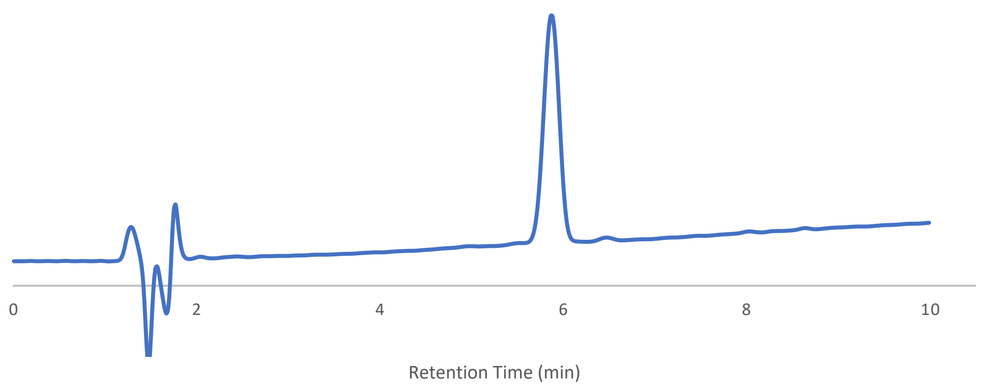

Purified peptide **1c** (Rt = 5.9 min, 50% to 75% B over 10 min, λ = 230 nm).

Comparison of isolated and synthetic natural products (1D NMR)

Comparison of biseokeaniamide A ^{1}H NMR data

Position	Literature[3] (400 MHz)	Synthetic (600 MHz)	Δδ
Thz-*N*-Me-Gly			
1	7.74 (d, *J* = 3.4 Hz, 1H)	7.73 (d, *J* = 3.3 Hz, 1H)	–0.01
2	7.59 (d, *J* = 3.4 Hz, 1H)	7.58 (d, *J* = 3.3 Hz, 1H)	–0.01
3	–	–	–
4a	5.13 (d, *J* = 15.7 Hz, 1H)	5.12 (m, 1H)	–0.01
4b	4.64 (d, *J* = 15.7 Hz, 1H)	4.64 (d, *J* = 15.3 Hz, 1H)	0
5	3.11 (s, 3H)	3.11 (s, 3H)	0
N-Me-Val1			
1	–	–	–
2	5.16 (d, *J* = 11.1 Hz, 1H)	5.16 (d, *J* = 10.7 Hz, 1H)	0
3	2.37 (m, 1H)	2.38 (m, 1H)	+0.01
4	0.96 (m, 3H)	0.95 (m, 3H)	–0.01
5	0.92 (m, 3H)	0.90 (m, 3H)	–0.02
6	3.08 (s, 3H)	3.08 (s, 3H)	0
Leu			
1	–	–	–
2	4.79 (m, 1H)	4.79 (m, 1H)	0
3a	1.79 (m, 1H)	1.78 (m, 1H)	–0.01
3b	1.30 (m, 1H)	1.30 (m, 1H)	0
4	1.74 (m, 1H)	1.73 (m, 1H)	–0.01
5/6	0.94 (m, 6H)	0.92 (m, 6H)	–0.02
*N*H	9.07 (d, *J* = 7.5 Hz, 1H)	9.06 (d, *J* = 7.4 Hz, 1H)	–0.01
N-Me-Phe			
1	–	–	–
2	5.14 (m, 1H)	5.12 (m, 1H)	–0.02
3a	3.22 (m, 1H)	3.22 (m, 1H)	0
3b	2.99 (m, 1H)	2.99 (m, 1H)	0
4	–	–	–
5/9	7.21 (m, 2H)	7.19 (m, 2H)	–0.02
6/8	7.32 (m, 2H)	7.30 (m, 2H)	–0.02
7	7.25 (m, 1H)	7.23 (m, 1H)	–0.02
10	2.84 (s, 3H)	2.84 (s, 3H)	0
Pro			
1	–	–	–
2	4.55 (m, 1H)	4.55 (m, 1H)	0
3	0.83 (m, 2H)	0.86 (m, 2H)	+0.03
4a	1.83 (m, 1H)	1.83 (m, 1H)	0
4b	1.58 (m, 1H)	1.56 (m, 1H)	–0.02
5a	3.79 (m, 1H)	3.79 (m, 1H)	0
5b	3.51 (m, 1H)	3.52 (m, 1H)	+0.01
N-Me-Val2			
1	–	–	–
2	4.98 (d, *J* = 11.0 Hz, 1H)	4.98 (d, *J* = 10.6, 1H)	0

3	2.14 (m, 1H)	2.13 (m, 1H)	–0.01
4	0.86 (m, 3H)	0.87 (m, 3H)	+0.01
5	0.79 (d, J = 6.8 Hz, 3H)	0.79 (d, J = 6.8 Hz, 3H)	0
6	2.93 (s, 3H)	2.93 (s, 3H)	0
BA			
1	–	–	–
2a	2.36 (m, 1H)	2.33 (m, 2H)	–
2b	2.33 (m, 1H)		
3	1.61 (m, 2H)	1.60 (m, 2H)	–0.01
4	0.95 (m, 3H)	0.95 (m, 3H)	0

Comparison of biseokeaniamide A ^{13}C NMR data

**Position**	**Literature[3] (400 MHz)**	**Synthetic (600 MHz)**	**Δδ**
Thz-*N*-Me-Gly			
1	143.0	143.0	0
2	121.7	121.7	0
3	168.0	168.0	0
4	49.8	49.6	–0.2
5	36.1	36.1	0
N-Me-Val1			
1	171.6	171.6	0
2	59.8	59.8	0
3	27.9	28.0	+0.1
4	19.9	19.9	0
5	20.9	21.0	+0.1
6	31.1	31.2	+0.1
Leu			
1	175.7	175.7	0
2	49.9	50.6	+0.7
3	40.7	40.7	0
4	26.0	26.0	0
5/6	23.9	23.9	0
N-Me-Phe			
1	171.8	171.8	0
2	64.3	64.3	0
3	34.6	34.7	+0.1
4	139.3	139.4	+0.1
5/9	130.6	130.6	0
6/8	129.9	129.9	0
7	128.0	128.0	0
10	30.1	30.1	0
Pro			
1	175.2	175.3	+0.1
2	56.9	56.9	0

3	29.7	29.8	+0.1
4	26.2	26.2	0
5	48.4	49.0	+0.6
N-Me-Val2			
1	170.0	170.0	0
2	60.4	60.4	0
3	28.9	28.9	0
4	19.5	19.5	0
5	18.8	18.8	0
6	30.8	30.8	0
BA			
1	176.2	176.3	+0.1
2	36.4	36.4	0
3	19.5	19.5	0
4	14.2	14.1	–0.1

Comparison of biseokeaniamide B ^{1}H NMR data

Position	Literature[3] (400 MHz)	Synthetic (600 MHz)	Δδ
Thz-*N*-Me-Gly			
1	7.73 (d, *J* = 3.5 Hz, 1H)	7.74 (d, *J* = 3.4 Hz, 1H)	+0.01
2	7.57 (d, *J* = 3.5 Hz, 1H)	7.56 (d, *J* = 3.3 Hz, 1H)	–0.01
3	–	–	–
4a	4.92 (m, 1H)	4.91 (m, 1H)	–0.01
4b	4.89 (m, 1H)	4.88 (m, 1H)	–0.01
5	3.26 (s, 3H)	3.26 (s, 3H)	0
Val			
1	–	–	–
2	4.72 (d, *J* = 8.0 Hz, 1H)	4.73 (m, 1H)	+0.01
3	2.13 (m, 1H)	2.13 (m, 1H)	0
4	0.97 (m, 3H)	0.98 (m, 3H)	+0.01
5	0.96 (m, 3H)	0.97 (m, 3H)	+0.01
Leu			
1	–	–	–
2	4.42 (m, 1H)	4.42 (m, 1H)	0
3a	1.77 (m, 1H)	1.79 (m, 1H)	+0.02
3b	1.53 (m, 1H)	1.54 (m, 1H)	+0.01
4	1.71 (m, 1H)	1.72 (m, 1H)	+0.01
5	0.94 (m, 3H)	0.95 (m, 3H)	+0.01
6	0.92 (m, 3H)	0.92 (m, 3H)	0
N-Me-Phe			
1	–	–	–
2	5.17 (m, 1H)	5.17 (m, 1H)	0
3a	3.22 (m, 1H)	3.22 (m, 1H)	0
3b	3.00 (m, 1H)	2.99 (m, 1H)	–0.01
4	–	–	–
5/9	7.20 (m, 2H)	7.21 (m, 2H)	+0.01
6/8	7.32 (m, 2H)	7.32 (m, 2H)	0
7	7.25 (m, 1H)	7.25 (m, 1H)	0
10	2.84 (s, 3H)	2.85 (s, 3H)	+0.01
Pro			
1	–	–	–
2	4.57 (m, 1H)	4.58 (m, 1H)	+0.01
3	0.84 (m, 2H)	0.85 (m, 2H)	+0.01
4a	1.81 (m, 1H)	1.85 (m, 1H)	+0.04
4b	1.55 (m, 1H)	1.56 (m, 1H)	+0.01
5a	3.79 (m, 1H)	3.79 (m, 1H)	0
5b	3.54 (m, 1H)	3.54 (m, 1H)	0
N-Me-Val			
1	–	–	–
2	4.98 (d, *J* = 11.0 Hz, 1H)	4.98 (d, *J* = 10.9, 1H)	0
3	2.16 (m, 1H)	2.18 (m, 1H)	+0.02
4	0.89 (m, 3H)	0.90 (m, 3H)	+0.01
5	0.81 (d, *J* = 6.9 Hz, 3H)	0.81 (d, *J* = 6.9 Hz, 3H)	0

6	2.96 (s, 3H)	2.97 (s, 3H)	+0.01
BA			
1	–	–	–
2a	2.37 (m, 1H)	2.38 (m, 1H)	+0.01
2b	2.33 (m, 1H)	2.33 (m, 1H)	0
3	1.61 (m, 2H)	1.62 (m, 2H)	+0.01
4	0.95 (m, 3H)	0.95 (m, 3H)	0

Comparison of Biseokeaniamide B ^{13}C NMR Data

**Position**	**Literature[3] (400 MHz)**	**Synthetic (600 MHz)**	**Δδ**
Thz-*N*-Me-Gly			
1	143.0	143.0	0
2	121.8	121.8	+0.1
3	168.1	168.1	0
4	50.0	50.0	0
5	36.5	36.5	0
Val			
1	173.9	173.9	0
2	55.7	55.6	–0.1
3	31.9	32.0	+0.1
4	19.8	19.8	0
5	18.6	18.6	0
Leu			
1	175.3	175.3	0
2	53.9	53.9	0
3	41.5	41.5	0
4	25.9	25.9	0
5	23.8	23.8	0
6	21.0	21.1	+0.1
N-Me-Phe			
1	172.0	171.9	–0.1
2	64.4	64.4	0
3	34.6	34.7	+0.1
4	139.4	139.4	0
5/9	130.6	130.6	0
6/8	129.9	129.9	0
7	128.0	128.0	0
10	30.1	30.1	0
Pro			
1	175.4	175.3	–0.1
2	56.9	56.9	0
3	29.8	29.8	0
4	26.2	26.2	0
5	49.6	49.6	0

N-Me-Val			
1	170.3	170.3	0
2	60.5	60.6	+0.1
3	29.0	29.0	0
4	19.6	19.6	0
5	18.9	18.8	–0.1
6	31.4	31.4	0
BA			
1	176.2	176.2	0
2	36.4	36.4	0
3	19.5	19.5	0
4	14.2	14.1	–0.1

Comparison of biseokeaniamide C ^{1}H NMR data

Position	Literature[3] (400 MHz)	Synthetic (600 MHz)	Δδ
Thz-*N*-Me-Gly			
1	7.74 (d, *J* = 3.4 Hz, 1H)	7.73 (dd, *J* = 3.3, 1.1 Hz, 1H)	–0.01
2	7.59 (d, *J* = 3.4 Hz, 1H)	7.57 (d, *J* = 3.4, 1.1 Hz, 1H)	–0.02
3	–	–	–
4a	5.08 (d, *J* = 15.7 Hz, 1H)	5.07 (d, *J* = 15.5 Hz, 1H)	–0.01
4b	4.70 (d, *J* = 15.7 Hz, 1H)	4.71 (m, 1H)	+0.01
5	3.15 (s, 3H)	3.15 (s, 3H)	0
N-Me-Val			
1	–	–	–
2	5.19 (m, 1H)	5.19 (m, 1H)	0
3	2.37 (m, 1H)	2.38 (m, 1H)	+0.01
4	0.94 (m, 3H)	0.95 (m, 3H)	+0.01
5	0.90 (m, 3H)	0.89 (m, 3H)	–0.01
6	3.12 (s, 3H)	3.13 (s, 3H)	+0.01
Leu			
1	–	–	–
2	4.79 (m, 1H)	4.83 (m, 1H)	+0.04
3a	1.80 (m, 1H)	1.78 (m, 1H)	–0.02
3b	1.32 (m, 1H)	1.34 (m, 1H)	+0.02
4	1.66 (m, 1H)	1.64 (m, 1H)	–0.02
5	0.92 (m, 3H)	0.91 (m, 3H)	–0.01
6	0.90 (m, 3H)	0.91 (m, 3H)	+0.01
*N*H	8.96 (br, 1H)	8.92 (d, *J* = 7.4 Hz, 1H)	–0.04
N-Me-Phe			
1	–	–	–
2	5.17 (m, 1H)	5.14 (m, 1H)	–0.03
3a	3.13 (m, 1H)	3.13 (m, 1H)	0
3b	2.97 (m, 1H)	3.00 (m, 1H)	+0.03
4	–	–	–
5/9	7.21 (m, 2H)	7.20 (m, 2H)	–0.01
6/8	7.31 (m, 2H)	7.30 (m, 2H)	–0.01
7	7.25 (m, 1H)	7.23 (m, 1H)	–0.02
10	2.83 (s, 3H)	2.83 (s, 3H)	0
Pro			
1	–	–	–
2	4.49 (m, 1H)	4.49 (m, 1H)	0
3	0.87 (m, 2H)	0.88 (m, 2H)	+0.01
4a	1.85 (m, 1H)	1.89 (m, 1H)	+0.04
4b	1.58 (m, 1H)	1.61 (m, 1H)	+0.03
5a	3.86 (m, 1H)	3.85 (m, 1H)	–0.01
5b	3.53 (m, 1H)	3.54 (m, 1H)	+0.01
Val			
1	–	–	–
2	4.33 (d, *J* = 9.2 Hz, 1H)	4.36 (d, *J* = 8.7, 1H)	+0.03
3	1.99 (m, 1H)	2.00 (m, 1H)	+0.01

4	0.90 (m, 3H)	0.89 (m, 3H)	–0.01
6	0.87 (m, 3H)	0.88 (s, 3H)	+0.01
BA			
1	–	–	–
2a	2.19 (m, 1H)	2.19 (m, 2H)	–
2b	2.17 (m, 1H)		
3	1.60 (m, 2H)	1.61 (m, 2H)	+0.01
4	0.92 (m, 3H)	0.92 (m, 3H)	0

Comparison of biseokeaniamide C ^{13}C NMR data

**Position**	**Literature[3] (400 MHz)**	**Synthetic (600 MHz)**	**Δδ**
Thz-*N*-Me-Gly			
1	143.0	143.0	0
2	121.7	121.6	–0.1
3	168.0	168.0	0
4	49.8	49.9	+0.1
5	36.2	36.3	+0.1
N-Me-Val			
1	171.6	171.5	–0.1
2	59.8	59.9	+0.1
3	28.2	28.2	0
4	20.0	20.0	0
5	18.9	18.8	–0.1
6	31.0	31.0	0
Leu			
1	175.6	175.6	0
2	50.2	50.2	0
3	40.7	40.9	+0.2
4	26.0	26.0	0
5/6	23.8	23.7	–0.1
N-Me-Phe			
1	171.8	171.7	–0.1
2	63.8	63.8	0
3	34.9	34.9	0
4	139.2	139.2	0
5/9	130.7	130.7	0
6/8	130.0	129.9	–0.1
7	128.0	128.0	0
10	30.0	30.0	0
Pro			
1	175.4	175.4	0
2	56.8	56.8	0
3	29.9	29.9	0
4	26.2	26.2	0

5	49.2	49.1	–0.1
Val			
1	171.6	171.7	+0.1
2	57.7	57.6	–0.1
3	32.0	32.0	0
4	18.8	18.8	0
5	19.5	19.6	+0.1
BA			
1	175.8	175.7	–0.1
2	38.6	38.7	+0.1
3	20.4	20.3	–0.1
4	14.1	14.1	0

Experimental procedures: sarcosine model

General procedure A: formation of *N*,*O*-acetal

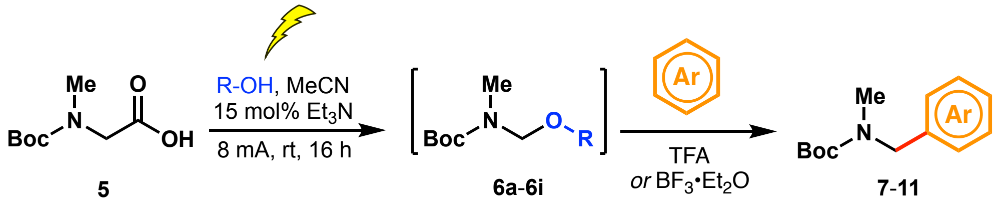

The undivided electrochemical cell was charged with Boc-protected sarcosine **5** (189 mg, 1.0 mmol, 1.0 equiv.) followed by the addition of alcohol or carboxylic acid (3.0 mmol, 3.0 equiv.). Acetonitrile (3 mL) was added to dissolve the solid, along with triethylamine (21 µL, 0.15 mmol, 15 mol%) to generate the electrolyte *in situ*. Carbon electrodes (W7 × D1.5 × H55 mm) were attached and the vial was affixed to the Electrasyn. The reaction was electrolyzed under constant current (8 mA, IKA® Electrasyn 2.0 utilized as the potentiostat) until complete consumption of starting material (monitored by TLC; usually 16 h) was observed. The reaction mixture was transferred to a 20 mL vial and concentrated under reduced pressure to afford the desired *N*,*O*-acetal as a yellow oil or solid. The crude product was used without further purification in the following transformations. *Note 1: In some cases (compounds **6e**, **6g**, **6h**, **6i**), carboxylic acids with high boiling points were employed. Since excess reagents were unable to be removed under reduced pressure, the crude mixture was instead washed with sat. aq. $NaHCO_3$ (3 × 5 mL) and extracted with EtOAc. Concentration of the organic layer afforded the target N,O-acetals. Note 2: N,O-acetals are generally stable in the absence of acid and should be stored at –20 °C if not used immediately.*

Fridel-Crafts type reaction: The *N*,*O*-acetal (1.0 equiv., 0.1 mmol) was dissolved in THF or MeCN (0.5 mL). The specified electron-rich aromatic compound (0.5 mmol, 5.0 equiv.) was added, followed by trifluoroacetic acid (TFA) (1.0 – 3.0 equiv.) or BF_3•Et_2O (2.0 equiv.). The reaction mixture was left stirring at room temperature overnight (typically 12 – 16 h). When complete consumption of *N*,*O*-acetal was observed, the reaction mixture was concentrated under reduced pressure and subjected to purification *via* flash column chromatography.

Probing the reactivity of *N*,*O*-acetals derived from Boc-Sar-OH:

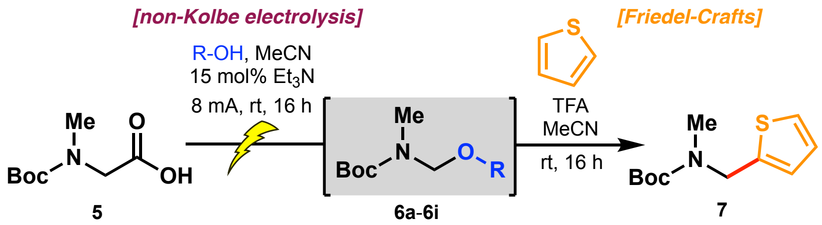

Entry	R =	*N*,*O*-acetal	Yield of 7[a]	Entry	R =	*N*,*O*-acetal	Yield of 7[a]
1	Me[b]	**6a**	n.d. (46%[c])	6	CHO	**6f**	47%
2	CF_3CH_2[d]	**6b**	53%	7	$ClCH_2CO$	**6g**	73%
3	$(CF_3)_2CH$[d]	**6c**	69%	8	Cl_2CHCO	**6h**	66%
4	Ac[d]	**6d**	65%	9	Cl_3CCO	**6i**	11%
5	Bz	**6e**	71%				

[a]Yield was determined by ^1H NMR using dibromomethane as an internal standard; 0.1 mmol scale; n.d. = not determined; [b]neat methanol was used as solvent; [c]isolated yield using microwave irradiation; [d]0.05 mmol scale.

The *N*,*O*-acetal (1.0 equiv., 0.1 mmol) was dissolved in MeCN/thiophene (0.6 mL, 1:5 v:v, 0.17 M), and treated with trifluoroacetic acid (TFA) (8 µL, 0.2 mmol, 2.0 equiv.). The reaction mixture was left stirring at room temperature overnight (typically 12 – 16 h). When complete consumption of *N*,*O*-acetal was observed, the reaction mixture was concentrated under reduced pressure. CH_2Br_2 (10 – 20 mg) was added and the mass was recorded. $CDCl_3$ (1 –2 mL) was added by pipette to the crude reaction mixture, which was subsequently analyzed by ^{1}H NMR spectroscopy.

Microwave optimization: The crude *N*,*O*-acetal **6a** (1.0 equiv., 0.5 mmol) was transferred to a microwave vessel and the volatiles were removed with a gentle stream of N_2. The vessel was backfilled with argon. Dry DCM/thiophene (1:9 v:v, 1 mL) was added to dissolve the crude *N*,*O*-acetal, before the addition of TFA (75 µL, 2.0 equiv.). The vessel was quickly capped and irradiated in a monomode microwave cavity (200 W, 50 °C, 16 h). Upon completion, the volatiles were removed under a stream of N_2 and the crude product was purified by column chromatography (silica gel, 10:1 petroleum spirits/Et_2O) afforded the title compound in 46% yield (52.1 mg).

Compound **7**

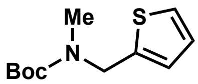

***tert*-Butyl methyl(thiophen-2-ylmethyl)carbamate**

Following general procedure **A** on a 1.0 mmol scale, Boc-Sar-OH was electrolyzed with 1,1,1,3,3,3-hexafluoro-2-propanol (HFIP) to afford the crude *N*,*O*-acetal **6c** as a yellow oil. The following transformation was conducted on a 0.05 mmol scale of *N*,*O*-acetal with TFA (2.0 equiv.) and MeCN/thiophene (0.6 mL, 1:5 v:v, 0.17 M). Purification by column chromatography (silica gel, 10:1 petroleum spirits/Et_2O) afforded the title compound in 65% yield (7.4 mg).

Physical State: colorless oil
TLC: R_f = 0.63 (6:1 petroleum spirits/EtOAc)
^{1}H NMR (400 MHz, $CDCl_3$) δ 7.22 (d, *J* = 5.0 Hz, 1H), 6.97 – 6.89 (m, 2H), 4.54 (s, 2H), 2.85 (s, 3H), 1.50 (s, 9H).
^{13}C NMR (151 MHz, $CDCl_3$) δ 155.4, 140.9, 126.6, 126.1, 125.2, 80.2, 47.7, 33.8, 28.6.
HRMS (ESI-TOF): calc'd for $C_{11}H_{17}NO_2SNa$ $[M+Na]^+$: 250.0872; found: 250.0878.

Spectral data matches the literature.[4]

Compound **8**

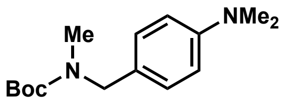

***tert*-Butyl (4-(dimethylamino)benzyl)(methyl)carbamate**

Following general procedure **A** on a 1.0 mmol scale, Boc-Sar-OH was electrolyzed with acetic acid (AcOH) to afford the crude *N*,*O*-acetal **6d** as a yellow oil. *N*,*O*-acetal **6d** (0.5 mmol) was

dissolved in THF (2 mL) and cooled to 0 °C. *N*,*N*-dimethylaniline (0.32 mL, 2.5 mmol, 5.0 equiv.) was added, followed by the addition of $BF_3{\bullet}Et_2O$ (0.12 mL, 1.0 mmol, 2.0 equiv.). The reaction mixture was stirred and slowly warmed to room temperature overnight (typically 12 – 16 h). Purification by column chromatography (silica gel, 100% to 5:1 petroleum spirits/EtOAc) afforded the title compound in 51% yield (67.6 mg).

Physical State: colorless oil
TLC: R_f = 0.67 (3:1 petroleum spirits/EtOAc)
^{1}H NMR (400 MHz, $CDCl_3$) δ 7.12 (d, *J* = 7.9 Hz, 2H), 6.70 (d, *J* = 7.8 Hz, 2H), 4.32 (s, 2H), 2.94 (s, 6H), 2.78 (s, 3H), 1.49 (s, 9H).
^{13}C NMR (101 MHz, $CDCl_3$) δ 156.0, 150.1, 129.1, 128.7, 126.0, 112.7, 79.5, 52.1, 51.4, 40.8, 33.6, 28.7.
Note: Rotamers of the NBoc group lead to a complex ^{13}C NMR spectrum. All observed signals are reported here.
LRMS (ESI): calc'd for $C_{15}H_{25}N_2O_2$ $[M+H]^+$: 265.2; found: 265.2.

Spectral data matches the literature.[4]

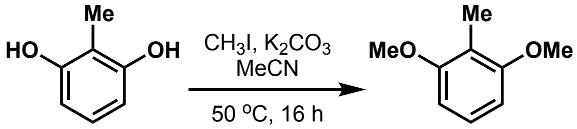

2,6-Dimethoxytoluene

2,6-Dimethoxytoluene was synthesized according to a literature procedure.[5] To an oven-dried two-neck flask containing 2,6-dihydroxytoluene (1.24 g, 10 mmol, 1.0 equiv.) in 50 mL acetonitrile was added potassium carbonate (6.9 g, 5.0 mmol, 5.0 equiv.). A condenser tube was attached and the suspension was cooled to 0 °C. Iodomethane (1.87 mL, 30 mmol, 3.0 equiv.) was then added to the stirring suspension. The resultant suspension was heated to 50 °C and stirred for 16 h. When full consumption of 2,6-dihydroxytoluene was observed, the reaction mixture was filtered through a pad of Celite® and all volatiles were removed under reduced pressure. The crude product was extracted with water and EtOAc. The combined organic layers were dried over $MgSO_4$ and concentrated under reduced pressure. Purification by flash column chromatography (silica gel, 100% to 20:1 petroleum spirits/EtOAc) afforded the title compound in 82% yield (2.07 g).

Physical State: white solid
TLC: R_f = 0.73 (5:1 petroleum sprits/EtOAc)
^{1}H NMR (400 MHz, $CDCl_3$) δ 7.12 (t, *J* = 8.3 Hz, 1H), 6.55 (d, *J* = 8.3 Hz, 2H), 3.83 (s, 6H), 2.11 (s, 3H).

Spectral data matches the literature.[6]

Compound **9**

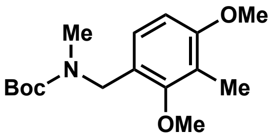

***tert*-Butyl (2,4-dimethoxy-3-methylbenzyl)(methyl)carbamate**

Following general procedure **A** on a 1.0 mmol scale, Boc-Sar-OH was electrolyzed with AcOH to afford the crude *N*,*O*-acetal **6d** as a yellow oil. The following transformation was conducted on a 0.3 mmol scale of *N*,*O*-acetal **6d** with TFA (2.0 equiv.) and 2,6-dimethoxytoluene (152.0 mg, 1.5 mmol, 5.0 equiv.). Purification by flash column chromatography (silica gel, 100% to 4:1 petroleum spirits/Et_2O) afforded the title compound in 54% yield (47.4 mg).

Physical State: colorless oil
TLC: R_f = 0.46 (5:1 petroleum spirits/EtOAc)
^{1}H NMR (400 MHz, $CDCl_3$) δ 6.98 (d, *J* = 8.5 Hz, 1H), 6.62 (d, *J* = 8.5 Hz, 1H), 4.45 (s, 2H), 3.82 (s, 3H), 3.69 (s, 3H), 2.81 (s, 3H), 2.15 (s, 3H), 1.47 (s, 9H).
^{13}C NMR (176 MHz, $CDCl_3$) δ 158.1, 126.3, 125.6, 122.9, 119.7, 106.3, 106.2, 79.6, 60.8, 55.8, 47.2, 34.0, 28.6, 9.2.
HRMS (ESI-TOF): calc'd for $C_{16}H_{25}NO_4Na$ $[M+Na]^+$: 318.1676; found: 318.1673.
IR (neat): 2974, 2937, 2836, 1691, 1390, 1365, 1267, 1139, 1106, 877 cm^{-1}.

Compound **10**

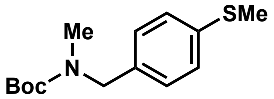

***tert*-Butyl methyl(4-(methylthio)benzyl)carbamate**

Following general procedure **A** on a 1.0 mmol scale, Boc-Sar-OH was electrolyzed with acetic acid (AcOH) to afford the crude *N*,*O*-acetal **6d** as a yellow oil. The following transformation was conducted on a 0.1 mmol scale of *N*,*O*-acetal with TFA (5.0 equiv.) and a mixture of thioanisole: MeCN (4:1 v:v, 0.5 mL). Purification by flash column chromatography (silica gel, 100% to 5:1 petroleum spirits/EtOAc) afforded the title compound in 28% yield (7.5 mg). *Note: Product was slowly oxidized in air to the corresponding sulfoxide.*

Physical State: colorless oil
TLC: R_f = 0.56 (5:1 petroleum spirits/EtOAc)
^{1}H NMR (400 MHz, $CDCl_3$) δ 7.23 (d, *J* = 7.9 Hz, 2H), 7.18 – 7.11 (m, 2H), 4.37 (s, 2H), 2.81 (s, 3H), 2.48 (s, 3H), 1.48 (s, 9H).
^{13}C NMR (151 MHz, $CDCl_3$) δ 156.2, 137.3, 135.2, 128.4, 129.0, 127.0, 79.8, 52.3, 51.6, 34.0, 28.6, 16.1.
Note: Rotamers of the NBoc group lead to a complex ^{13}C NMR spectrum. All observed signals are reported here.
IR (neat): 2974, 2924, 1690, 1390, 1365, 1139, 878 cm^{-1}.
HRMS (ESI-TOF): calc'd for $C_{14}H_{21}NO_2SNa$ $[M+Na]^+$: 290.1185; found: 290.1182.

Compound **11**

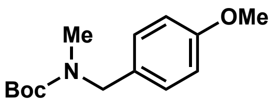

***tert*-Butyl (4-methoxybenzyl)(methyl)carbamate**

Following general procedure **A** on a 1.0 mmol scale, Boc-Sar-OH was electrolyzed with 2,2,2-trifluoroethanol (TFE) to afford the crude *N*,*O*-acetal **6b** as a colorless oil. The following transformation was conducted on a 0.1 mmol scale of *N*,*O*-acetal **6b** with TFA (2.0 equiv.) and anisole (1 mL). Purification by flash column chromatography (silica gel, 100% to 5:1 petroleum spirits/Et_2O) afforded the title compound in 45% yield (11.3 mg, 5:1 *p*-/*o*-isomers). *Note: Characterization data, below, is provided for the p-isomer.*

Physical State: colorless oil
TLC: R_f = 0.50 (5:1 petroleum spirits/EtOAc)
^{1}H NMR (400 MHz, $CDCl_3$) δ 7.16 (d, *J* = 8.0 Hz, 2H), 6.86 (d, *J* = 7.9 Hz, 2H), 4.35 (s, 2H), 3.80 (s, 3H), 2.78 (s, 3H), 1.48 (s, 9H).
^{13}C NMR (151 MHz, $CDCl_3$) δ 159.0, 130.3, 128.9, 128.9 (br, two overlapping signals), 114.0, 79.7, 55.4, 52.0 (br), 33.8, 28.6.
LRMS (ESI): calc'd for $C_{13}H_{21}NO_3Na$ $[M+Na]^+$: 274.3; found: 274.2.

Spectral data matches the literature.[4]

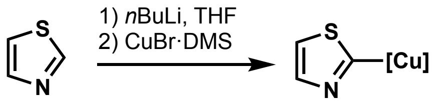

Thiazol-2-ylcopper solution

To a solution of thiazole (142 µL, 2.0 mmol, 1.0 equiv.) in dry THF (2 mL) at –78 °C was added *n*-butyllithium (1.59 mL, 2.0 mmol, 1.26 M in hexane), and the reaction mixture was stirred at –78 °C for 30 min. The lithium reagent was added to a stirring suspension of copper(I) bromide-dimethyl sulfide complex (410 mg, 2.0 mmol, 1.0 equiv.) at –78 °C in THF (2 mL), and the resultant black solution was stirred at –78 °C for 1 h.

Compound **12**

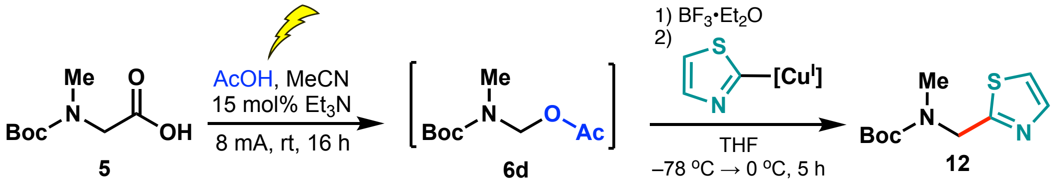

***tert*-Butyl methyl(thiazol-2-ylmethyl)carbamate**

Following general procedure **A** on a 1.0 mmol scale, Boc-Sar-OH was electrolyzed with acetic acid (AcOH) to afford the crude *N*,*O*-acetal **6d** as a yellow oil. To an oven-dried reaction tube containing *N*,*O*-acetal **6d** (101.5 mg, 1.0 equiv., 0.5 mmol) was added dry THF (0.5 mL). The mixture was then cooled to –78 °C and treated with BF_3•Et_2O (630 µL, 5.0 mmol, 10 equiv.). The mixture was stirred at this temperature for 30 min. A solution of organocuprate (2.5 mmol, 5.0 equiv.) was then added, and the mixture was allowed to slowly warm to 0 °C and stirred for an additional 5 h. The black suspension was quenched with a 1:1 mixture of aqueous ammonia and saturated ammonium chloride. The aqueous phase was extracted with Et_2O (3 × 5 mL), and the organic extracts were combined, washed with brine, dried over anhydrous Na_2SO_4, and then filtered. The solvent was removed under reduced pressure, and the dark oil

was purified by flash column chromatography (silica gel, 3:1 petroleum spirits/EtOAc) to afford the title compound in 28% yield (31.9 mg).

Physical State: light yellow oil.
TLC: R_f= 0.13 (5:1 petroleum spirits/EtOAc).
^{1}H NMR (400 MHz, $CDCl_3$) δ 7.70 (d, *J* = 3.1 Hz, 1H), 7.29 (d, *J* = 3.2 Hz, 1H), 4.70 (s, 2H), 2.95 (s, 3H), 1.48 (s, 9H).
^{13}C NMR (101 MHz, $CDCl_3$) δ 168.4, 155.9, 155.1, 142.4, 119.6, 80.7, 50.8, 50.1, 34.8, 28.5.
Note: Rotamers of the NBoc group lead to a complex ^{13}C NMR spectrum. All observed signals are reported here.
HRMS (ESI-TOF): calc'd for $C_{10}H_{16}N_2O_2SNa$ $[M+Na]^+$: 251.0825; found: 251.0816.
IR (neat): 3083, 2976, 2932, 1692, 1389, 1366, 1245, 1150, 873, 771 cm^{-1}

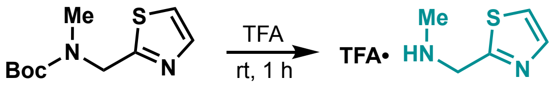

***N*-methyl-1-(thiazol-2-yl)methanamine TFA salt**

In a 10 mL round-bottom flask containing Boc-protected amine **12** (20.0 mg, 0.088 mmol, 1.0 equiv.) was added trifluoroacetic acid (0.5 mL). The reaction mixture was stirred at room temperature for an additional 1 h before all the volatiles were removed under a gentle stream of N_2. The title compound was characterized directly without further purification.

Physical State: yellow oil.
^{1}H NMR (600 MHz, $CDCl_3$): δ 7.84 (d, *J* = 3.3 Hz, 1H), 7.47 (d, *J* = 3.2 Hz, 1H), 4.56 (s, 2H), 2.83 (s, 3H).
^{13}C NMR (151 MHz, $CDCl_3$): δ 158.6, 143.4, 122.2, 48.3, 33.0.
^{19}F NMR (376 MHz, $CDCl_3$): δ –76.65.
LRMS (ESI): calc'd for $C_5H_9N_2S$ $[M-TFA+H]^+$: 129.0; found: 129.0.

Compound **13**

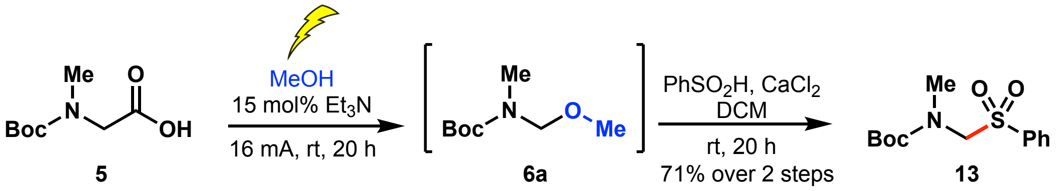

***tert*-Butyl methyl((phenylsulfonyl)methyl)carbamate**

The undivided electrochemical cell was charged with Boc-protected sarcosine **5** (945 mg, 5.0 mmol, 1.0 equiv.). The solid was dissolved in methanol (10 mL), and treated with triethylamine (105 µL, 0.75 mmol, 15 mol%) to enable generation of the electrolyte *in situ*. Carbon electrodes (W7 × D1.5 × H55 mm) were attached. The reaction was electrolyzed under constant current (16 mA, IKA® Electrasyn 2.0 utilized as the potentiostat) until complete consumption of starting material (monitored by TLC; usually 16 h) was observed. The reaction mixture was transferred to a round-bottom flask and concentrated under reduced pressure to afford the desired *N*,*O*-acetal **6a** as a light yellow oil. (*Note: This intermediate is volatile.*) To the crude product was added freshly prepared $PhSO_2H$ (by acidification of sodium

benzenesulfinate, 2.13 g, 15.0 mmol, 3.0 equiv.) and $CaCl_2$ (1.67 mg, 15.0 mmol, 3.0 equiv.). The flask was sealed and backfilled again with argon before the addition of dry DCM (10 mL). The suspension was stirred at room temperature for 20 h before filtering through a short pad of Celite®. The crude residue was purified by column chromatography (silica gel, 3:1 petroleum spirits/EtOAc) to afford the desired product **13** in 71% yield (976.2 mg).

Physical State: white solid.
m.p.: 121 – 122 °C.
TLC: R_f = 0.30 (3:1 petroleum spirits/EtOAc).
^{1}H NMR (700 MHz, $CDCl_3$, 1: 1.1 mixture of rotamers) δ (minor rotamer) 7.90 (d, *J* = 7.7 Hz, 2H), 7.63 (d, *J* = 7.5 Hz, 1H), 7.54 (t, *J* = 7.6 Hz, 2H), 4.67 (s, 2H), 3.10 (s, 3H), 1.24 (s, 9H); (major rotamer) 7.90 (d, *J* = 7.7 Hz, 2H), 7.70 (t, *J* = 7.5 Hz, 1H), 7.59 (d, *J* = 7.7 Hz, 1H), 7.54 (t, *J* = 7.6 Hz, 1H), 4.64 (s, 2H), 3.10 (s, 3H), 1.09 (s, 9H).
^{13}C NMR (176 MHz, $CDCl_3$, 1: 1.1 mixture of rotamers) δ 154.5, 153.6, 137.9, 137.6, 134.3, 134.0, 129.5, 129.2, 129.2, 129.1, 81.4, 81.2, 70.5, 69.6, 35.9, 35.6, 28.1, 27.8.
HRMS (ESI-TOF): calc'd for $C_{13}H_{19}NO_4SNa$ $[M+Na]^+$: 308.0927; found: 308.0924.
IR (neat): 3011, 2978, 2926, 1689, 1293, 1139 cm^{-1}

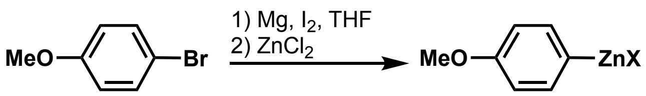

(4-Methoxyphenyl)zinc reagent

To an oven-dried round-bottom flask containing Mg turnings (0.30 g, 12.5 mmol) and a granule of iodine was added dropwise a solution of *p*-bromoanisole (628 µL, 5.0 mmol) in dry THF (5 mL). Following addition, the heterogeneous solution was stirred at room temperature for an additional 1 h. The concentration of the Grignard solution was determined by titration of a small aliquot with a solution of quantitative iodine in dry THF. To a separate oven-dried round-bottom flask, a solution of $ZnCl_2$ (0.27 g, 1.0 M in THF, 2.0 mmol) was added. A portion of the aryl Grignard reagent (2.0 mmol) was added dropwise to the $ZnCl_2$ solution, and the mixture was stirred for 20 min before use. The yield was assumed to be quantitative for the transmetallation step.

Compound **11**

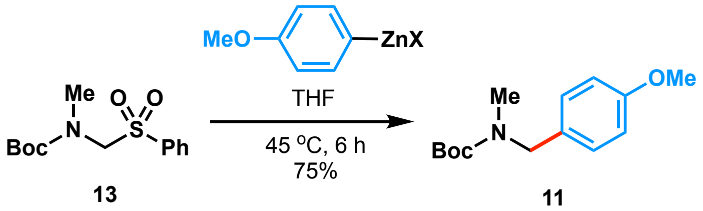

***tert*-Butyl (4-methoxybenzyl)(methyl)carbamate**

A round-bottom flask containing sulfone **13** (57.0 mg, 0.2 mmol, 1.0 equiv.) was backfilled with argon. Dry THF (1 mL) was added to dissolve the solid, along with freshly prepared zinc reagent (5.0 equiv.). The resulting solution was placed in a preheated 45 °C oil bath and stirred for an additional 6 h. The reaction process was monitored by TLC. When complete consumption of the starting material was observed, the mixture was diluted with EtOAc and washed with 0.1 M aq. HCl and brine successively. The organic layer was concentrated under

reduced pressure. Purification by flash column chromatography (silica gel, 4:1 petroleum spirits/EtOAc) afforded the title compound in 75% yield (37.6 mg). *Note: For detailed characterization data please see page 26.*

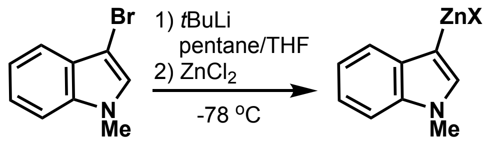

(1-Methyl-1*H*-indol-3-yl)zinc reagent

To a solution of 3-bromo-1-methylindole (420 mg, 2.0 mmol) in dry THF (2 mL) at –78 °C was added *tert*-butyl lithium (1.46 M in pentane, 2.2 mmol) dropwise. The dark yellow solution was stirred at –78 °C for an additional 40 min. A solution of $ZnCl_2$ (0.27 g, 1.0 M in THF, 2.0 mmol) was added at –78 °C and the solution was stirred at –78 °C for an additional 1 h. The yield was assumed to be quantitative for this step.

Compound **14**

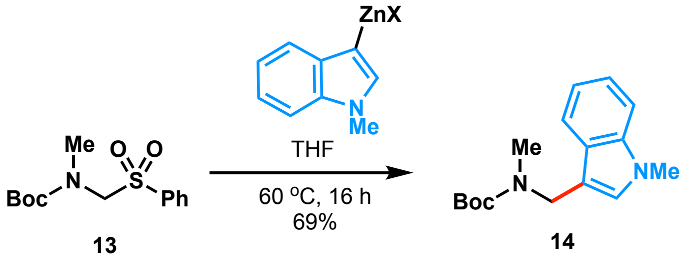

***tert*-Butyl methyl((1-methyl-1*H*-indol-3-yl)methyl)carbamate**

A round-bottom flask containing sulfone **13** (114.0 mg, 0.4 mmol, 1.0 equiv.) was backfilled with argon. Dry THF (1 mL) was added to dissolve the solid. This solution was transferred into the freshly prepared zinc reagent (5.0 equiv.) at room temperature. The resulting solution was placed in a preheated 60 °C oil bath and stirred for an additional 16 h. The reaction process was monitored by TLC. When complete consumption of the starting material was observed, the mixture was diluted with EtOAc, washed with sat. aq. NH_4Cl and brine successively. The organic layer was concentrated under reduced pressure. Purification by flash column chromatography (silica gel, 100% to 5:1 petroleum spirits/EtOAc) afforded the title compound in 69% yield (75.6 mg). *Note: This product is acid-sensitive, and therefore slow decomposition was observed during purification.*

Physical State: yellow oil.
TLC: R_f= 0.56 (4:1 petroleum spirits/EtOAc).
^{1}H NMR (400 MHz, $CDCl_3$) δ 7.69 (br, 1H), 7.33 – 7.27 (m, 1H), 7.25 – 7.20 (m, 1H), 7.14 – 7.09 (m, 1H), 6.98 (br, 1H), 4.58 (s, 2H), 3.77 (s, 3H), 2.77 (s, 3H), 1.51 (s, 9H).
^{13}C NMR (101 MHz, CD_3OD) δ 157.5, 138.8, 129.6, 128.6, 122.7, 120.0, 120.0 (two overlapping signals), 111.6, 110.3, 81.2 & 80.8 (rotameric), 44.7 & 43.9 (rotameric), 33.3, 32.7, 28.8.
Note: CD_3OD was used instead of $CDCl_3$ to obtain the ^{13}C NMR spectrum in order to reduce the rotameric effect.
HRMS (ESI-TOF): calc'd for $C_{16}H_{22}N_2O_2Na$ $[M+Na]^+$: 297.1573; found: 297.1570.

IR (neat): 2973, 2928, 1684, 1139, 740 cm^{-1}

Experimental procedures: tetrapeptide model

Compound **15**

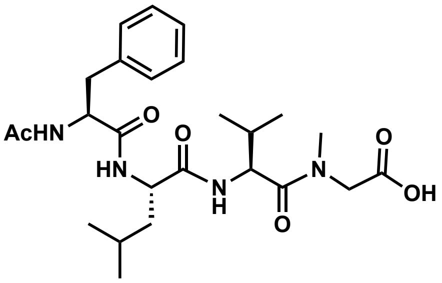

Peptide acid **15** was prepared on a 159 µmol scale on 2-chlorotrityl chloride resin according to the general procedures for SPPS. Following cleavage from the resin and removal of volatiles, the crude peptide was purified by preparative reverse-phase HPLC (50% MeCN to 75% MeCN over 20 min, 10 mL/min) to afford peptide **15** (31.2 mg, 40% yield based on the original resin loading).

Physical State: white fluffy solid (following lyophilization).
^{1}H NMR (400 MHz, CD_3OD) δ 8.20 (d, *J* = 7.9 Hz, 1H, N*H*), 8.09 (d, *J* = 8.8 Hz, 1H, N*H*), 7.30 – 7.15 (m, 5H), 4.75 (t, *J* = 8.1 Hz, 1H), 4.66 (dd, *J* = 9.9, 4.9 Hz, 1H), 4.51 – 4.43 (m, 1H), 4.29 (d, *J* = 17.3 Hz, 1H), 3.93 (d, *J* = 17.2 Hz, 1H), 3.21 (s, 3H), 3.17 – 3.10 (m, 1H), 2.88 – 2.80 (m, 1H), 2.16 – 2.05 (m, 1H), 1.87 (s, 3H), 1.68 – 1.51 (m, 3H), 1.02 – 0.87 (m, 12H).
^{13}C NMR (176 MHz, CD_3OD) δ 174.4, 174.0, 173.7, 173.1, 172.0, 138.5, 130.3, 129.4, 127.7, 55.8, 55.4, 53.2, 50.5, 41.9, 38.8, 37.3, 32.0, 25.8, 23.4, 22.3, 22.1, 19.8, 18.5.
HRMS (ESI-TOF): calc'd for $C_{25}H_{37}N_4O_6^-$ [M-H]$^-$: 489.2708; found: 489.2692.
UPLC trace:

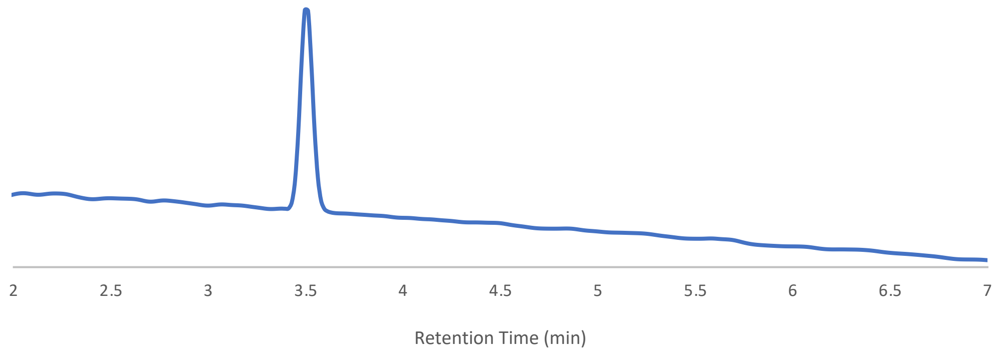

Purified peptide **15** (Rt = 3.5 min, 30% to 85% B over 10 min, λ = 210 nm).

Friedel-Crafts type reaction:

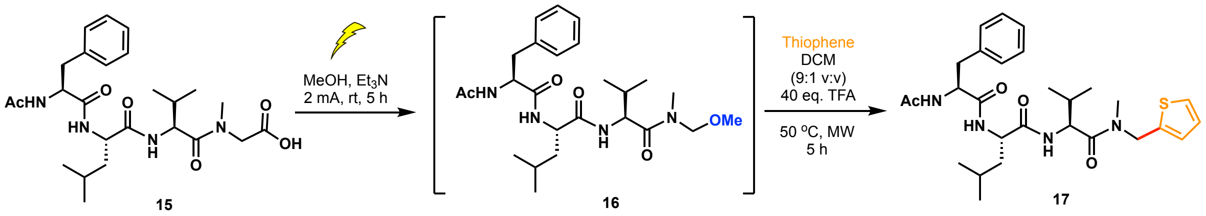

Peptide acid **15** (2.45 mg, 5.0 µmol, 1.0 equiv.) was transferred into the undivided electrochemical cell with MeOH (2 mL), along with a drop of triethylamine (approximately 5 µL) to generate the electrolyte *in situ*. Carbon electrodes (W7 × D1.5 × H55 mm) were attached and the vial was affixed to the Electrasyn. The reaction was electrolyzed under constant current (2 mA, IKA® Electrasyn 2.0 utilized as the potentiostat) until complete consumption of starting material (monitored by UPLC; usually 5 – 7 h) was observed. The crude product **16** was used without further purification in the following transformation.

The crude peptide was transferred to a microwave vessel and the volatiles were removed with a stream of N_2. The vessel was backfilled with argon. Dry DCM/thiophene (1:9 v:v, 1 mL) was added to dissolve the crude *N*,*O*-acetal, before the addition of TFA (15 µL, 40 equiv.). The vessel was quickly capped and irradiated in a monomode microwave cavity (200 W, 50 °C, 5 h). Upon completion, the volatiles were removed under a stream of N_2 and the crude product was purified by preparative reverse-phase HPLC (40% MeCN to 80% MeCN over 20 min, 5 mL/min) to afford peptide **17** in 66% yield (1.73 mg) over 2 steps.

Compound **16**

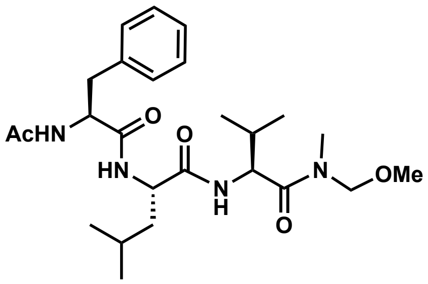

Intermediate **16** was isolated for characterization purposes.
Physical State: white fluffy solid (following lyophilization).
^{1}H NMR (700 MHz, CD_3OD) δ 8.09 – 8.05 (m, 1H, N*H*), 7.28 – 7.23 (m, 4H), 7.19 (t, *J* = 6.9 Hz, 1H), 5.10 (d, *J* = 10.9 Hz, 1H), 4.82 – 4.76 (m, 1H), 4.66 – 4.63 (m, 2H), 4.46 – 4.39 (m, 1H), 3.33 & 3.24 (s, rotameric, 3H), 3.16 & 2.99 (s, rotameric, 3H), 3.15 – 3.12 (m, 1H), 2.84 (dd, *J* = 14.1, 9.6 Hz, 1H), 2.12 – 2.06 (m, 1H), 1.88 (s, 3H), 1.66 – 1.59 (m, 1H), 1.58 – 1.51 (m, 2H), 0.99 – 0.88 (m, 12H).
^{13}C NMR (176 MHz, CD_3OD) δ 174.9, 174.3, 173.7, 173.2, 138.5, 130.2, 129.4, 127.7, 82.3, 79.1, 56.3, 55.8, 53.2, 41.9, 38.7, 34.5, 32.4, 25.8, 23.4, 22.3, 22.0, 19.8, 18.7.
HRMS (ESI-TOF): calc'd for $C_{25}H_{40}N_4O_5Na$ $[M+Na]^+$: 499.2891; found: 499.2881.

UPLC trace:

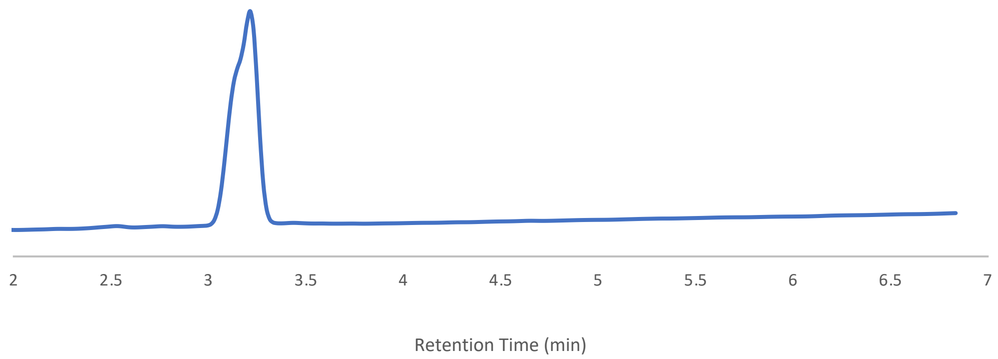

Purified peptide **16** (Rt = 3.2 min, 40% to 85% B over 10 min, λ = 230 nm).

Compound **17**

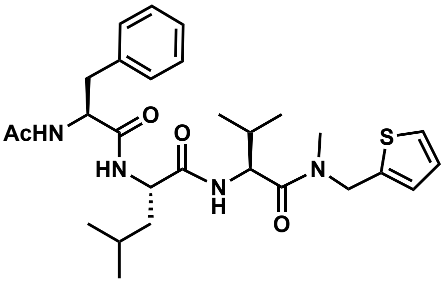

Physical State: light yellow fluffy solid (following lyophilization).

^{1}H NMR (700 MHz, CD_3OD, major rotamer) δ 8.15 (d, *J* = 7.8 Hz, 1H, N*H*), 8.12 – 8.08 (m, 1H, N*H*), 7.30 (dd, *J* = 5.1, 1.2 Hz, 1H), 7.28 – 7.24 (m, 4H), 7.21 – 7.18 (m, 1H), 7.03 – 7.01 (m, 1H), 6.94 (dd, *J* = 5.1, 3.4 Hz, 1H), 4.79 – 4.76 (m, 1H), 4.70 – 4.67 (m, 1H), 4.67 – 4.61 (m, 2H), 4.46 – 4.41 (m, 1H), 3.17 – 3.11 (m, 4H), 2.88 – 2.82 (m, 1H), 2.13 – 2.06 (m, 1H), 1.88 (s, 3H), 1.68 – 1.58 (m, 1H), 1.58 – 1.53 (m, 2H), 0.99 – 0.88 (m, 12H).

^{13}C NMR (176 MHz, CD_3OD) δ 174.4, 173.7, 173.3, 173.2, 140.5, 138.5, 130.3, 129.4, 128.1, 127.7, 127.5, 126.7, 55.8, 55.8, 53.2, 47.0, 42.0, 38.8, 35.5, 32.0, 25.8, 23.4, 22.3, 22.2, 19.7, 18.7.

HRMS (ESI-TOF): calc'd for $C_{28}H_{41}N_4O_4S$ $[M+H]^+$: 529.2849; found: 529.2847.

UPLC trace:

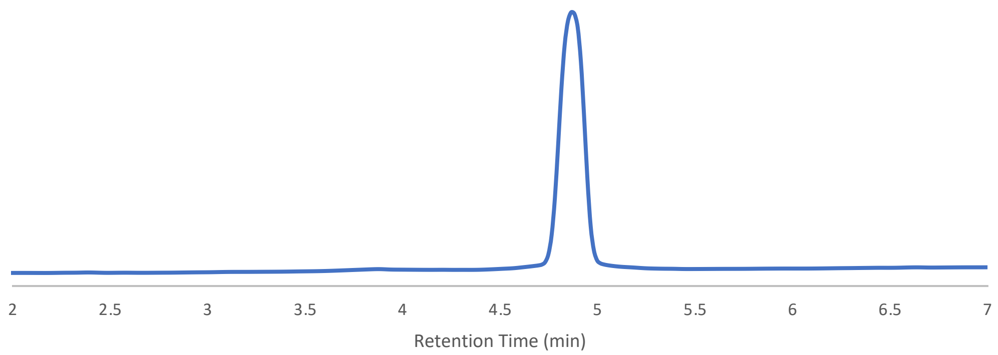

Purified peptide **17** (Rt = 4.9 min, 40% to 85% B over 10 min, λ = 230 nm).

Identified byproduct (hydrolysis of iminium intermediate): Compound **24**

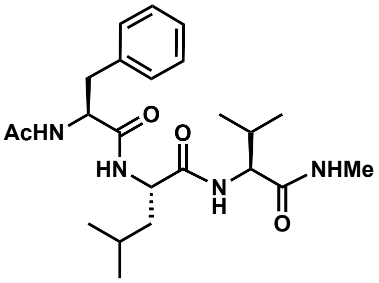

Physical State: white fluffy solid (following lyophilization).
^{1}H NMR (700 MHz, CD_3OD) δ 8.17 (d, *J* = 7.7 Hz, 1H, N*H*), 7.88 (d, *J* = 8.5 Hz, 1H, N*H*), 7.28 – 7.23 (m, 4H), 7.21 – 7.18 (m, 1H), 4.63 (dd, *J* = 9.5, 5.2 Hz, 1H), 4.45 – 4.39 (m, 1H), 4.10 – 4.07 (m, 1H), 3.13 (dd, *J* = 14.1, 5.1 Hz, 1H), 2.86 (dd, *J* = 14.1, 9.5 Hz, 1H), 2.73 (d, *J* = 4.9 Hz, 3H), 2.10 – 1.99 (m, 1H), 1.88 (s, 3H), 1.67 – 1.52 (m, 3H), 0.96 – 0.86 (m, 12H).
^{13}C NMR (176 MHz, CD_3OD) δ 174.4, 174.0, 173.9, 173.2, 138.5, 130.2, 129.4, 127.7, 60.3, 56.0, 53.4, 41.6, 38.7, 32.0, 26.1, 25.8, 23.5, 22.3, 22.0, 19.7, 18.9.
HRMS (ESI-TOF): calc'd for $C_{23}H_{36}N_4O_4Na$ $[M+Na]^+$: 455.2629; found: 455.2622.
UPLC trace:

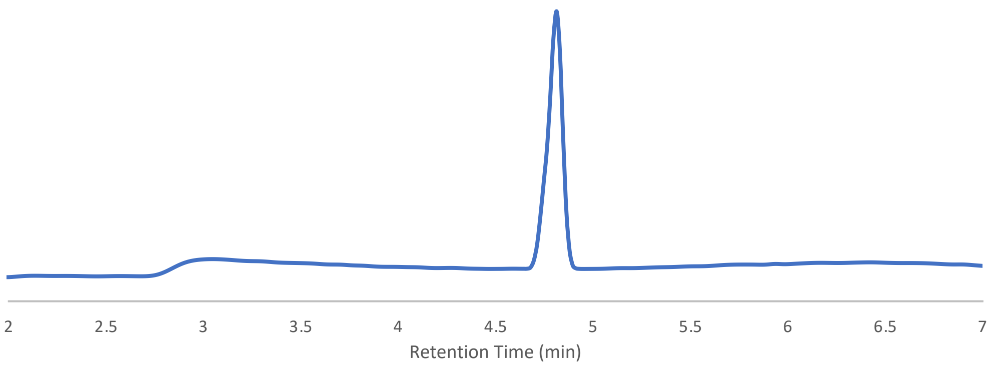

Purified peptide **24** (Rt = 4.8 min, 20% to 80% B over 5 min, λ = 210 nm).

<u>Sulfonylation:</u>

Compound **18**

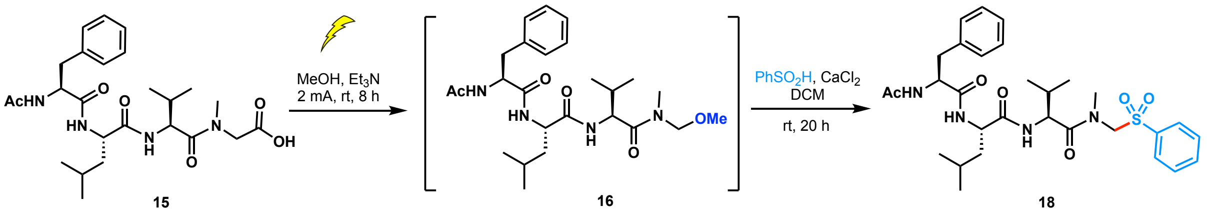

Peptide acid **15** (5.83 mg, 11.8 µmol, 1.0 equiv.) was transferred into the undivided electrochemical cell with MeOH (2 mL), along with a drop of triethylamine (approximately 5 µL) to generate the electrolyte *in situ*. Carbon electrodes (W7 × D1.5 × H55 mm) were attached and the vial was affixed to the Electrasyn. The reaction was electrolyzed under constant current (2 mA, IKA® Electrasyn 2.0 utilized as the potentiostat) until complete consumption of starting

material (monitored by UPLC; usually 8 – 10 h) was observed. The crude product **16** was used without further purification in the following transformation.

The crude peptide was transferred to a 10 mL round-bottom flask and the volatiles were removed under a stream of N_2. To the crude peptide was added freshly prepared $PhSO_2H$ (by acidification of sodium benzenesulfinate, 33.5 mg, 236 µmol, 20 equiv.) and anhydrous $CaCl_2$ (26.2 mg, 236 µmol, 20 equiv.). The flask was backfilled with argon before the addition of dry DCM (1 mL), and the suspension was stirred for 16 – 20 h. Upon completion, the volatiles were removed under a stream of N_2 and the crude product was filtered through Celite® with a small amount of MeOH. The filtrate was concentrated and subsequently purified by reverse-phase HPLC (40% MeCN to 75% MeCN over 20 min, 5 mL/min) to afford peptide **18** in 56% yield (3.84 mg) over 2 steps.

Physical State: white fluffy solid (following lyophilization).
^{1}H NMR (700 MHz, CD_3OD, major rotamer) δ 7.93 – 7.90 (m, 2H), 7.74 – 7.70 (m, 1H), 7.60 (t, J = 7.8 Hz, 2H), 7.29 – 7.17 (m, 5H), 5.23 (d, J = 14.1 Hz, 1H), 4.71 (d, J = 14.1 Hz, 1H), 4.63 (dd, J = 8.6, 6.7 Hz, 2H), 4.40 (ddd, J = 10.0, 7.7, 5.0 Hz, 1H), 3.28 (s, 3H), 3.12 (dd, J = 14.2, 4.9 Hz, 1H), 2.83 (dd, J = 14.1, 9.7 Hz, 1H), 1.98 – 1.92 (m, 1H), 1.87 (s, 3H), 1.67 – 1.60 (m, 1H), 1.60 – 1.55 (m, 1H), 1.53 – 1.48 (m, 1H), 0.96 – 0.78 (m, 12H).
^{13}C NMR (176 MHz, CD_3OD) δ 174.5, 174.5, 174.0, 173.8, 173.7, 173.2, 139.7, 138.5, 135.4, 130.5, 130.2, 129.8, 129.4, 127.7, 69.0, 55.9, 55.8, 55.4, 53.2, 41.9, 41.8, 38.7, 37.4, 31.7, 25.8, 23.5, 22.3, 21.9, 19.8, 18.0.
Note: Rotameric mixture leads to a complex ^{13}C NMR spectrum. All observed signals are reported here.
HRMS (ESI-TOF): calc'd for $C_{30}H_{42}N_4O_6SNa$ $[M+Na]^+$: 609.2717; found: 609.2701.
UPLC trace:

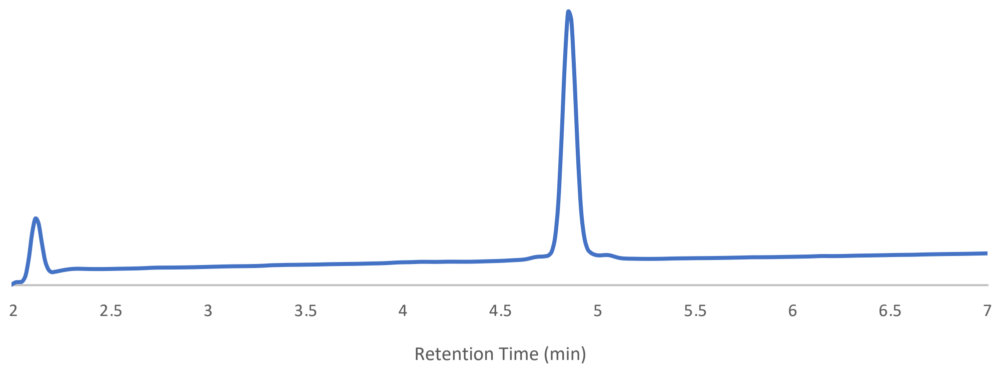

Purified peptide **18** (Rt = 4.9 min, 30% to 85% B over 10 min, λ = 230 nm).

Allylation:

Compound **19**

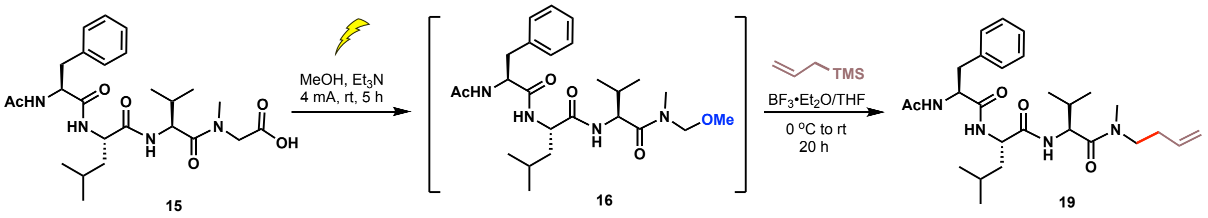

Peptide acid **15** (6.36 mg, 13.0 µmol, 1.0 equiv.) was transferred into the undivided electrochemical cell with MeOH (2 mL), along with a drop of triethylamine (approximately 5 µL) to generate the electrolyte *in situ*. Carbon electrodes (W7 × D1.5 × H55 mm) were attached and the vial was affixed to the Electrasyn. The reaction was electrolyzed under constant current (4 mA, IKA® Electrasyn 2.0 utilized as the potentiostat) until complete consumption of starting material (monitored by UPLC; usually 4 – 6 h) was observed. The crude product **16** was used without further purification in the following transformation.

The crude peptide was transferred to a 10 mL round-bottom flask and the volatiles were removed under a stream of N_2. The flask was backfilled with argon and THF (1 mL) was added. Allyltrimethylsilane (103 µL, 0.65 mmol, 50 equiv.) and BF_3•Et_2O (80 µL, 0.65 mmol, 50 equiv.) was added at 0 °C. The reaction was left stirring at 0 °C for 1 h then warmed to room temperature and stirred for another 16 – 20 h before the addition of Na_2SO_4•$10H_2O$ (around 30 mg). The crude product was filtered through Celite® with a small amount of MeOH. The filtrate was concentrated and subsequently purified by reverse-phase HPLC (40% MeCN to 80% MeCN over 20 min, 5 mL/min) to afford peptide **19** in 27% yield (1.08 mg) over 2 steps.

Physical State: white fluffy solid (following lyophilization).
^{1}H NMR (700 MHz, CD_3OD) δ 8.20 – 8.15 (m, 1H, N*H*), 8.11 (d, *J* = 8.1 Hz, 1H, N*H*), 8.01 – 7.95 (m, 1H, N*H*), 7.28 – 7.24 (m, 4H), 7.21 – 7.17 (m, 1H), 5.87 – 5.74 (m, 1H), 5.17 – 5.11 & 5.09 – 5.04 & 5.03 – 4.99 (m, 2H), 4.71 – 4.61 (m, 2H), 4.46 – 4.42 (m, 1H), 3.62 – 3.56 & 3.55 – 3.46 & 3.39 – 3.34 (m, 2H), 3.17 – 3.12 (m, 1H), 3.15 & 2.92 (s, 3H), 2.87 – 2.82 (m, 1H), 2.44 – 2.35 (m, 1H), 2.33 – 2.27 (m, 1H), 2.05 (dq, *J* = 13.9, 6.9 Hz, 1H), 1.87 (s, 3H), 1.67 – 1.61 (m, 1H), 1.60 – 1.53 (m, 2H), 0.96 – 0.93 (m, 9H), 0.90 (d, *J* = 6.5 Hz, 3H).
^{13}C NMR (176 MHz, CD_3OD) δ 174.4, 174.3, 174.2, 174.2, 173.7, 173.7, 173.4, 173.4, 173.3, 173.3, 173.1, 138.5, 136.6, 135.7, 130.2, 129.4, 127.7, 118.2, 117.3, 55.9, 55.8, 55.8, 55.6, 55.5, 53.3, 53.3, 53.3, 53.2, 50.6, 49.3, 48.8, 41.9, 38.7, 36.4, 34.3, 34.1, 32.7, 32.7, 32.0, 31.9, 25.9, 25.8, 23.4, 22.3, 22.1, 22.0, 19.9, 19.8, 18.6, 18.6.
Note: Rotameric mixture leads to complex ^{1}H & ^{13}C NMR spectra. All observed signals are reported here.
HRMS (ESI-TOF): calc'd for $C_{27}H_{43}N_4O_4$ $[M+H]^+$: 487.3279; found: 487.3266.
UPLC trace:

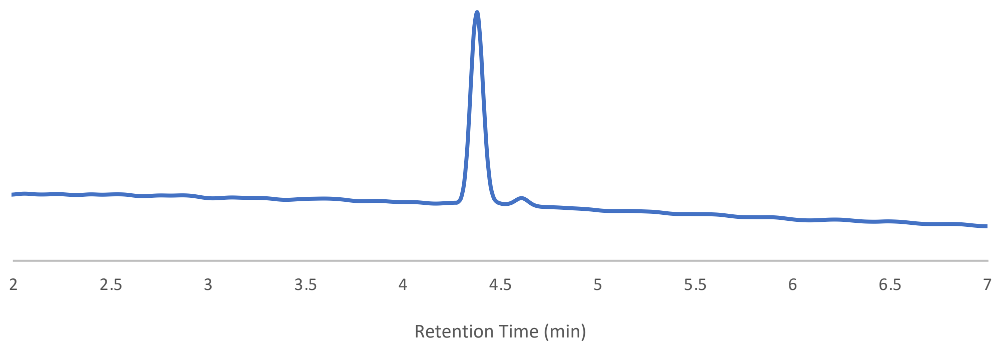

Purified peptide **19** (Rt = 4.4 min, 40% to 85% B over 10 min, λ = 210 nm).

Experimental procedures: natural product analogues

Compound **4**

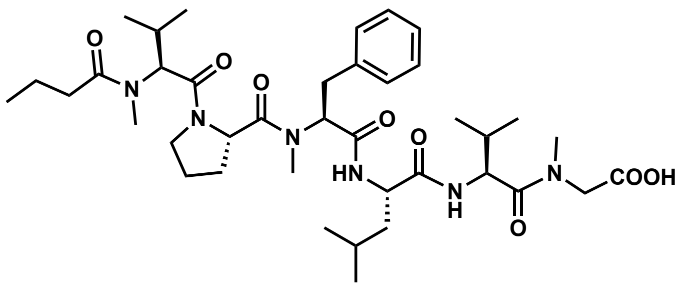

Peptide acid **4** was prepared on a 329 µmol scale on 2-chlorotrityl chloride resin according to the general procedures for SPPS. Following cleavage from the resin and removal of volatiles, the crude peptide was purified by preparative reverse-phase HPLC (60% MeCN to 90% MeCN over 20 min, 10 mL/min) to afford peptide **4** (37.9 mg, 16% yield based on the original resin loading). *Note: Due to the formation of diketopiperazine (DKP), resin loading was reduced after deprotection of the Val Fmoc- group. In order to minimize DKP formation, this deprotection step was modified as follows: The resin was treated with cold piperidine/DMF (1:9 v:v, –20 °C, 1 × 3 min) and washed with DMF (5 × 3 mL), DCM (5 × 3 mL) and DMF (5 × 3 mL). Following washing, a preactivated coupling solution was directly added to the resin and agitated overnight (approximately 16 h).*

Physical State: white fluffy solid (following lyophilization).
^{1}H NMR (600 MHz, CD_3OD) δ 8.91 (d, *J* = 7.5 Hz, 1H, N*H*), 7.87 (d, *J* = 8.9 Hz, 1H, N*H*), 7.34 – 7.29 (m, 2H), 7.26 – 7.20 (m, 3H), 5.18 (dd, *J* = 11.7, 3.2 Hz, 1H), 4.98 (d, *J* = 10.9 Hz, 1H), 4.78 – 4.74 (m, 1H), 4.62 – 4.55 (m, 1H), 4.41 (ddd, *J* = 11.6, 7.4, 4.1 Hz, 1H), 4.25 (d, *J* = 17.3 Hz, 1H), 4.01 (d, *J* = 17.3 Hz, 1H), 3.78 (ddd, *J* = 10.5, 7.5, 4.6 Hz, 1H), 3.54 (dt, *J* = 10.4, 7.5 Hz, 1H), 3.25 – 3.18 (m, 4H), 3.03 – 2.95 (m, 4H), 2.84 (s, 3H), 2.43 – 2.36 (m, 1H), 2.36 – 2.29 (m, 1H), 2.23 – 2.16 (m, 1H), 2.14 – 2.08 (m, 1H), 1.88 – 1.78 (m, 2H), 1.75 – 1.68 (m, 1H), 1.64 – 1.53 (m, 4H), 1.01 (d, *J* = 6.8 Hz, 3H), 0.97 – 0.88 (m, 17H), 0.81 (d, *J* = 6.7 Hz, 3H).
^{13}C NMR (151 MHz, CD_3OD) δ 176.2, 175.4, 175.3, 174.0, 172.0, 171.9, 170.4, 139.4, 130.7, 129.9, 128.0, 64.4, 60.6, 56.9, 55.4, 54.1, 50.7, 41.3, 37.3, 36.4, 35.5, 34.6, 32.1, 31.5, 30.1, 29.8, 29.0, 26.1, 25.9, 23.7, 21.0, 19.9, 19.6, 19.5, 18.8, 18.4, 14.1.
HRMS (ESI-TOF): calc'd for $C_{39}H_{62}N_6O_8Na$ $[M+Na]^+$: 765.4527; found: 765.4519.
UPLC trace:

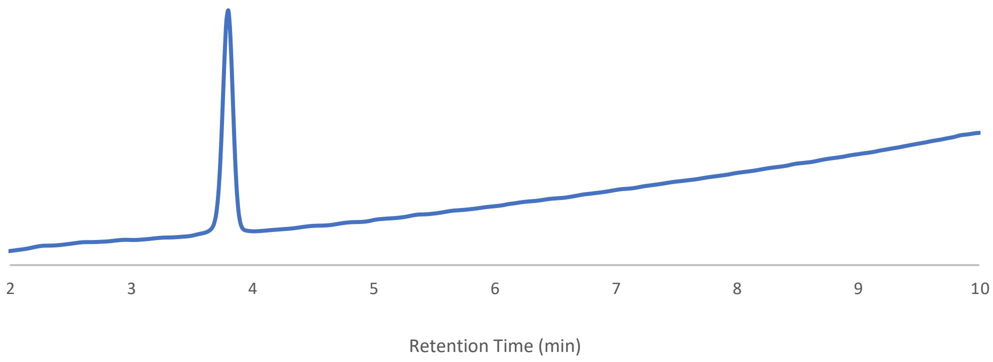

Purified peptide **4** (Rt = 3.8 min, 50% to 95% B over 10 min, λ = 230 nm).

Screening conditions for decreasing diketopiperazine (DKP) formation:

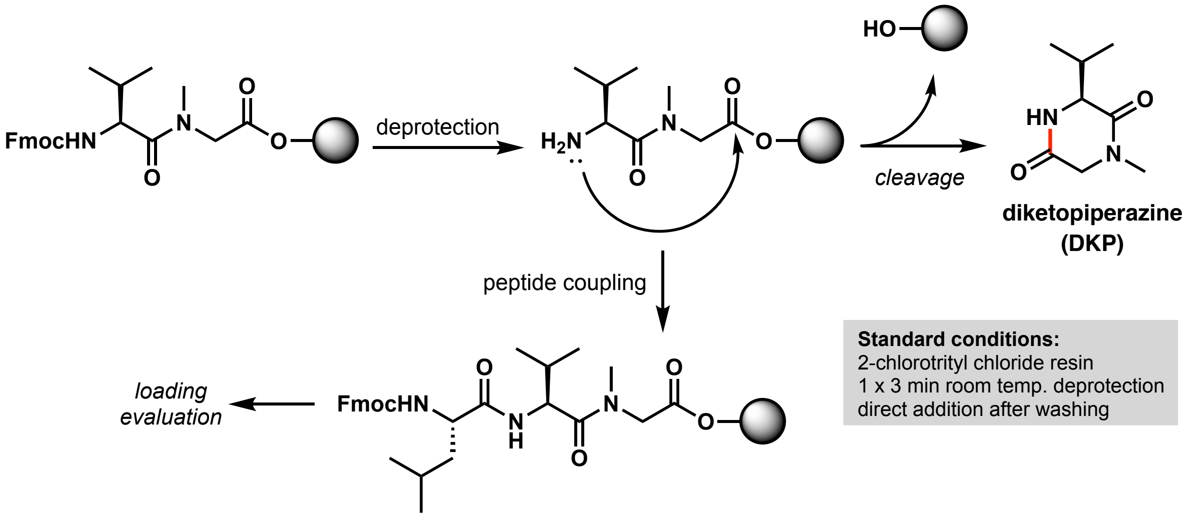

Entry	Variation from standard	Percentage of loading preserved[a]
1	–	68%
2	wang resin	53%
3	5 min room temp. standing[b] before adding coupling reagents	43%
4	deprotection solution cooled to –20 °C	86%

[a] Based on resin loading. [b] After washing with DMF, DCM and DMF, the resin was filtered and left on bench.

Compound **20**

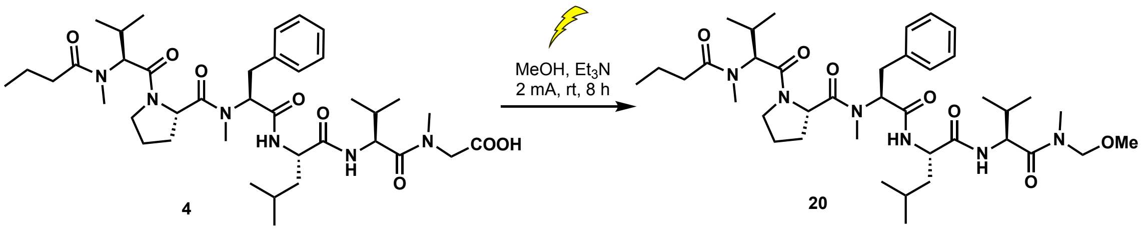

Peptide acid **4** (7.33 mg, 9.9 µmol, 1.0 equiv.) was transferred into the undivided electrochemical cell with MeOH (2 mL), along with a drop of triethylamine (approximately 5 µL) to generate the electrolyte *in situ*. Carbon electrodes (W7 × D1.5 × H55 mm) were attached and the vial was affixed to the Electrasyn. The reaction was electrolyzed under constant current (2 mA, IKA® Electrasyn 2.0 utilized as the potentiostat) until complete consumption of starting material (monitored by UPLC; usually 8 – 10 h) was observed. The reaction mixture was concentrated and purified by preparative reverse-phase HPLC (65% MeCN to 85% MeCN over 20 min, 5 mL/min) to afford peptide **20** in 80% yield (5.76 mg).

Physical State: white fluffy solid (following lyophilization).
^{1}H NMR (700 MHz, CD_3OD) δ 7.32 (t, *J* = 7.5 Hz, 2H), 7.27 – 7.20 (m, 3H), 5.17 (dd, *J* = 11.6, 3.2 Hz, 1H), 5.10 & 4.98 (d, *J* = 10.8 Hz, 1H), 4.86 – 4.85 (overlapping with water

signal) & 4.78 (d, J = 9.8 Hz, 1H), 4.72 – 4.66 (m, 2H), 4.57 (t, J = 7.2 Hz, 1H), 4.41 (td, J = 11.0, 4.3 Hz, 1H), 3.81 – 3.76 (m, 1H), 3.57 – 3.51 (m, 1H), 3.34 & 3.27 (s, rotameric, 3H), 3.23 – 3.18 (m, 1H), 3.17 & 3.01 (s, rotameric, 3H), 3.01 – 2.96 (m, 1H), 3.00 (s, 3H), 2.84 (s, 3H), 2.40 (dt, J = 15.2, 7.5 Hz, 1H), 2.36 – 2.30 (m, 1H), 2.19 (dt, J = 11.8, 6.3 Hz, 1H), 2.09 (tt, J = 14.2, 7.0 Hz, 1H), 1.84 (dq, J = 11.9, 6.0 Hz, 1H), 1.79 (ddd, J = 13.7, 11.6, 4.8 Hz, 1H), 1.74 – 1.67 (m, 1H), 1.66 – 1.59 (m, 2H), 1.58 – 1.51 (m, 2H), 1.01 – 0.87 (m, 20H), 0.81 (d, J = 6.7 Hz, 3H).

Note: Rotameric mixture leads to a complex ^{1}H NMR spectrum.

^{13}C NMR (176 MHz, CD_3OD) δ 176.2, 175.3, 175.2, 175.1, 171.9, 170.3, 139.4, 130.6, 129.9, 128.0, 82.2, 64.4, 64.3, 60.6, 56.8, 56.4, 56.2, 56.0, 55.8, 53.9, 53.9, 48.6, 41.3, 36.4, 34.7, 34.5, 34.4, 32.5, 31.8, 31.4, 30.1, 30.1, 29.8, 29.0, 28.9, 26.1, 25.8, 23.7, 21.0, 21.0, 19.8, 19.8, 19.6, 19.6, 19.5, 18.8, 18.8, 18.7, 18.5, 14.1.

Note: Rotameric mixture leads to a complex ^{13}C NMR spectrum.

HRMS (ESI-TOF): calc'd for $C_{39}H_{64}N_6O_7Na$ $[M+Na]^+$: 751.4729; found: 751.4715.

UPLC trace:

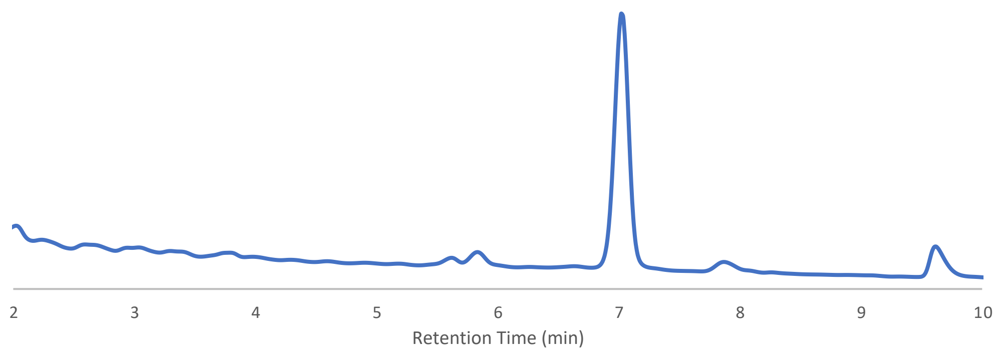

Crude UPLC trace of the formation of *N*,*O*-acetal (40% to 85% B over 10 min, λ = 210 nm).

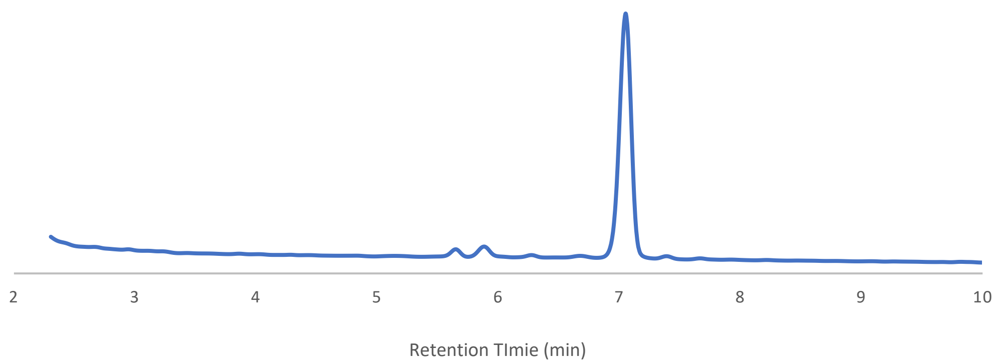

Purified peptide **20** (Rt = 7.1 min, 40% to 85% B over 10 min, λ = 210 nm).

Compound **21**

(stepwise protocol)

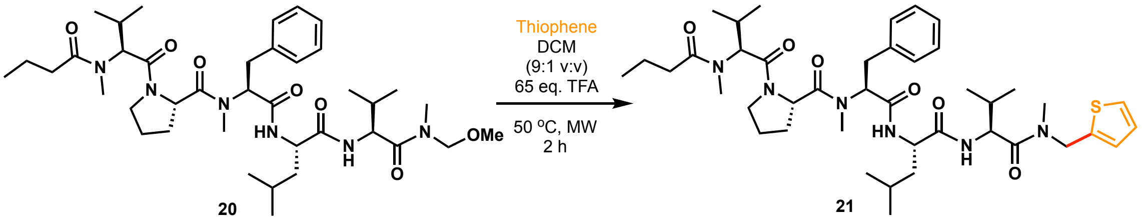

Peptide *N*,*O*-acetal **20** (2.22 mg, 3.04 µmol, 1.0 equiv.) was transferred into a microwave vessel. Following lyophilization, the vessel was backfilled with argon. Dry DCM/thiophene (1:9 v:v, 1 mL) was added to dissolve the crude *N*,*O*-acetal before the addition of TFA (15 µL, 65 equiv.). The vessel was quickly capped and irradiated in a monomode microwave cavity (200 W, 50 °C, 2 h). Upon completion, the volatiles were removed under a stream of N_2 and the crude product was purified by preparative reverse-phase HPLC (65% MeCN to 85% MeCN over 20 min, 5 mL/min) to afford peptide **21** in 25% yield (0.59 mg).

(two-steps protocol)

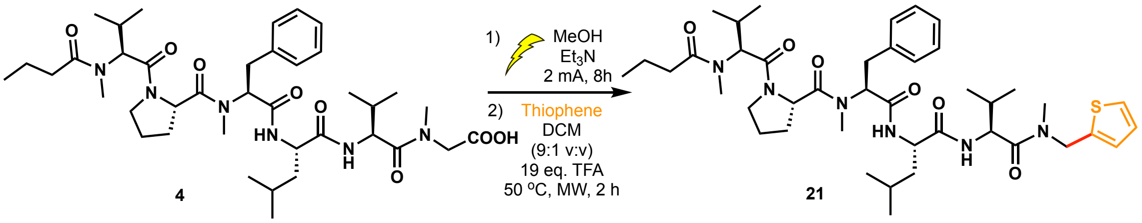

Peptide acid **4** (7.70 mg, 10.4 µmol, 1.0 equiv.) was transferred into the undivided electrochemical cell with MeOH (2 mL), along with a drop of triethylamine (approximately 5 µL) to generate the electrolyte *in situ*. Carbon electrodes (W7 × D1.5 × H55 mm) were attached and the vial was affixed to the Electrasyn. The reaction was electrolyzed under constant current (2 mA, IKA® Electrasyn 2.0 utilized as the potentiostat) until complete consumption of starting material (monitored by UPLC; usually 5 – 7 h) was observed. The crude product was used without further purification in the following transformation.

The crude peptide was transferred to a microwave vessel and the volatiles were removed with a stream of N_2. Following lyophilization, the vessel was backfilled with argon. Dry DCM/thiophene (1:9 v:v, 1 mL) was added to dissolve the crude *N*,*O*-acetal, before the addition of TFA (15 µL, 19 equiv.). The vessel was quickly capped and irradiated in a monomode microwave cavity (200 W, 50 °C, 5 h). Upon completion, the volatiles were removed under a stream of N_2 and the crude product was purified by preparative reverse-phase HPLC (40% MeCN to 80% MeCN over 20 min, 5 mL/min) to afford peptide **21** in 18% yield (1.46 mg) over 2 steps.

Physical State: white fluffy solid (following lyophilization).
^{1}H NMR (700 MHz, CD_3OD) δ 8.96 – 8.91 (m, 1H, N*H*), 8.00 (d, *J* = 8.7 Hz, 1H, N*H*), 7.33 – 7.29 (m, 2H), 7.28 – 7.18 (m, 4H), 7.10 – 7.03 (m, 1H), 7.02 – 6.93 (m, 1H), 5.18 (dd, *J* = 11.7, 3.3 Hz, 1H), 5.04 – 4.95 (m, 1H), 4.83 – 4.72 (m, 2H), 4.71 – 4.66 (m, 1H), 4.62 – 4.56 (m, 1H), 4.45 – 4.39 (m, 1H), 3.82 – 3.76 (m, 1H), 3.58 – 3.49 (m, 1H), 3.26 – 3.20 (m, 1H), 3.17 –

3.12 (m, 2H), 3.02 – 2.93 (m, 5H), 2.87 – 2.82 (m, 3H), 2.40 – 2.33 (m, 1H), 2.32 – 2.26 (m, 1H), 2.22 – 2.14 (m, 1H), 2.13 – 2.06 (m, 1H), 1.88 – 1.76 (m, 2H), 1.75 – 1.69 (m, 1H), 1.64 – 1.52 (m, 4H), 0.99 – 0.79 (m, 23H), .
^{13}C NMR (176 MHz, CD_3OD) δ 176.2, 175.3, 175.3, 173.4, 172.0, 170.3, 140.5, 139.4, 130.6, 129.9, 128.1, 128.0, 127.6, 126.8, 64.4, 60.6, 56.9, 55.7, 54.0, 49.1 (assigned through HSQC), 47.1, 41.5, 36.4, 35.5, 34.6, 32.0, 31.4, 30.1, 29.8, 29.0, 26.2, 25.9, 23.8, 21.1, 19.7, 19.6, 19.5, 18.9, 18.6, 14.2.
HRMS (ESI-TOF): calc'd for $C_{42}H_{64}N_6O_6SNa$ $[M+Na]^+$: 803.4506; found: 803.4509.
UPLC trace:

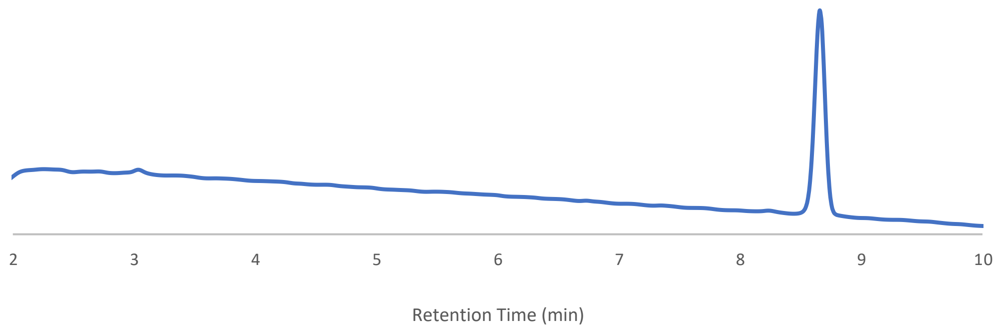

Purified peptide **21** (Rt = 8.7 min, 40% to 85% B over 10 min, λ = 210 nm).

Compound **22**

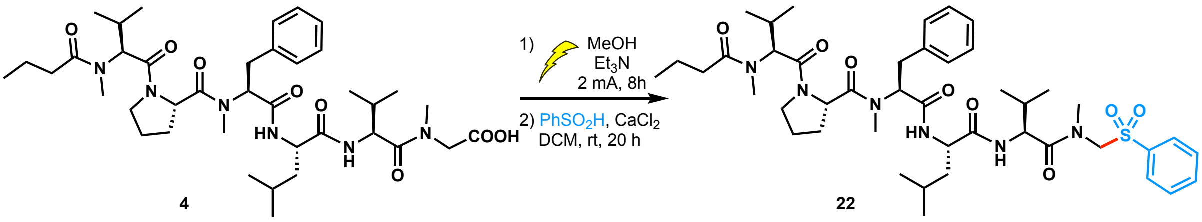

Peptide acid **4** (8.89 mg, 12.0 μmol, 1.0 equiv.) was transferred into the undivided electrochemical cell with MeOH (2 mL), along with a drop of triethylamine (approximately 5 μL) to generate the electrolyte *in situ*. Carbon electrodes (W7 × D1.5 × H55 mm) were attached and the vial was affixed to the Electrasyn. The reaction was electrolyzed under constant current (2 mA, IKA® Electrasyn 2.0 utilized as the potentiostat) until complete consumption of starting material (monitored by UPLC; usually 8 – 10 h) was observed. The crude product was used without further purification in the following transformation.

The crude peptide was transferred to a 10 mL round-bottom flask and the volatiles were removed under a stream of N_2. To the crude peptide was added freshly prepared $PhSO_2H$ (by acidification of sodium benzenesulfinate, 34.1 mg, 240 μmol, 20 equiv.) and anhydrous $CaCl_2$ (26.6 mg, 240 μmol, 20 equiv.). The flask was backfilled with argon before the addition of dry DCM (1 mL), and the suspension was stirred for 16 – 20 h. Upon completion, the volatiles were removed under a stream of N_2 and the crude was filtered through Celite® with a small amount of MeOH. The filtrate was concentrated and subsequently purified by reverse-phase HPLC (65% MeCN to 85% MeCN over 20 min, 5 mL/min) to afford peptide **22** in 21% yield (2.13 mg) over 2 steps.

Physical State: white fluffy solid (following lyophilization).
^{1}H NMR (700 MHz, CD$_3$OD, major rotamer) δ 8.85 (d, *J* = 7.4 Hz, 1H, N*H*), 7.97 – 7.94 (m, 2H), 7.80 – 7.70 (m, 2H), 7.65 (t, *J* = 7.8 Hz, 1H), 7.29 – 7.24 (m, 2H), 7.24 – 7.17 (m, 3H), 5.16 (dd, *J* = 11.6, 3.3 Hz, 1H), 5.08 (d, *J* = 14.1 Hz, 1H), 4.98 (d, *J* = 10.9 Hz, 1H), 4.91 (d, *J* = 14.0 Hz, 1H), 4.70 – 4.66 (m, 1H), 4.60 – 4.56 (m, 1H), 4.41 (ddd, *J* = 11.6, 7.3, 4.2 Hz, 1H), 3.76 (ddd, *J* = 11.7, 7.4, 4.8 Hz, 1H), 3.54 (dt, *J* = 10.5, 7.4 Hz, 1H), 3.25 (s, 3H), 3.01 (s, 3H), 2.99 – 2.90 (m, 2H), 2.84 (s, 3H), 2.42 – 2.36 (m, 1H), 2.35 – 2.30 (m, 1H), 2.24 – 2.15 (m, 1H), 2.03 – 1.97 (m, 1H), 1.87 – 1.79 (m, 2H), 1.74 – 1.68 (m, 1H), 1.65 – 1.58 (m, 2H), 1.56 – 1.51 (m, 2H), 0.97 – 0.76 (m, 23H).
HRMS (ESI-TOF): calc'd for $C_{44}H_{66}N_6O_8SNa$ $[M+Na]^+$: 861.4555; found: 861.4552.
UPLC trace:

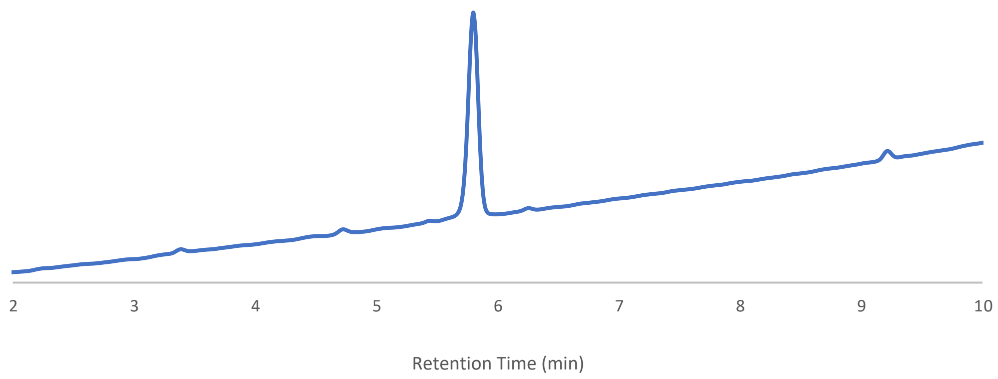

Purified peptide **22** (Rt = 5.8 min, 50% to 95% B over 10 min, λ = 230 nm).

Identified byproduct (hydrolysis of iminium intermediate): Compound **23**

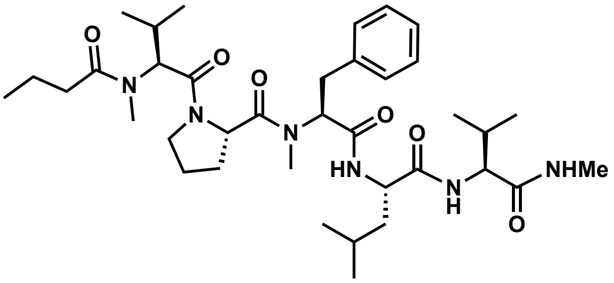

Physical State: white fluffy solid (following lyophilization).
^{1}H NMR (700 MHz, CD$_3$OD) δ 7.32 (t, *J* = 7.6 Hz, 2H), 7.26 – 7.20 (m, 3H), 5.18 (dd, *J* = 12.3, 2.8 Hz, 1H), 4.98 (d, *J* = 10.9 Hz, 1H), 4.57 (t, *J* = 7.3 Hz, 1H), 4.45 – 4.38 (m, 1H), 4.13 (d, *J* = 7.8 Hz, 1H), 3.82 – 3.76 (m, 1H), 3.59 – 3.50 (m, 1H), 3.21 (dd, *J* = 14.5, 3.2 Hz, 1H), 3.03 – 2.97 (m, 4H), 2.84 (s, 3H), 2.75 (s, 3H), 2.42 – 2.36 (m, 1H), 2.36 – 2.29 (m, 1H), 2.22 – 2.16 (m, 1H), 2.04 – 1.97 (m, 1H), 1.87 – 1.78 (m, 2H), 1.76 – 1.65 (m, 1H), 1.65 – 1.53 (m, 4H), 0.97 – 0.93 (m, 14H), 0.92 (d, *J* = 6.6 Hz, 3H), 0.89 (d, *J* = 6.5 Hz, 3H), 0.81 (d, *J* = 6.7 Hz, 3H).
^{13}C NMR (176 MHz, CD$_3$OD, major rotamer) δ 176.2, 175.3, 174.1, 172.1, 172.0, 170.3, 139.4, 130.6, 129.9, 128.0, 64.3, 60.6, 60.2, 56.8, 54.0, 48.9, 41.2, 36.4, 34.7, 32.4, 31.4, 30.1, 29.8, 28.9, 26.2, 26.1, 25.8, 23.8, 21.0, 19.7, 19.6, 19.5, 19.0, 18.8, 14.1.
HRMS (ESI-TOF): calc'd for $C_{37}H_{60}N_6O_6Na$ $[M+Na]^+$: 707.4472; found: 707.4485.

UPLC trace:

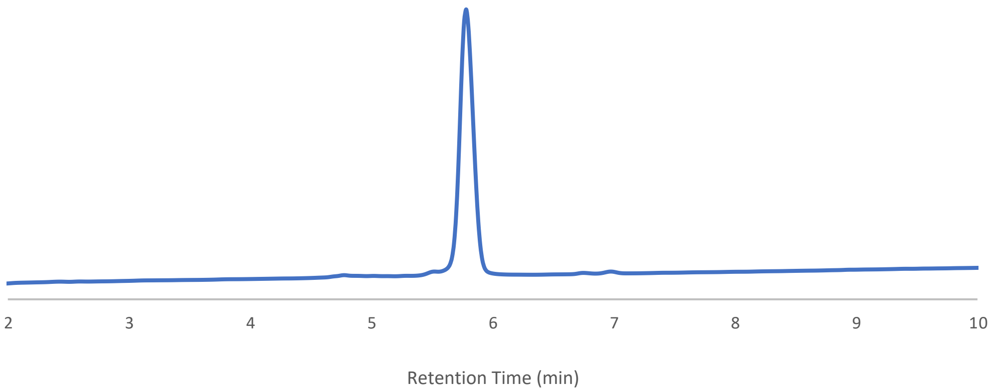

Purified peptide **23** (Rt = 5.8 min, 40% to 85% B over 10 min, λ = 230 nm).

Evaluation of biological activity

Cell Culture of A549 and HeLa S3 Cells

1. A549 cells (ATCC® CCL-185) were cultured at 37 °C with 5% CO_2 in DMEM/F12 (Ham) medium (Gibco, CAT#11330-032) supplemented with 10% fetal bovine serum (FBS) (Sigma-Aldrich, CAT#F9423) and 2 mM GlutaMAX (Gibco, CAT#35050061).

2. HeLa S3 cells were cultured at 37 °C with 5% CO_2 in F-12K nutrient mixture, Kaighn's modification medium (Gibco, CAT#21127-022) supplemented with 10% fetal bovine serum (FBS) (Sigma-Aldrich, CAT#F9423).

3. To subculture cells, media was aspirated and cell monolayers were washed once with dPBS (Gibco), and then detached using Trypsin-EDTA (0.25%, Gibco CAT#25200114), followed by neutralisation with growth media. Quantitation of cell number was obtained using the Life Technologies Countess II automated cell counter.

MTT 72 Hour Viability Assay

1. A549 and HeLa S3 cells were seeded at 600 cells/well in 40 µL into a Corning clear 384-well plate (CAT# 3701) using the Biotek EL406, and cultured overnight.

2. Cells were then treated with various concentrations of compounds for 72 h. 0.2% DMSO and 25 µM Etoposide (Sigma-Aldrich) were used as controls.

3. 10 µL of MTT reagent (ThermoFisher, diluted to 2.5 mg/mL in DPBS) was then added to cells and incubated at 37 °C for 1.5 h.

4. Plates were then centrifuged at 800 × g for 5 min and the supernatant flicked off. 25 µL of isopropanol/HCl solution was then added to each well using the Biotek EL406 and the plates were shaken for 20 s.

5. Absorbance was then read at 570 nm using the PerkinElmer EnVision Multimode plate reader.

6. *Statistical Analysis:* Results were normalised to the average blank value (media only control), and then presented as a percentage of the control (0.2% DMSO (vehicle) treated cells) (percentage viability).

Dose-response curves (MTT 72 Hour Viability Assay)

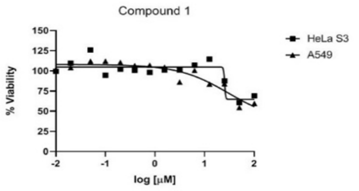

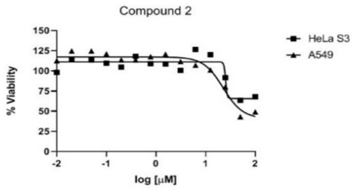

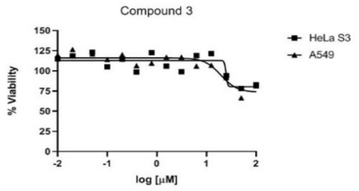

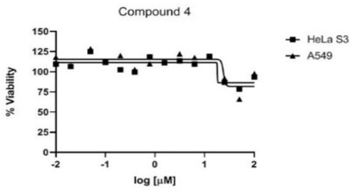

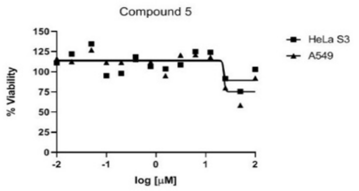

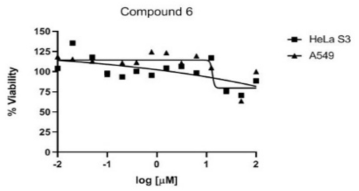

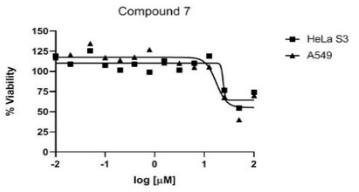

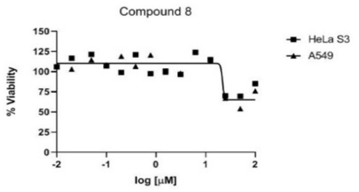

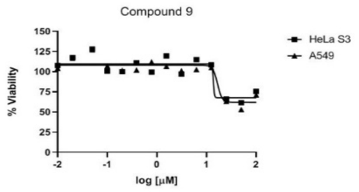

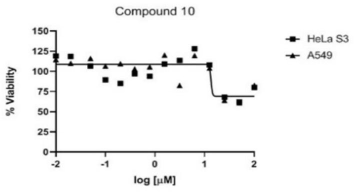

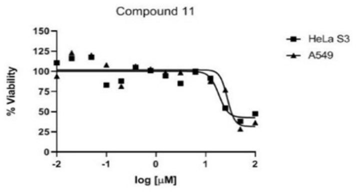

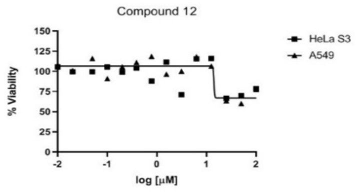

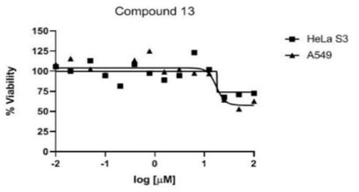

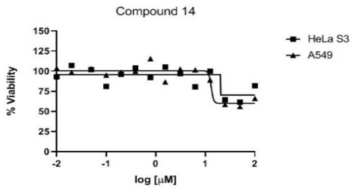

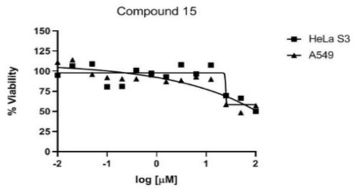

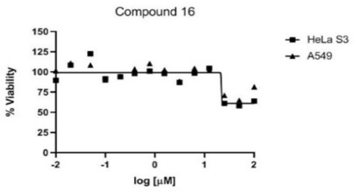

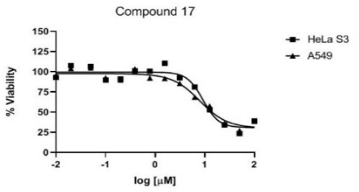

References

[1] J. Han, J. Lian, X. Tian, S. Zhou, X. Zhen, S. Liu, *Eur. J. Org. Chem.* **2014**, *2014*, 7232-7238.

[2] M. Keller, S. Teng, G. Bernhardt, A. Buschauer, *Chem. Med. Chem.* **2009**, *4*, 1733-1745.

[3] A. Iwasaki, T. Tadenuma, S. Sumimoto, T. Ohshiro, K. Ozaki, K. Kobayashi, T. Teruya, H. Tomoda, K. Suenaga, *J. Nat. Prod.* **2017**, *80*, 1161-1166.

[4] A. Millet, D. Dailler, P. Larini, O. Baudoin, *Angew. Chem. Int. Ed. Engl.* **2014**, *53*, 2678-2682.

[5] S. A. Baker Dockrey, A. L. Lukowski, M. R. Becker, A. R. H. Narayan, *Nat. Chem.* **2017**, *10*, 119-125.

[6] J. M. Saa, G. Martorell, A. Garcia-Raso, *J. Org. Chem.* **1992**, *57*, 678-685.

NMR spectral data

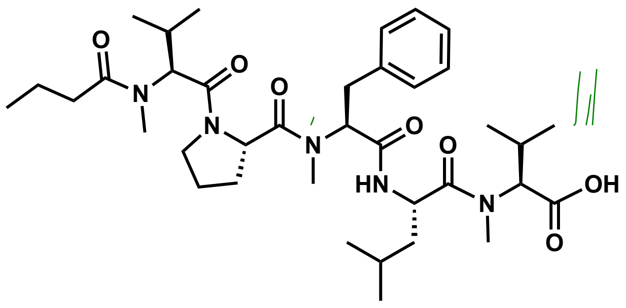

2a, ^{1}H, CD_3OD, 600 MHz

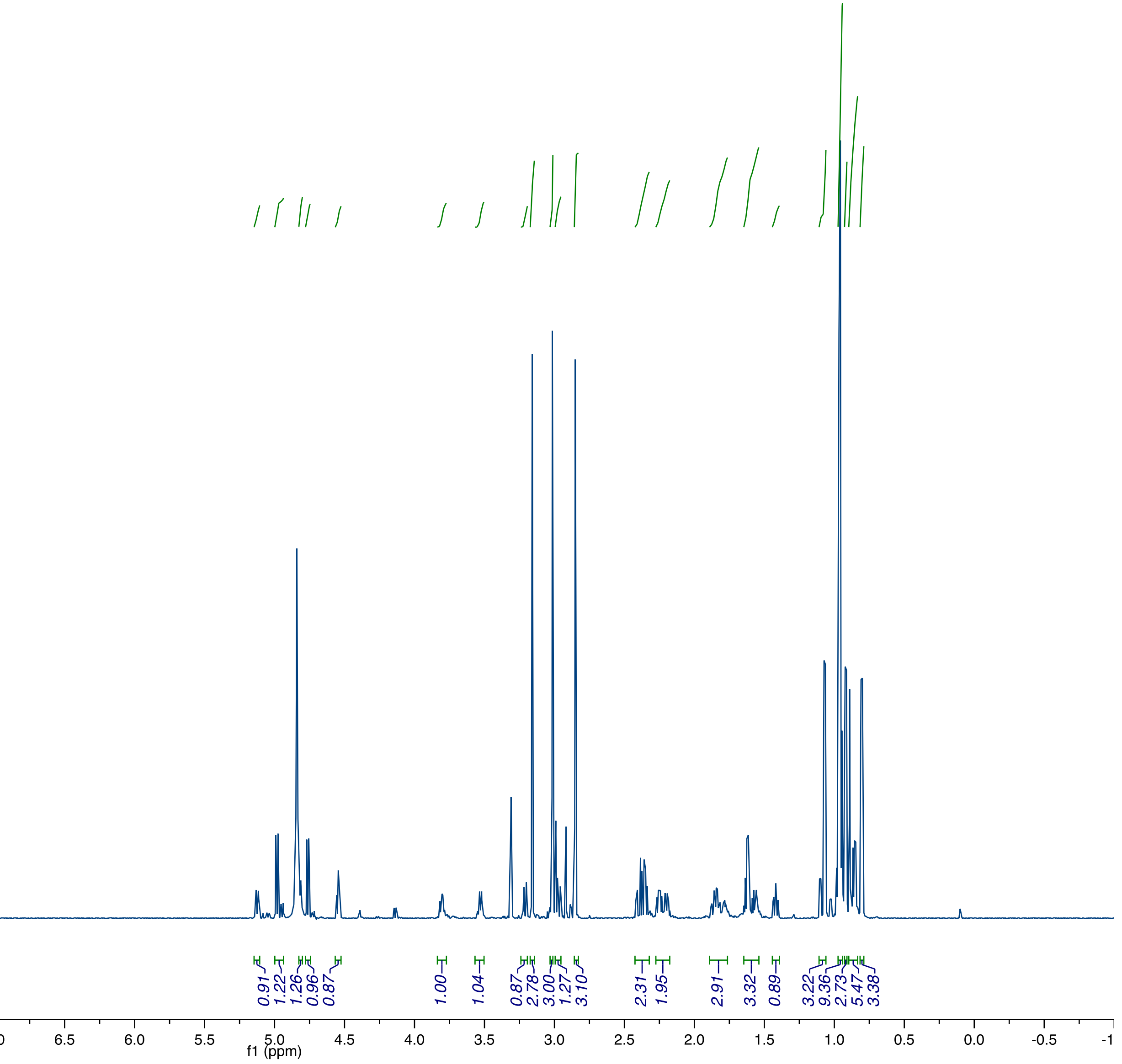

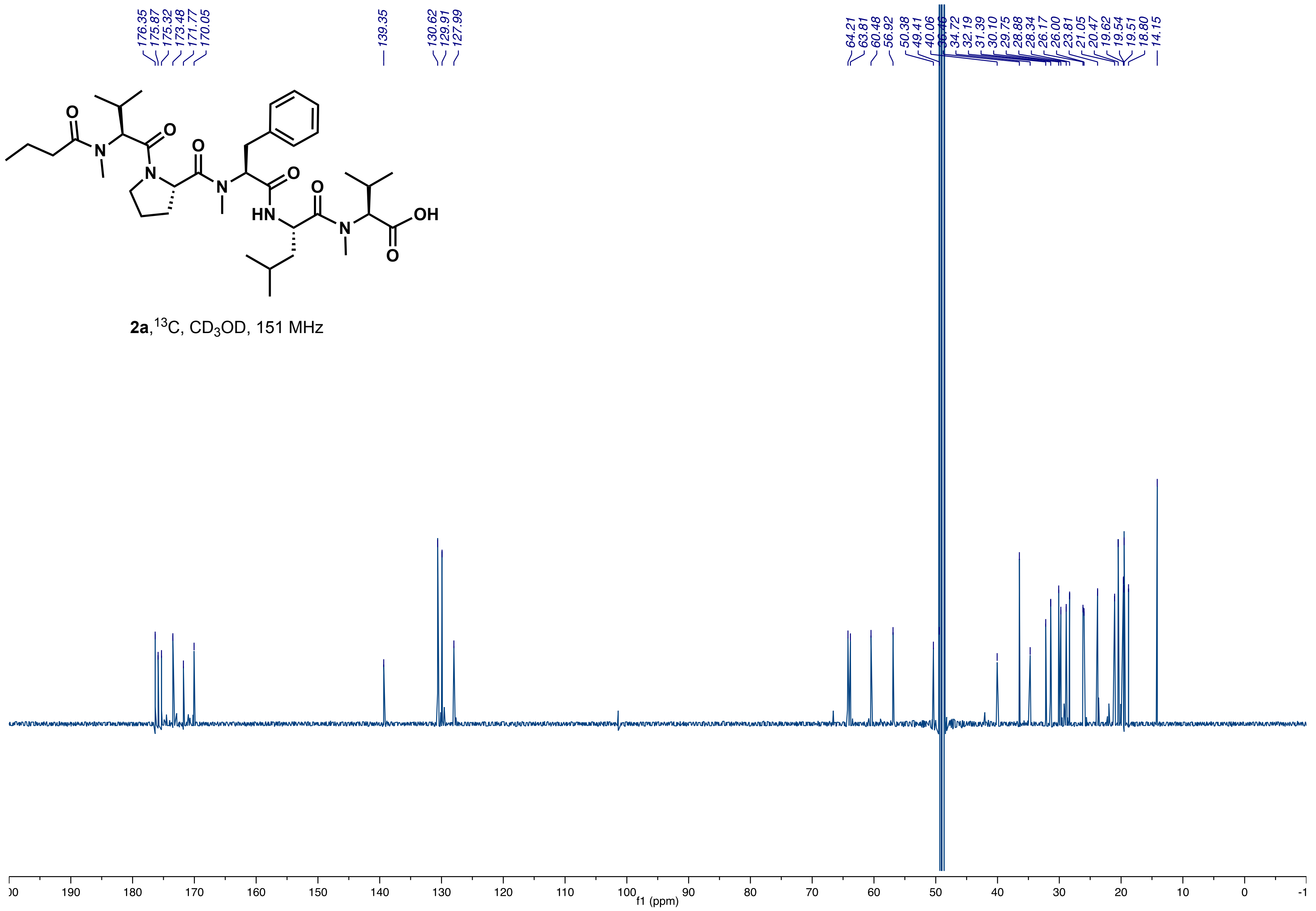
176.35
175.87
175.32
173.48
171.77
170.05
139.35
130.62
129.91
127.99
64.21
63.81
60.48
56.92
50.38
49.41
40.06
34.72
32.19
31.39
30.10
29.75
28.88
28.34
26.17
26.00
23.81
21.05
20.47
19.62
19.54
19.51
18.80
14.15
2a, ^{13}C, CD_3OD, 151 MHz
f1 (ppm)

2a, COSY, CD_3OD, 600 MHz

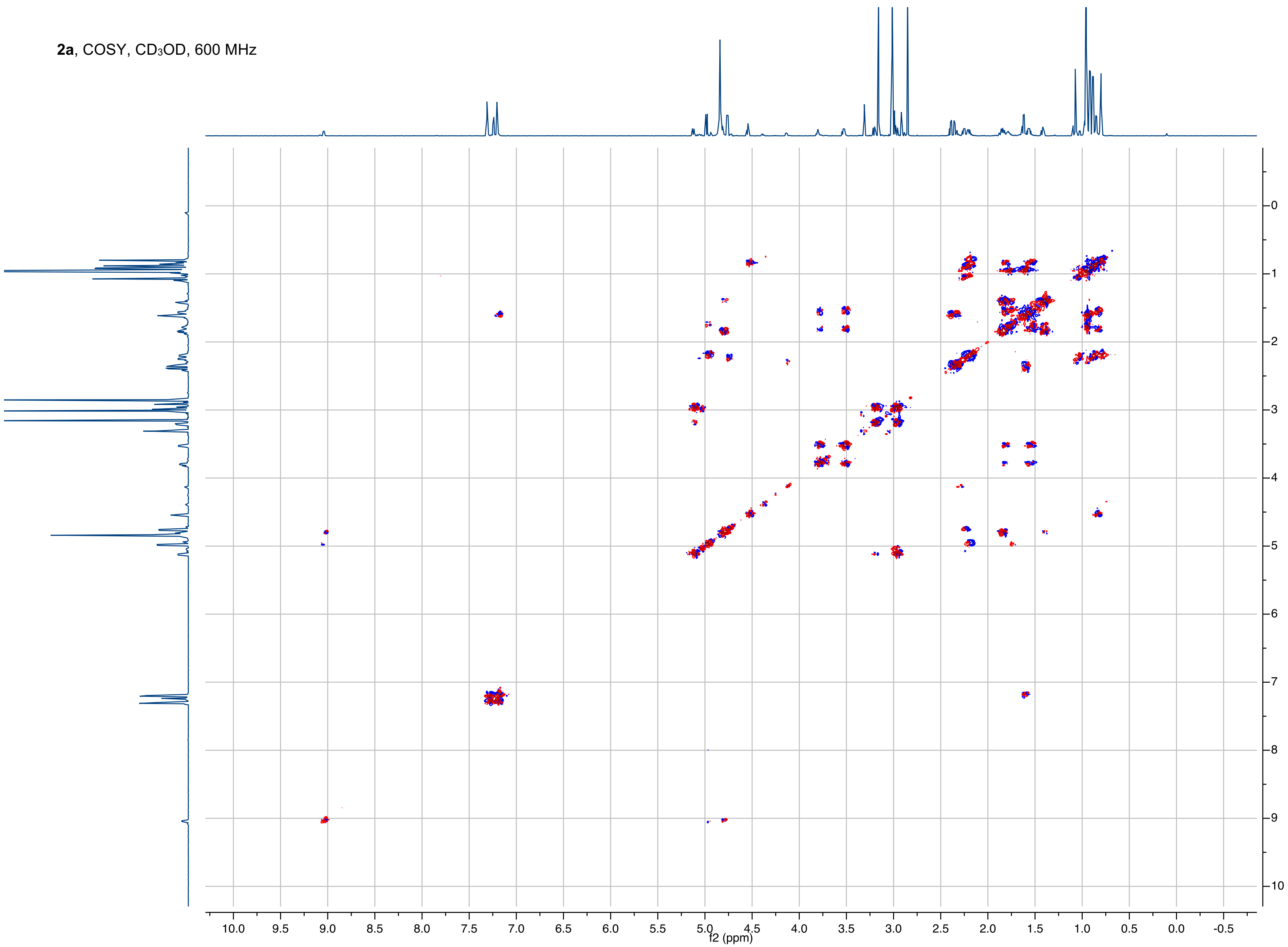

2a, HSQC, CD_3OD, 600 MHz

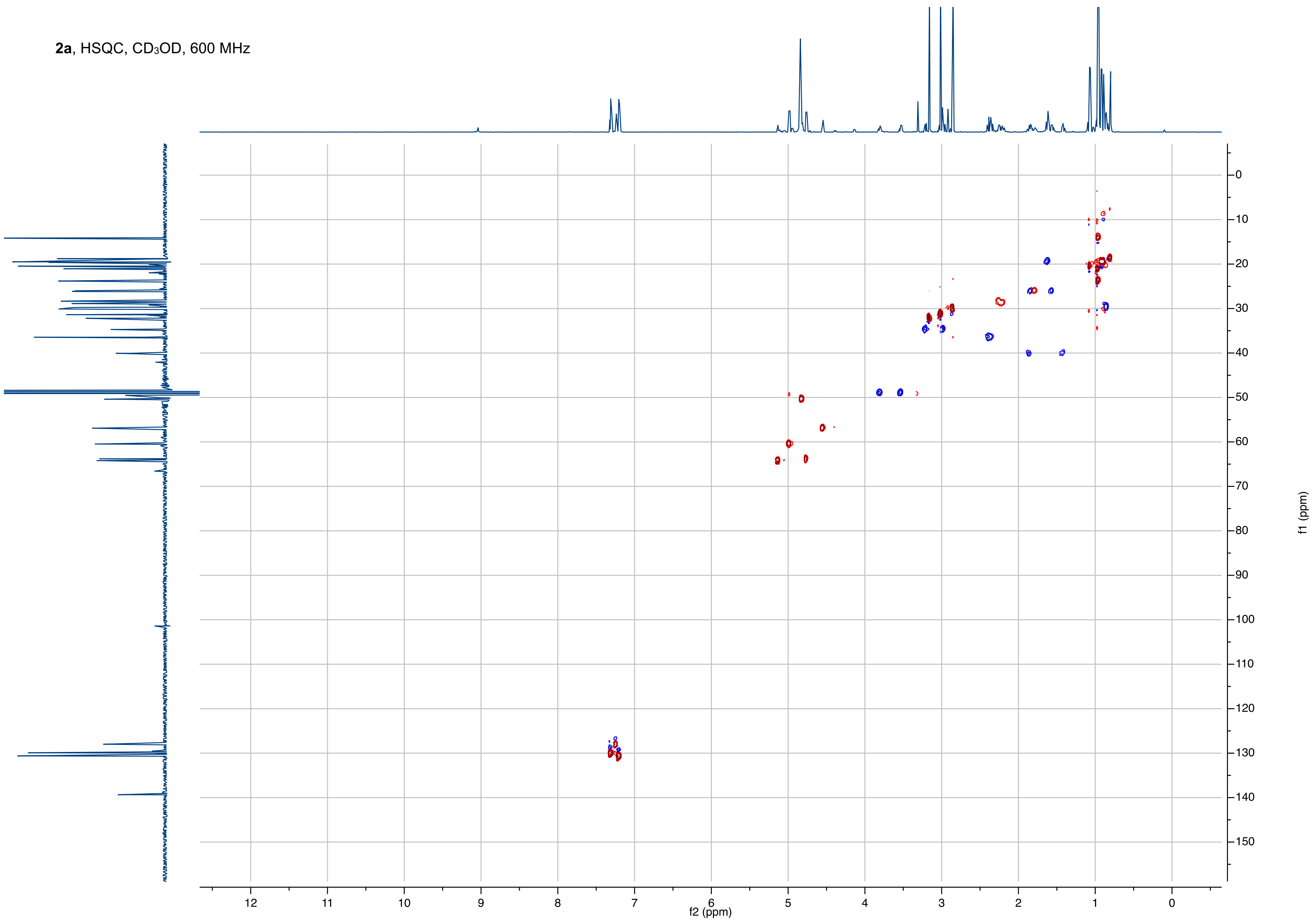

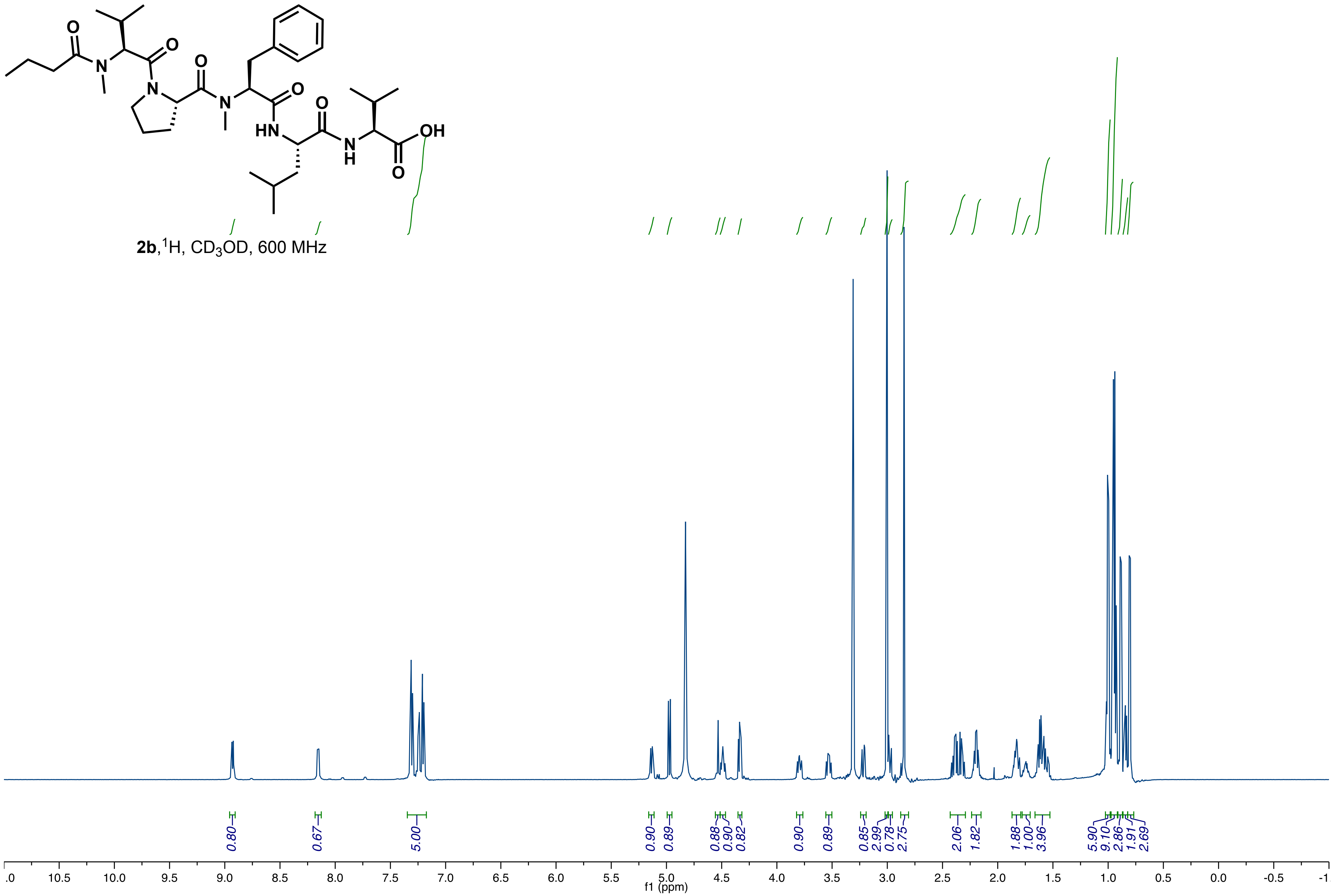

2b,^{1}H, CD_3OD, 600 MHz

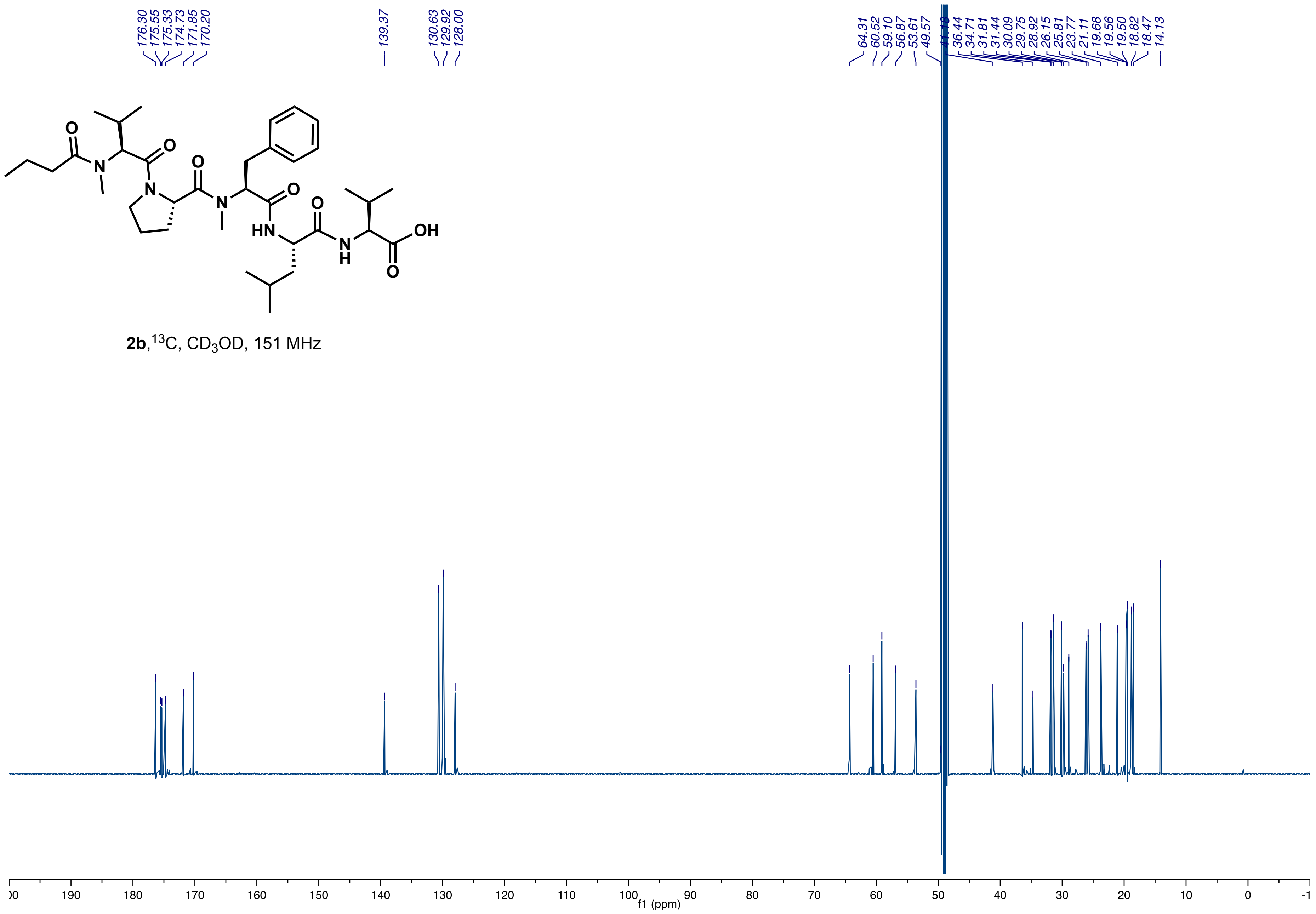

176.30
175.55
175.33
174.73
171.85
170.20
139.37
130.63
129.92
128.00
64.31
60.52
59.10
56.87
53.61
49.57
41.18
36.44
34.71
31.81
31.44
30.09
29.75
28.92
26.15
25.81
23.77
21.11
19.68
19.56
19.50
18.82
18.47
14.13
2b, 13C, CD3OD, 151 MHz
f1 (ppm)

2b, COSY, CD_3OD, 600 MHz

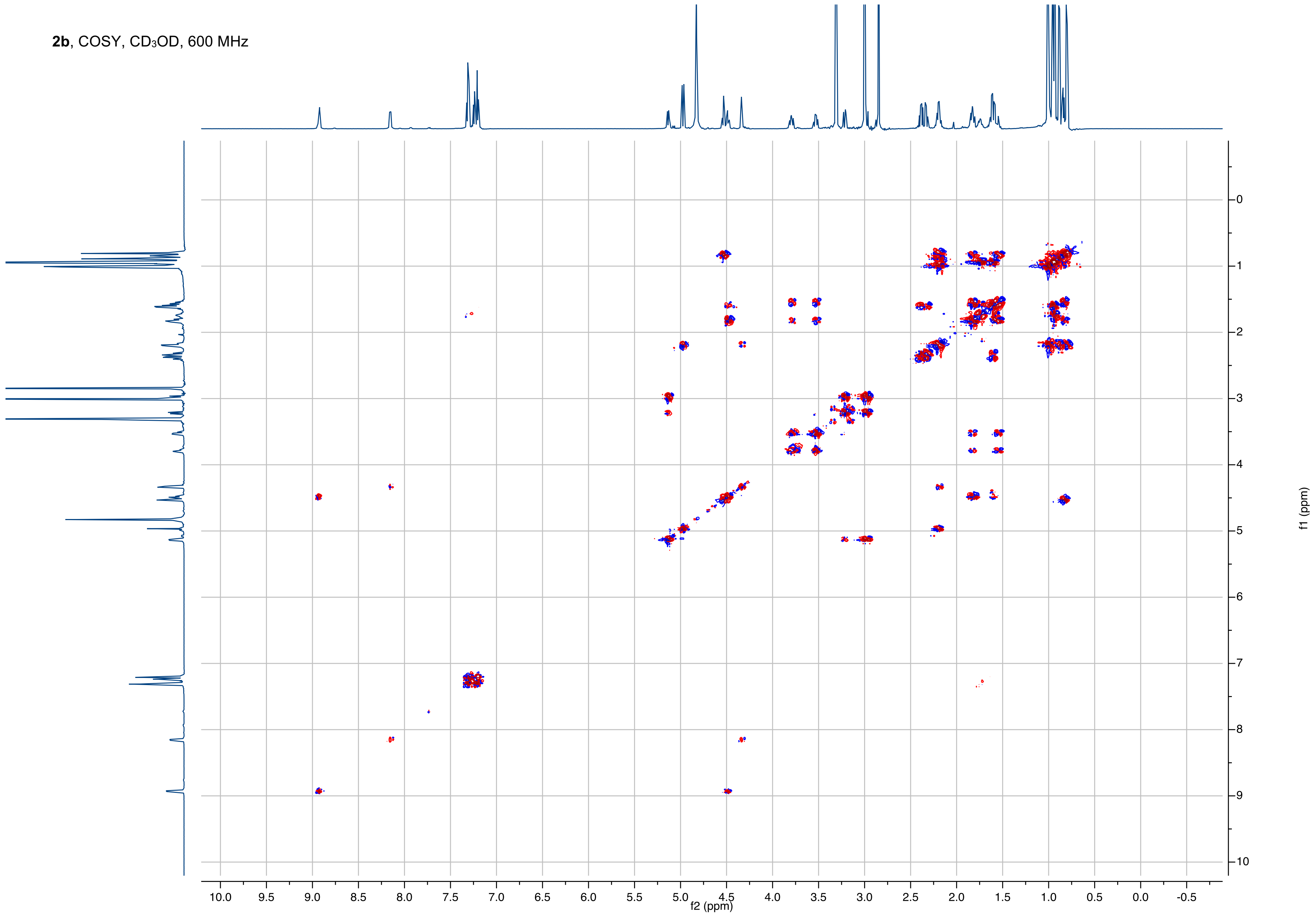

2b, HSQC, CD_3OD, 600 MHz

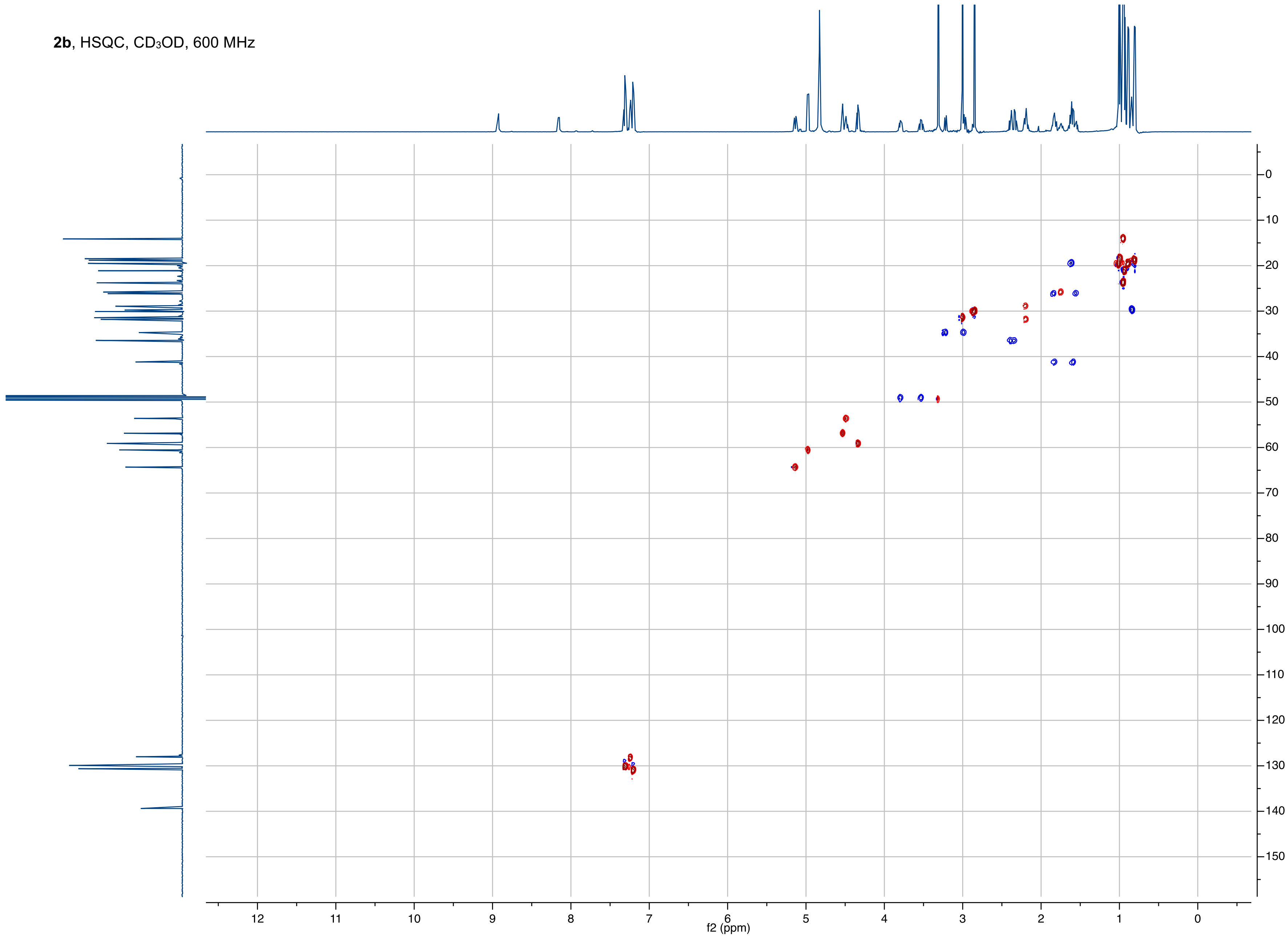

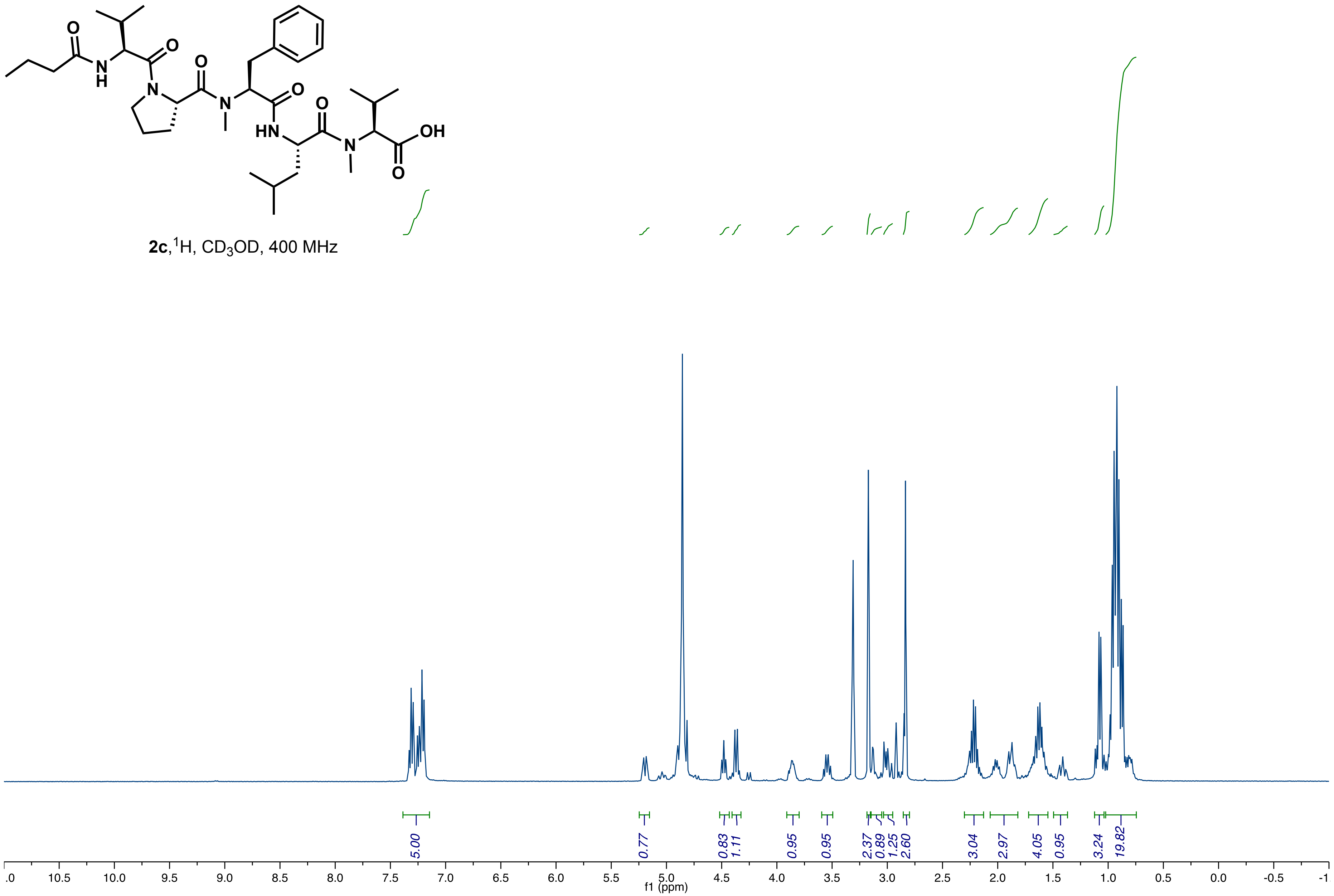

2c, ¹H, CD_3OD, 400 MHz

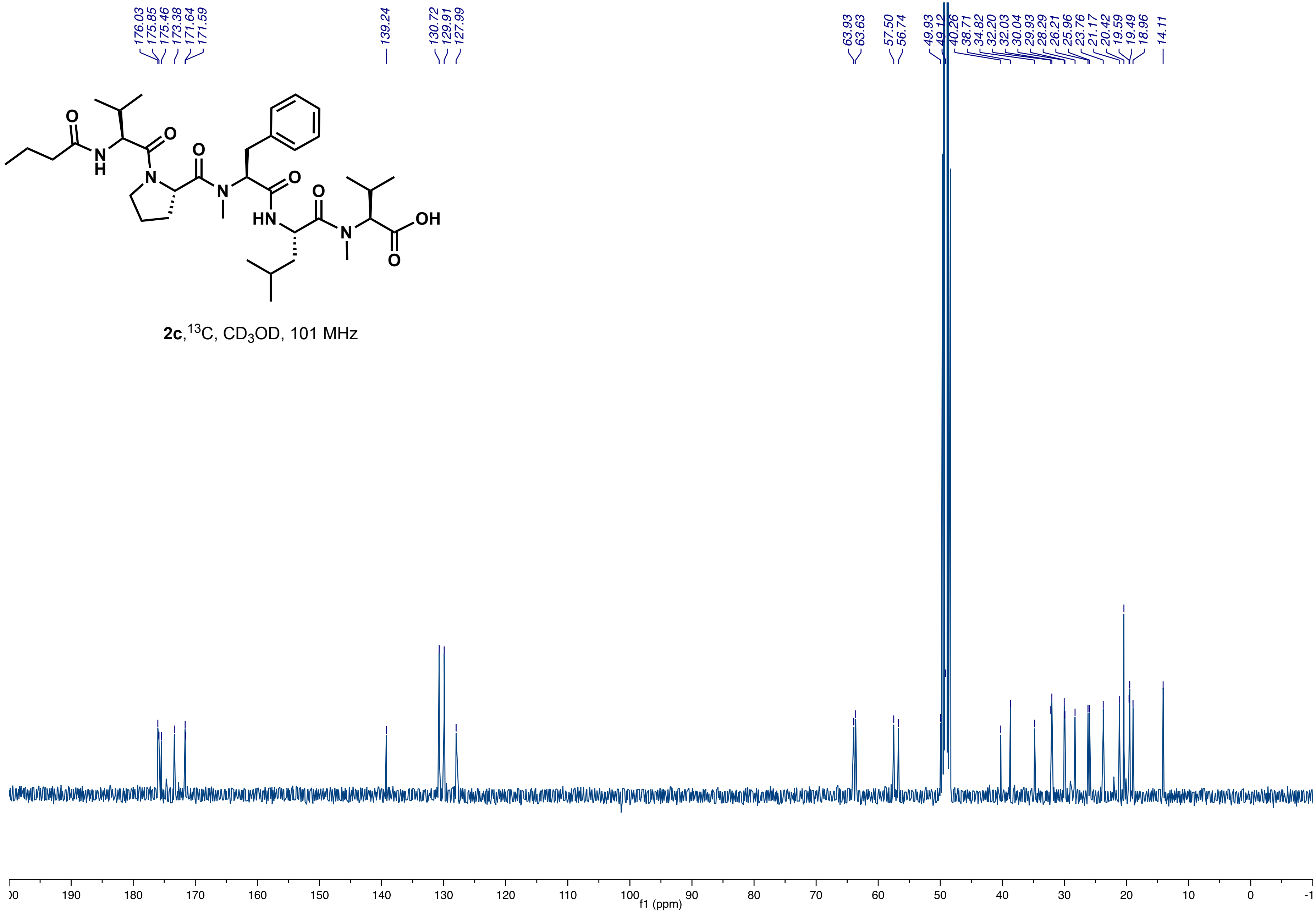

2c, ^{13}C, CD_3OD, 101 MHz

2c, COSY, CD_3OD, 400 MHz

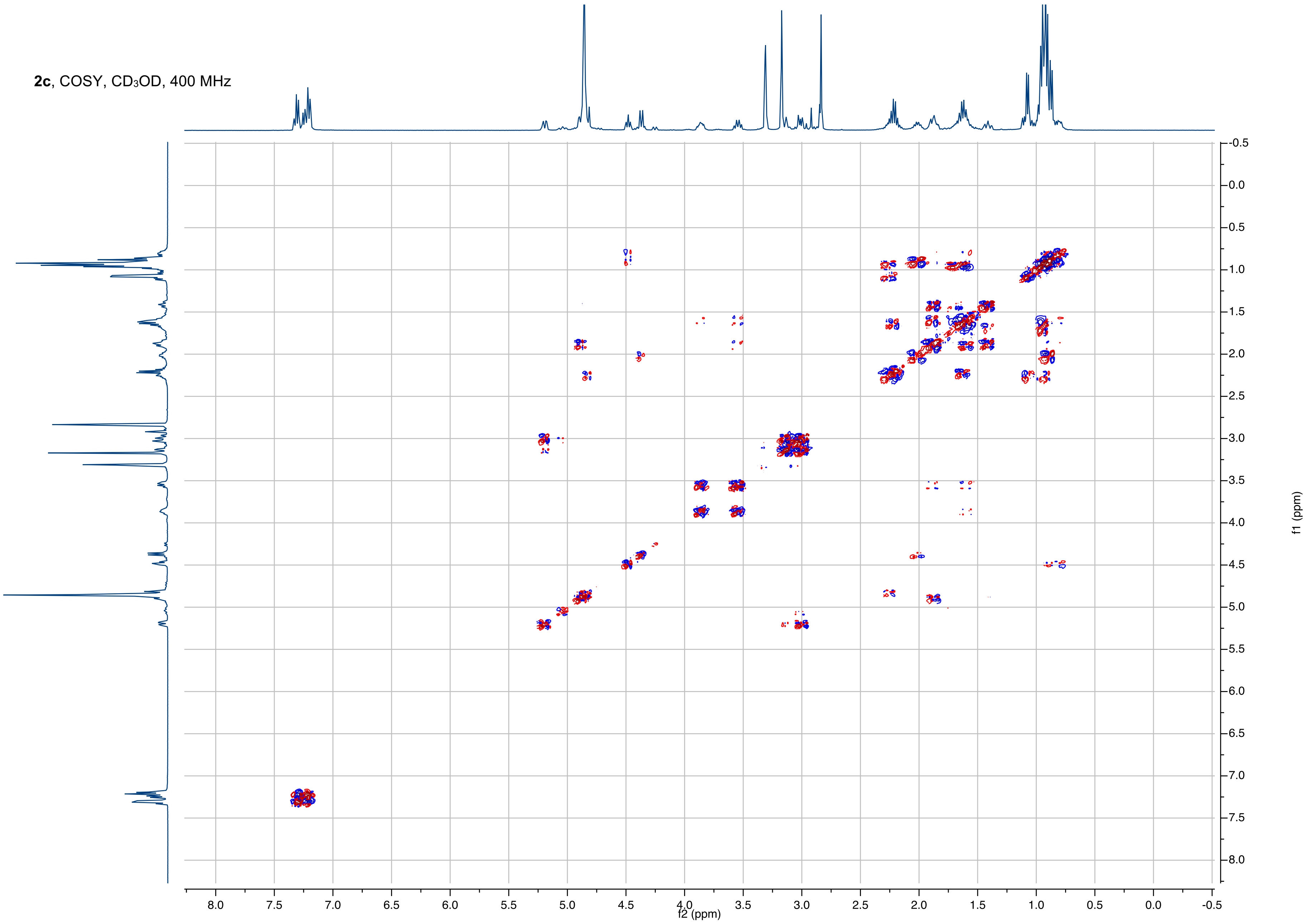

2c, HSQC, CD3OD, 400 MHz

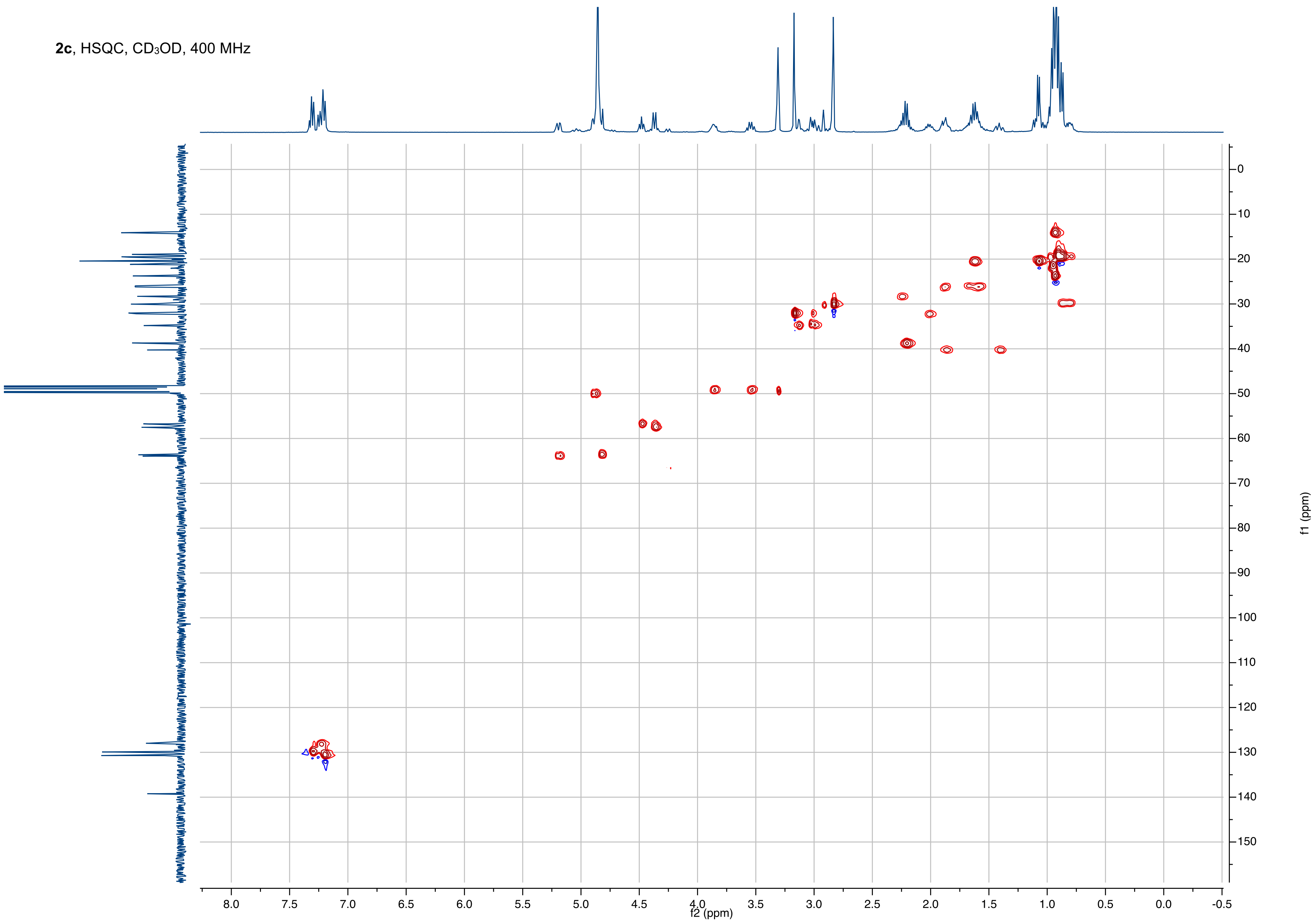

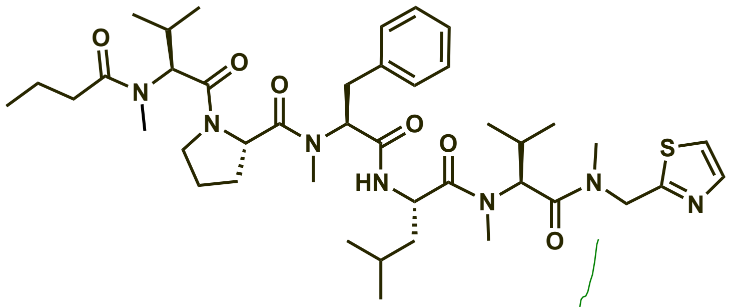

Biseokeaniamide A

1a, ^{1}H, CD_3OD, 600 MHz

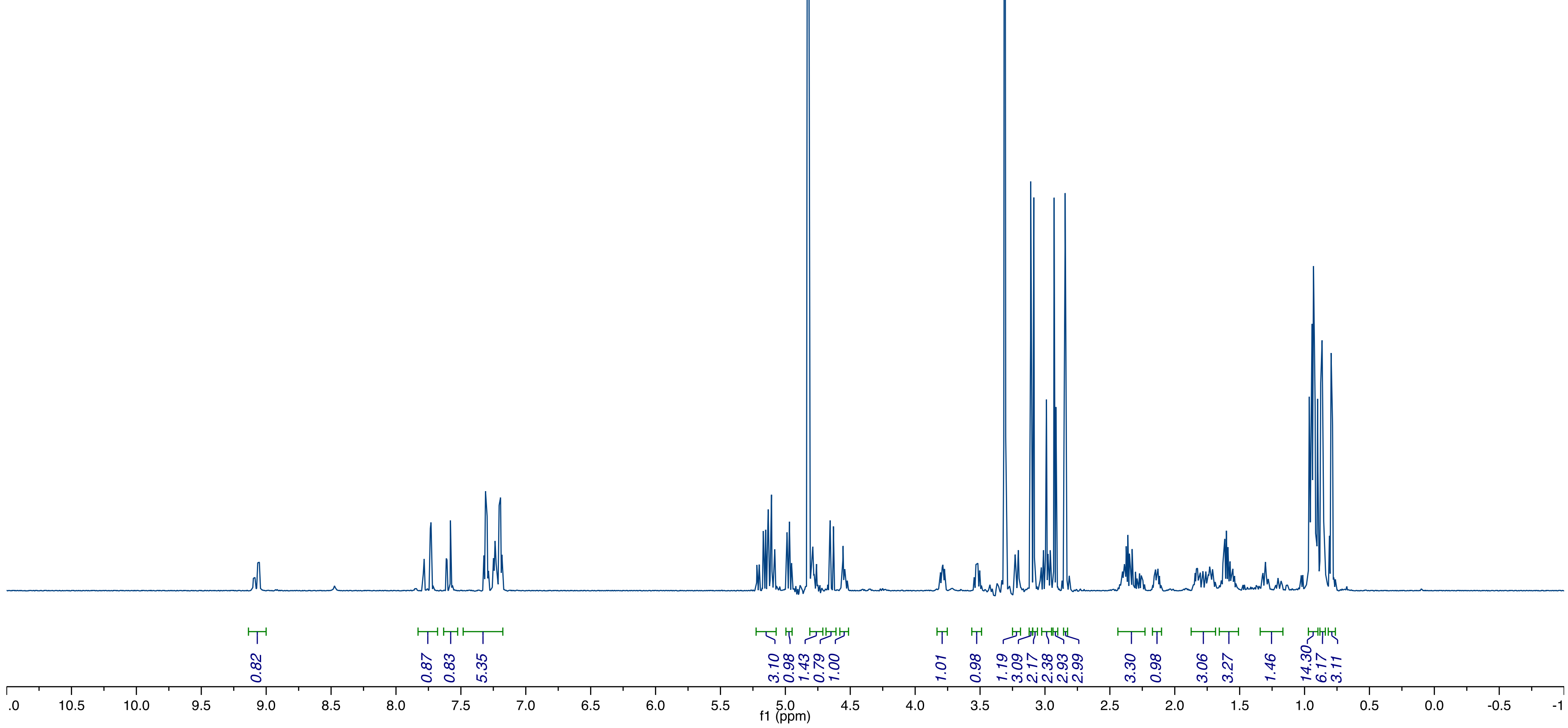

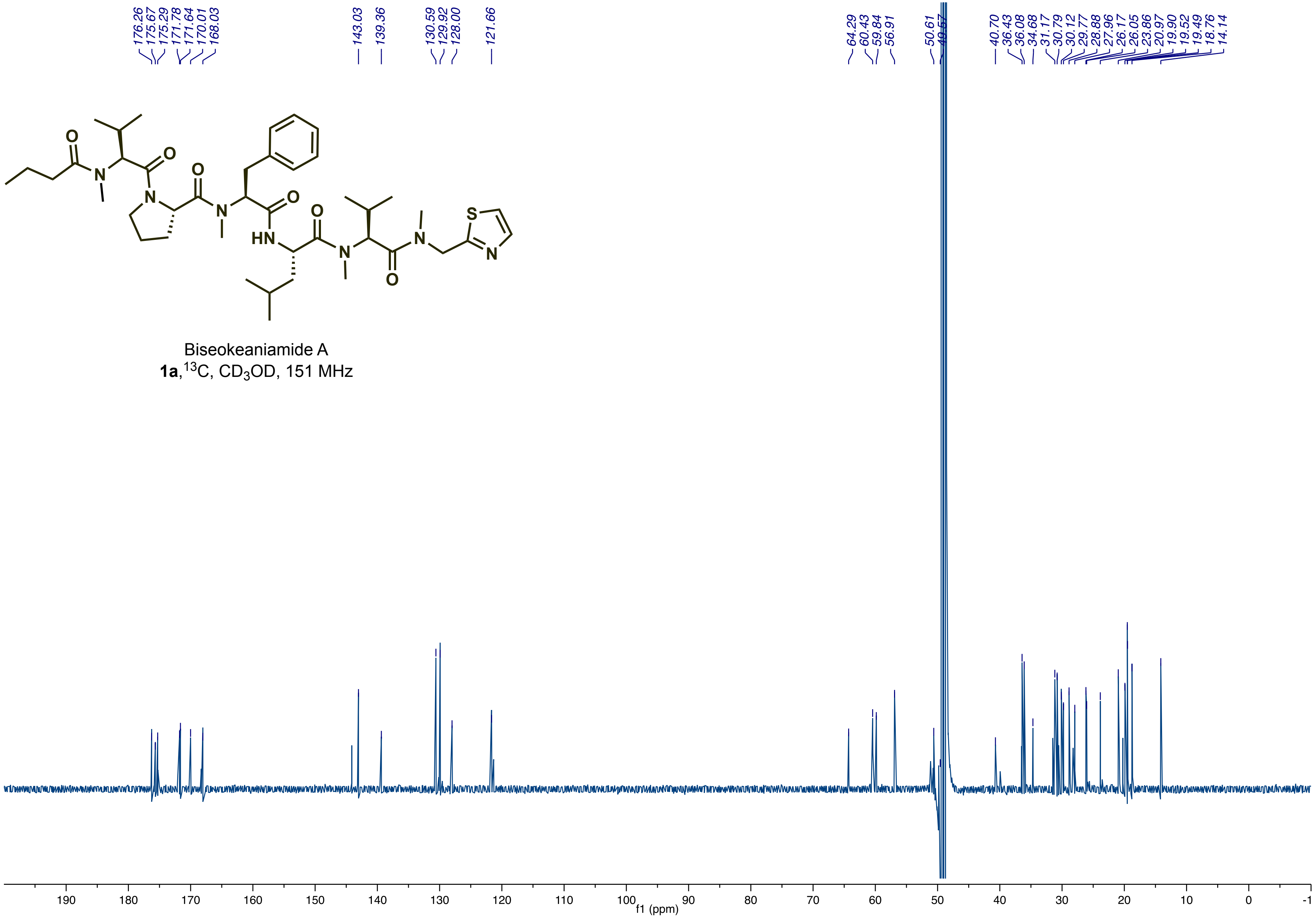

Biseokeaniamide A

1a, ^{13}C, CD_3OD, 151 MHz

1a, COSY, CD_3OD, 600 MHz

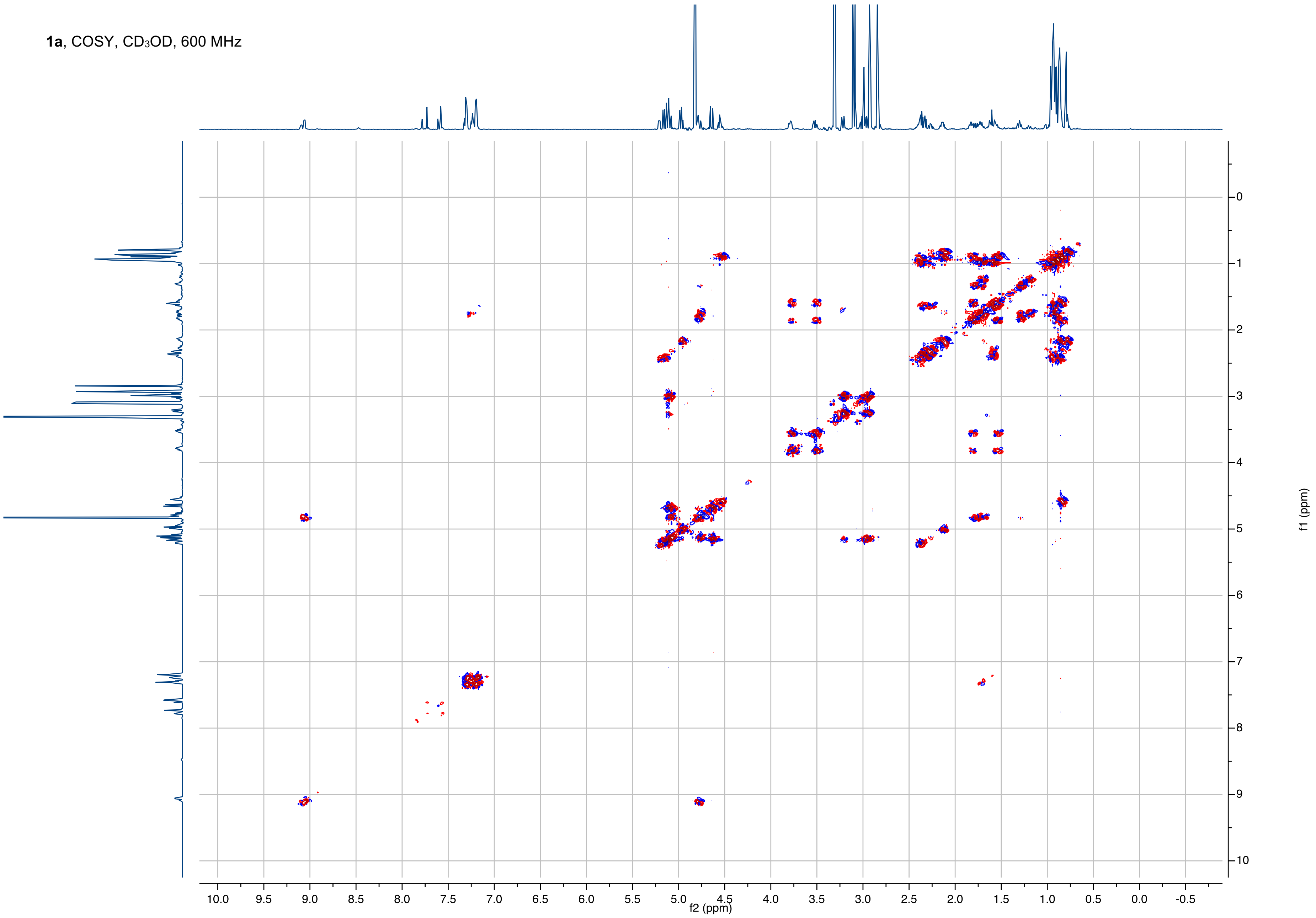

1a, HSQC, CD_3OD, 600 MHz

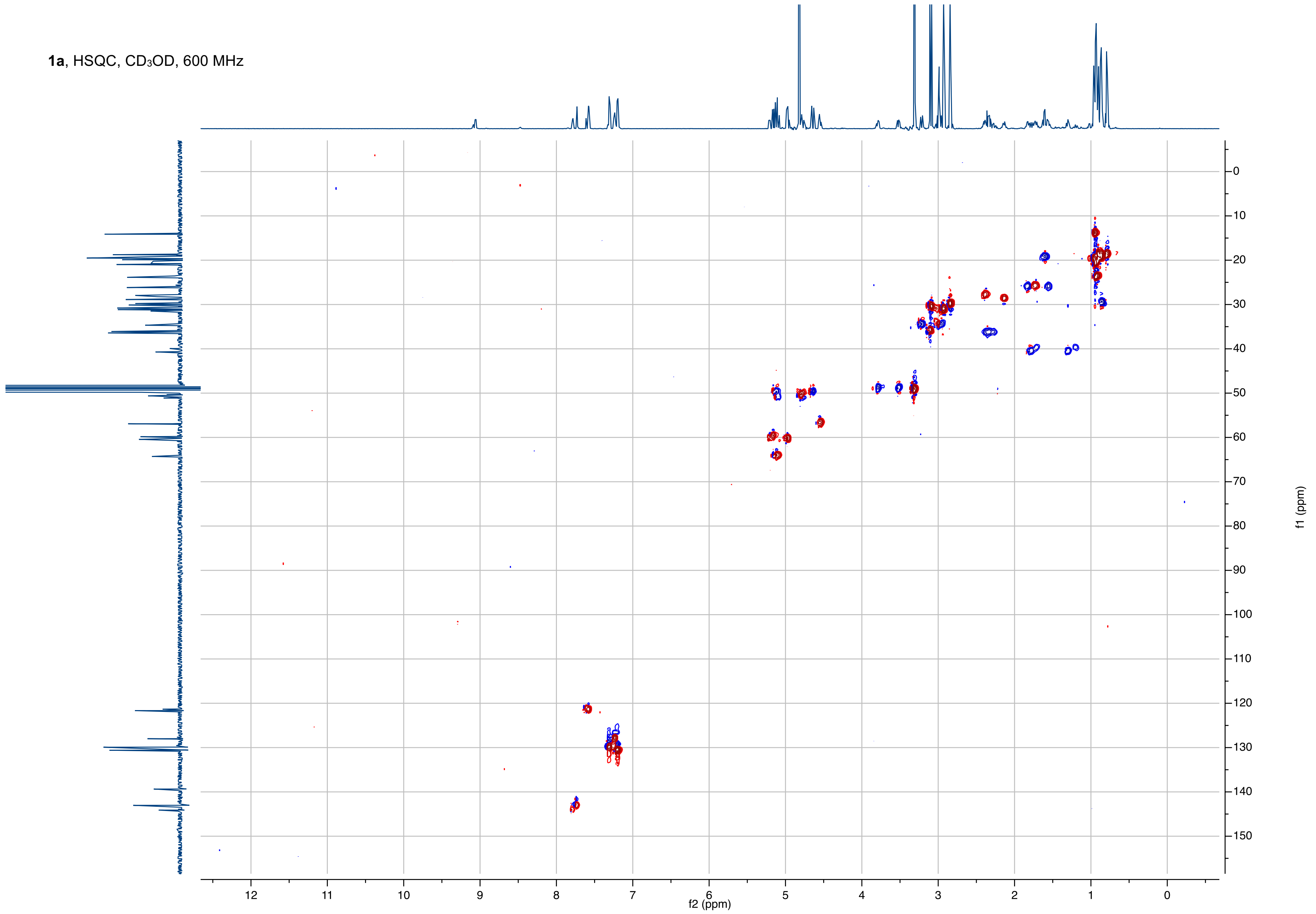

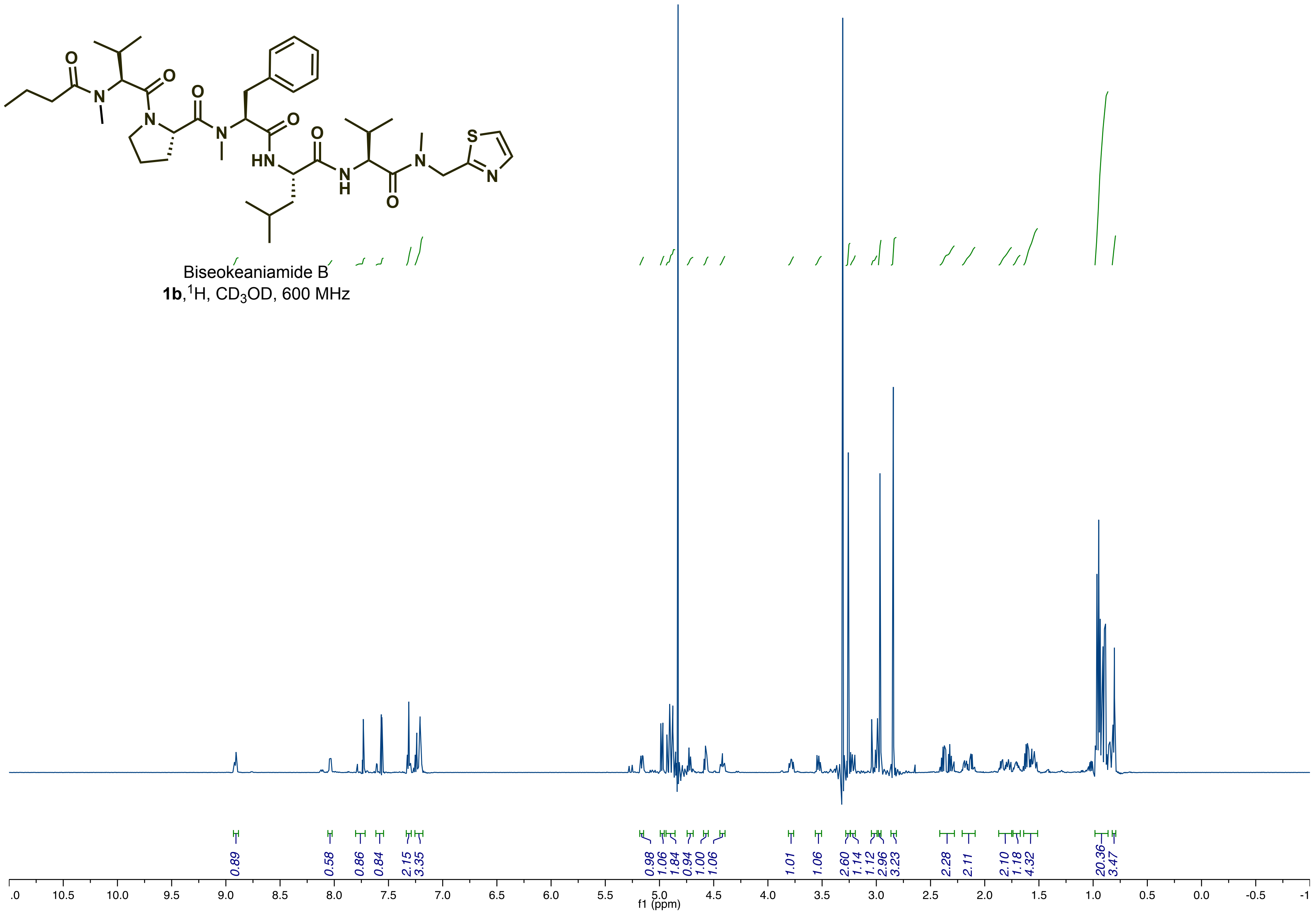

Biseokeaniamide B

1b, ^{1}H, CD_3OD, 600 MHz

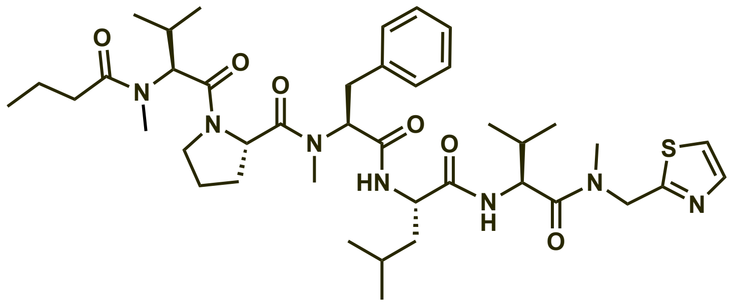

Biseokeaniamide B
1b, ^{13}C, CD_3OD, 151 MHz

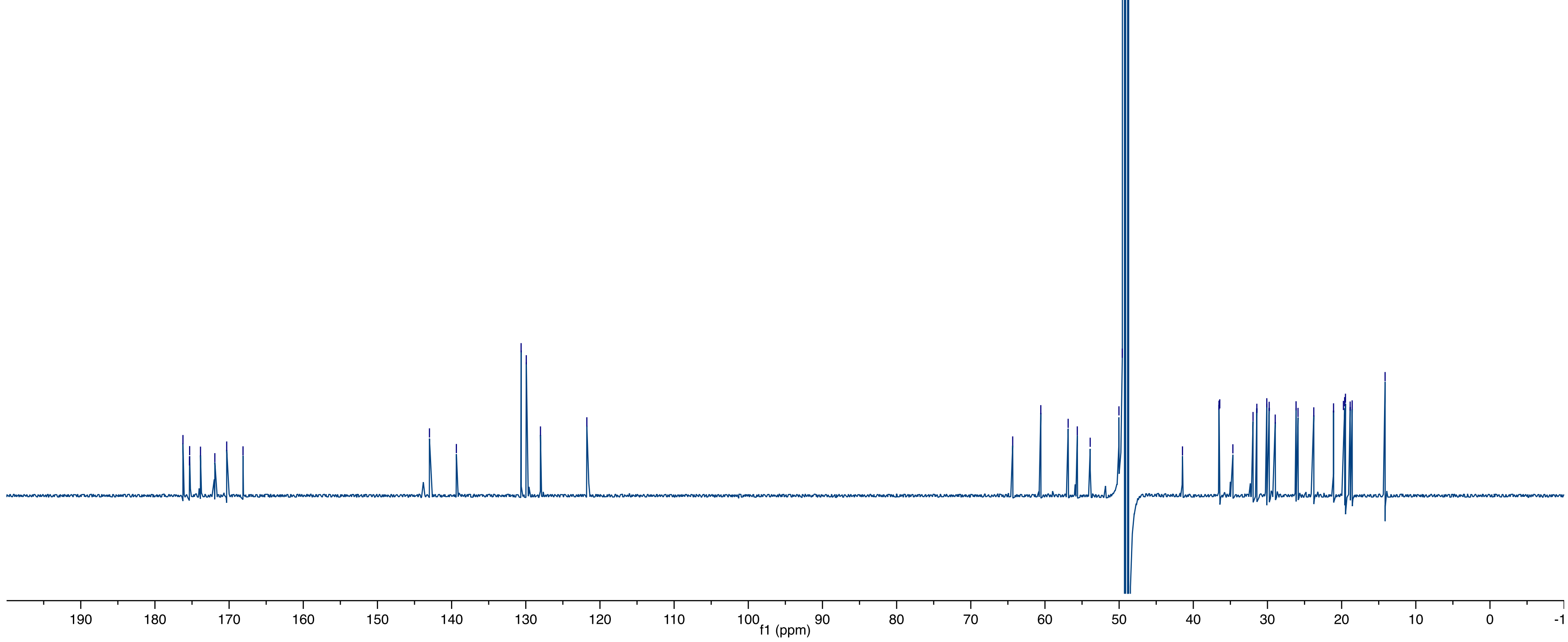

1b, COSY, CD_3OD, 600 MHz

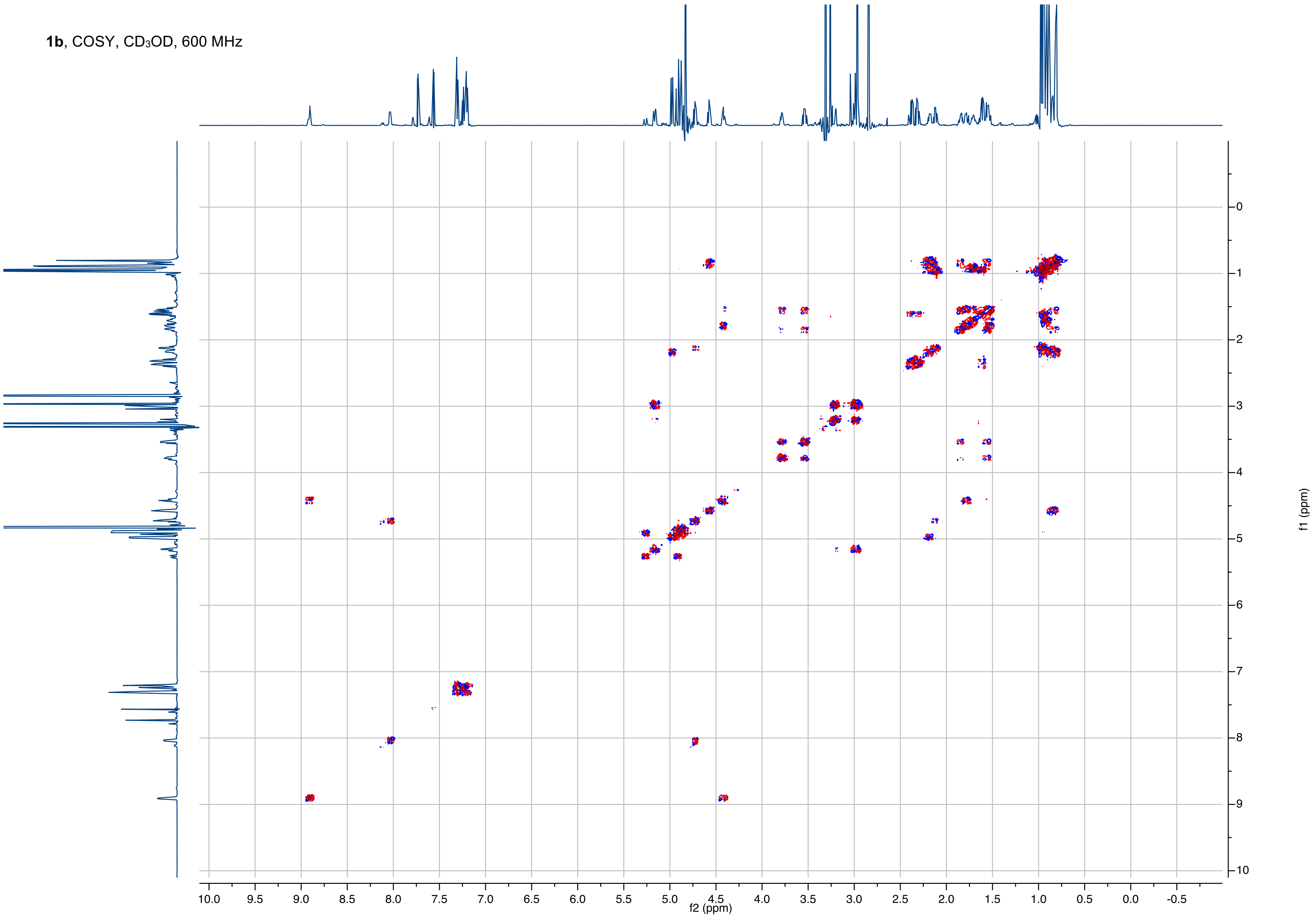

1b, HSQC, CD_3OD, 600 MHz

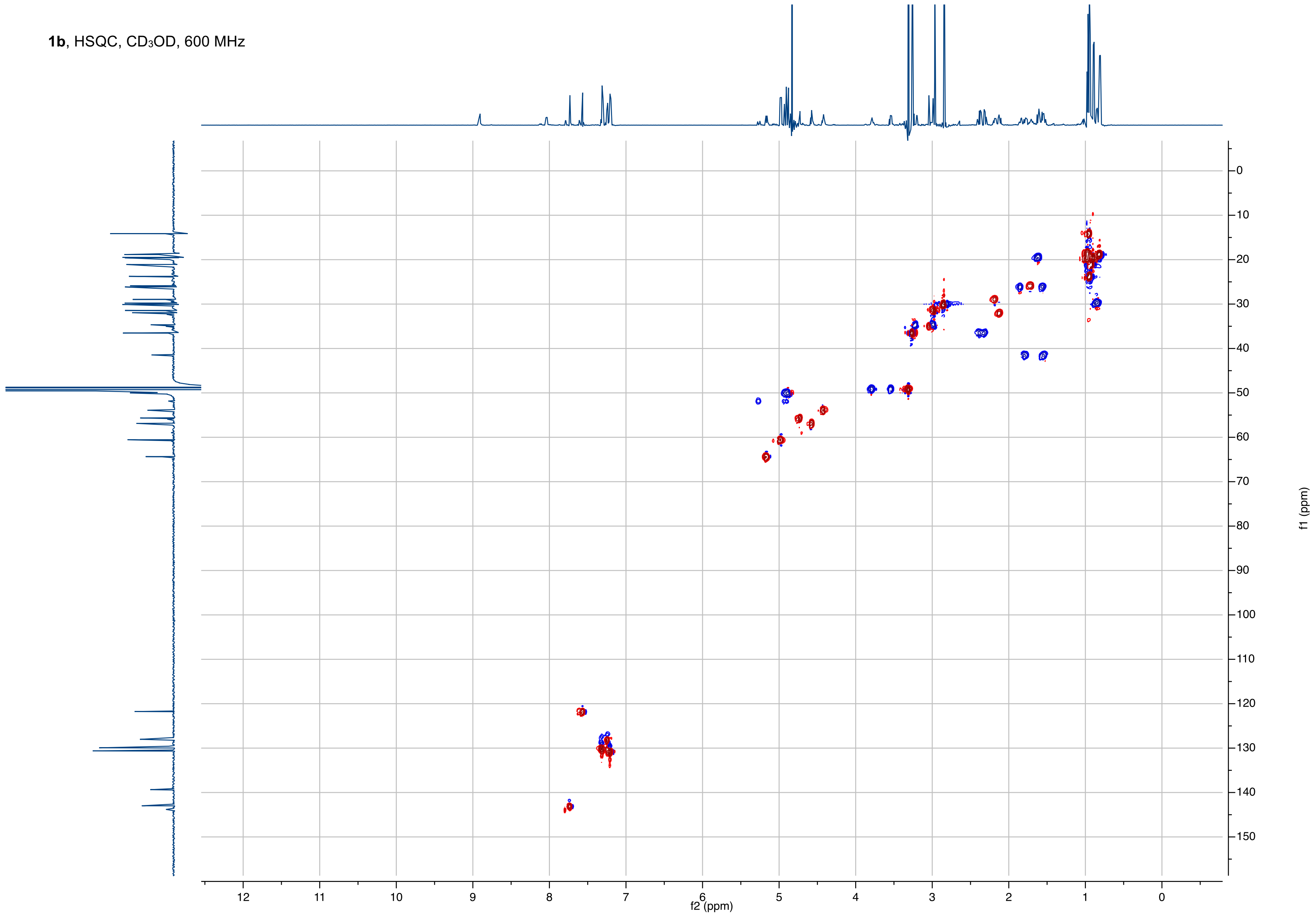

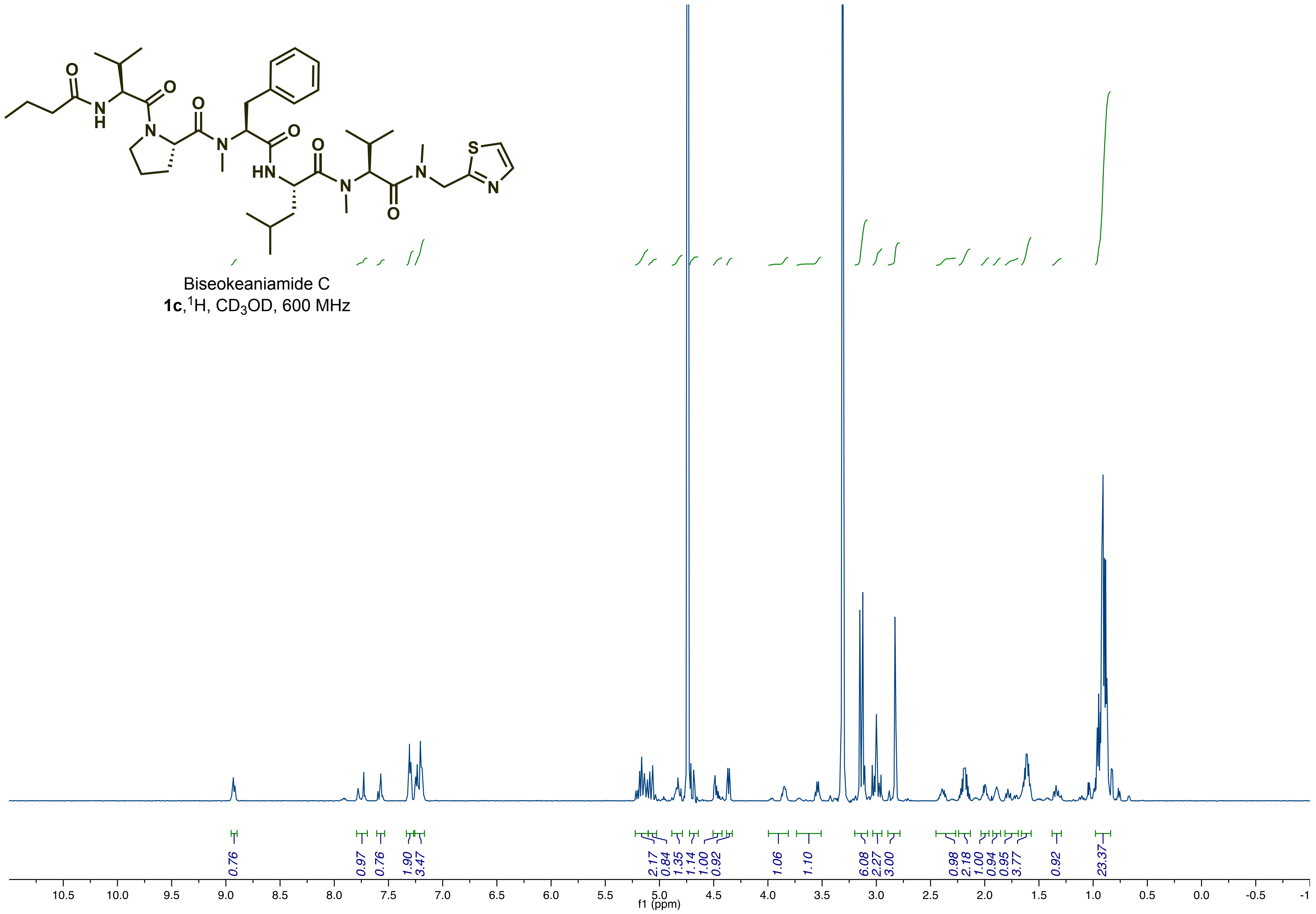

Biseokeaniamide C
1c, 1H, CD3OD, 600 MHz
0.76
0.97
0.76
1.90
3.47
2.17
0.84
1.35
1.14
1.00
0.92
1.06
1.10
6.08
2.27
3.00
0.98
2.18
1.00
0.94
0.95
3.77
0.92
23.37
10.5
10.0
9.5
9.0
8.5
8.0
7.5
7.0
6.5
6.0
5.5
5.0
4.5
4.0
3.5
3.0
2.5
2.0
1.5
1.0
0.5
0.0
-0.5
-1
f1 (ppm)

^{1}H NMR spectra comparison[3]:
(Biseokeaniamide C, CD_3OD)

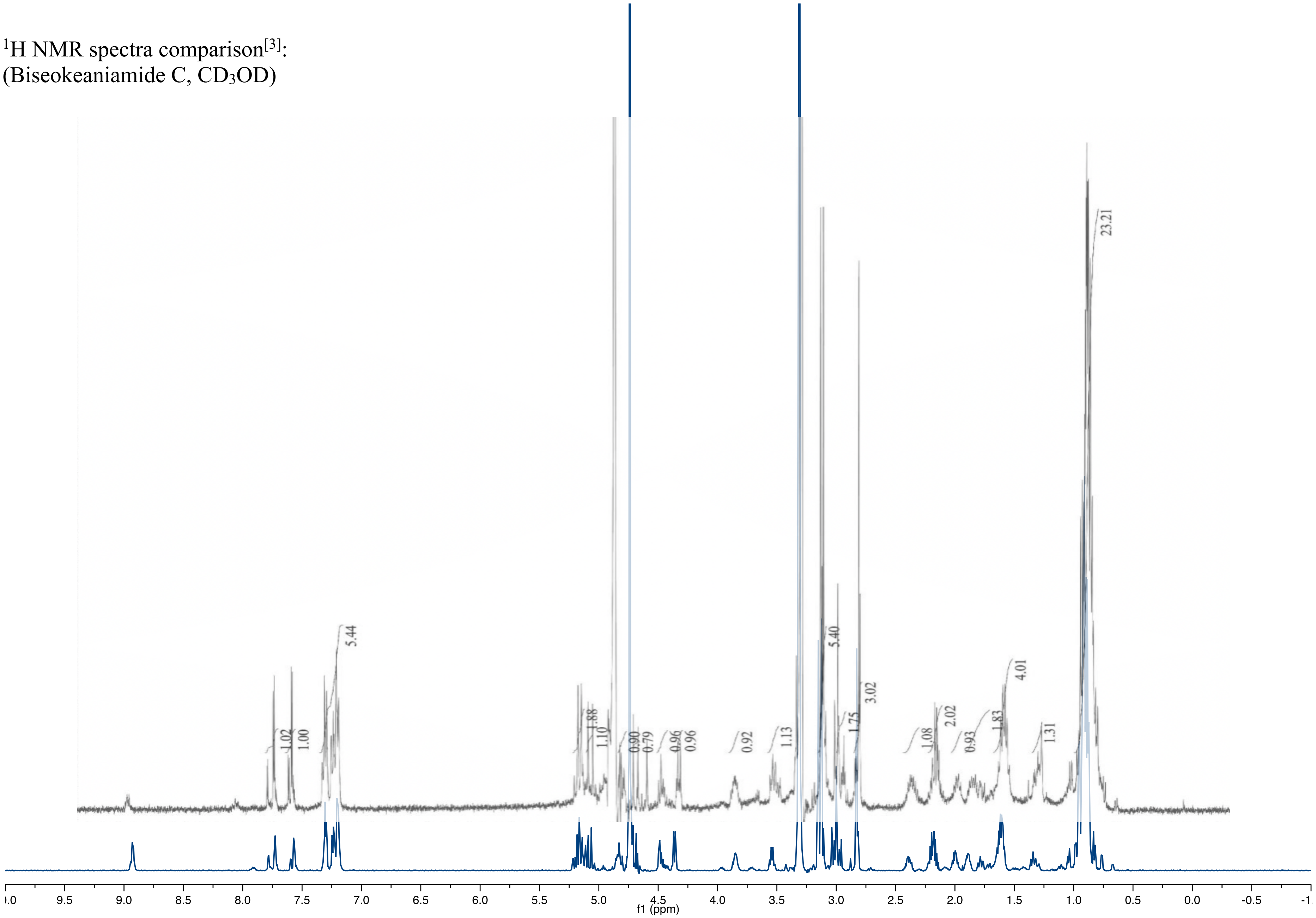

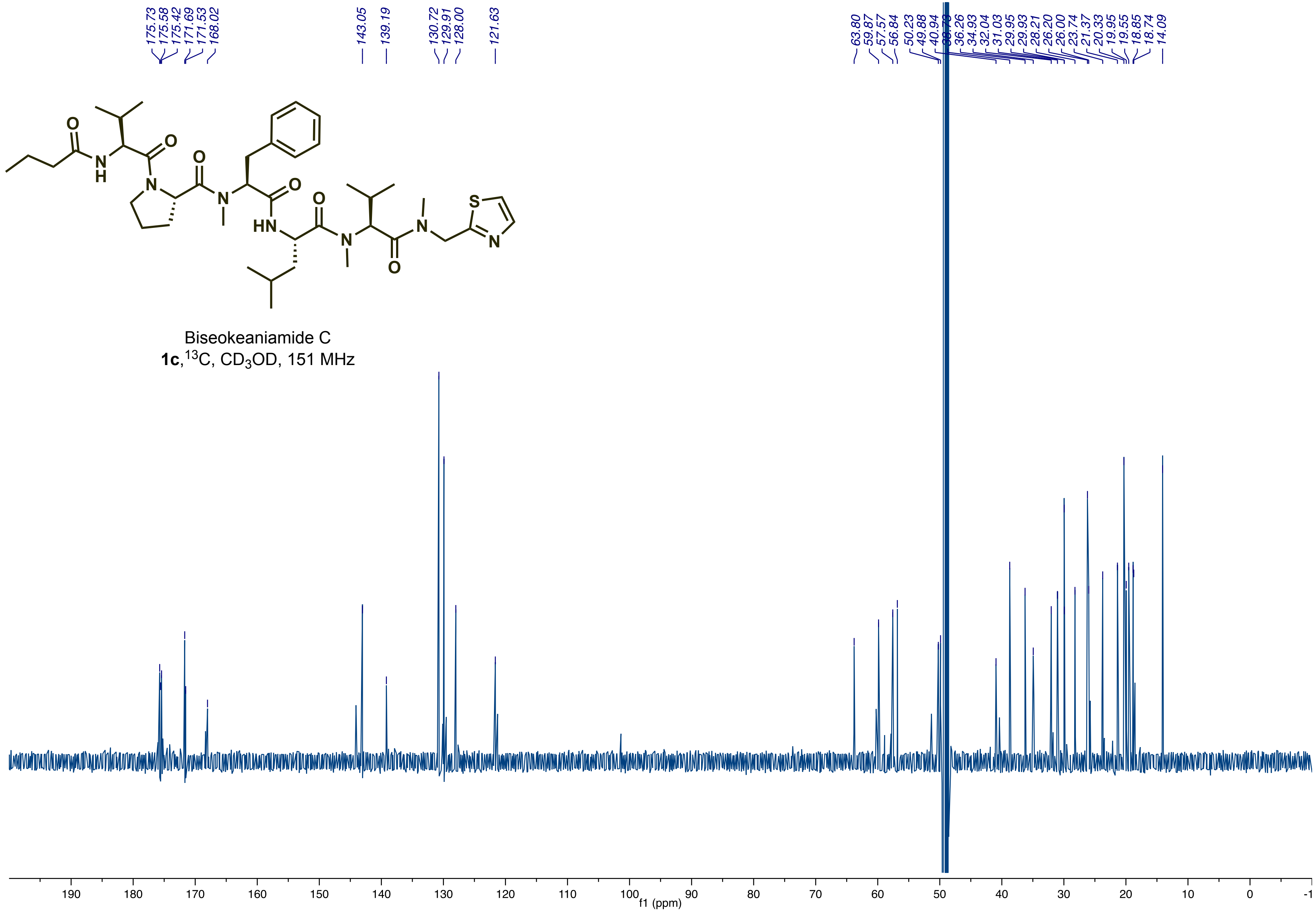

Biseokeaniamide C

1c, ^{13}C, CD_3OD, 151 MHz

1c, COSY, CD_3OD, 600 MHz

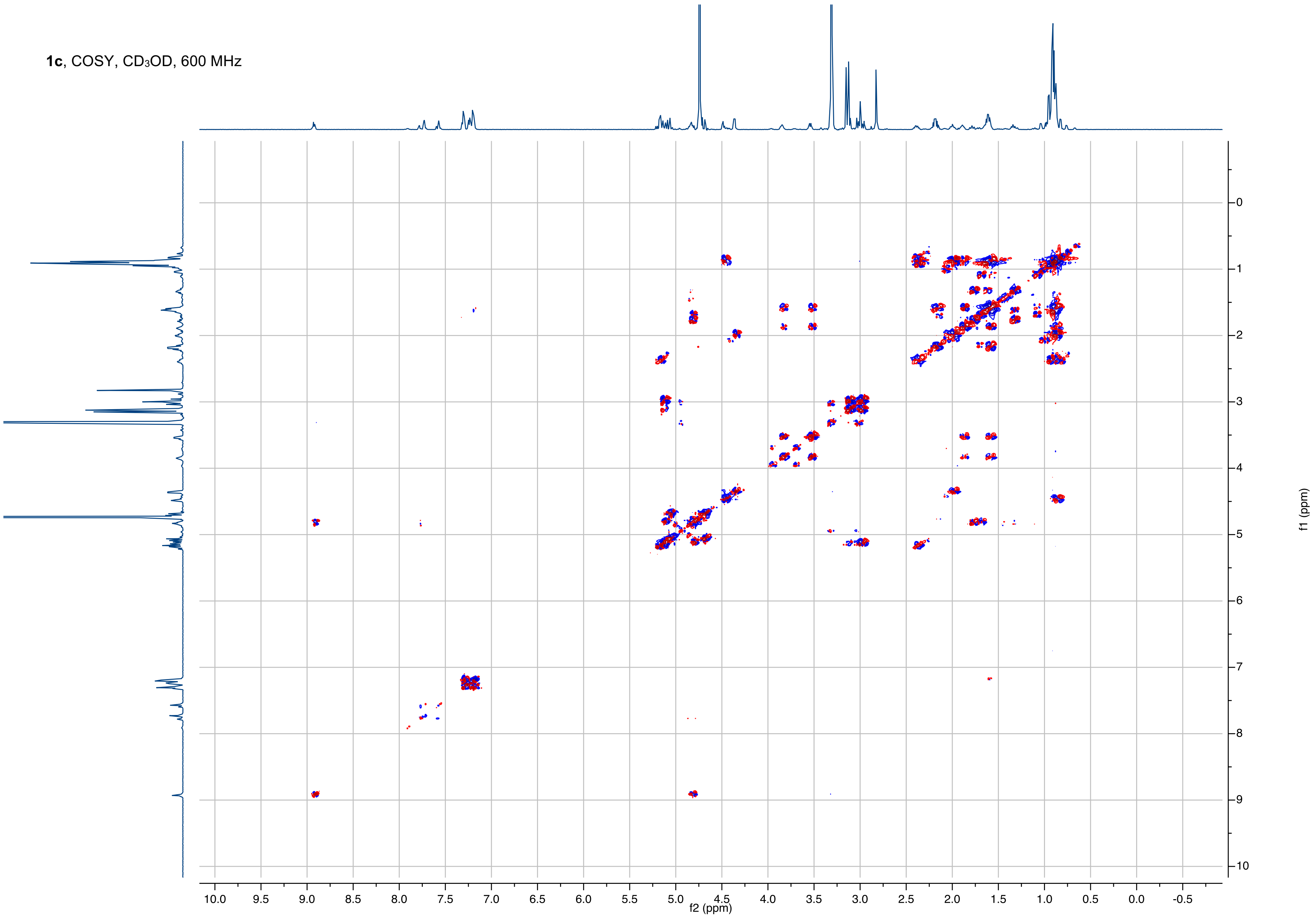

1c, HSQC, CD_3OD, 600 MHz

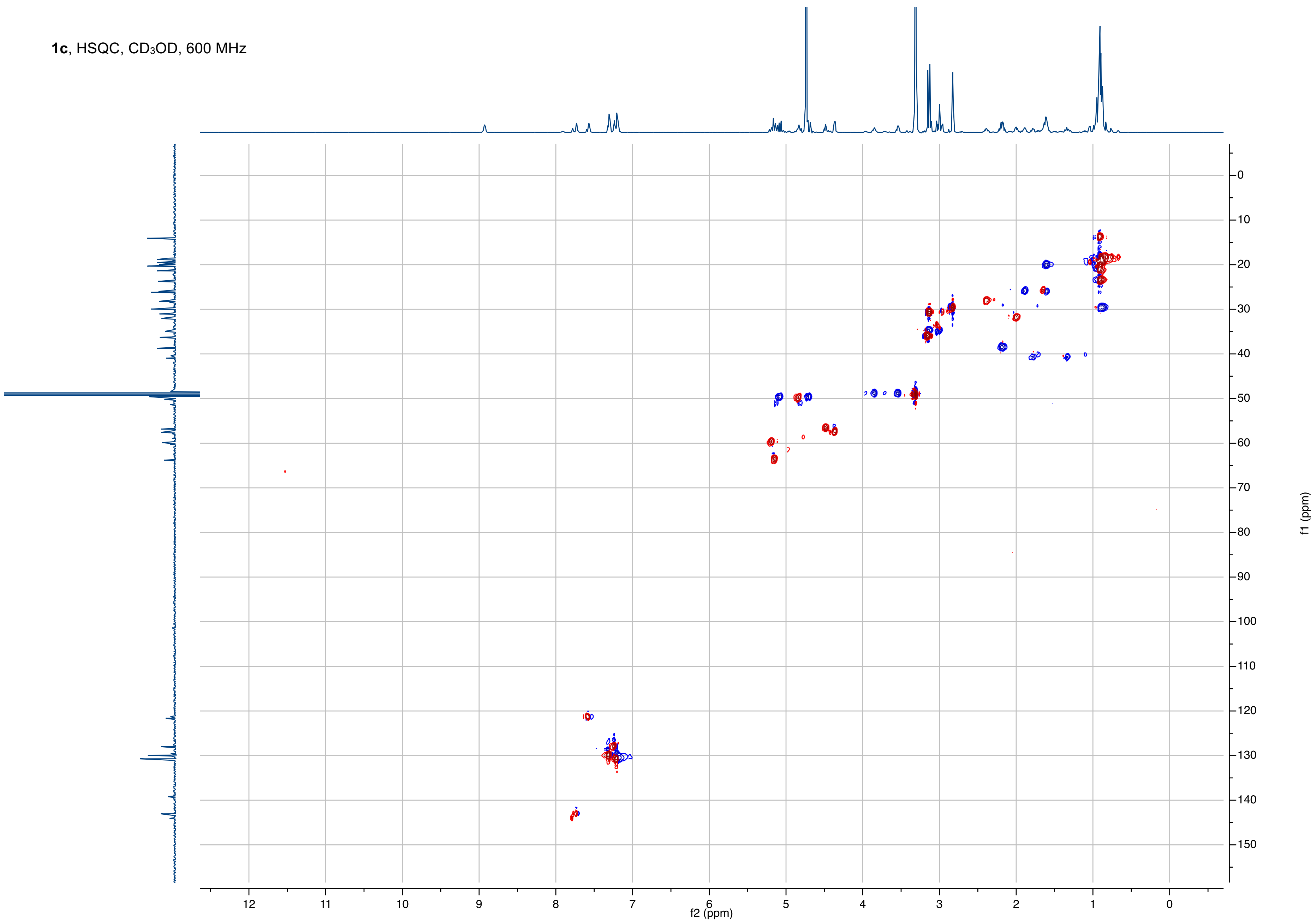

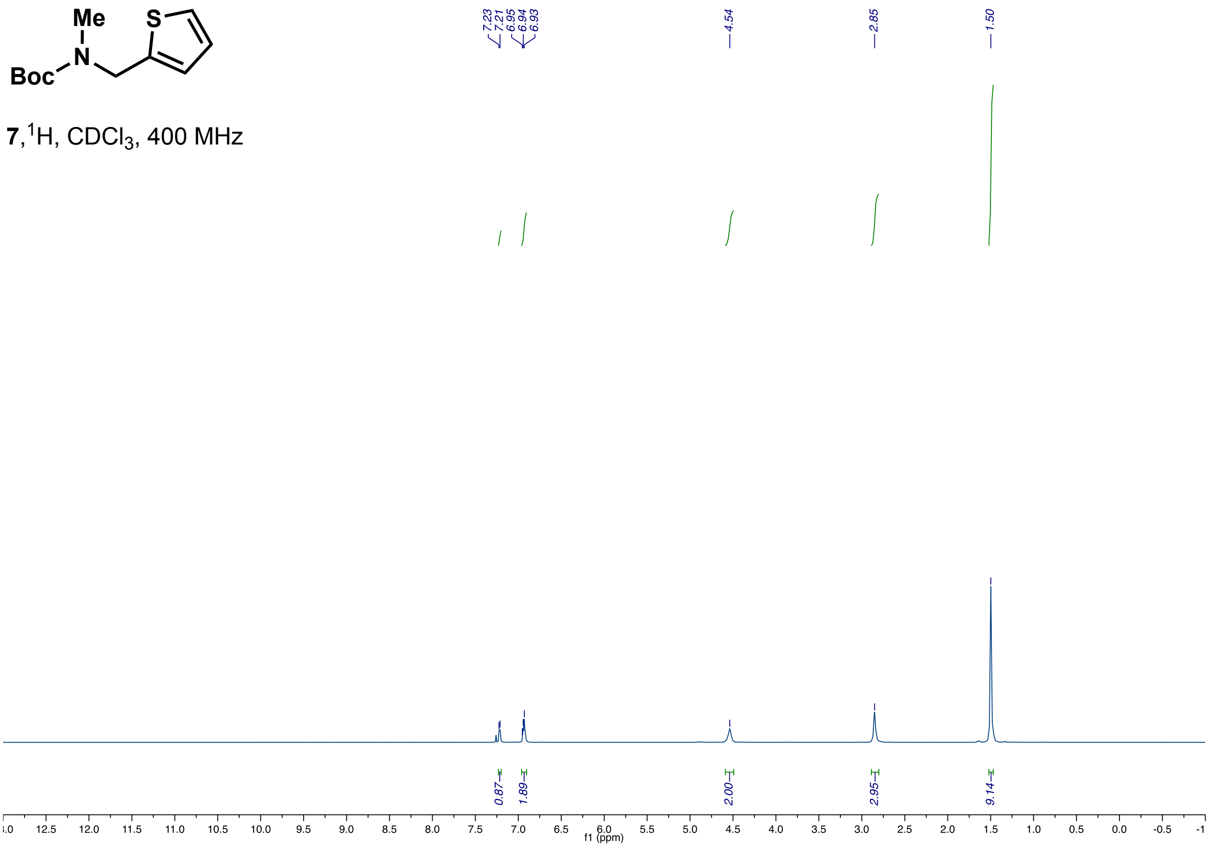

7,^{1}H, $CDCl_3$, 400 MHz

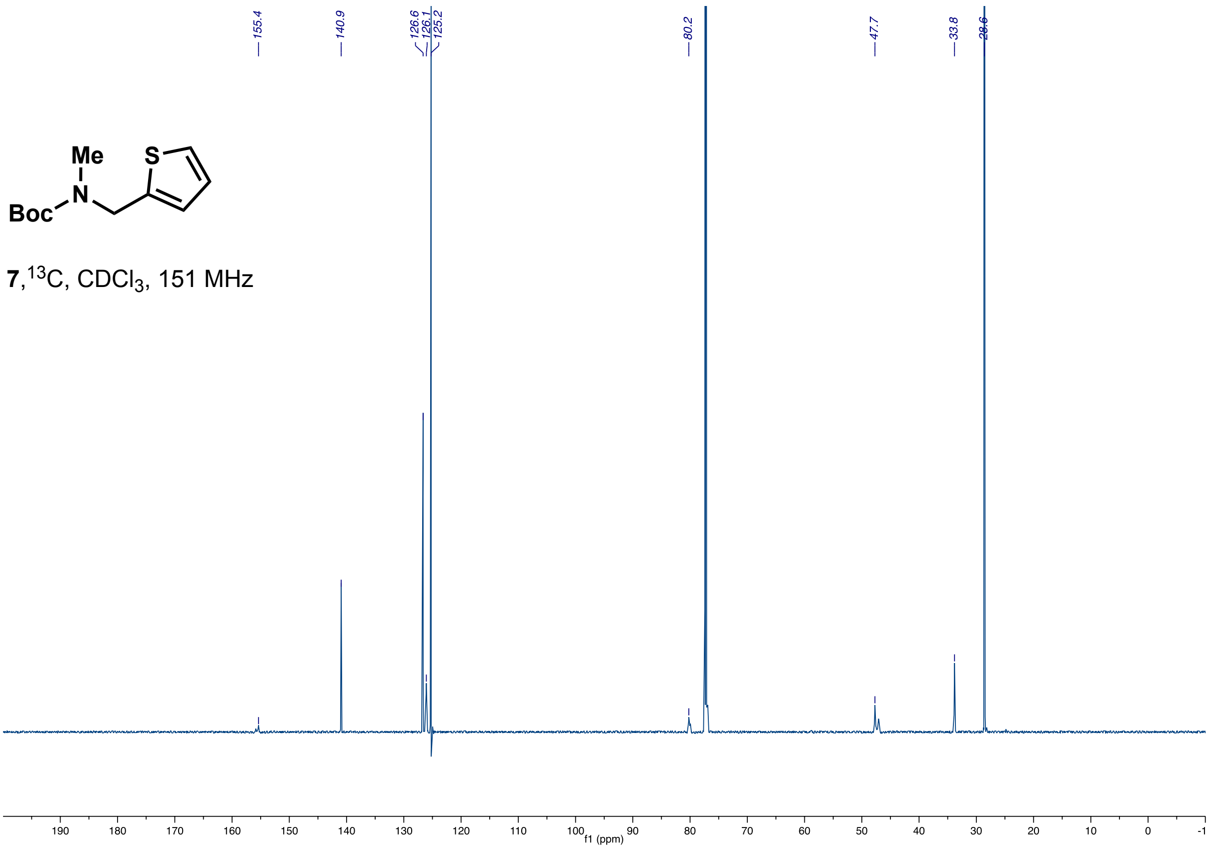

7,^{13}C, $CDCl_3$, 151 MHz

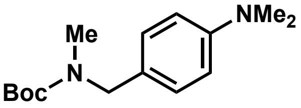

8,^{1}H, CDCl$_3$, 400 MHz

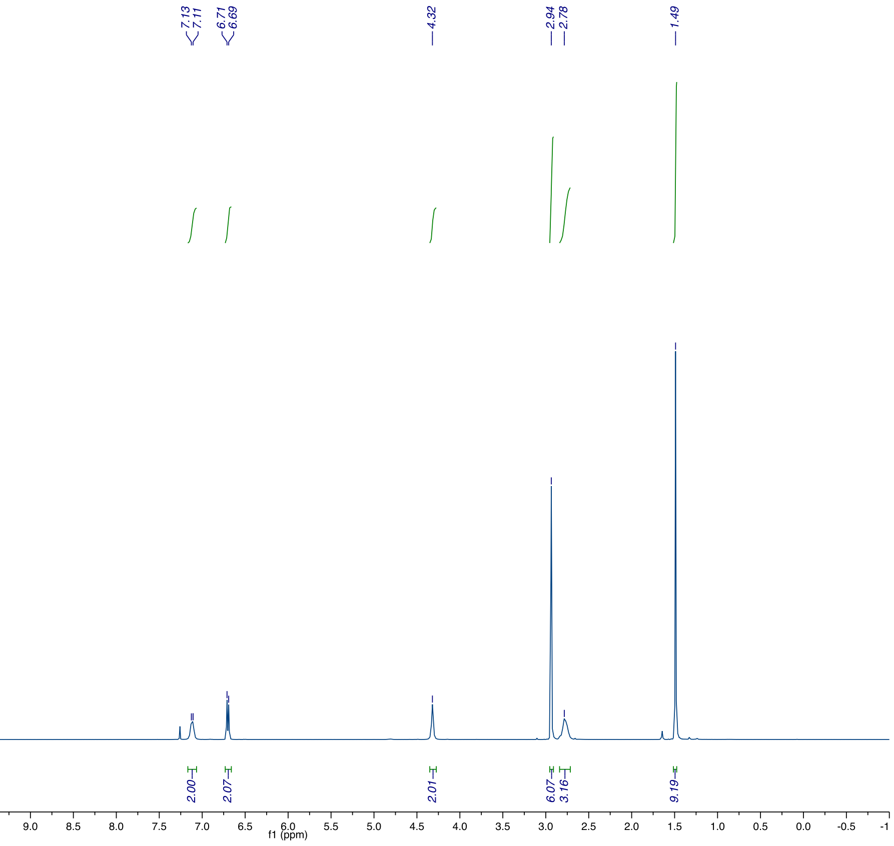

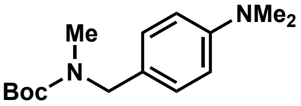

8,^{13}C, CDCl$_3$, 101 MHz

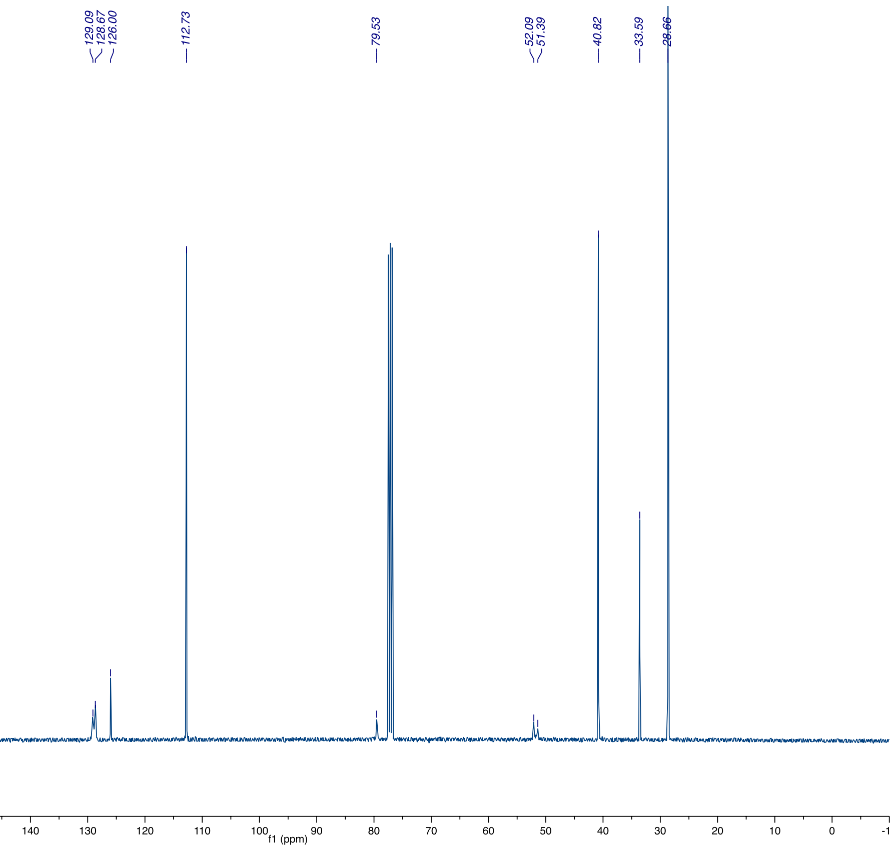

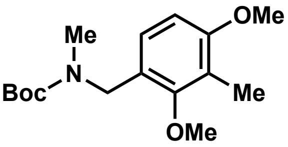

9, ^{1}H, $CDCl_3$, 400 MHz

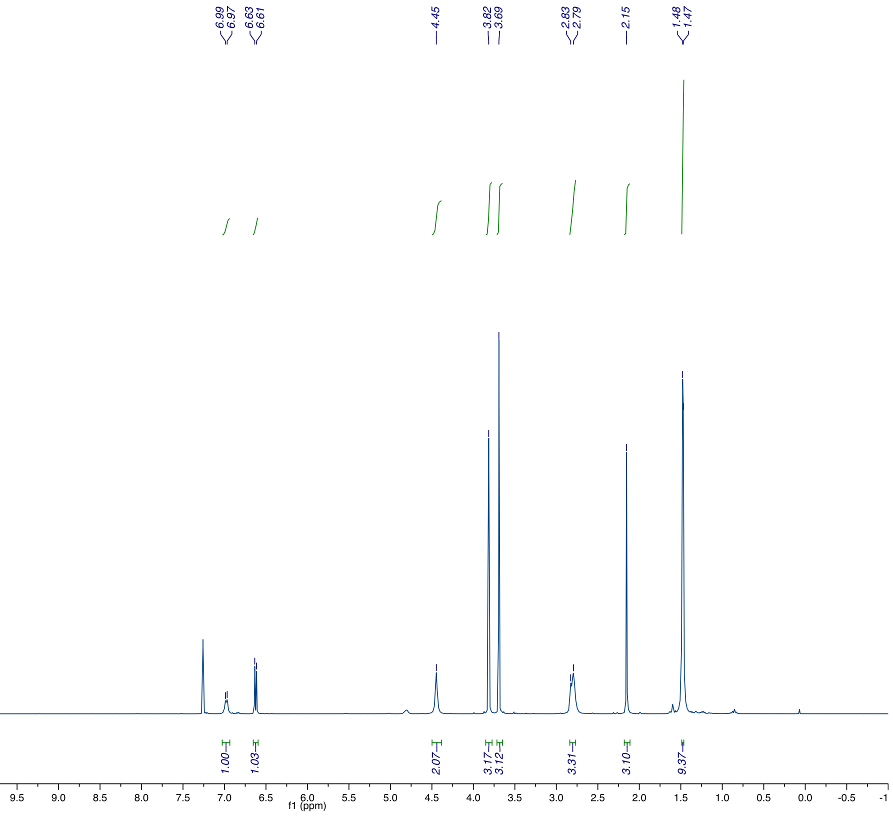

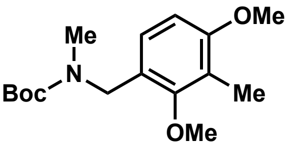

9, ^{13}C, $CDCl_3$, 101 MHz

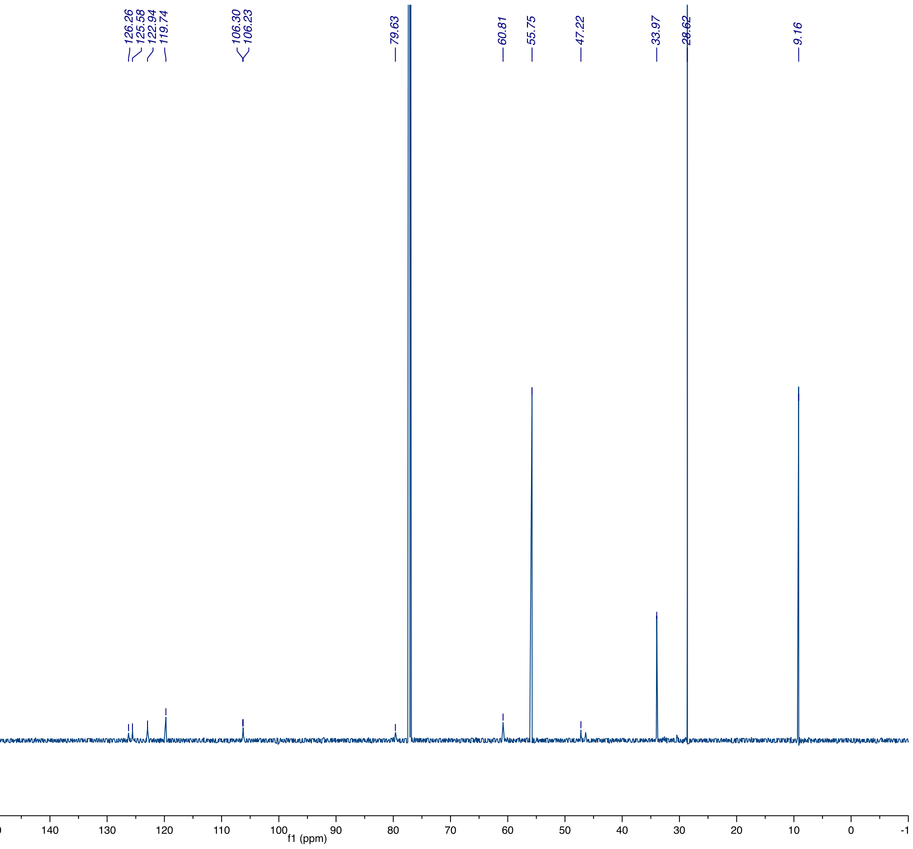

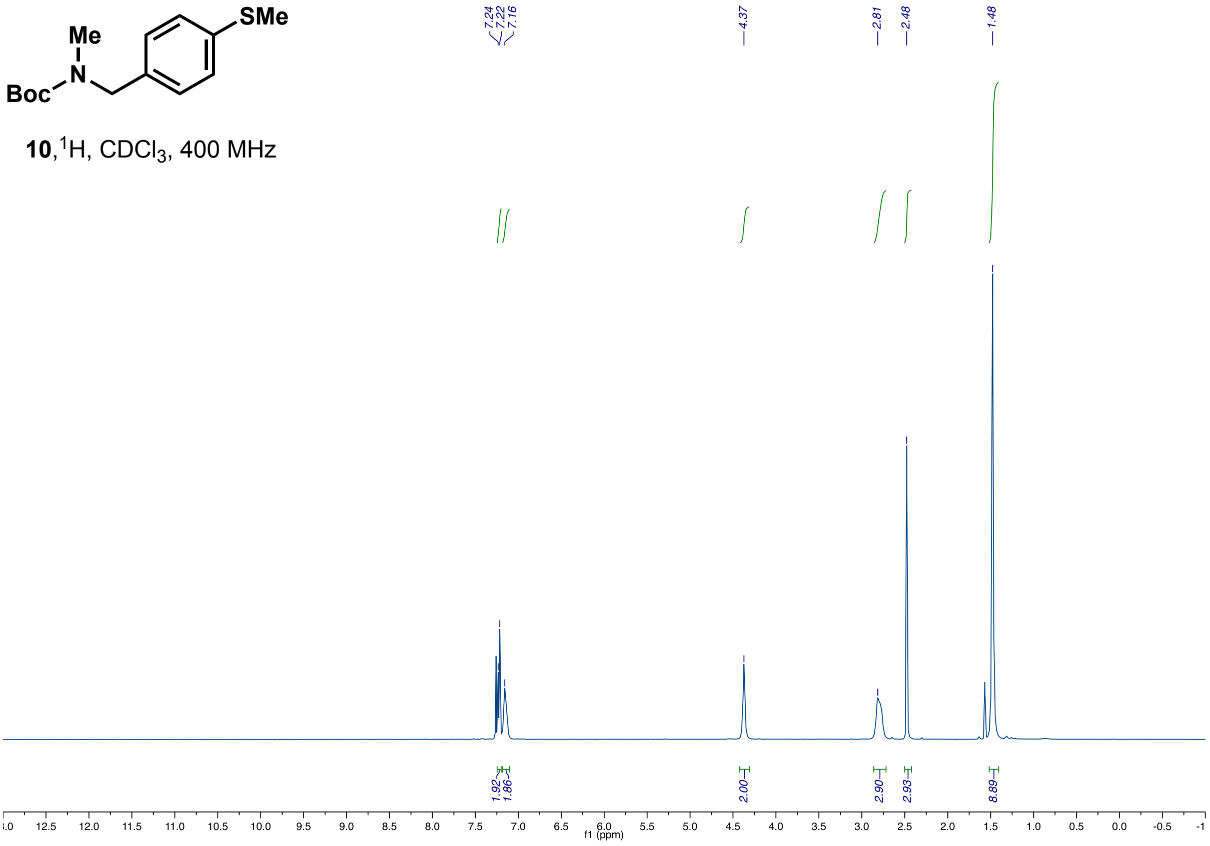
SMe
Me
N
Boc
10,¹H, CDCl3, 400 MHz
7.24
7.22
7.16
4.37
2.81
2.48
1.48
1.92
1.86
2.00
2.90
2.93
8.89
f1 (ppm)

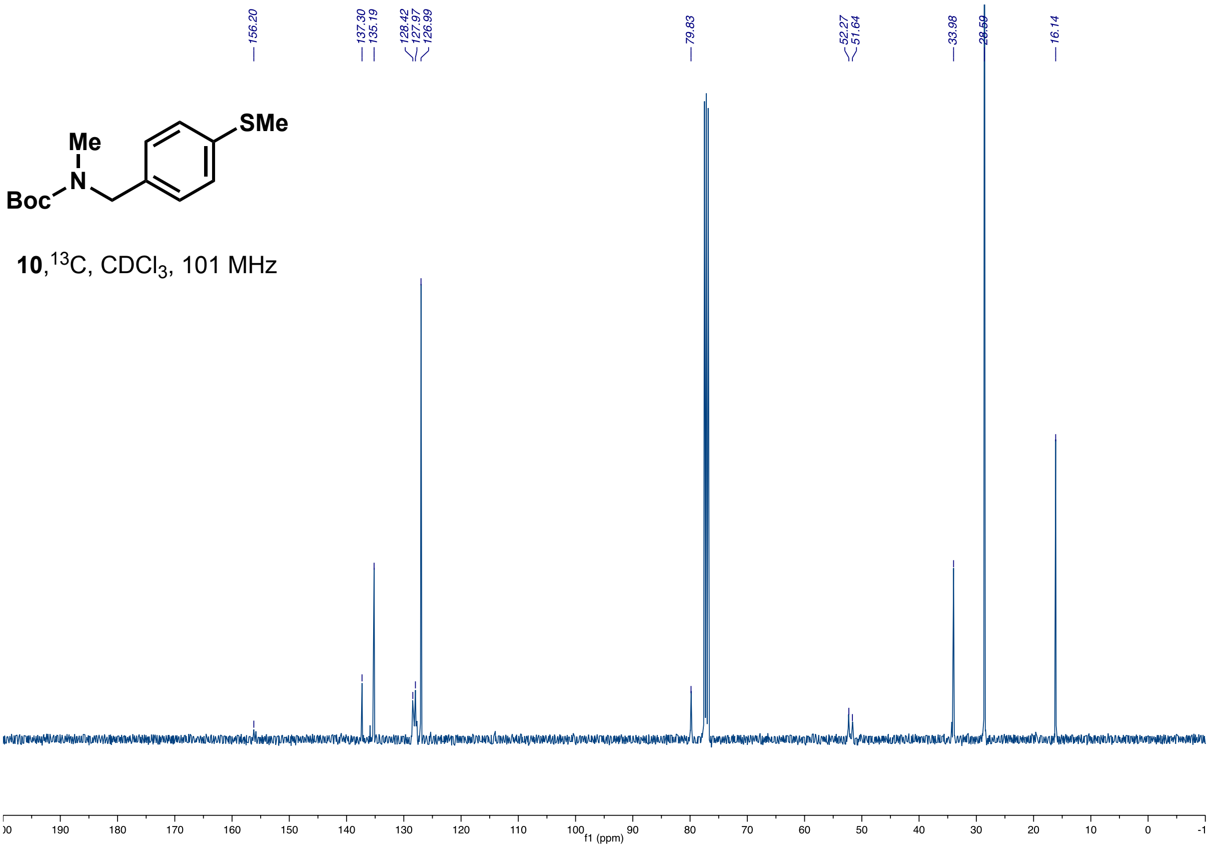
156.20
137.30
135.19
128.42
127.97
126.99
79.83
52.27
51.64
33.98
28.59
16.14
SMe
Me
N
Boc
10,¹³C, CDCl3, 101 MHz
f1 (ppm)

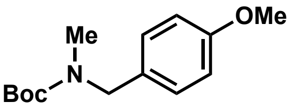

11, ^{1}H, $CDCl_3$, 400 MHz

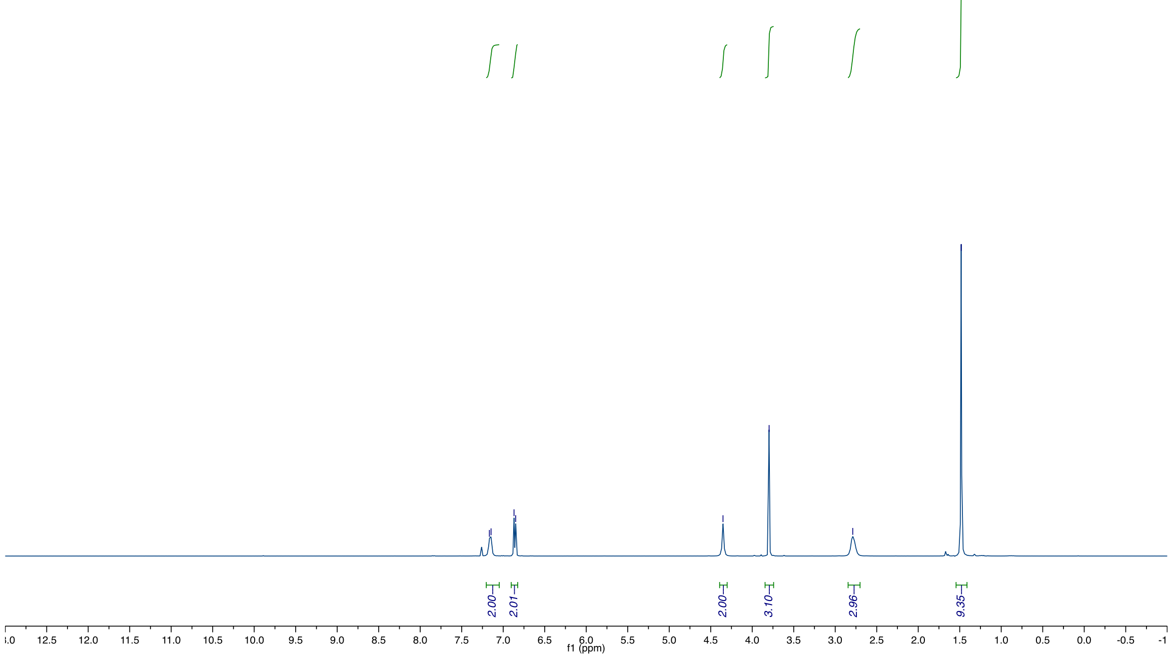

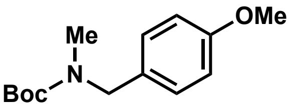

11, ^{13}C, $CDCl_3$, 101 MHz

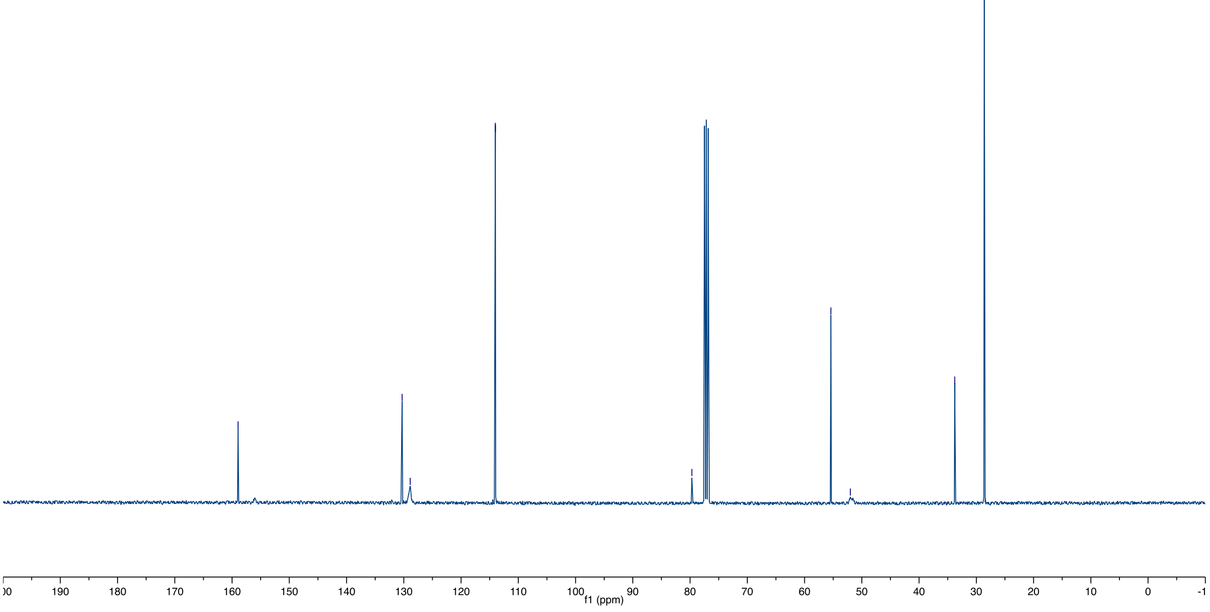

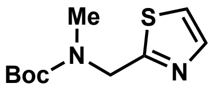

12, ^{1}H, $CDCl_3$, 400 MHz

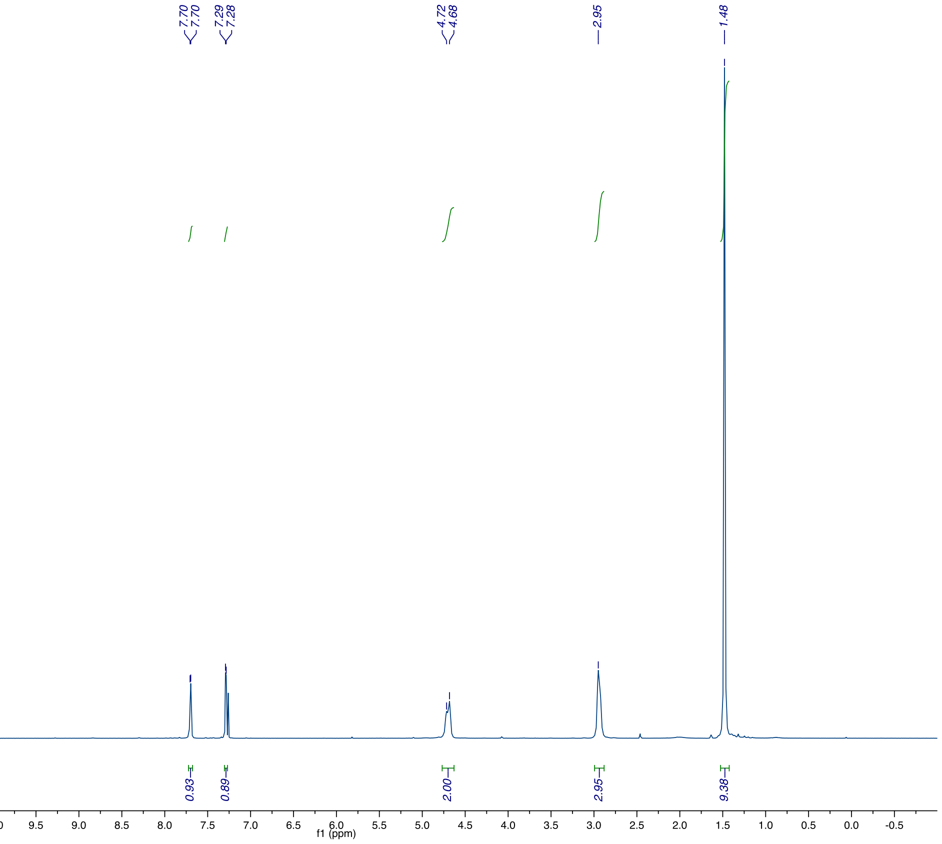

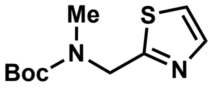

12, ^{13}C, $CDCl_3$, 101 MHz

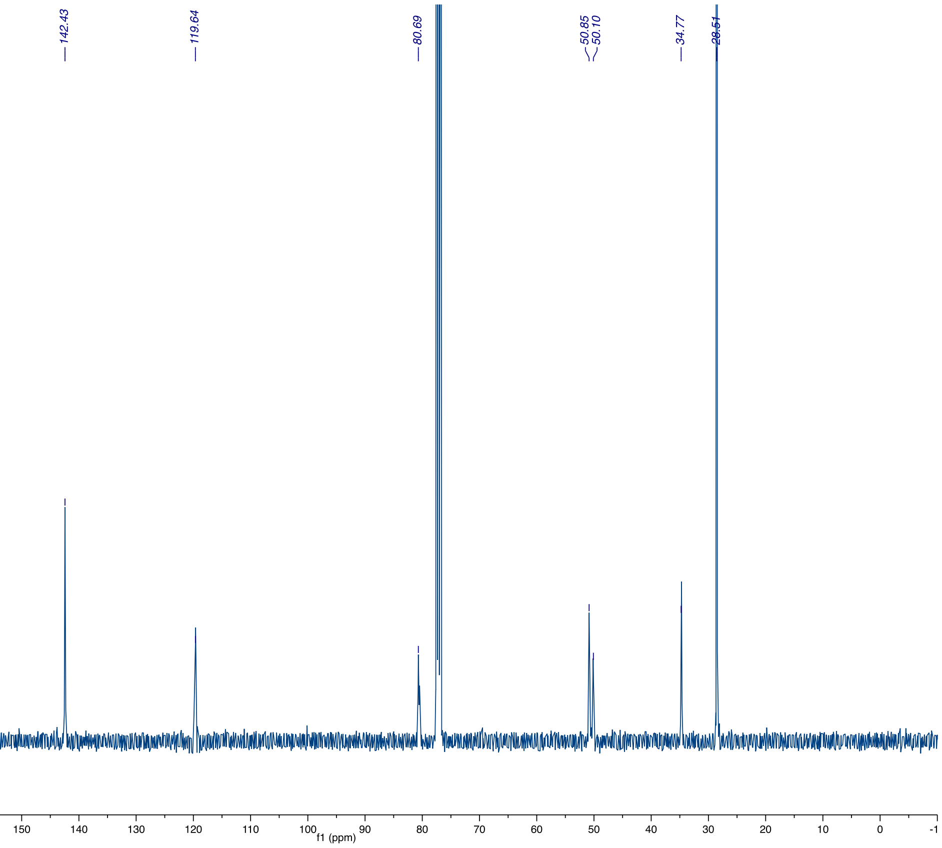

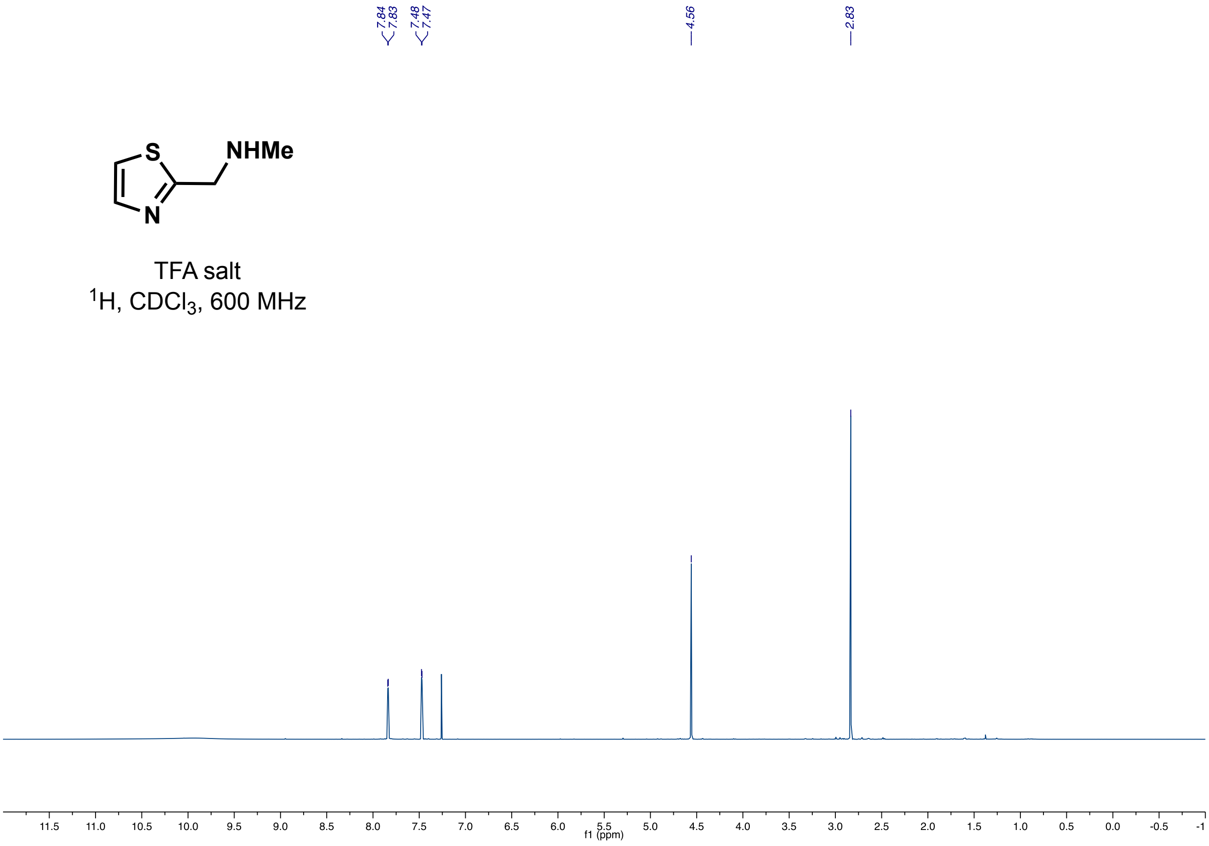
7.84
7.83
7.48
7.47
4.56
2.83
S
N
NHMe
TFA salt
^{1}H, $CDCl_3$, 600 MHz
f1 (ppm)

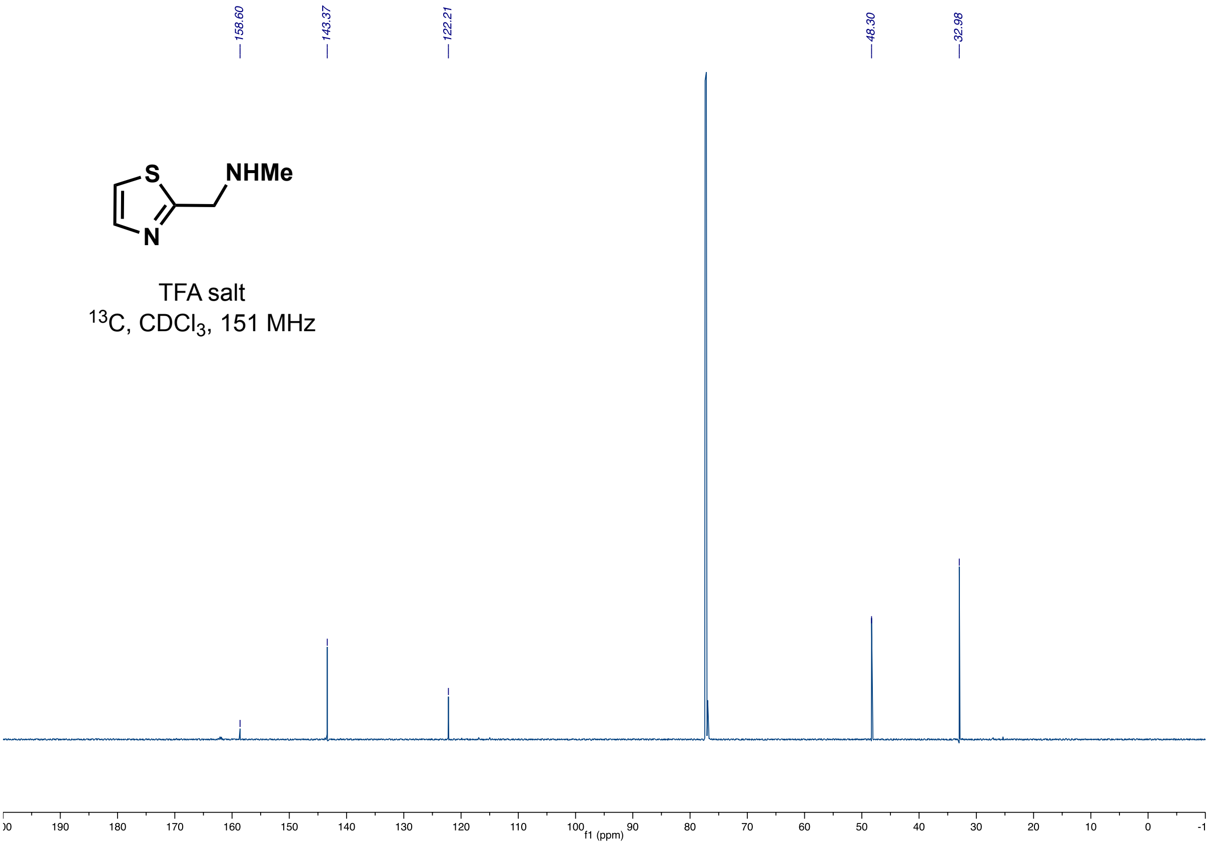
158.60
143.37
122.21
48.30
32.98
S
N
NHMe
TFA salt
^{13}C, $CDCl_3$, 151 MHz
f1 (ppm)

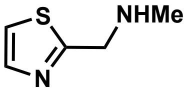

TFA salt
^{19}F, $CDCl_3$, 376 MHz
(CF_3CH_2OH as internal standard)

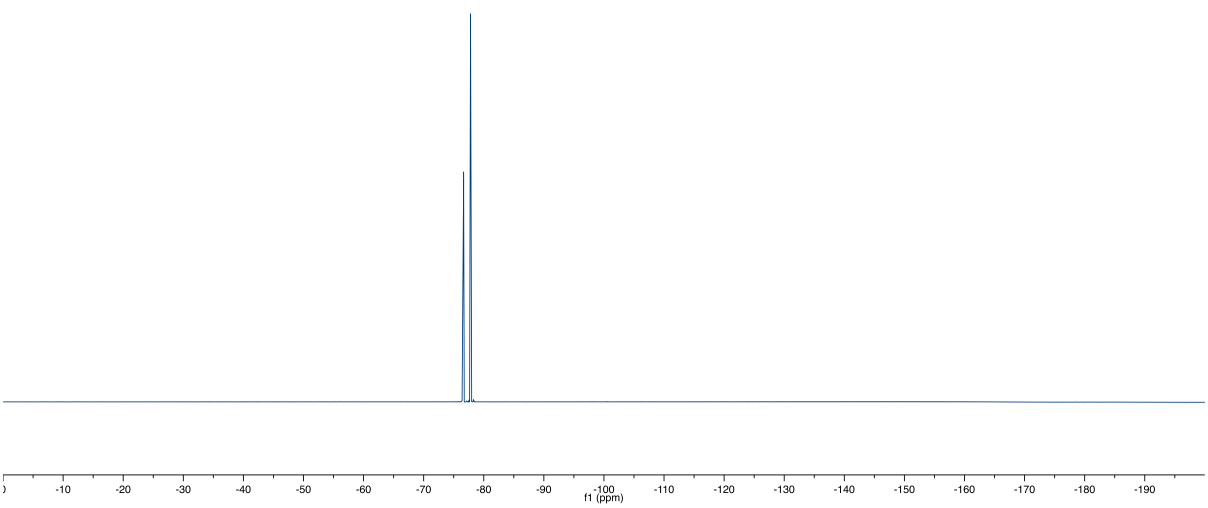

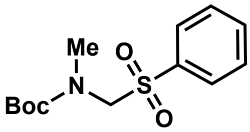

13, ^{1}H, $CDCl_3$, 700 MHz

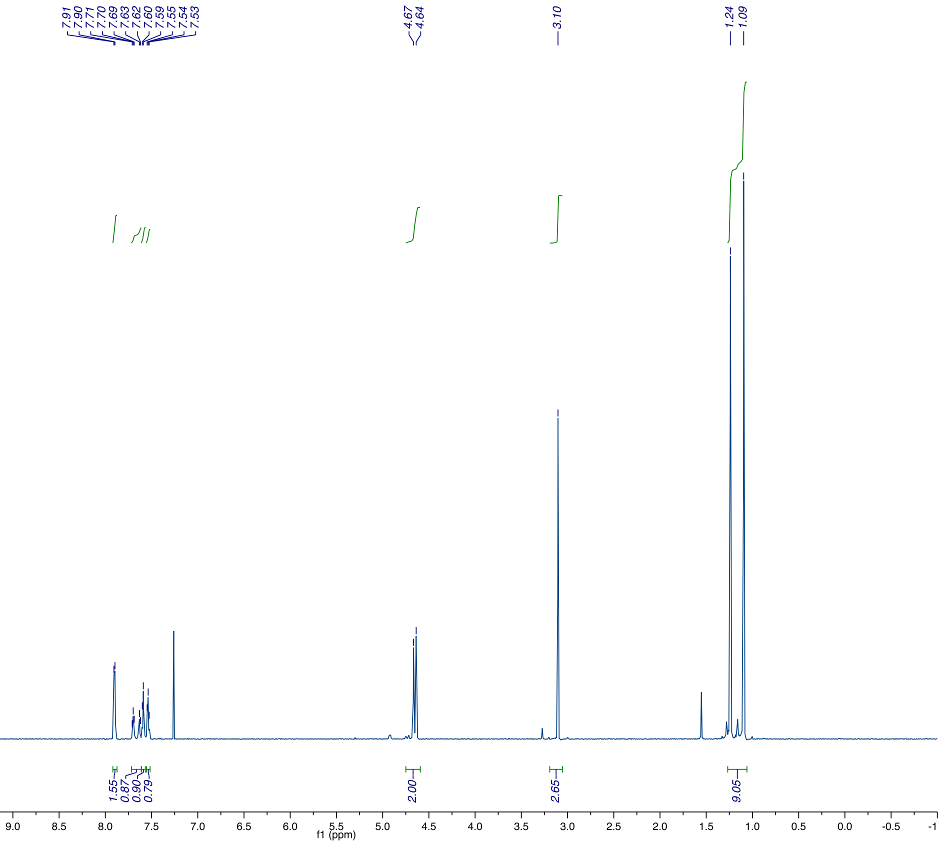

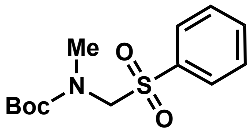

13, ^{13}C, $CDCl_3$, 176 MHz

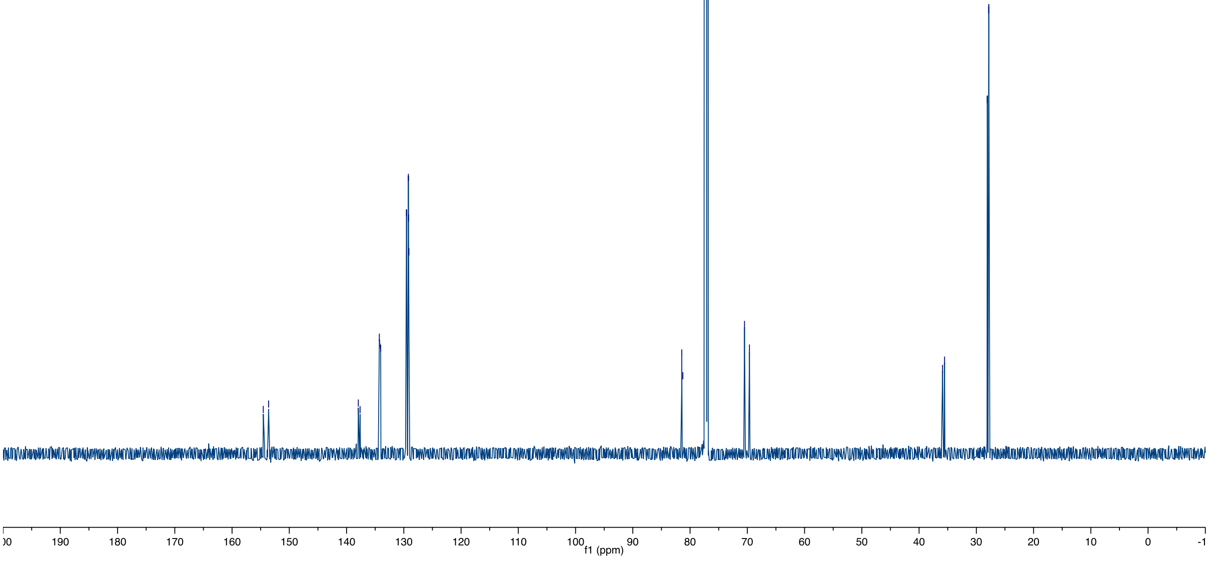

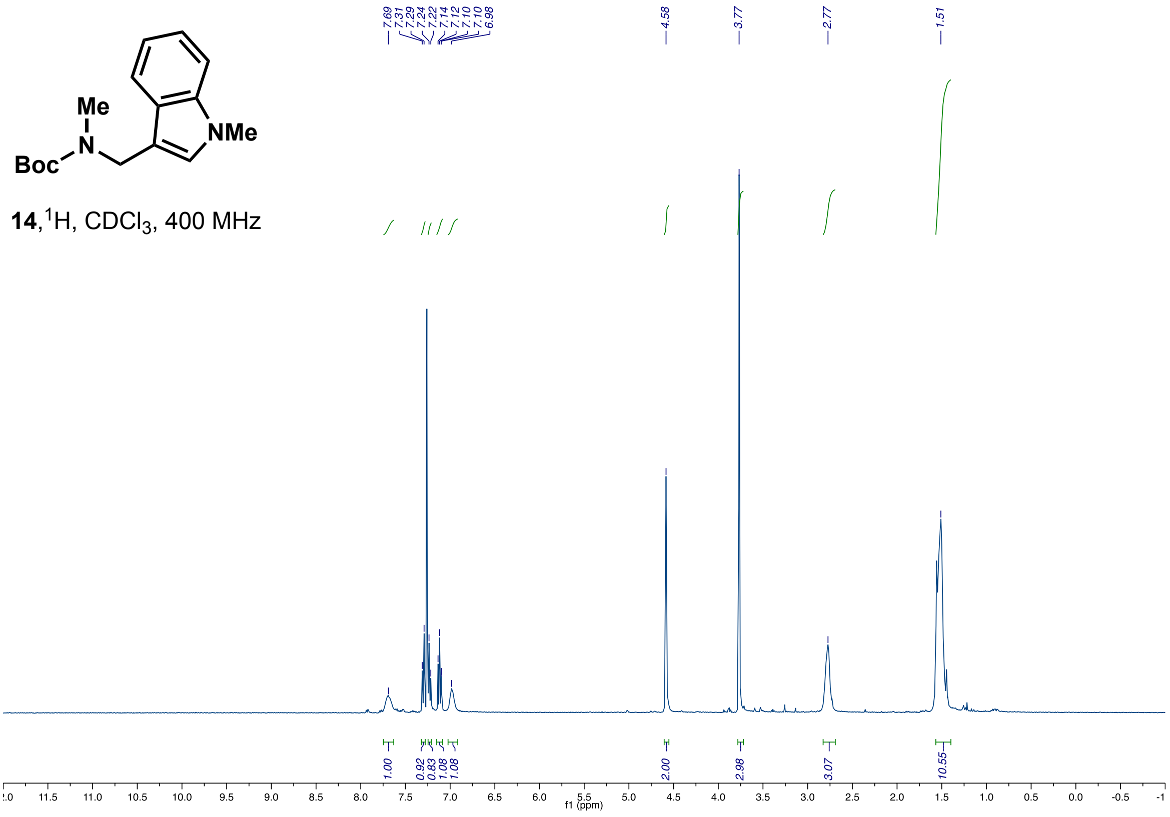

14,^{1}H, $CDCl_3$, 400 MHz

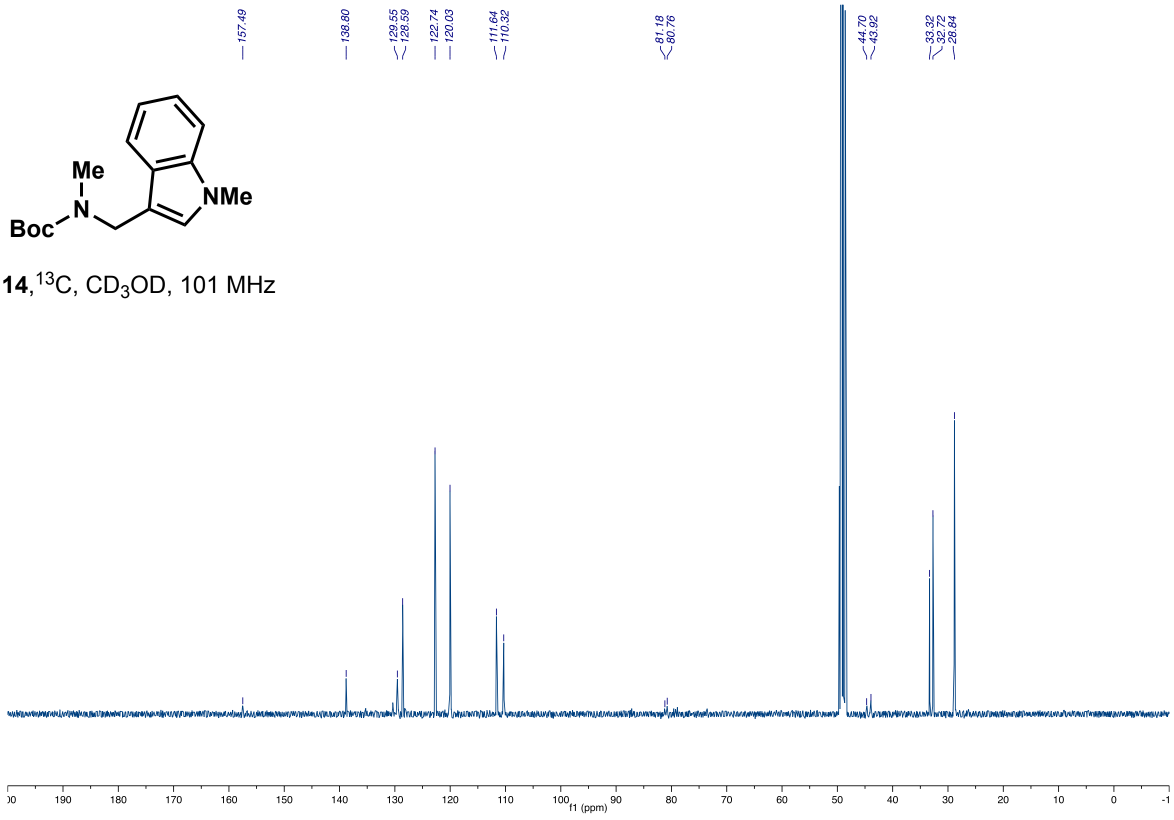

14,^{13}C, CD_3OD, 101 MHz

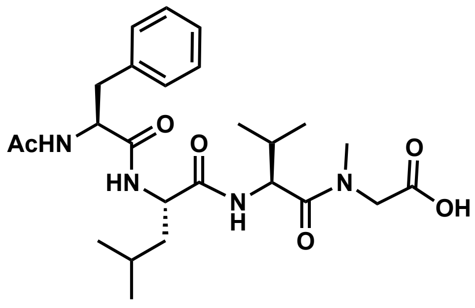

15, ^{1}H, CD_3OD, 400 MHz

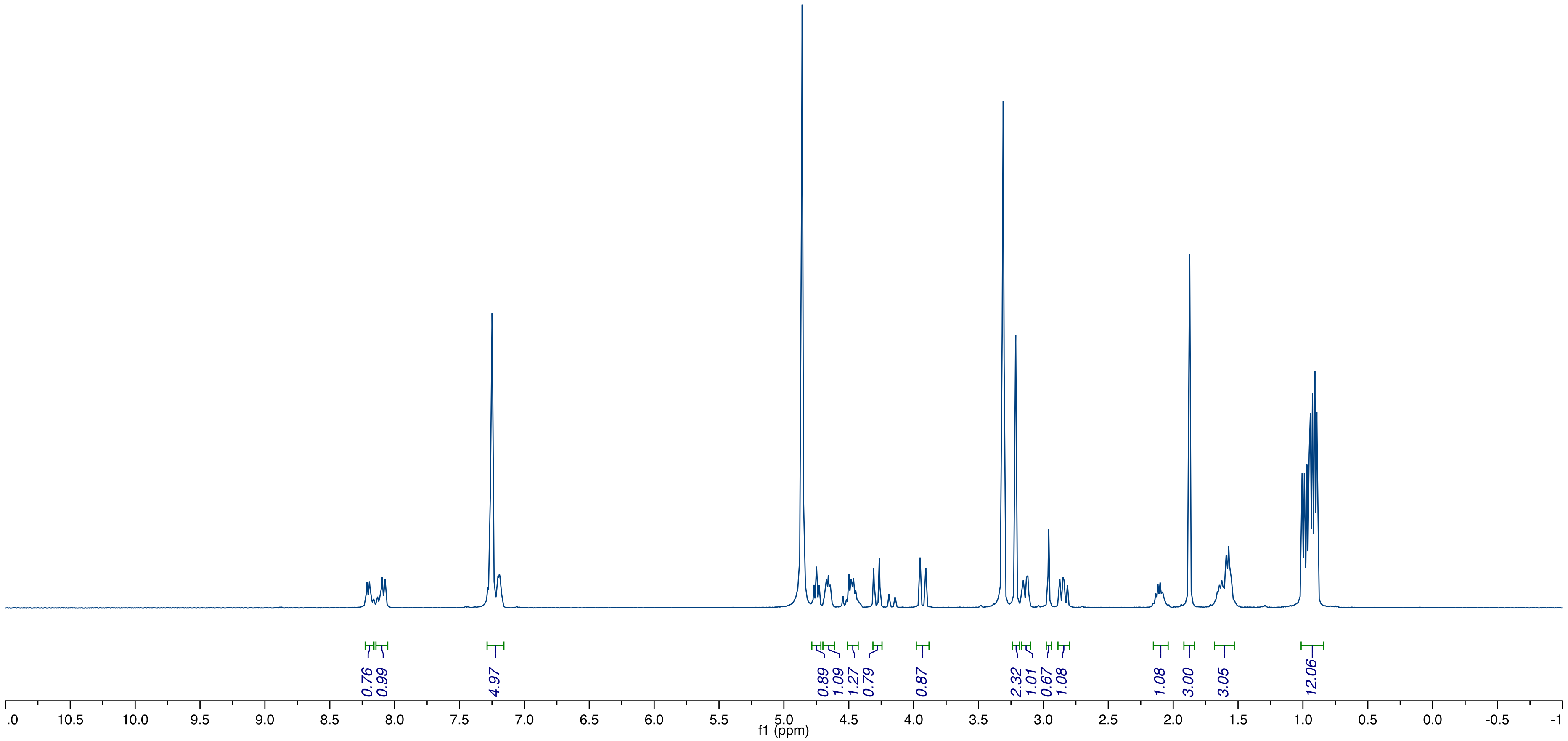

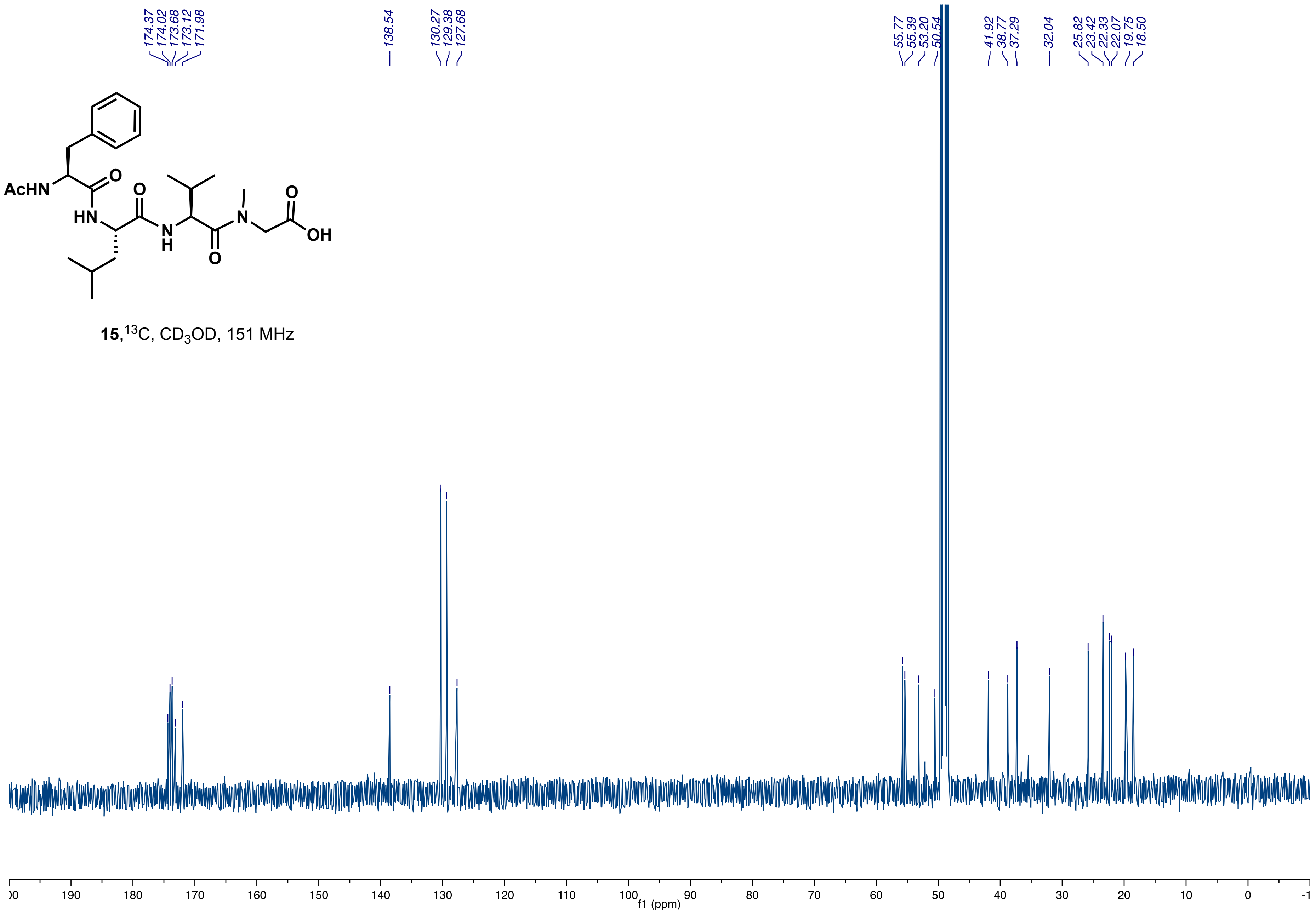

15, ^{13}C, CD_3OD, 151 MHz

15, COSY, CD_3OD, 400 MHz

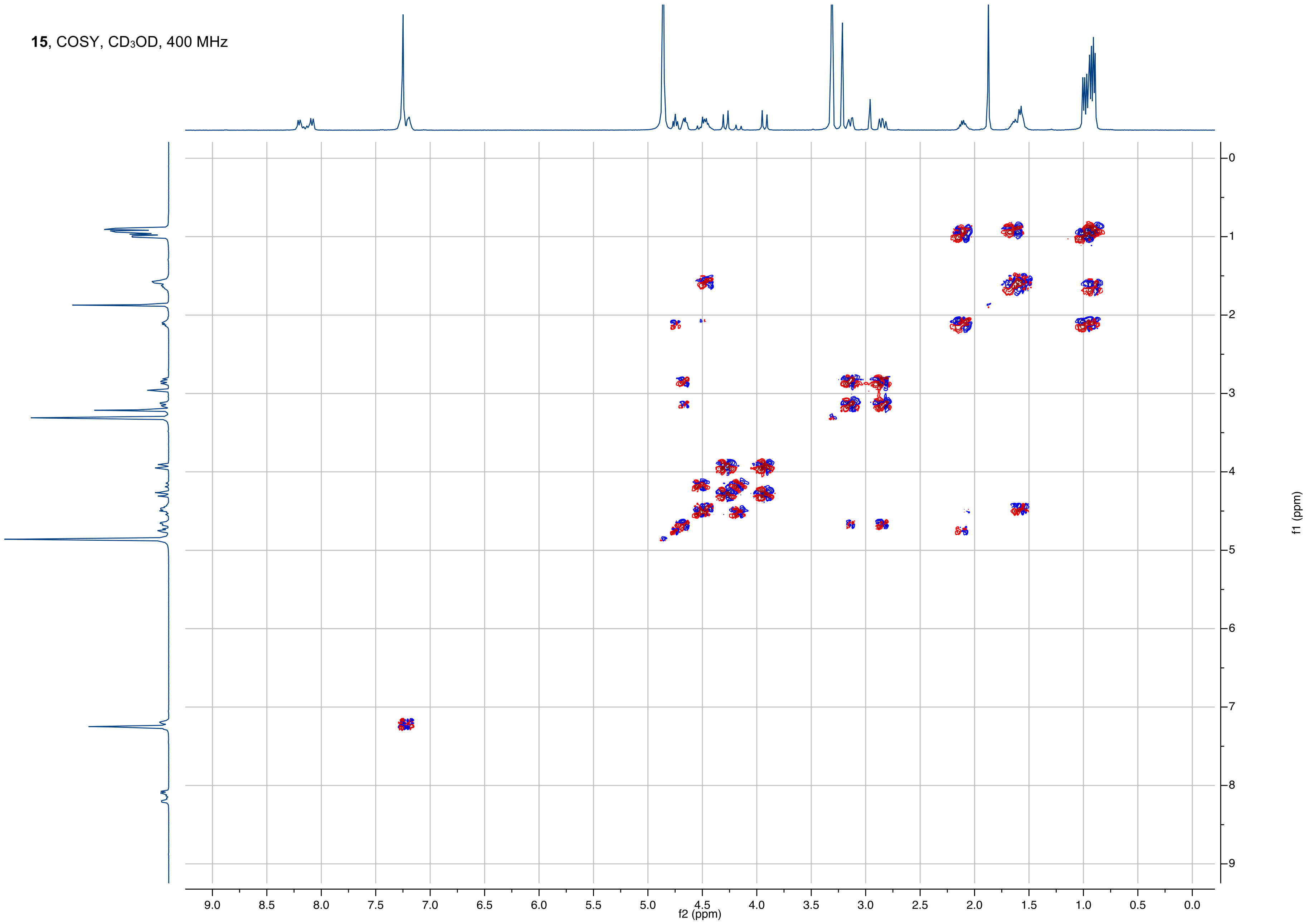

15, HSQC, CD_3OD, 400 MHz

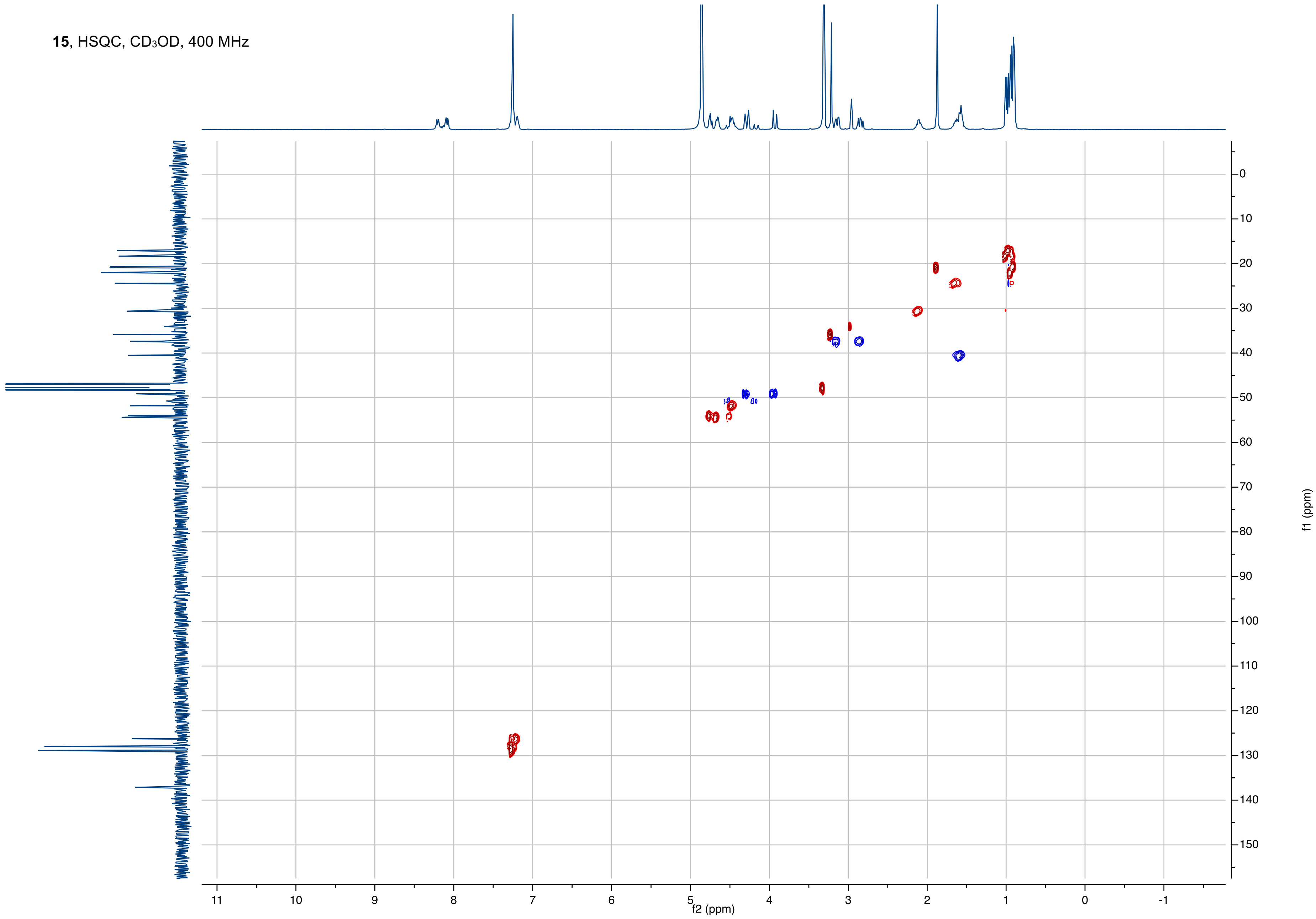

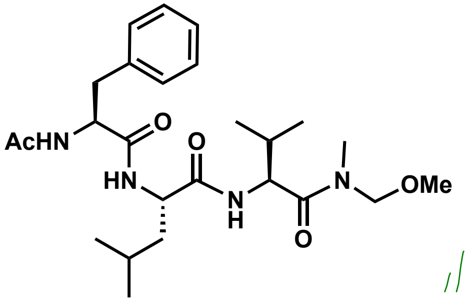

16, ^{1}H, CD_3OD, 700 MHz

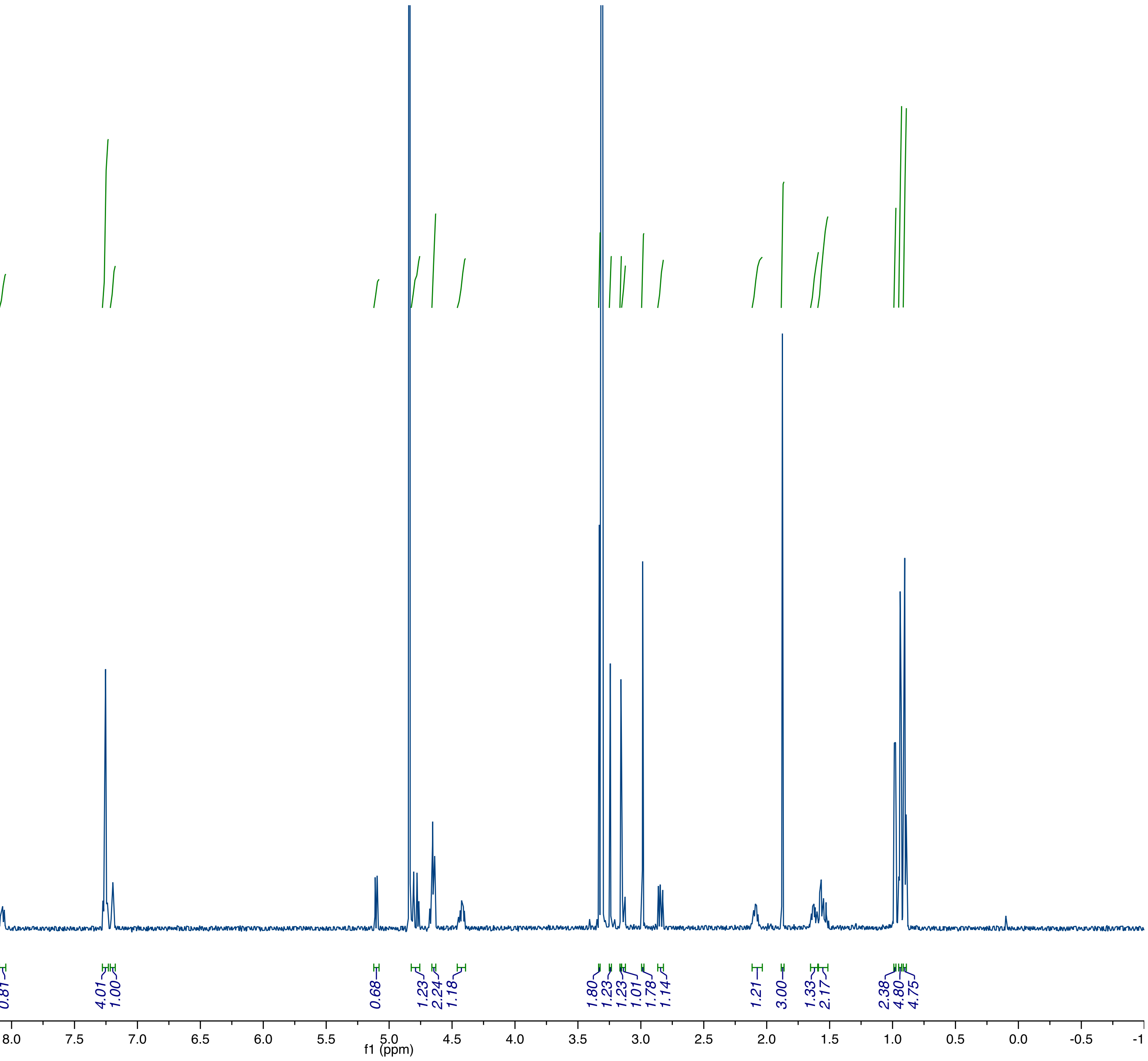

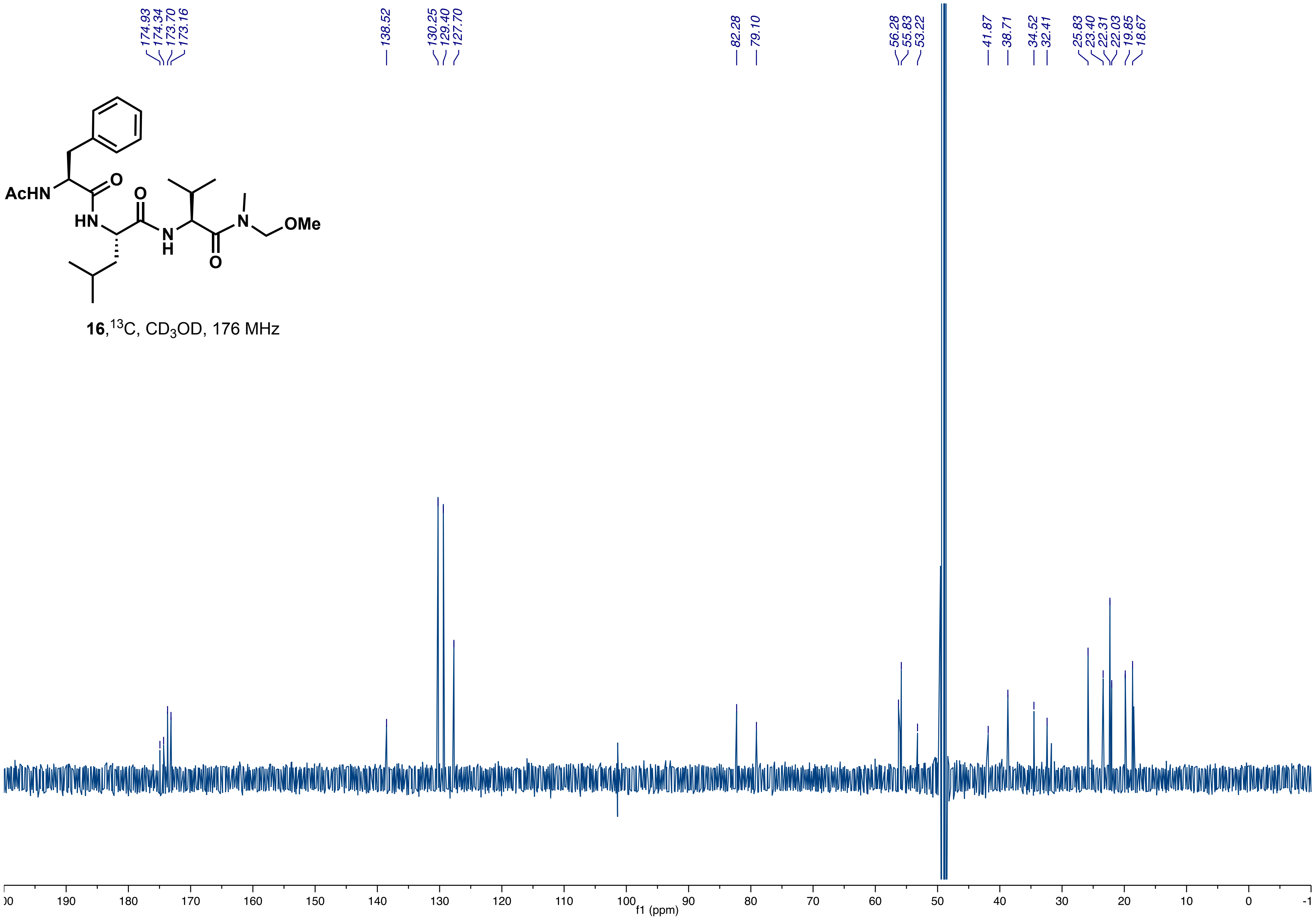

16, ^{13}C, CD_3OD, 176 MHz

16, COSY, CD_3OD, 700 MHz

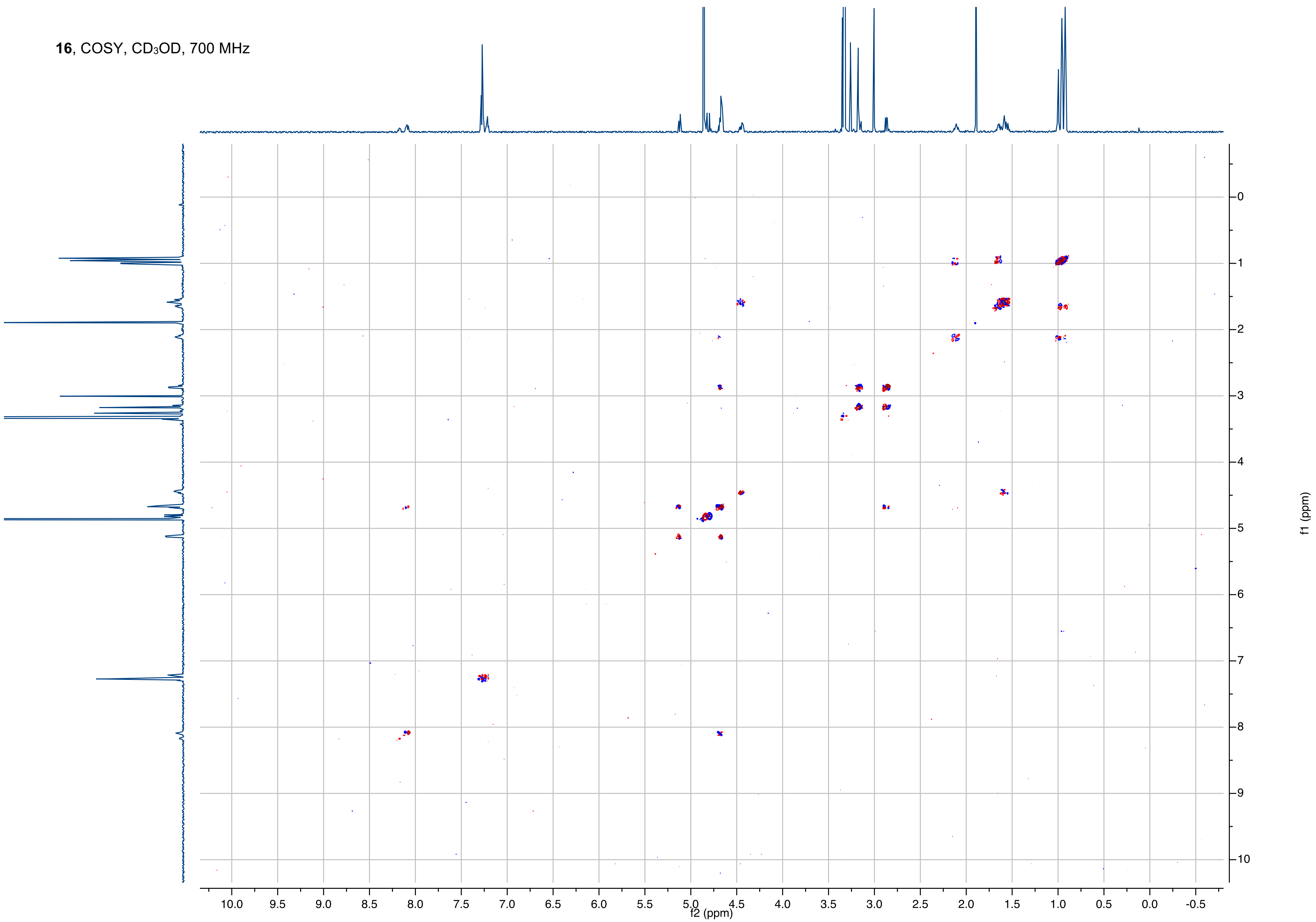

16,HSQC, CD_3OD, 700 MHz

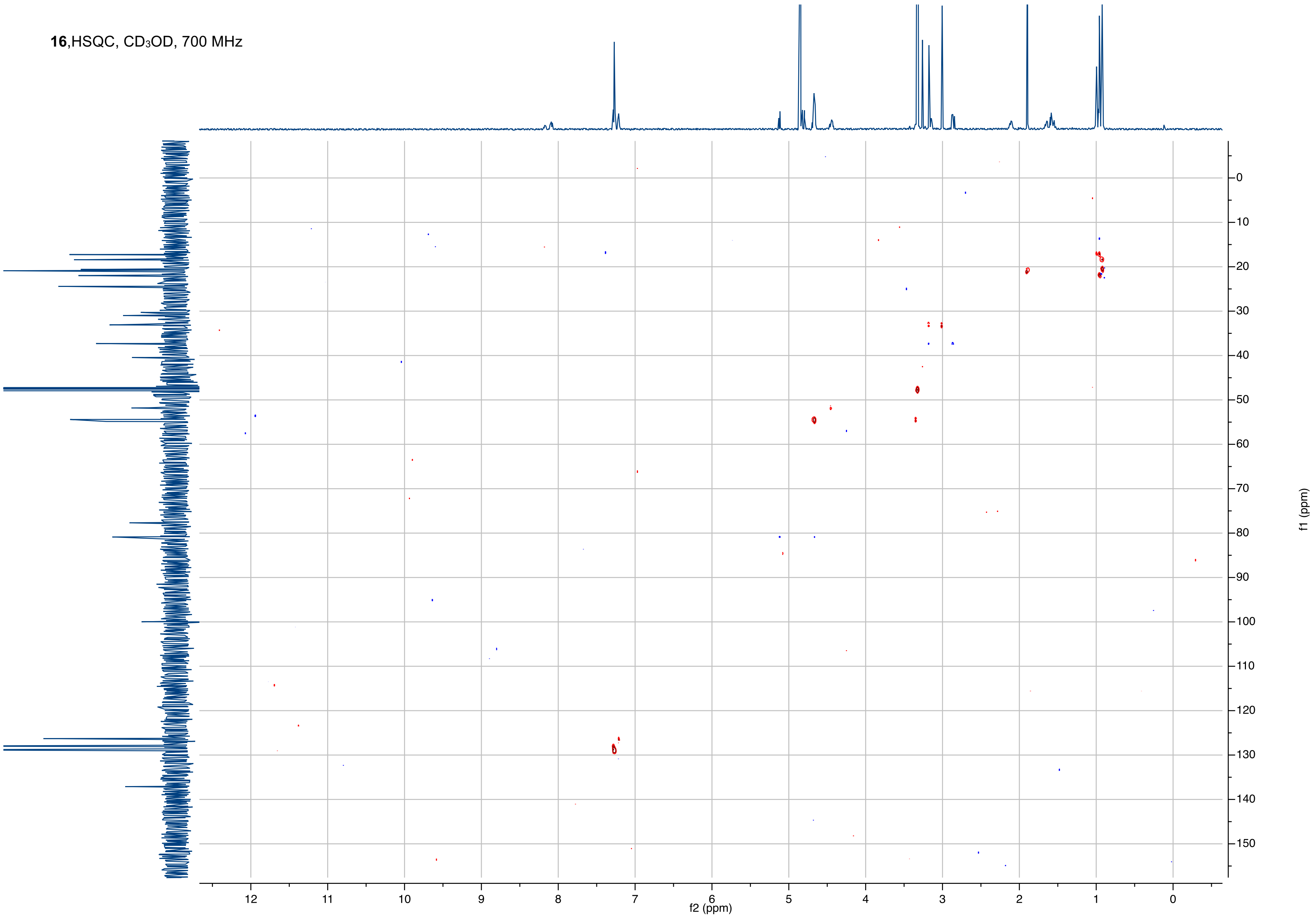

16, HMBC, CD_3OD, 700 MHz

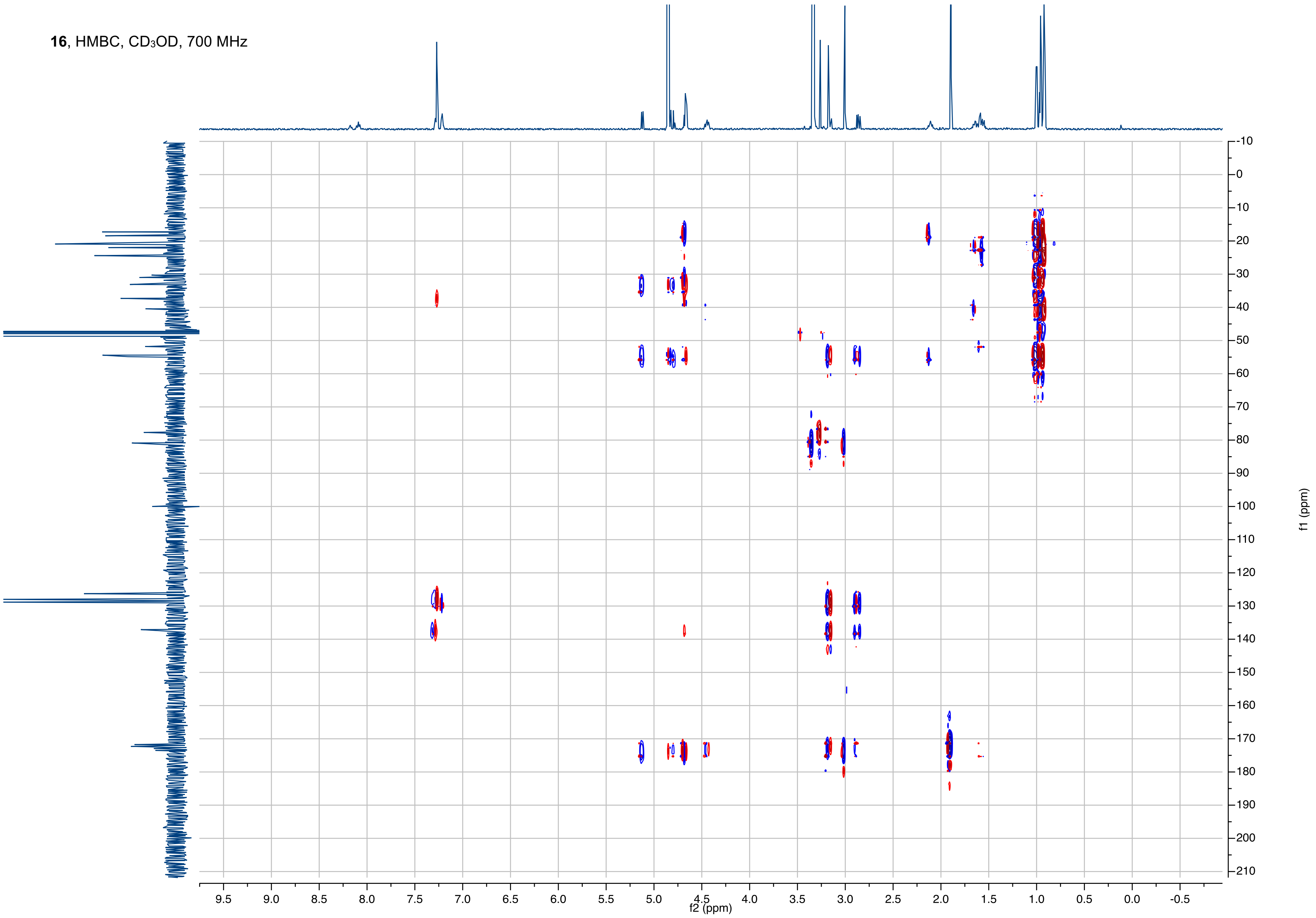

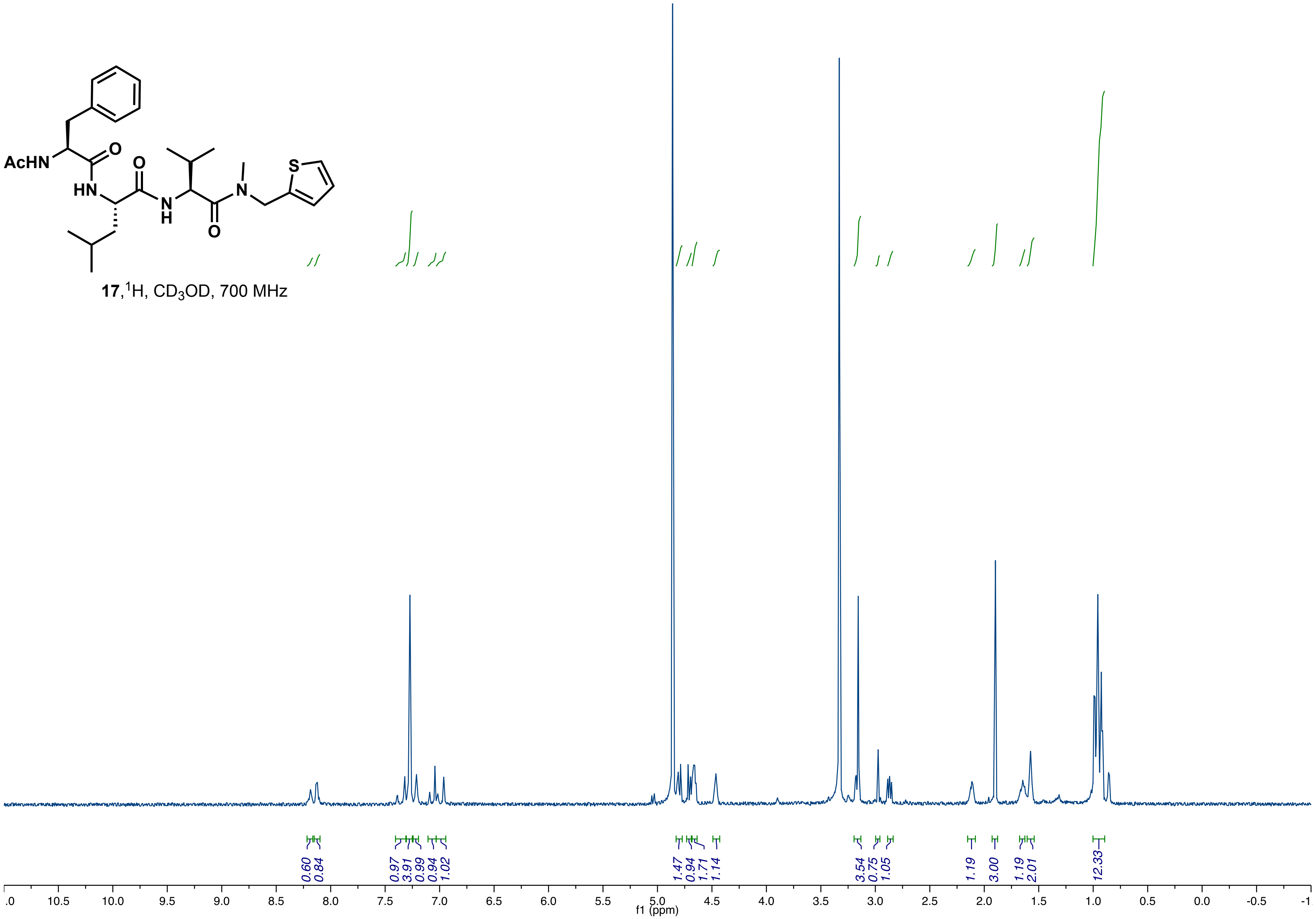

17,^{1}H, CD_3OD, 700 MHz

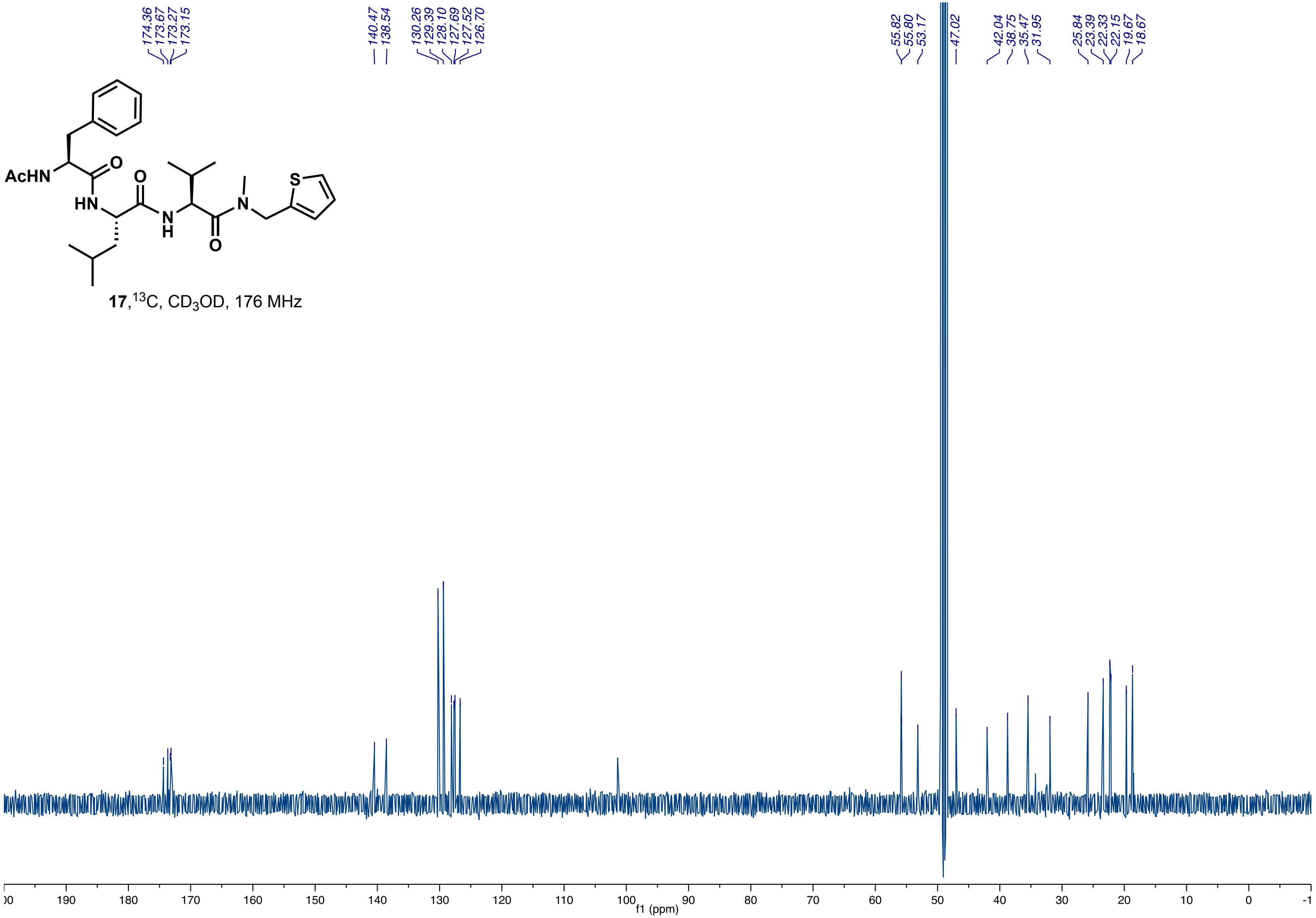
174.36
173.67
173.27
173.15
140.47
138.54
130.26
129.39
128.10
127.69
127.52
126.70
55.82
55.80
53.17
47.02
42.04
38.75
35.47
31.95
25.84
23.39
22.33
22.15
19.67
18.67
AcHN
HN
N
H
O
O
O
N
S
17, 13C, CD3OD, 176 MHz
190
180
170
160
150
140
130
120
110
100
f1 (ppm)
90
80
70
60
50
40
30
20
10
0
-1

17, COSY, CD_3OD, 700 MHz

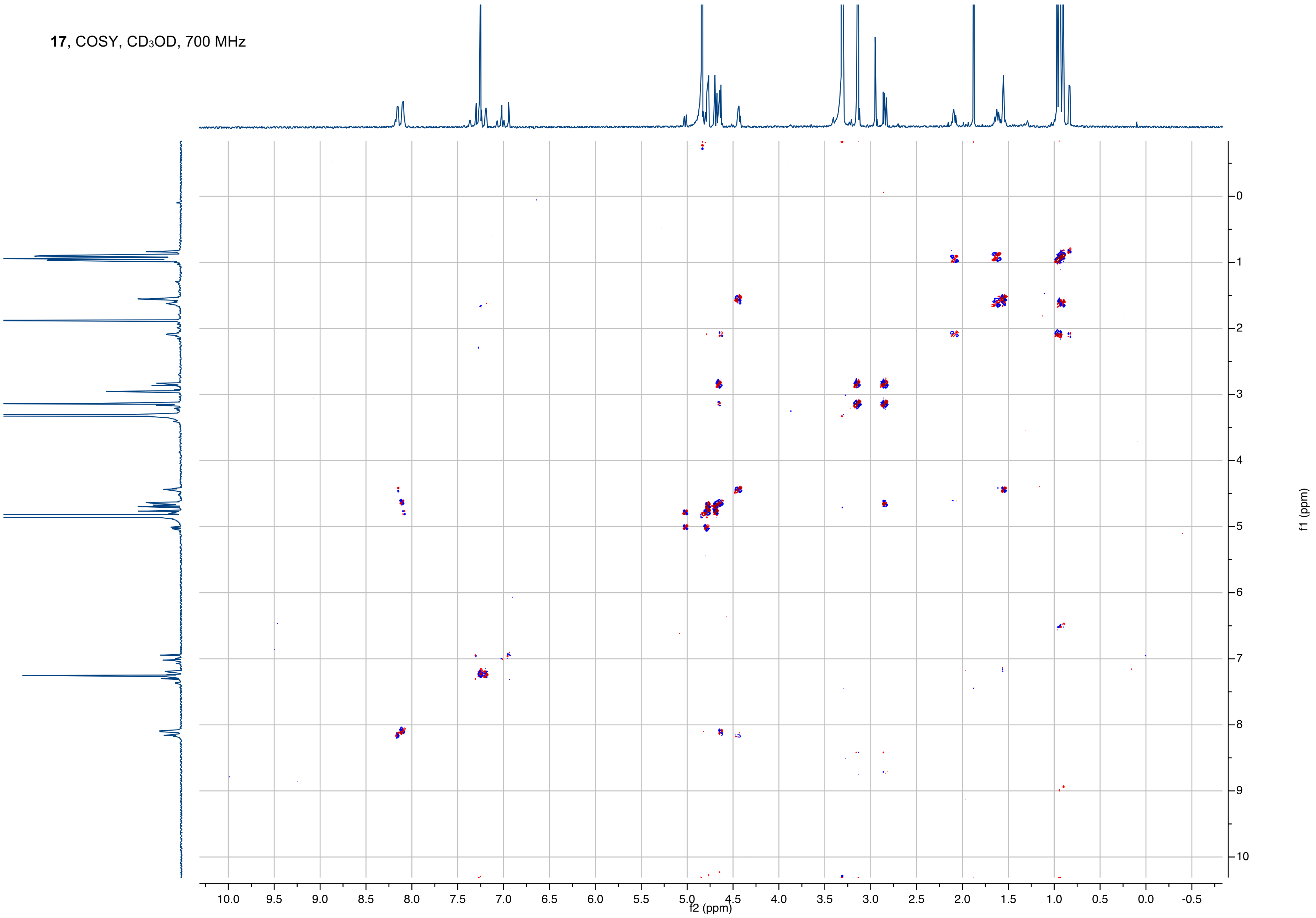

17, HSQC, CD_3OD, 700 MHz

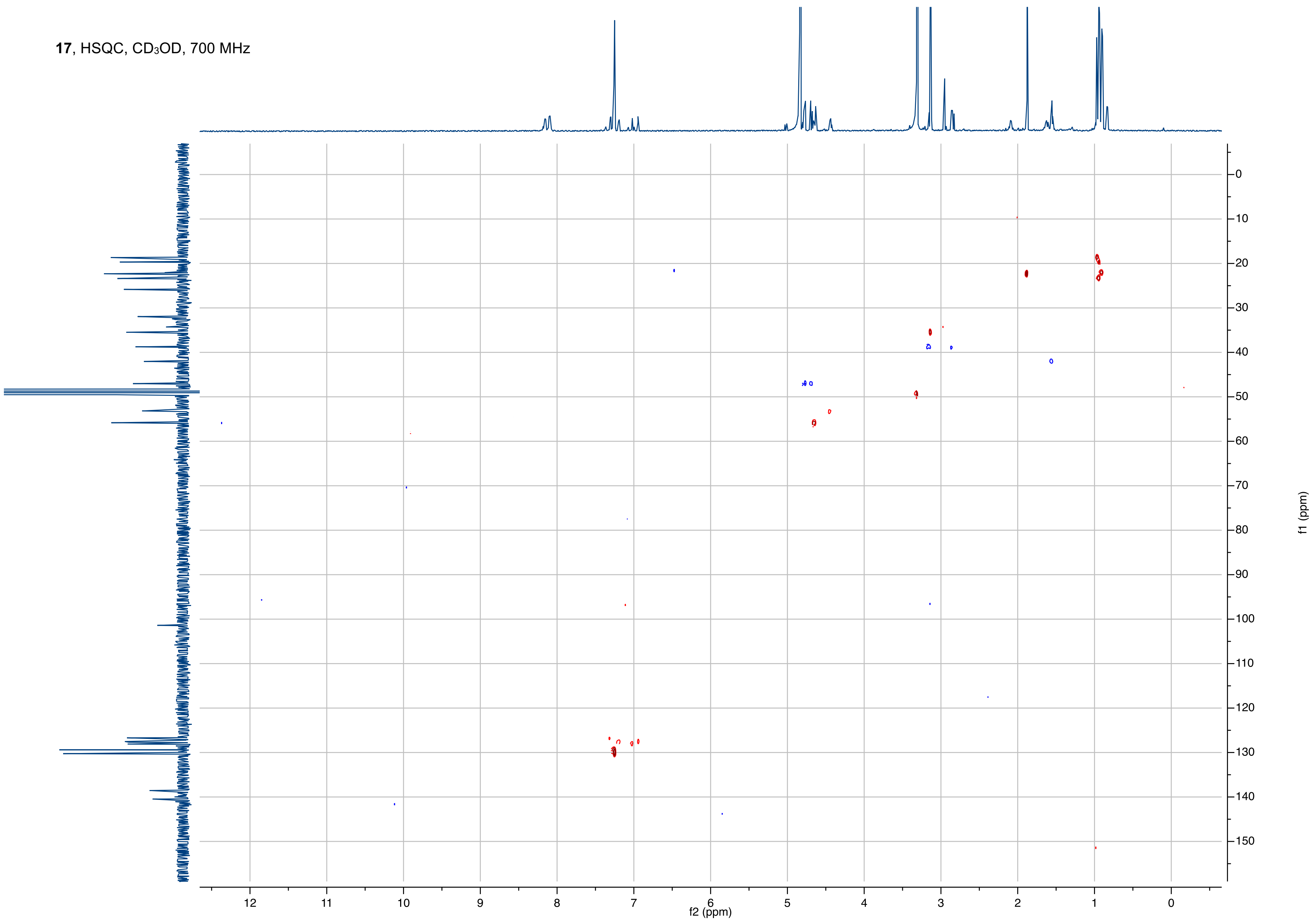

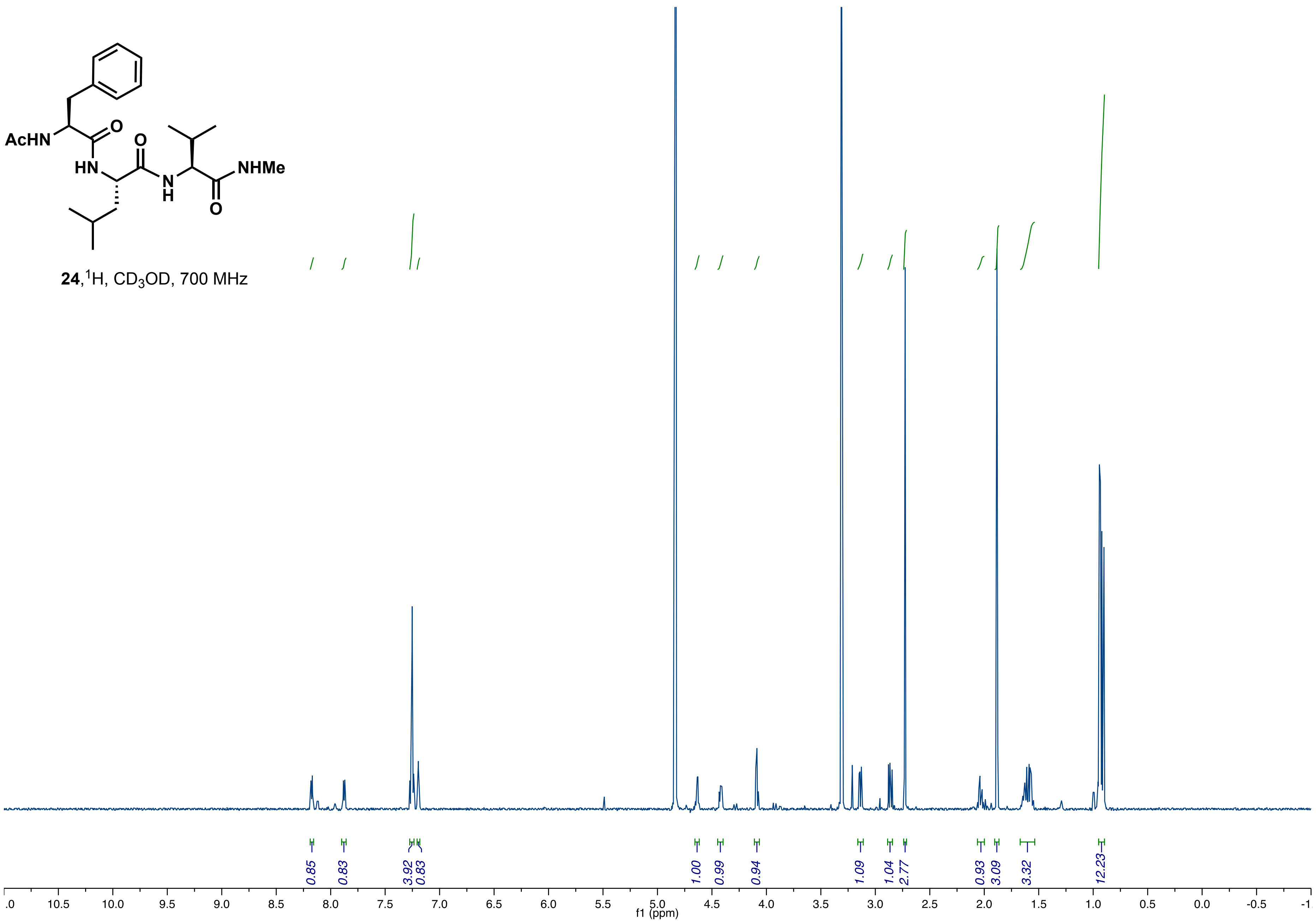

24, ^{1}H, CD_3OD, 700 MHz

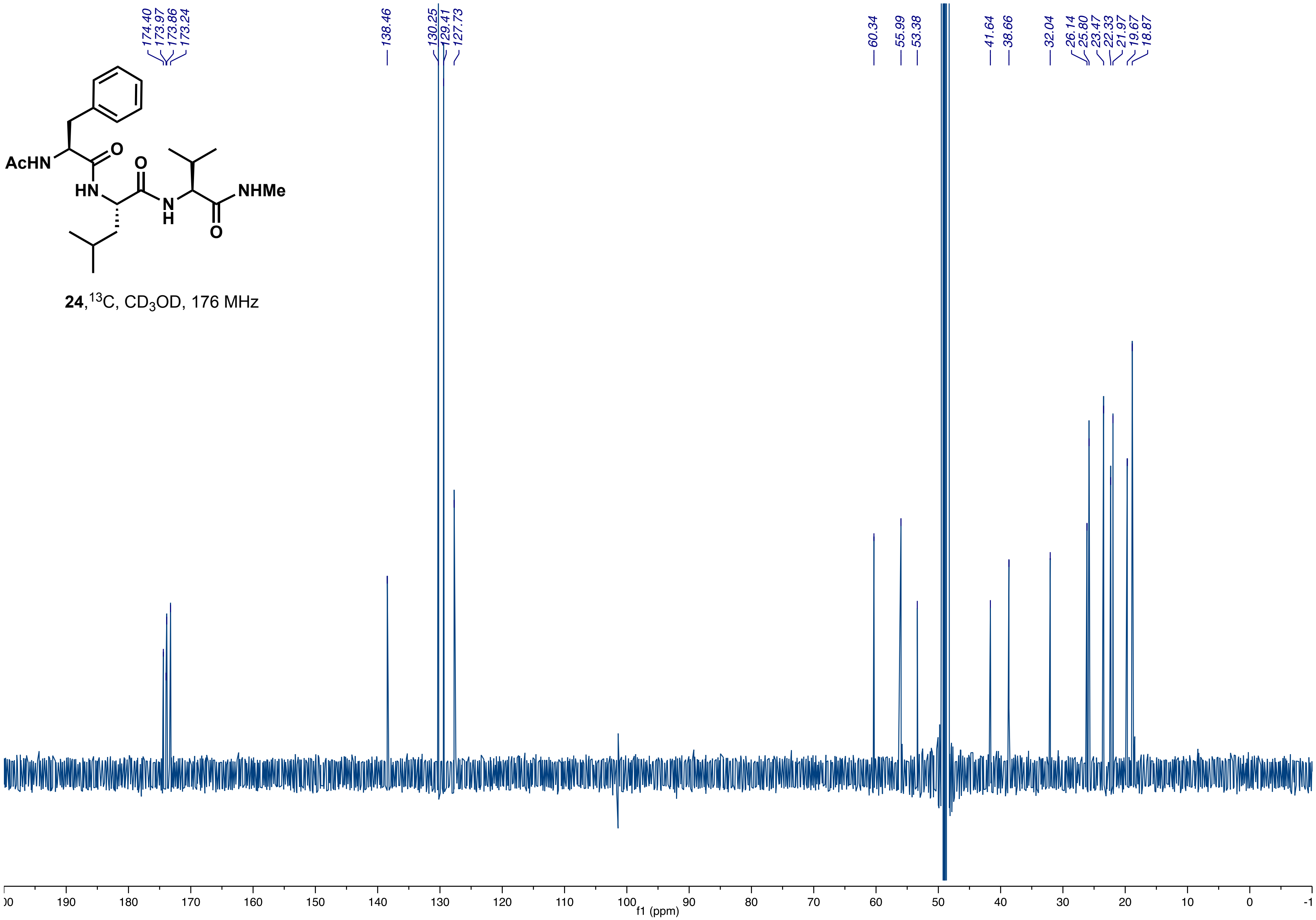

24,^{13}C, CD_3OD, 176 MHz

24, COSY, CD_3OD, 700 MHz

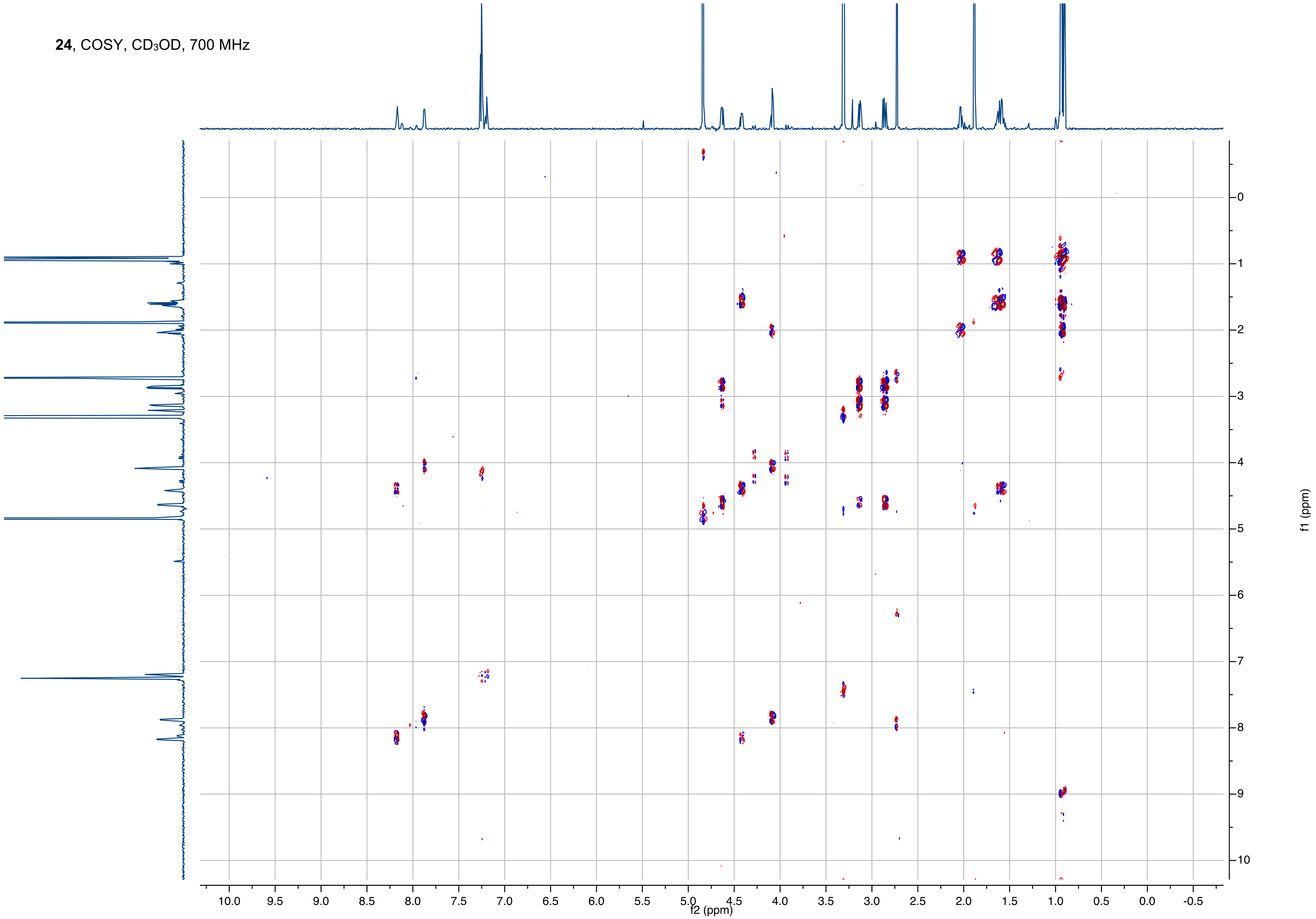

24, HSQC, CD_3OD, 700 MHz

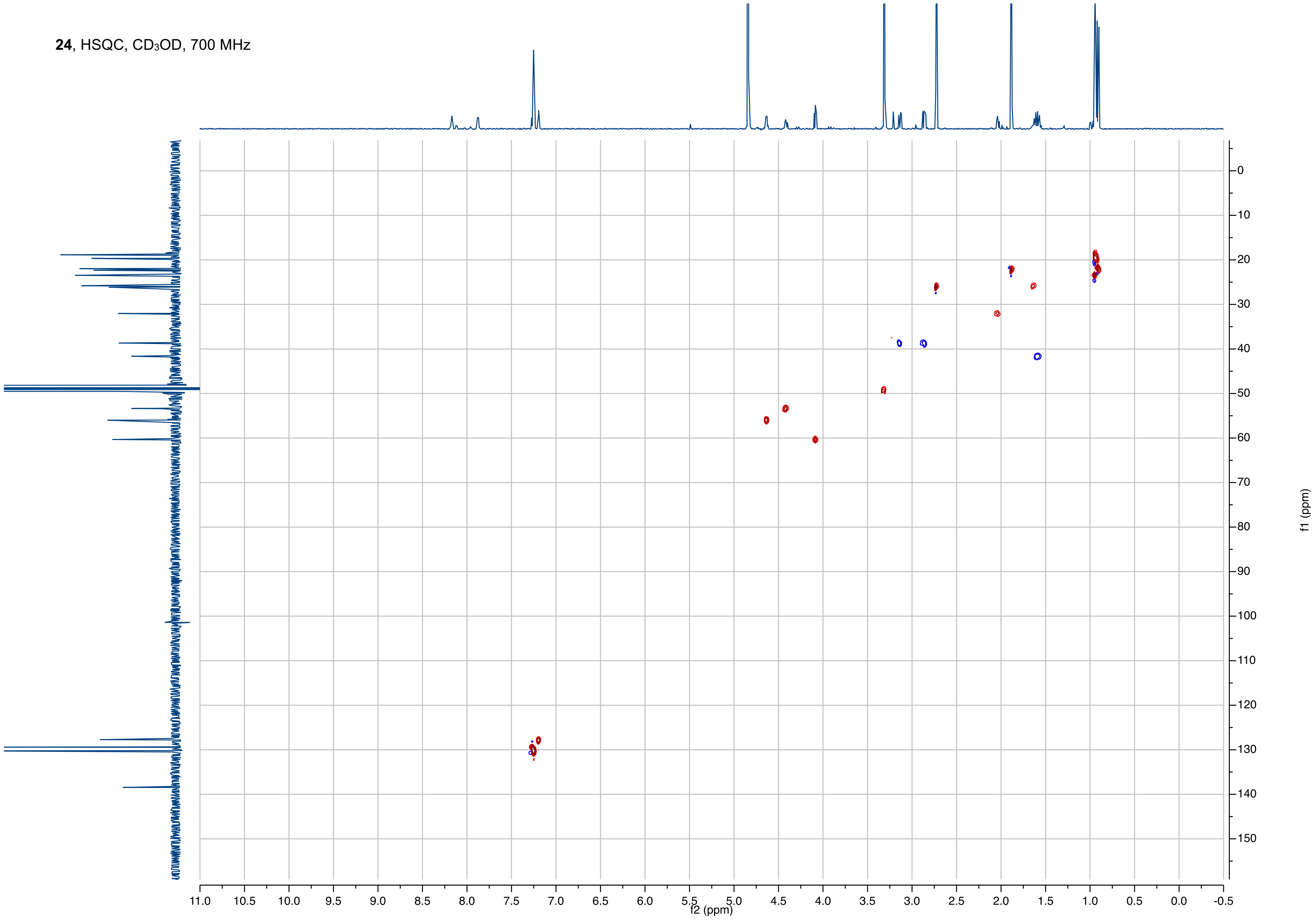

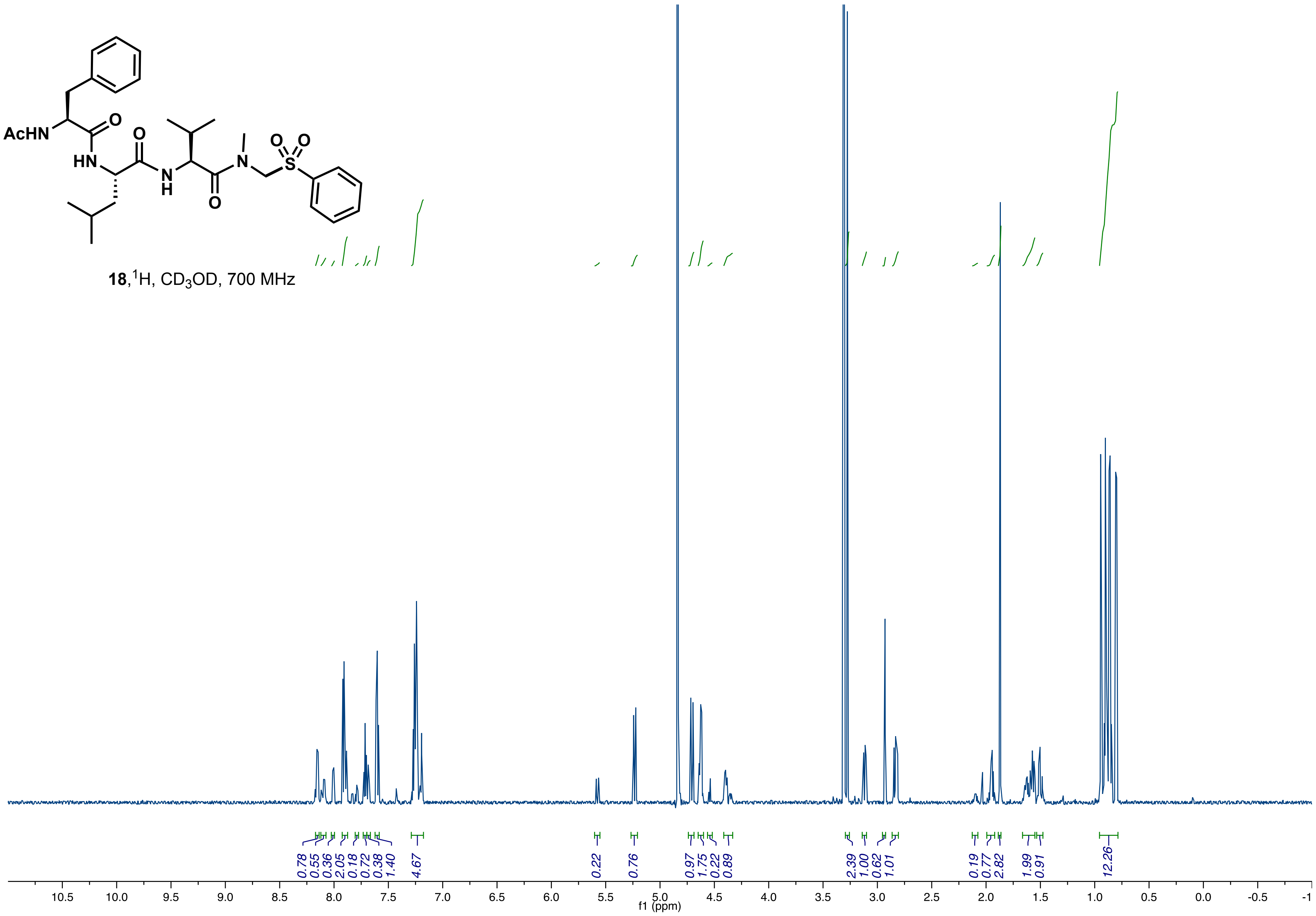

18, ^{1}H, CD_3OD, 700 MHz

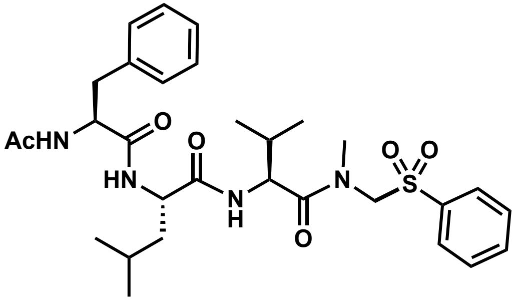

18, ^{13}C, CD_3OD, 176 MHz

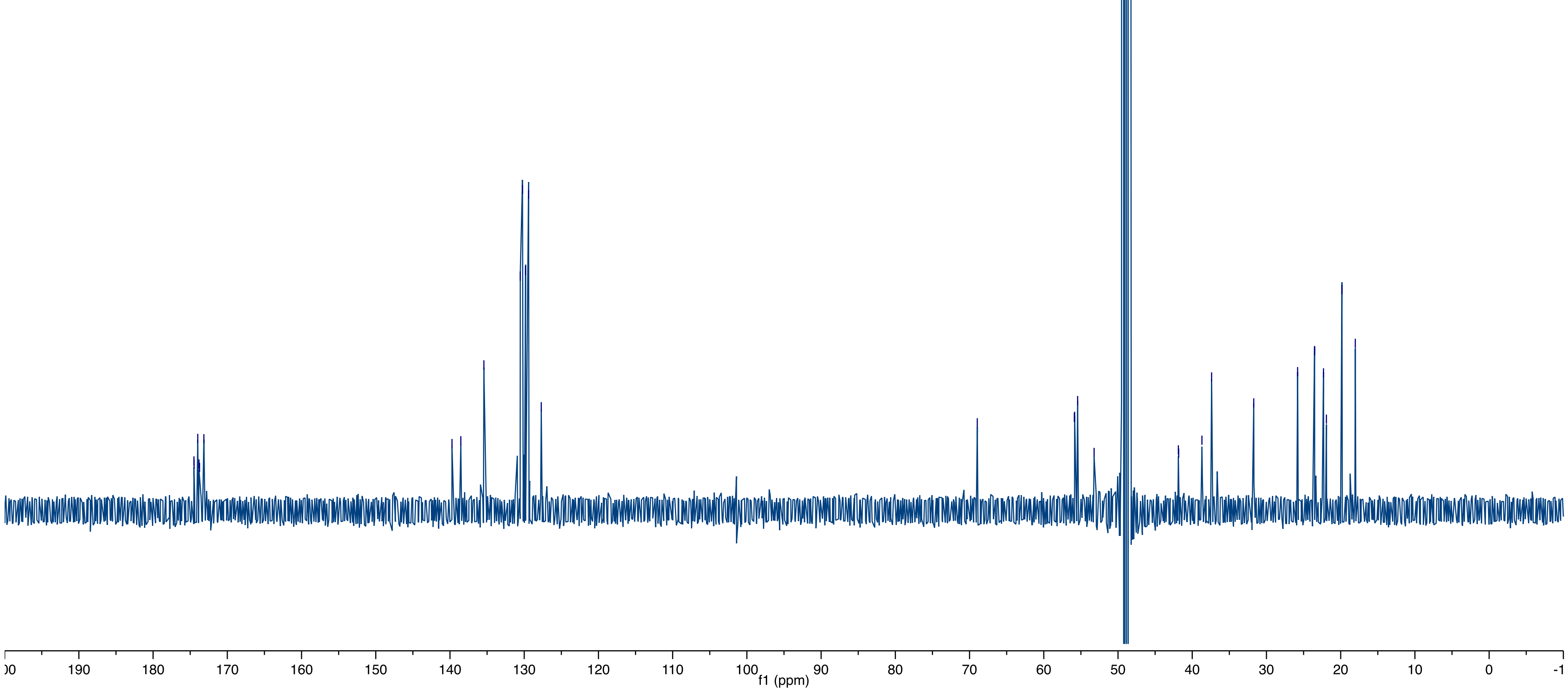

18, COSY, CD_3OD, 700MHz

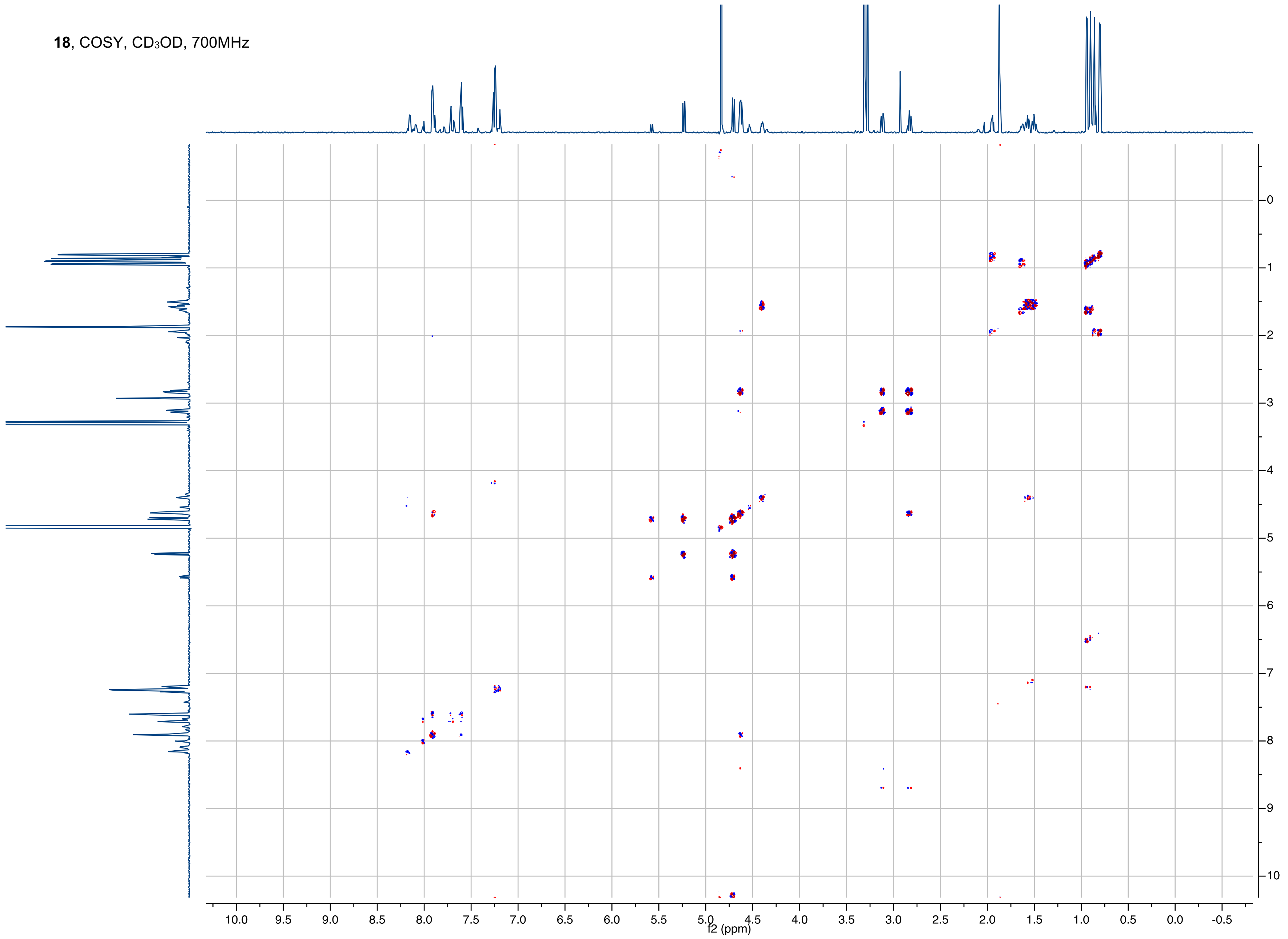

18, HSQC, CD_3OD, 700MHz

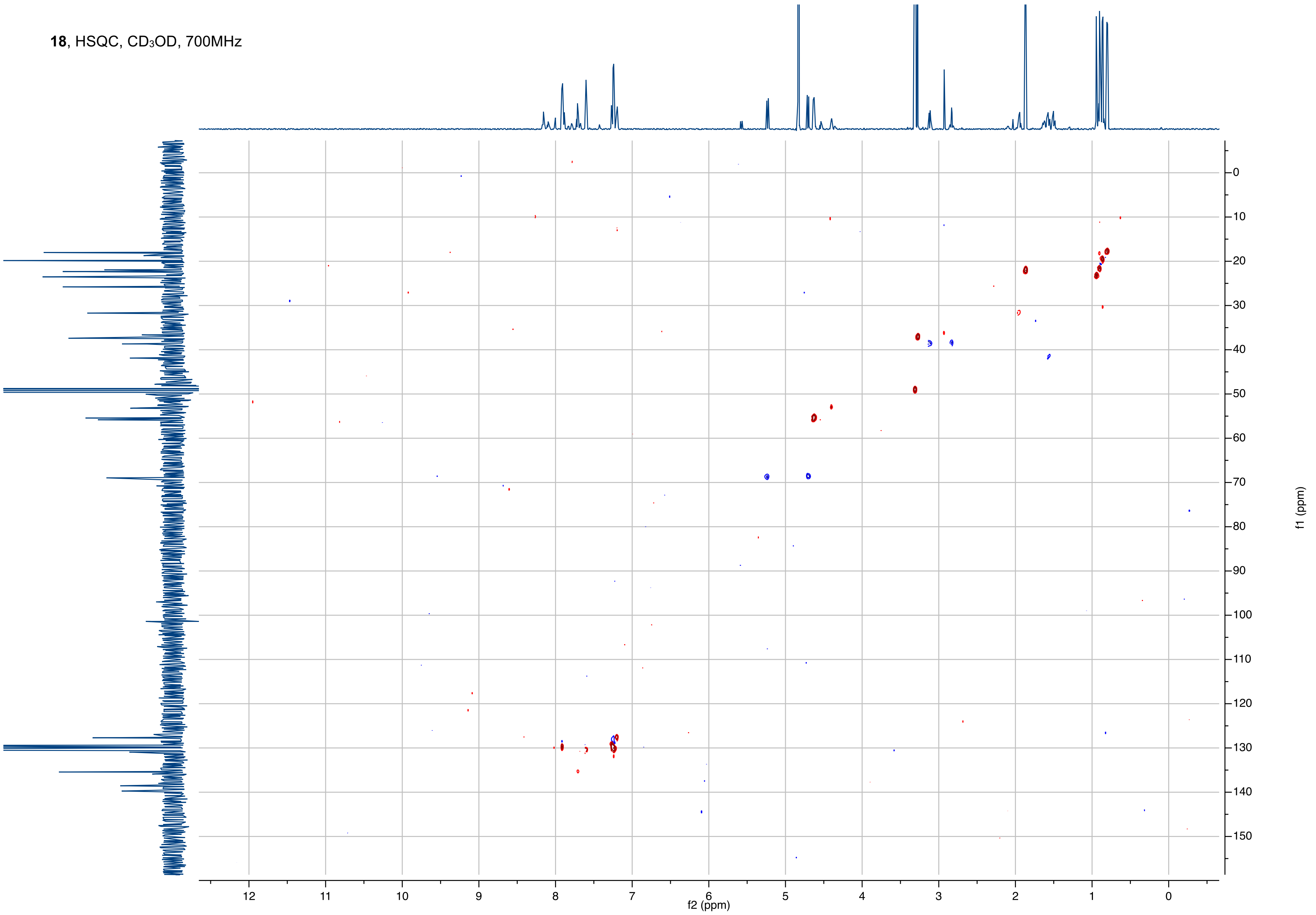

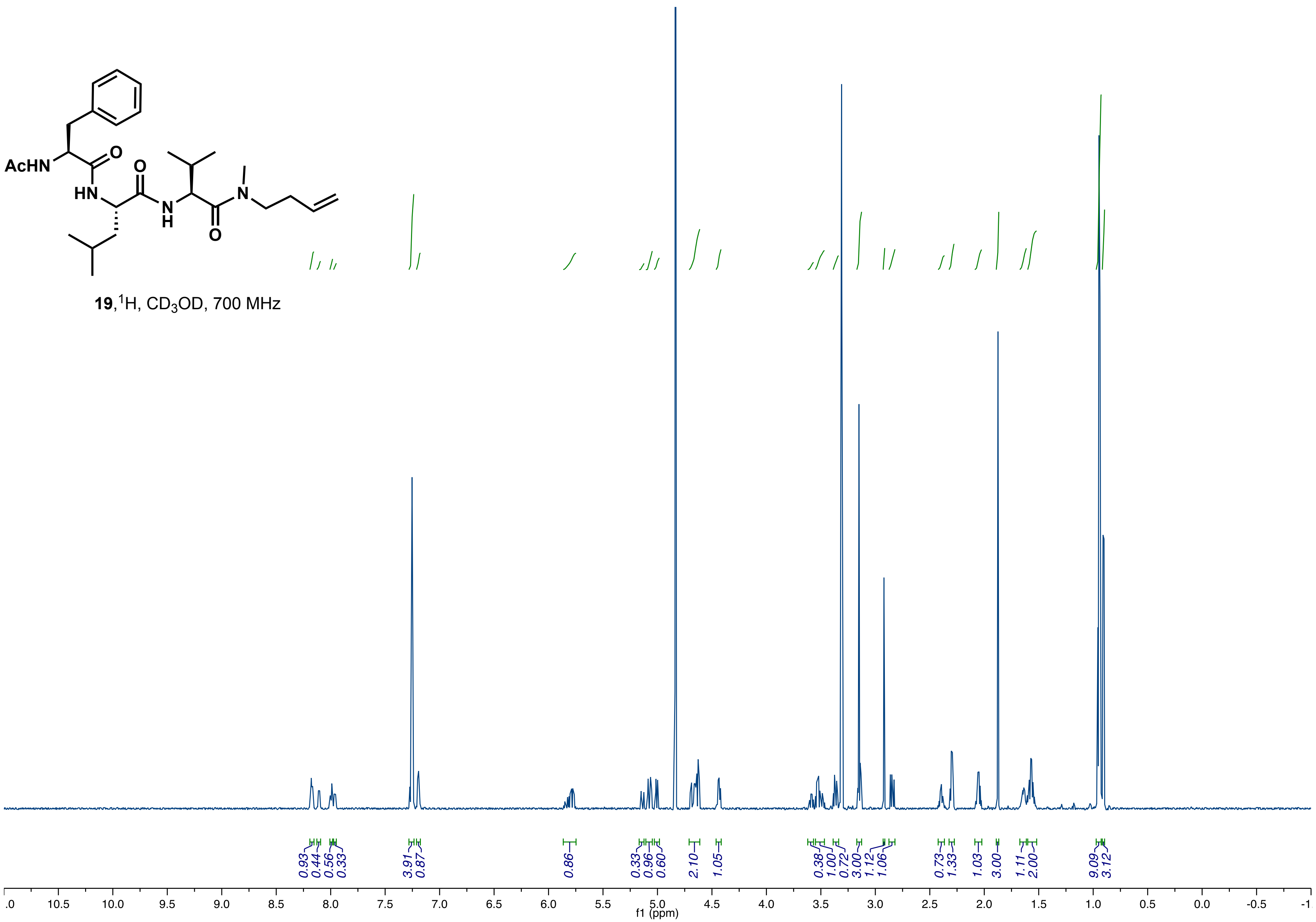

19, ^{1}H, CD_3OD, 700 MHz

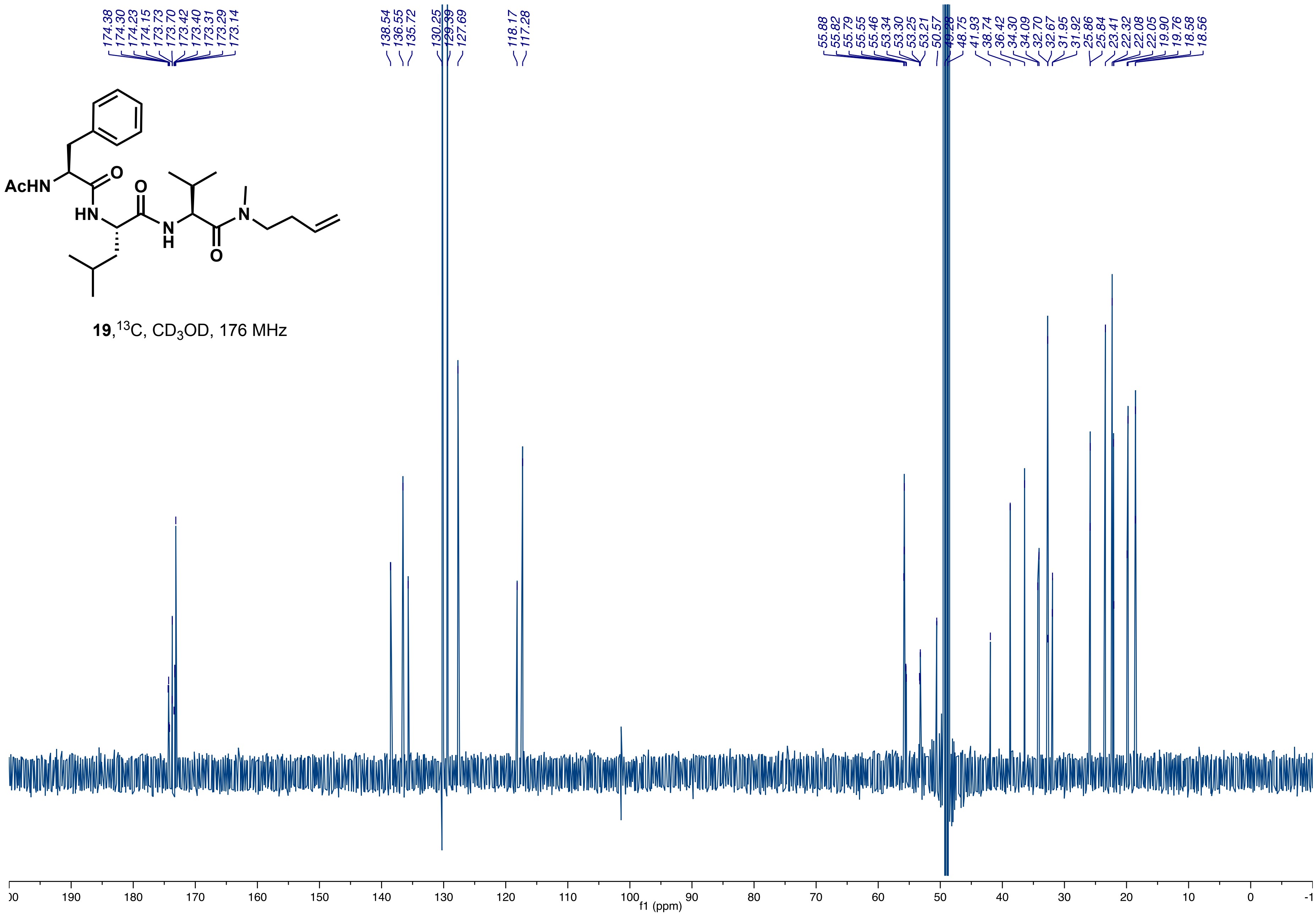

19, ^{13}C, CD_3OD, 176 MHz

19, COSY, CD_3OD, 700MHz

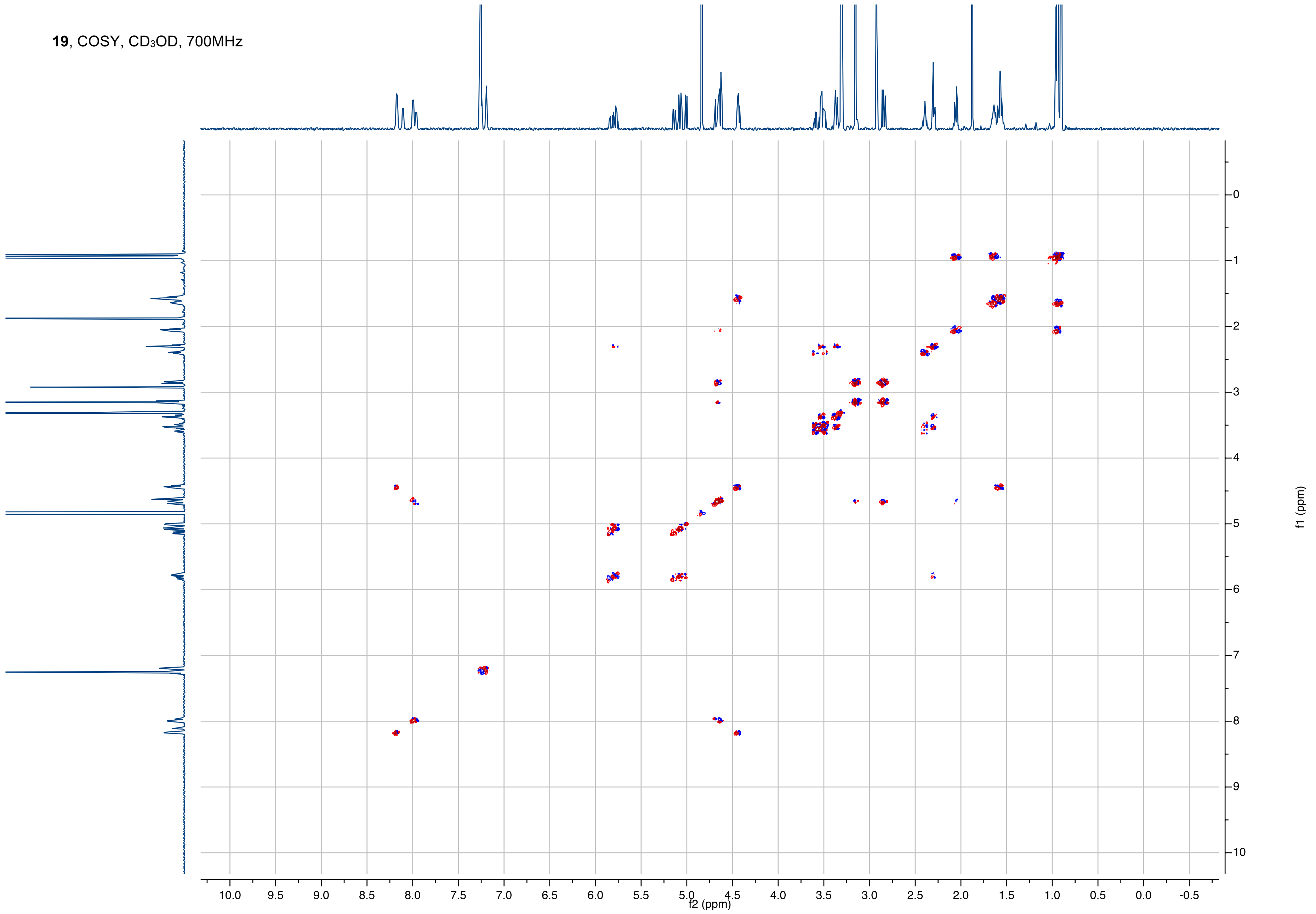

19, HSQC, CD_3OD, 700MHz

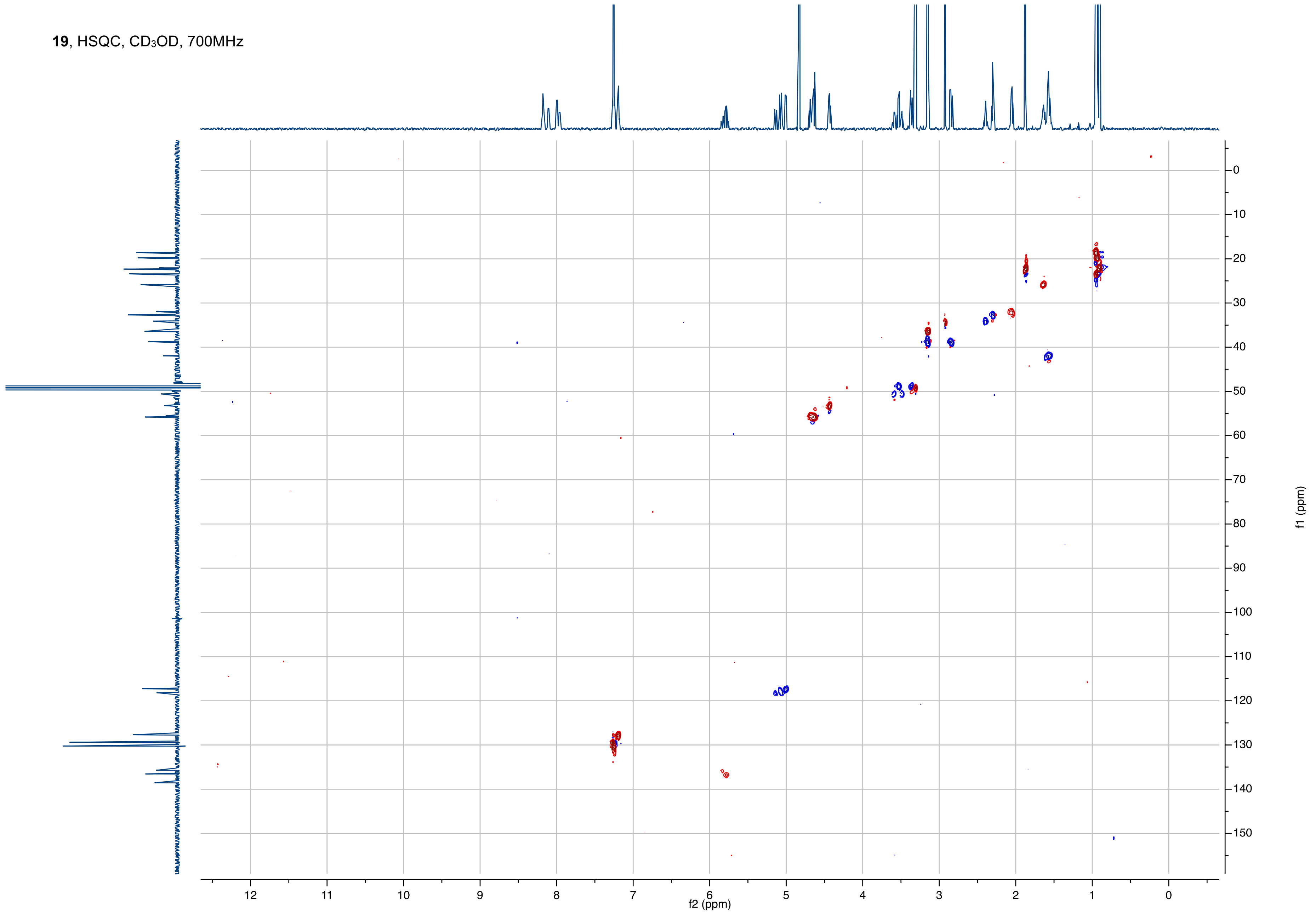

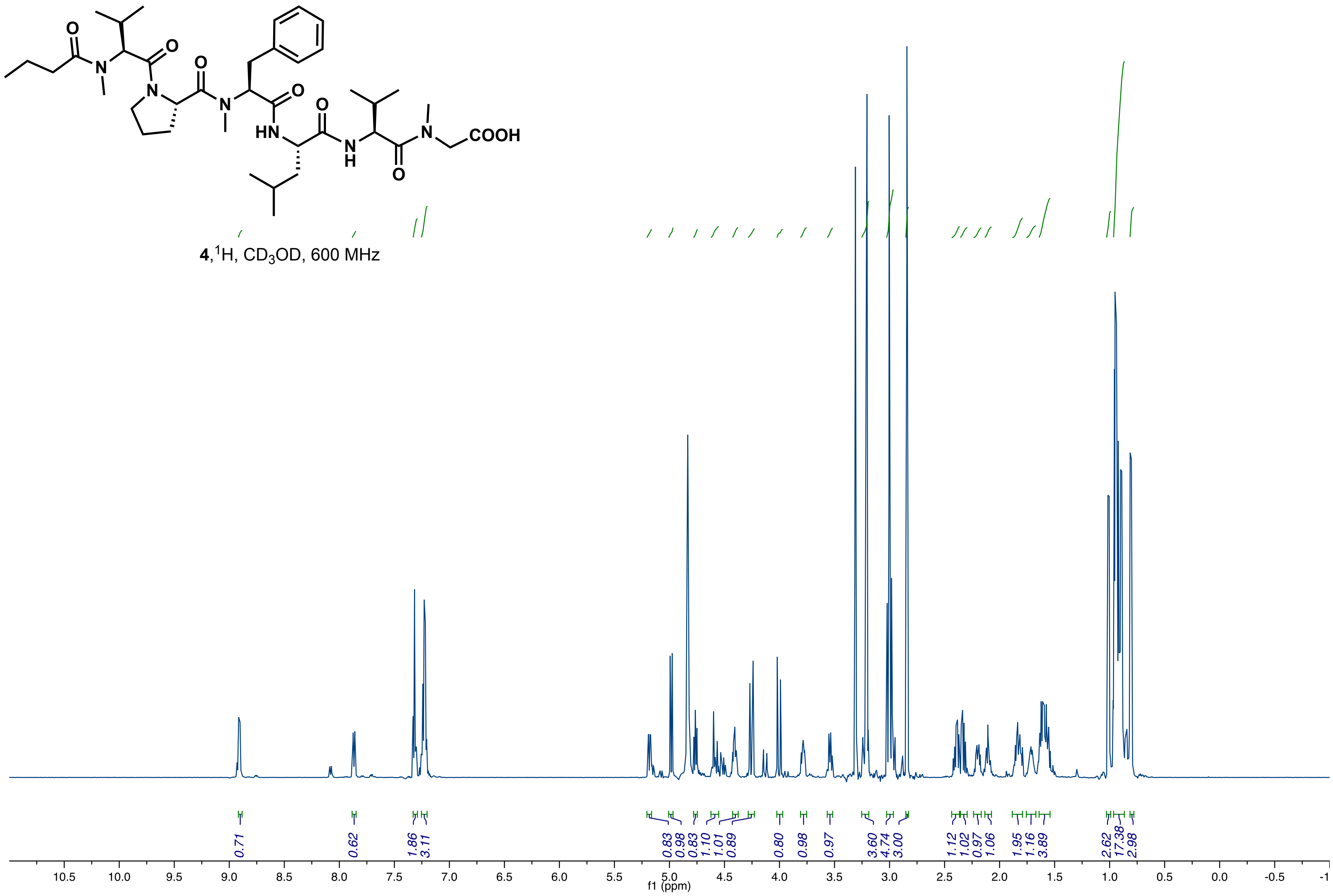

4,^{1}H, CD_3OD, 600 MHz

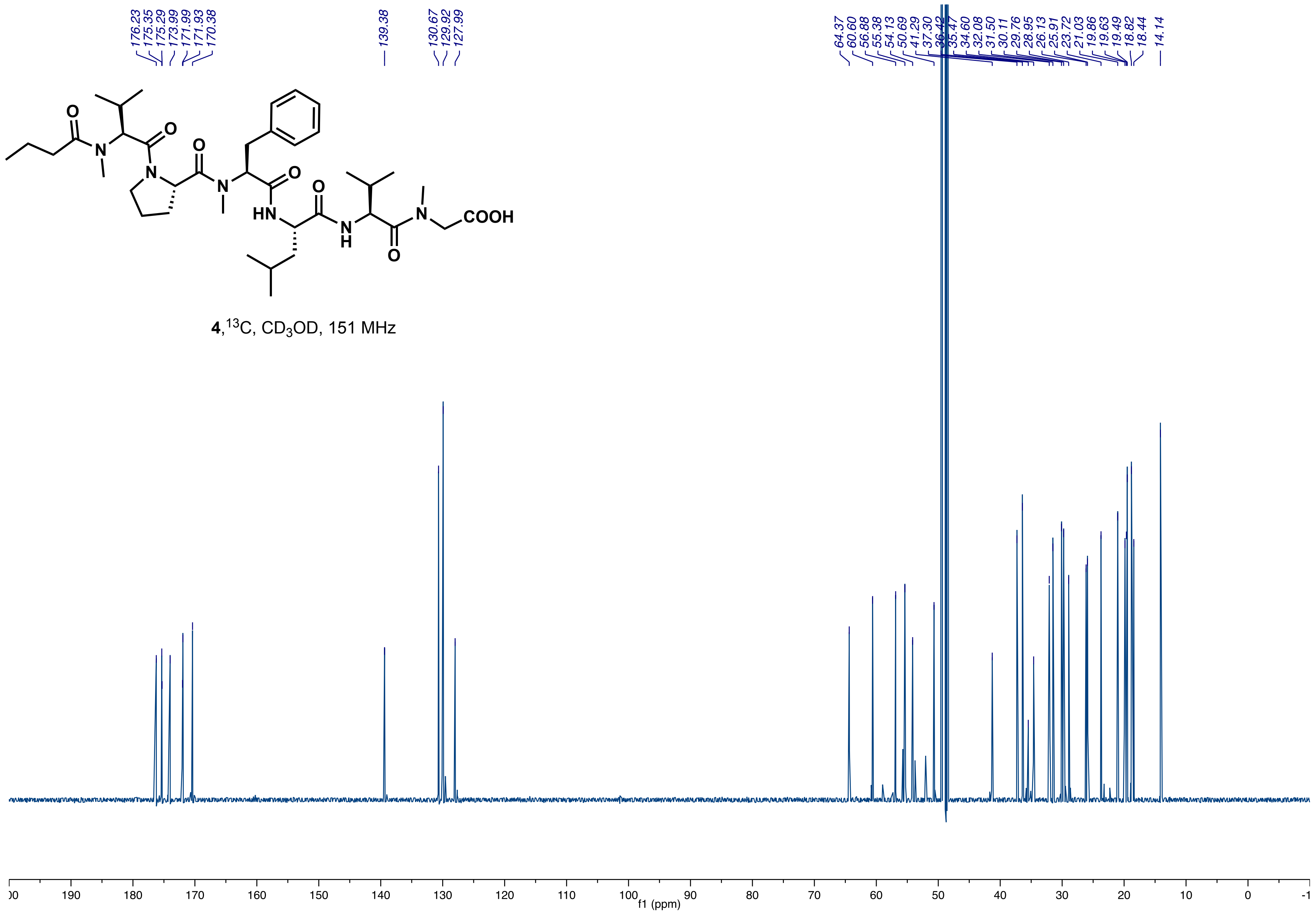

4, ^{13}C, CD_3OD, 151 MHz

4, COSY, CD_3OD, 600 MHz

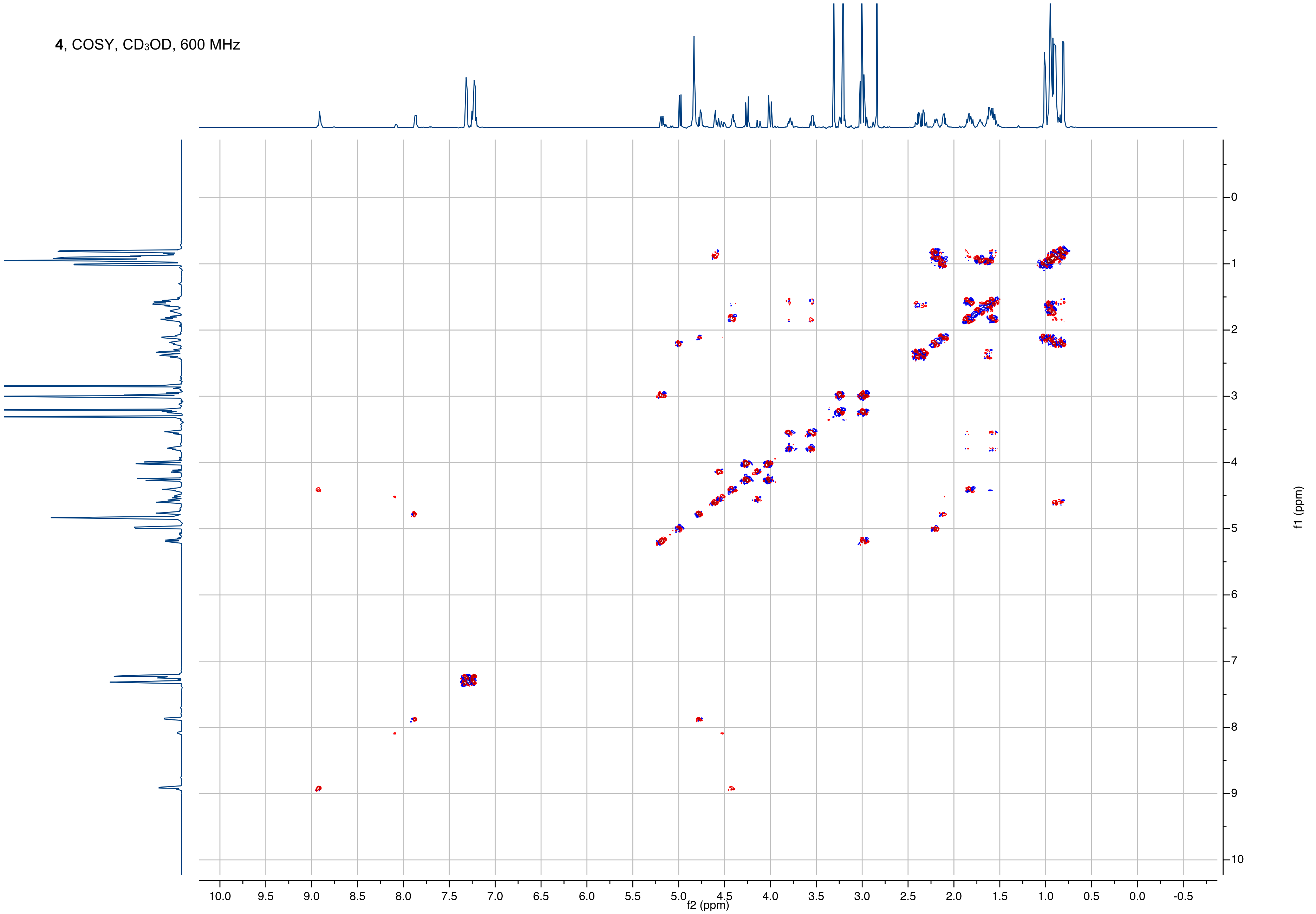

4, HSQC, CD_3OD, 600 MHz

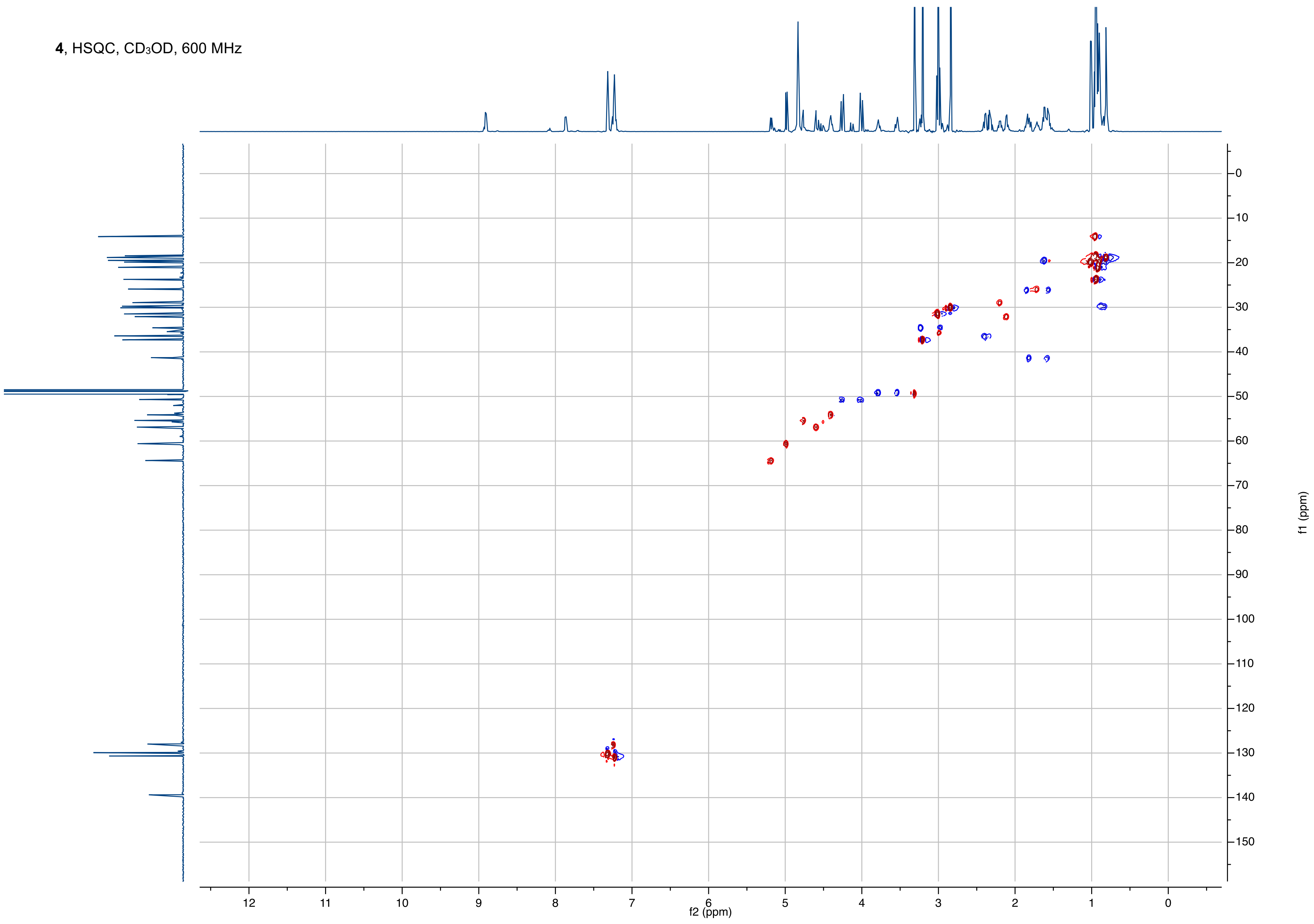

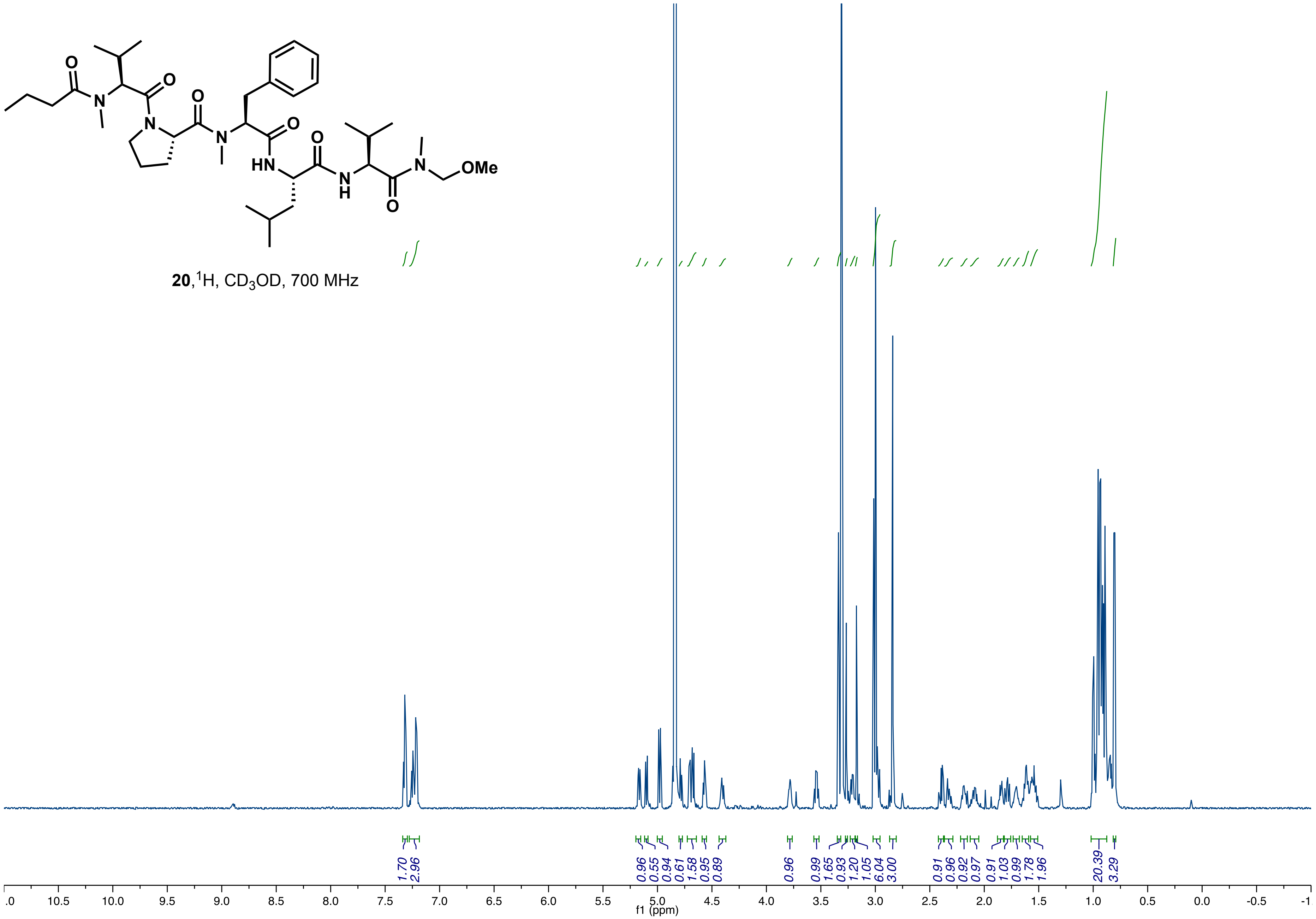

20, ^{1}H, CD_3OD, 700 MHz

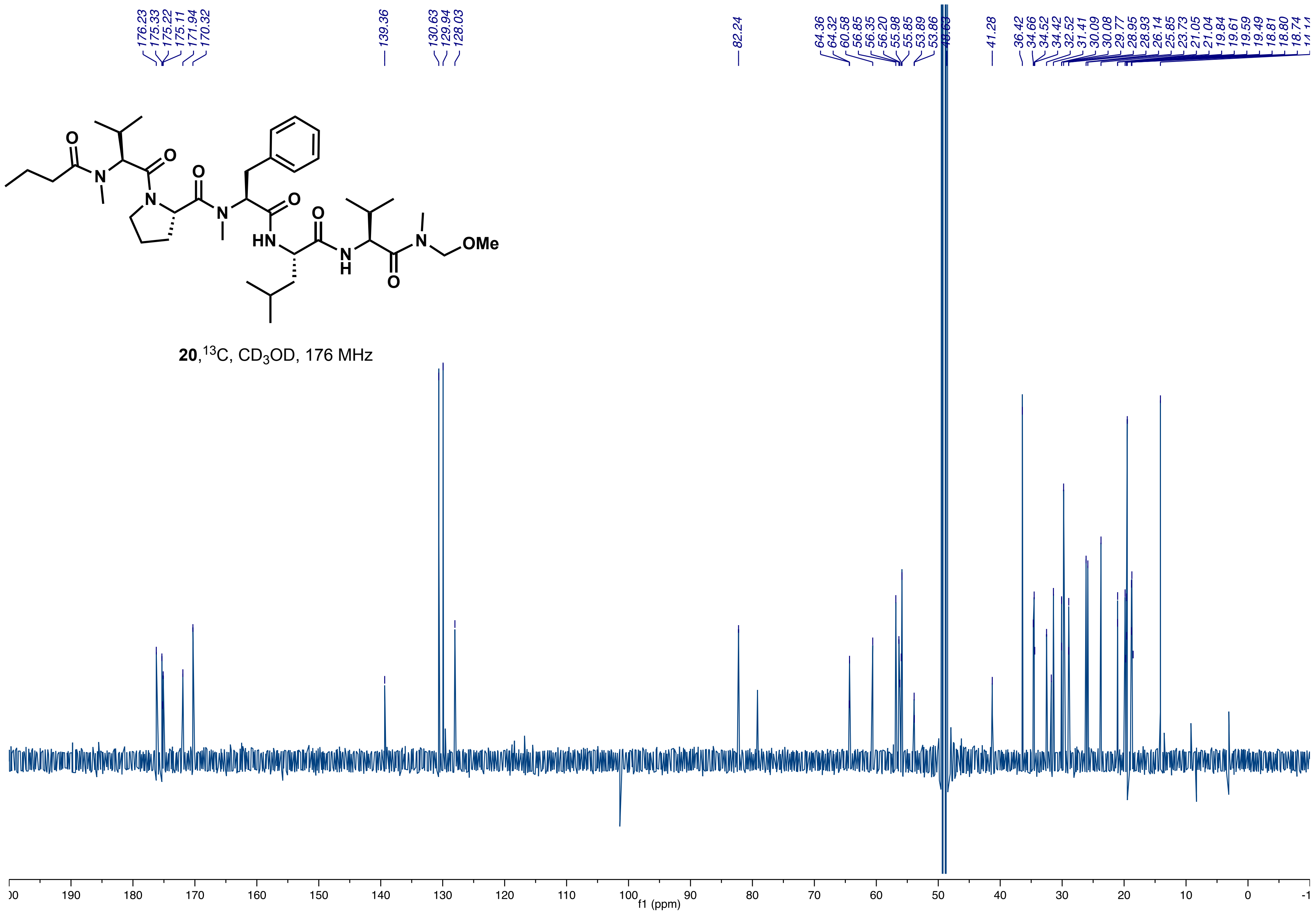

20, ^{13}C, CD_3OD, 176 MHz

20, COSY, CD_3OD, 700 MHz

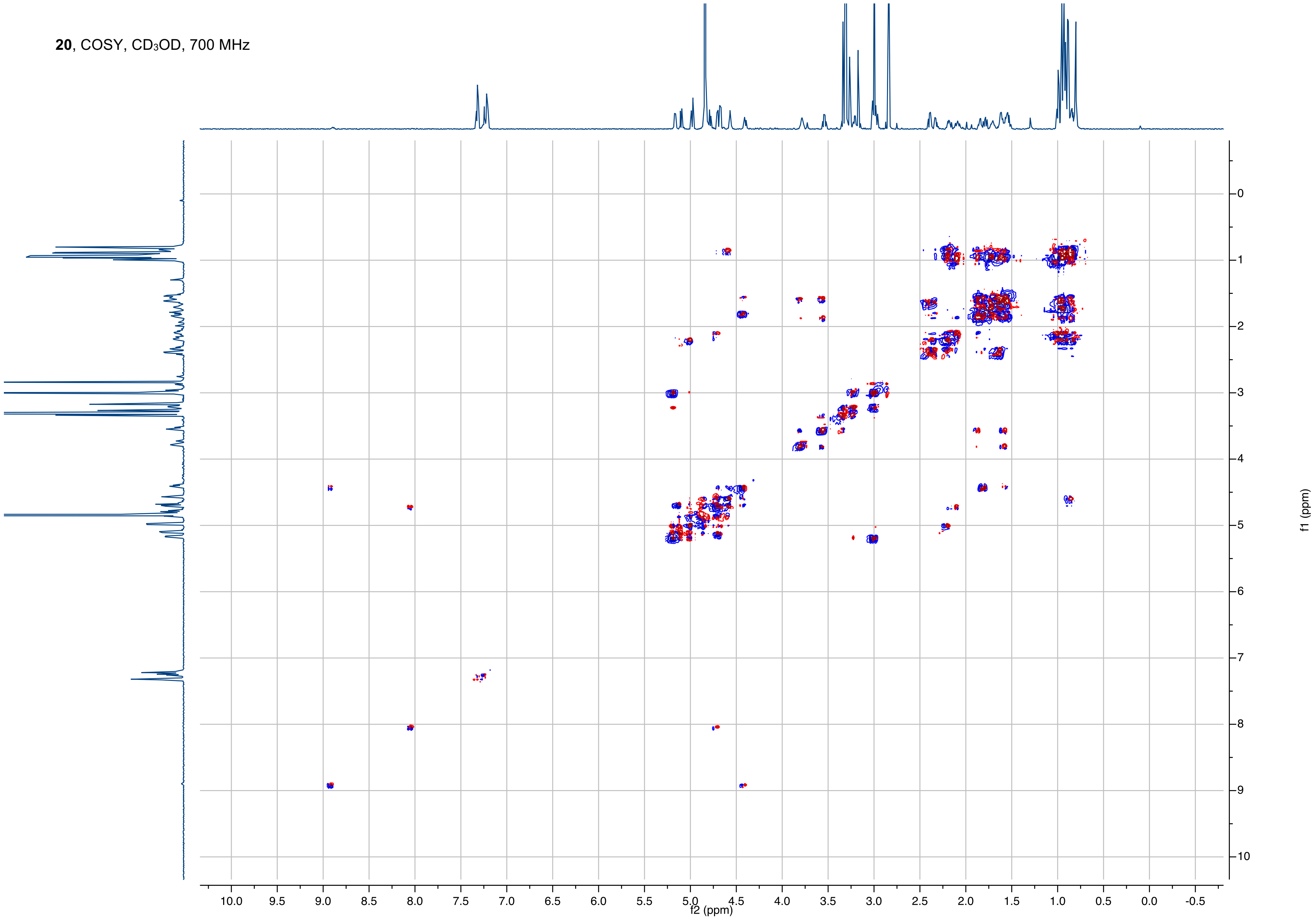

20, HSQC, CD_3OD, 700 MHz

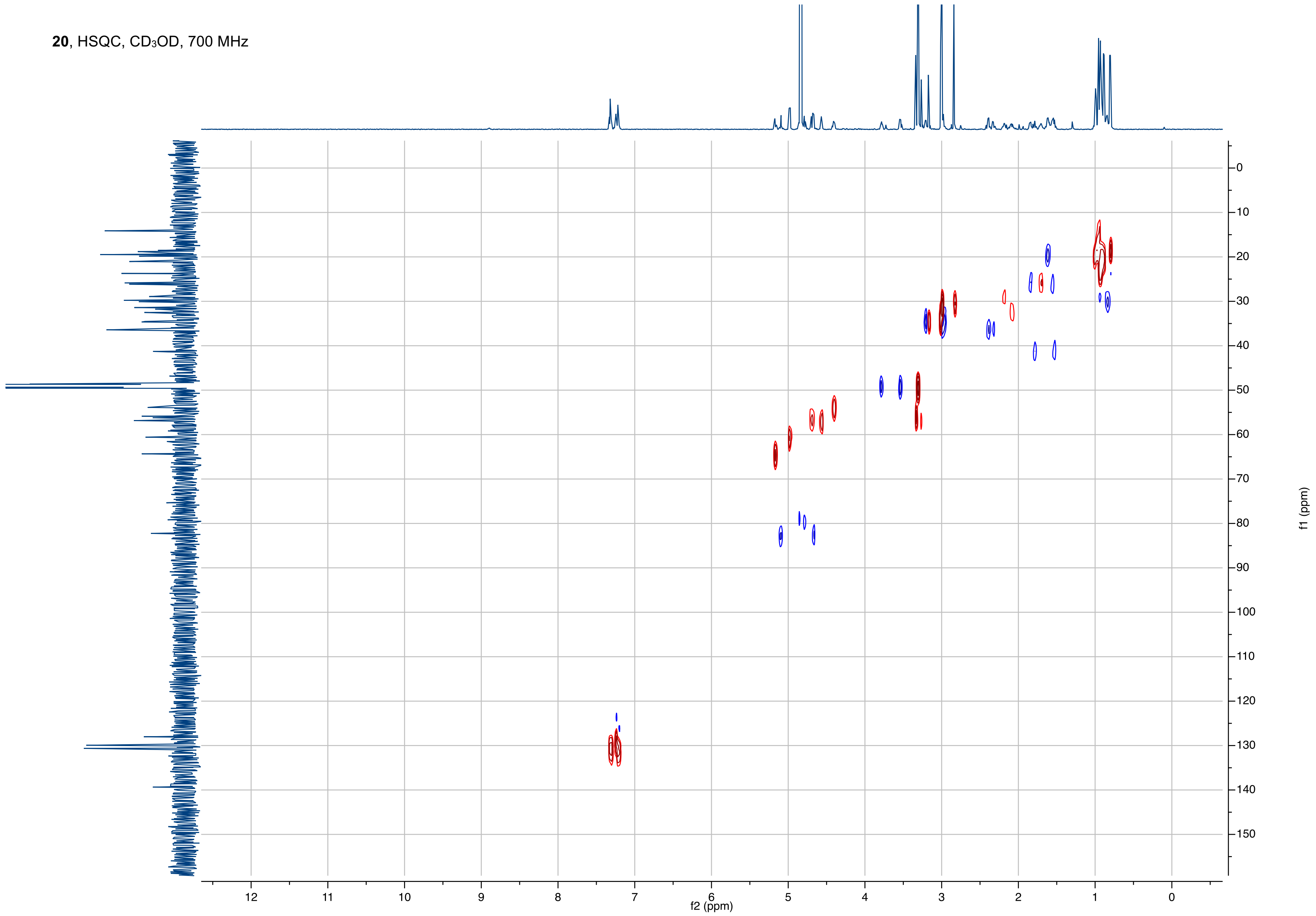

20, HMBC, CD_3OD, 700 MHz

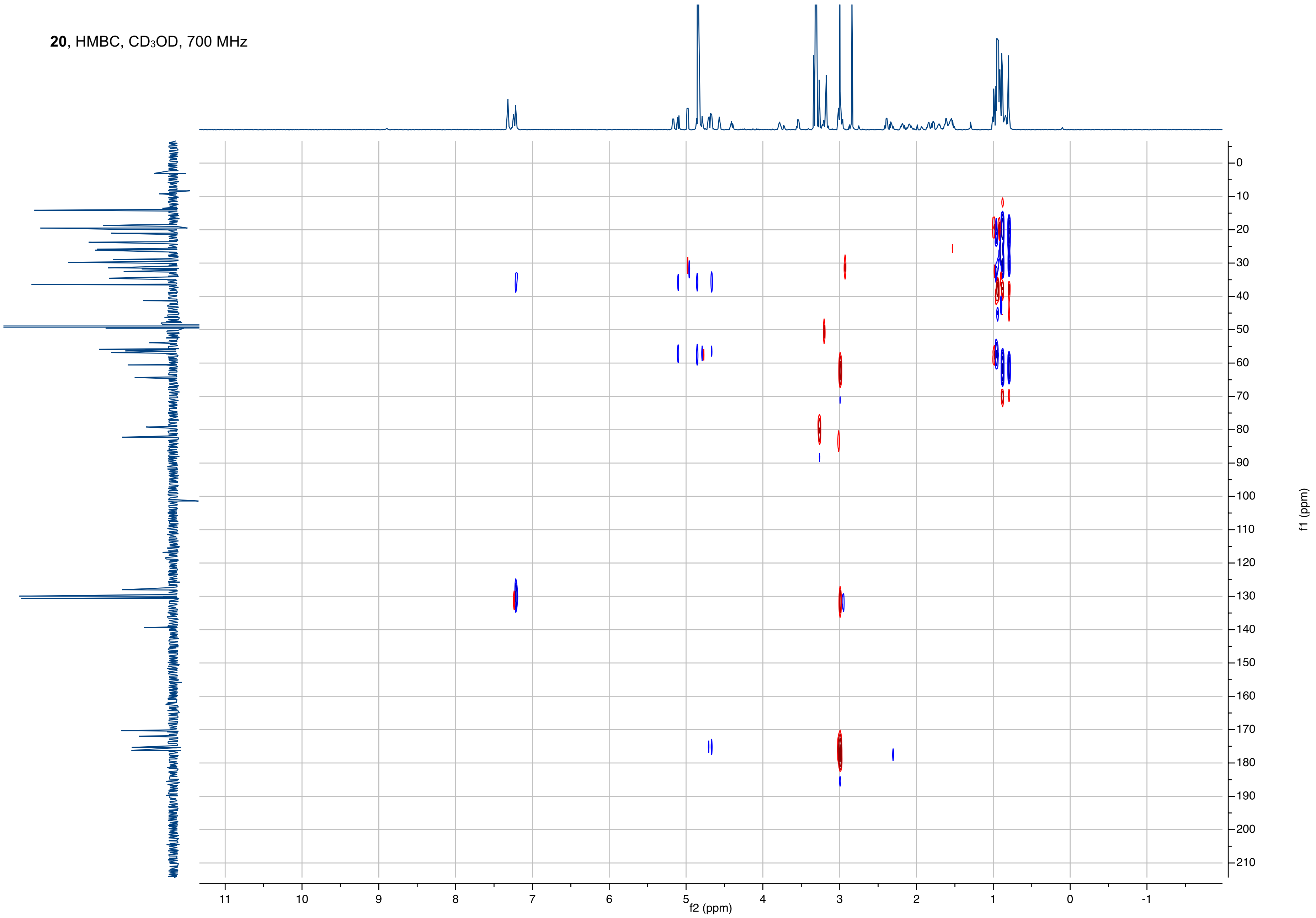

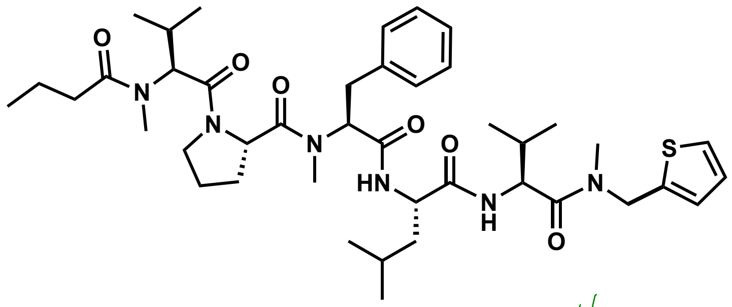

21, ^{1}H, CD_3OD, 700 MHz

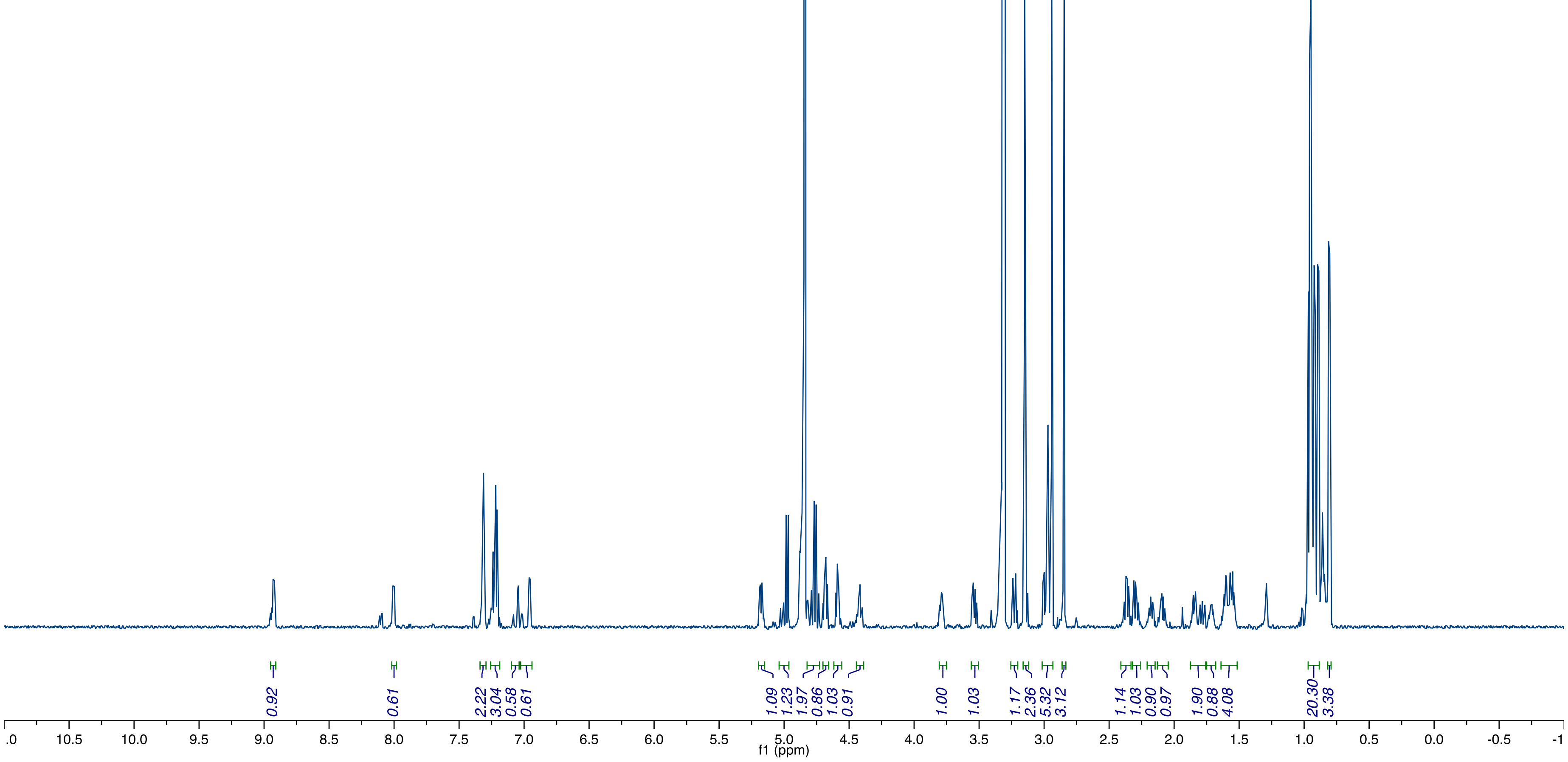

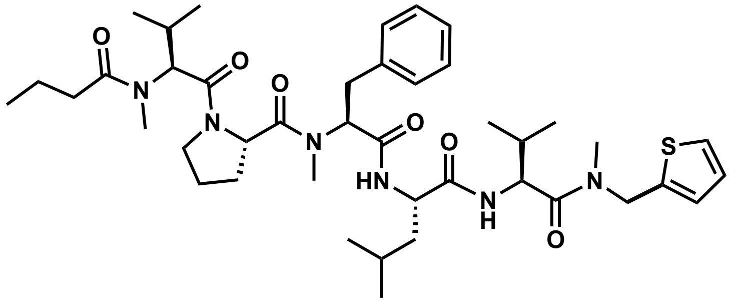

21, ^{13}C, CD_3OD, 176 MHz

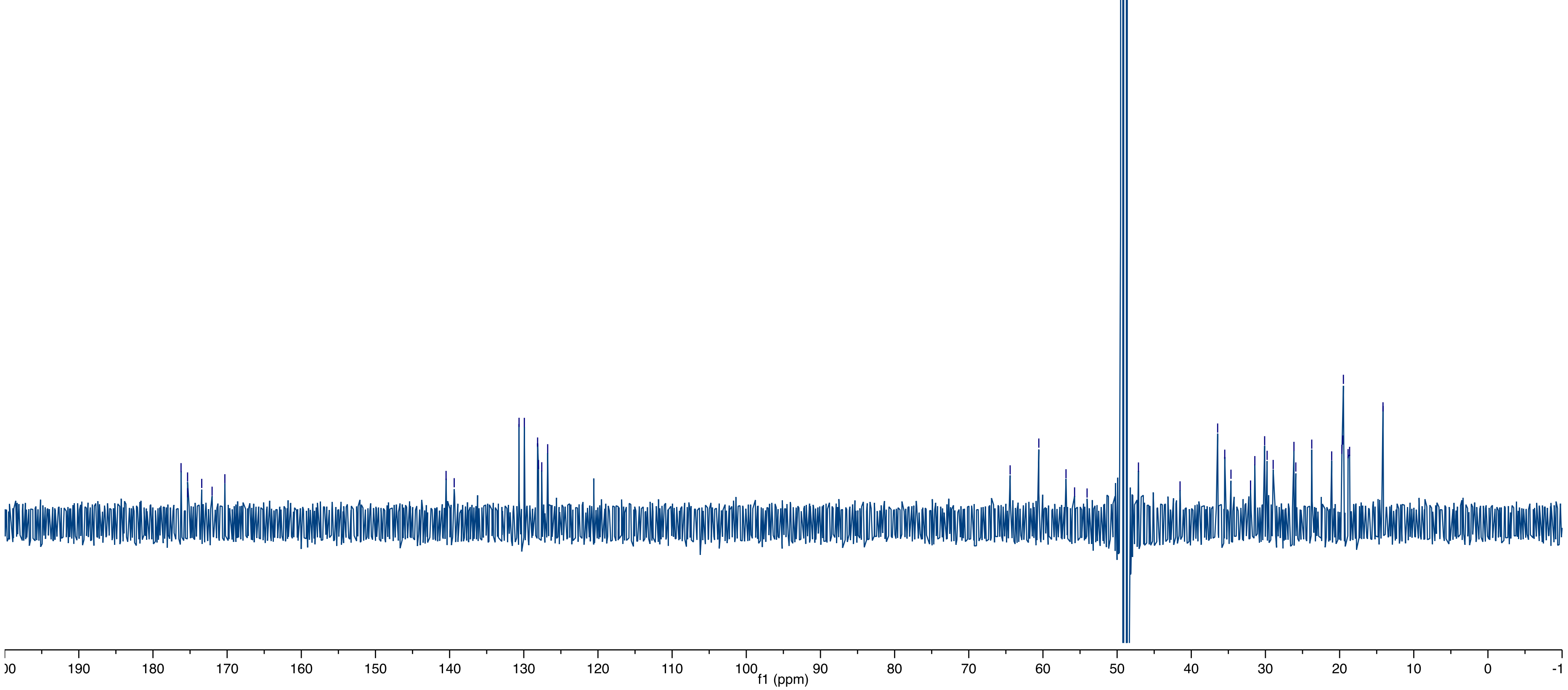

21, COSY, CD_3OD, 700 MHz

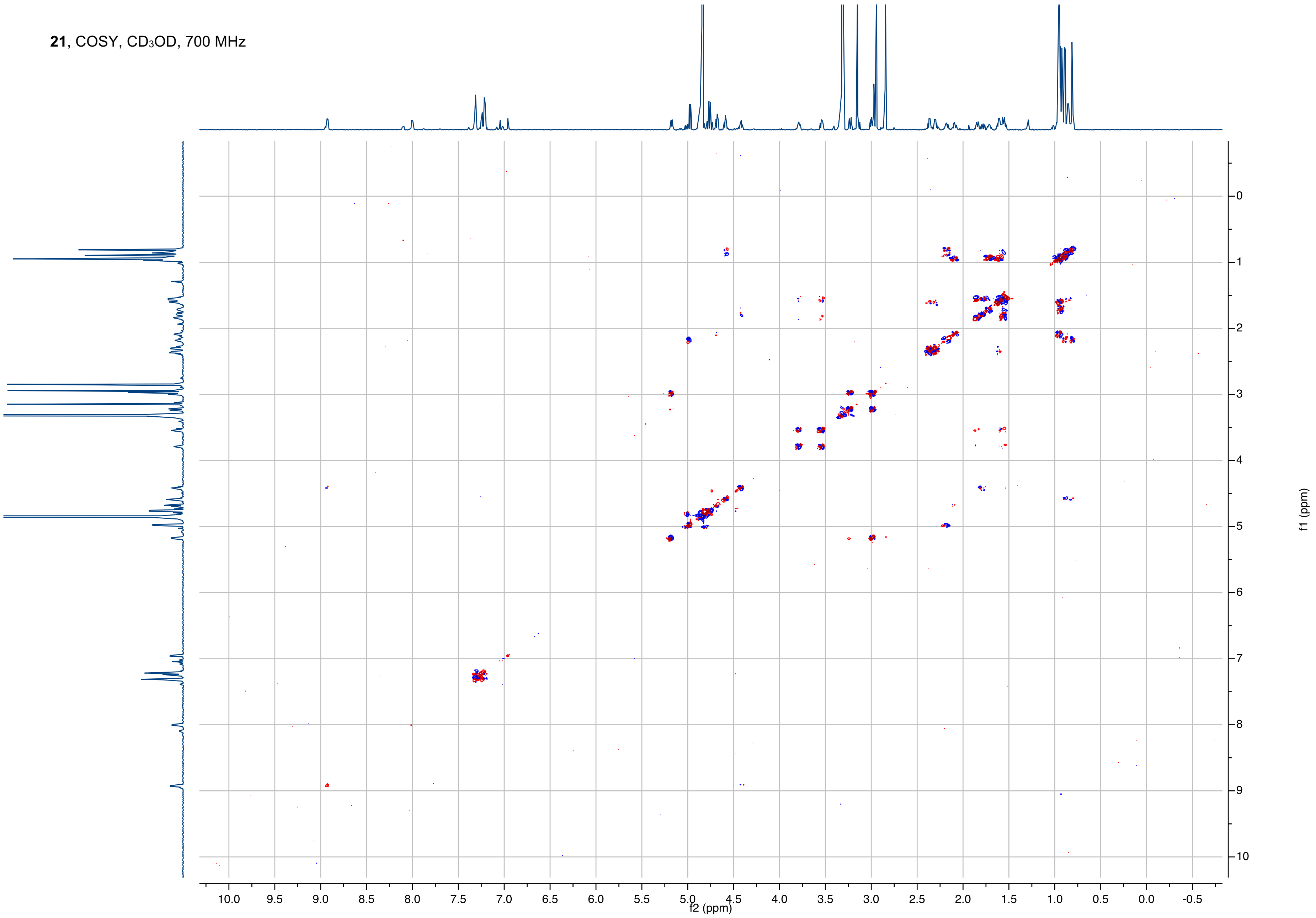

21, HSQC, CD_3OD, 700 MHz

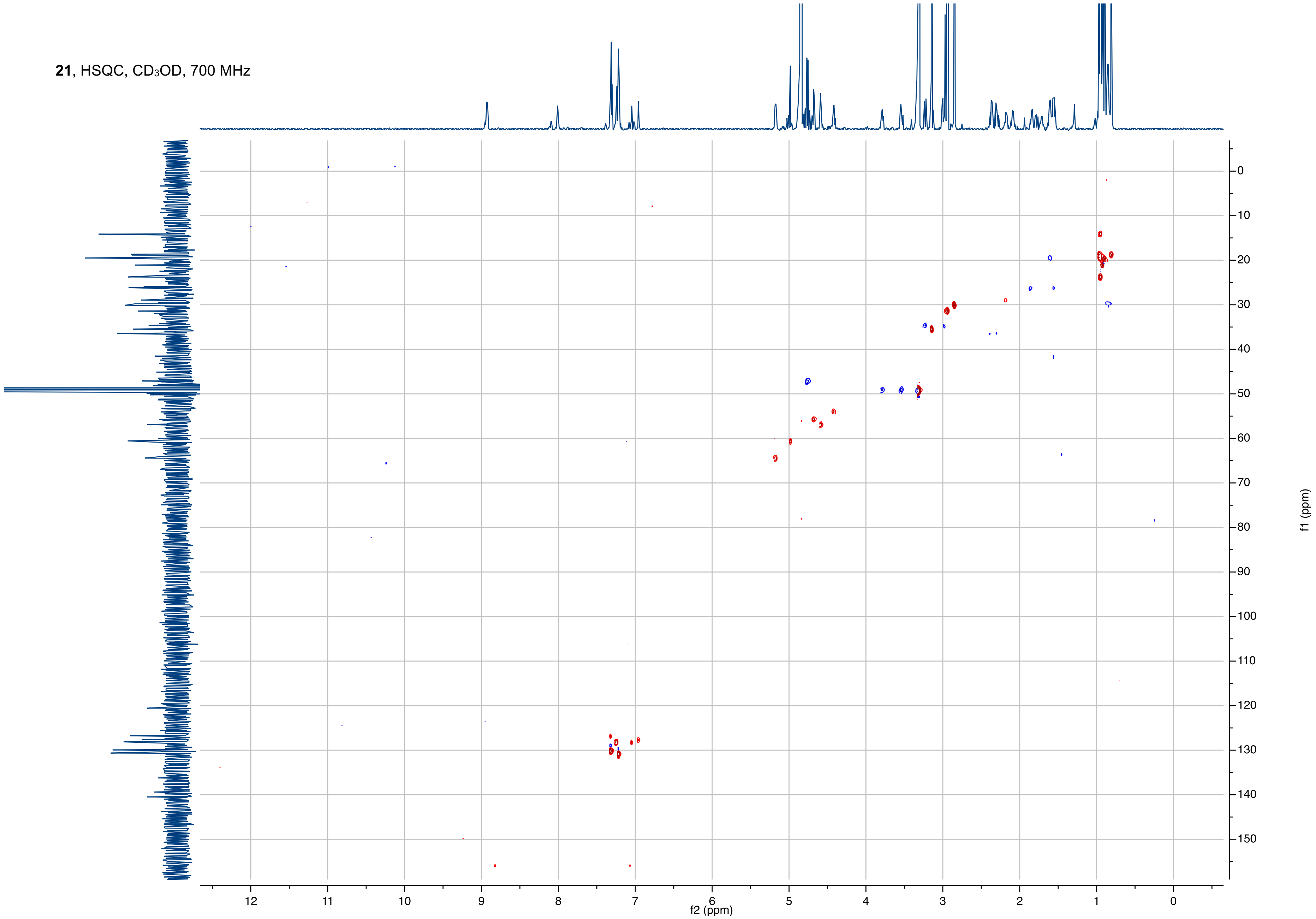

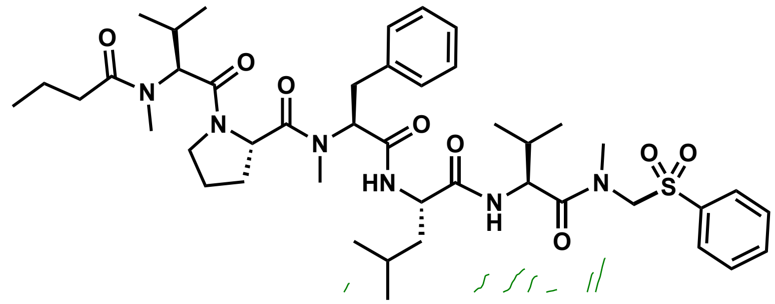

22, ^{1}H, CD_3OD, 700 MHz

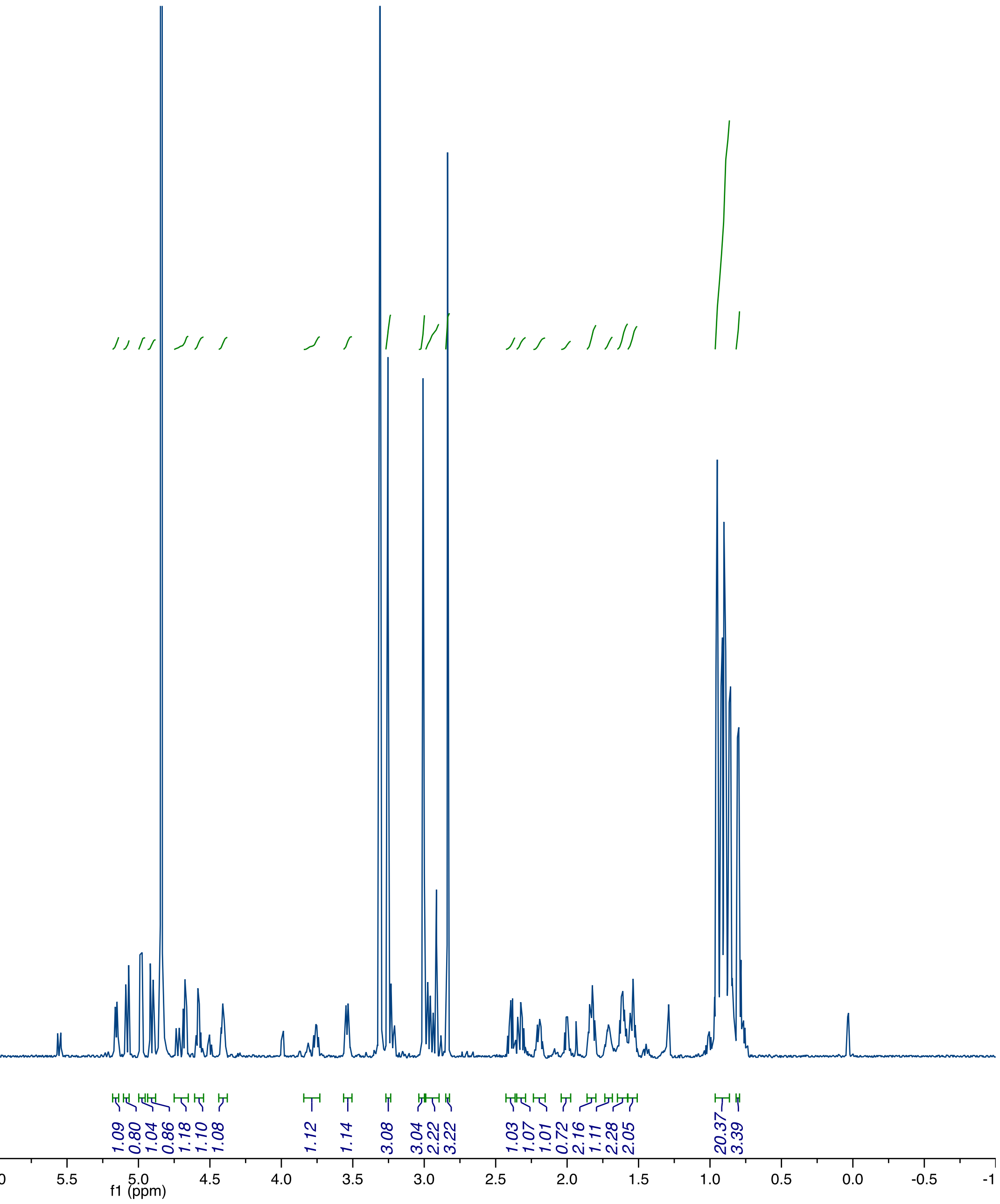

22, COSY, CD_3OD, 700 MHz

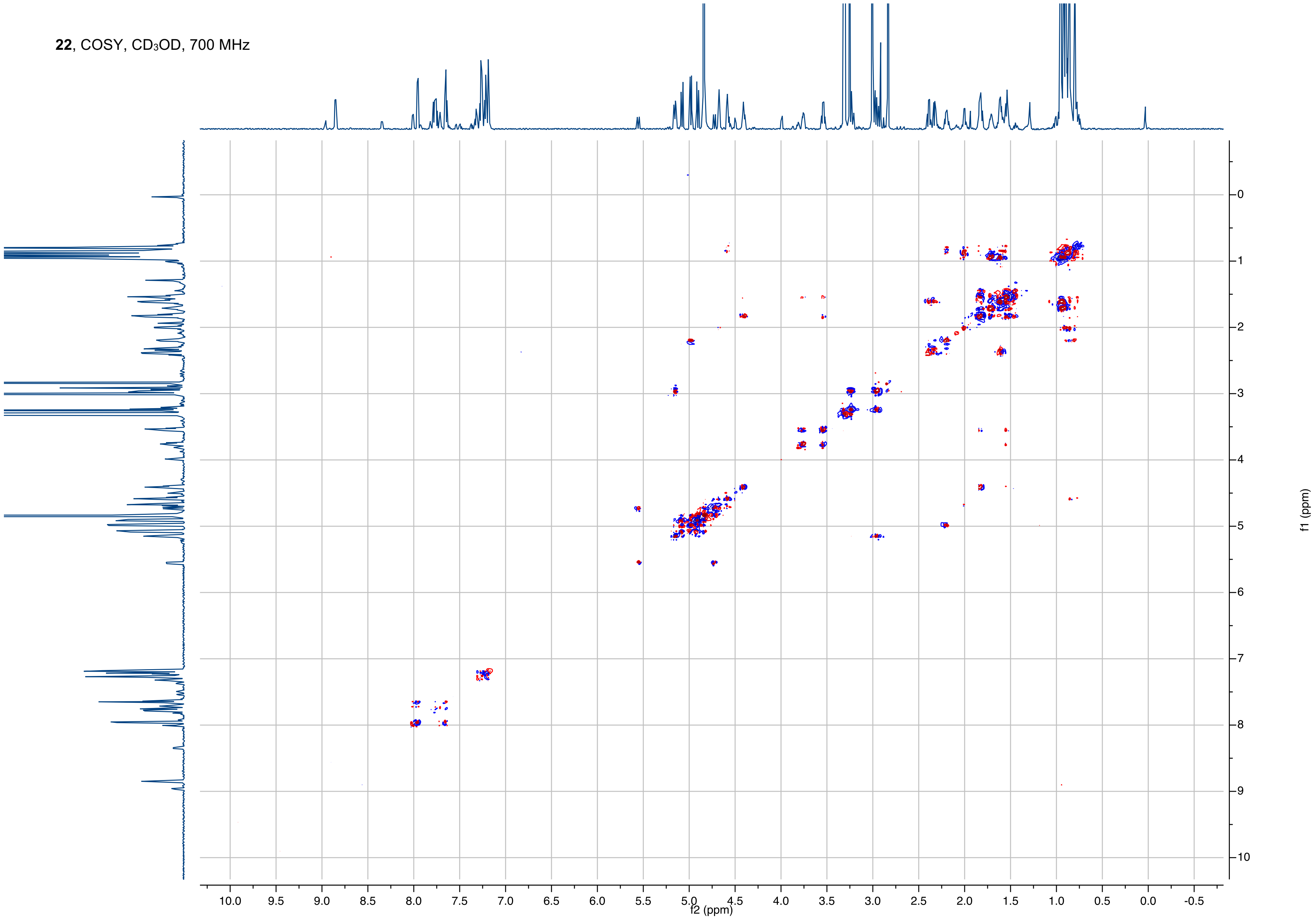

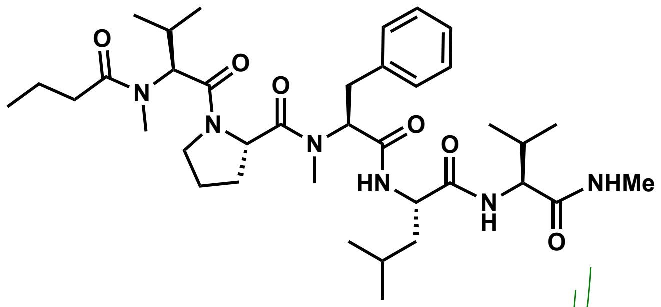

23,^{1}H, CD_3OD, 700 MHz

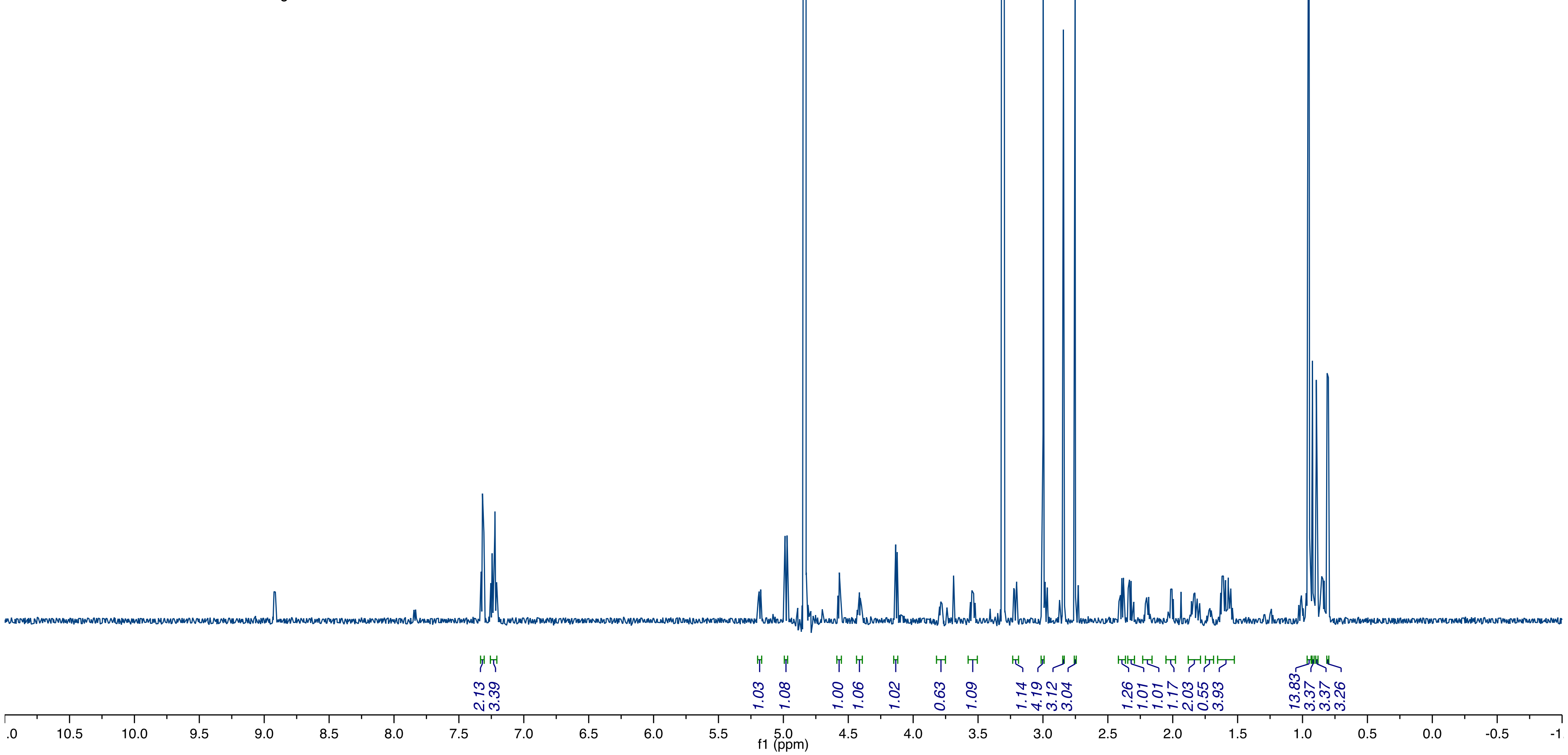

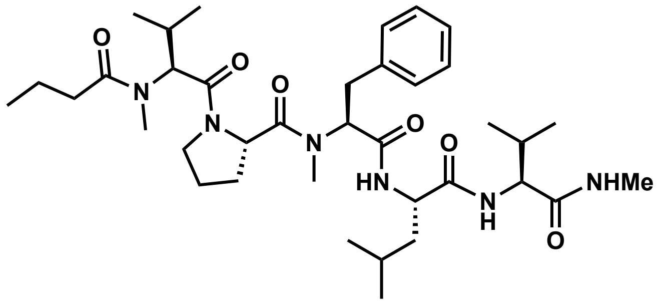

23,^{13}C, CD_3OD, 176 MHz

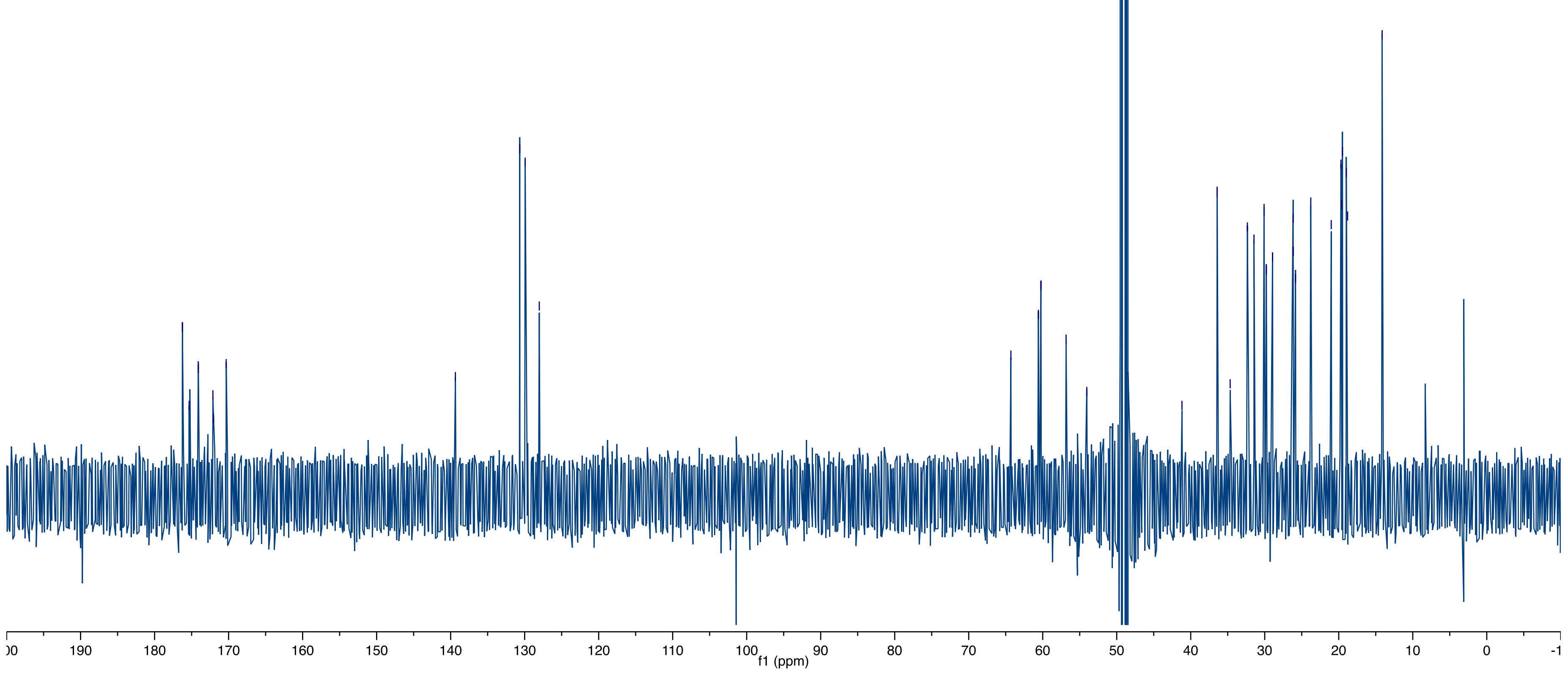

23, COSY, CD_3OD, 700 MHz

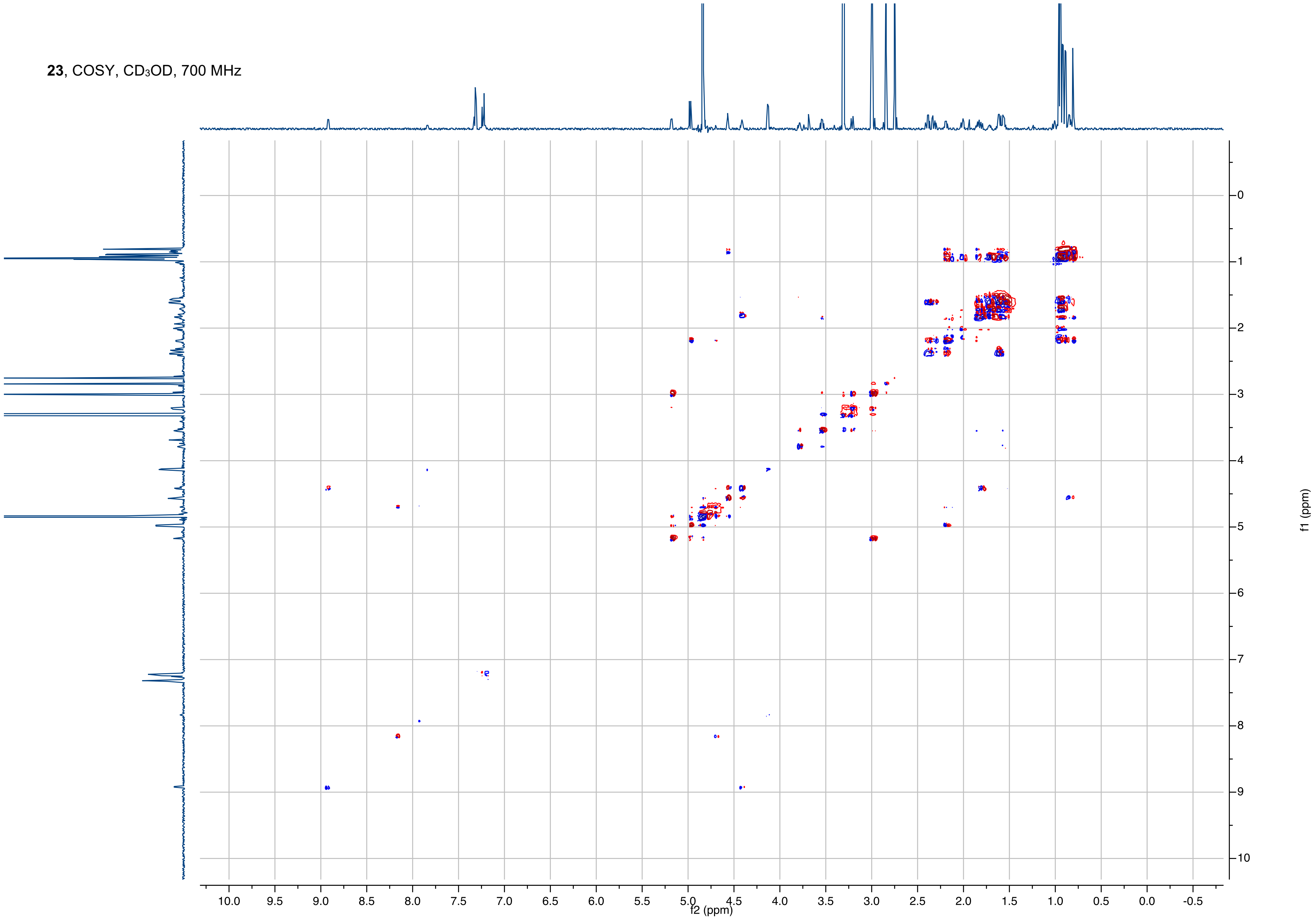

23, HSQC, CD_3OD, 700 MHz

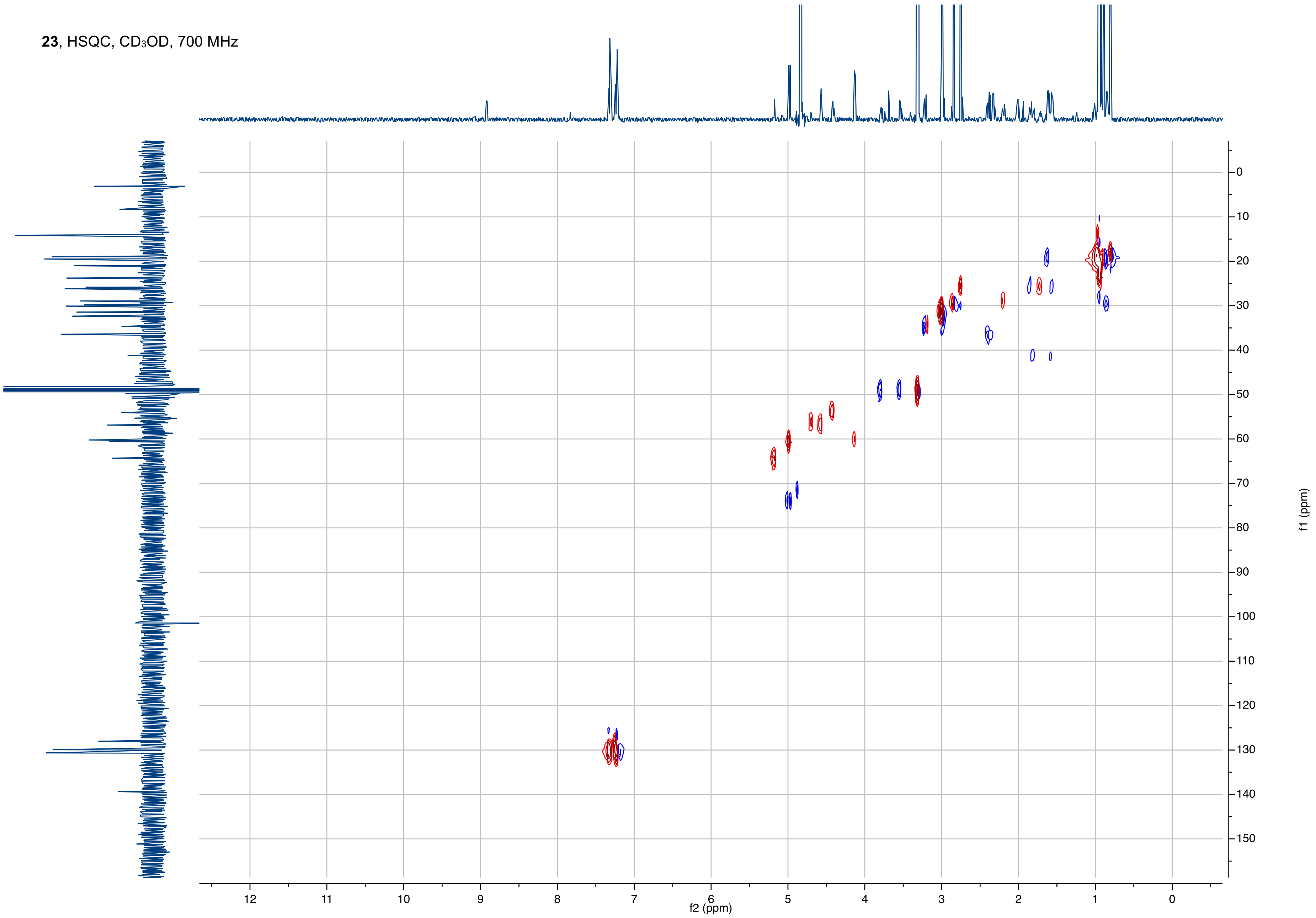

Supplement: SC-011-D0SC03701J-s001 [file SC-011-D0SC03701J-s001.pdf]
